# A combinatorial approach of Proteomics and Systems Biology in unravelling the mechanisms of acute kidney injury (AKI): involvement of NMDA receptor GRIN1 in murine AKI

**DOI:** 10.1186/1752-0509-7-110

**Published:** 2013-10-30

**Authors:** Holger Husi, Maria Dolores Sanchez-Niño, Christian Delles, William Mullen, Antonia Vlahou, Alberto Ortiz, Harald Mischak

**Affiliations:** 1 BHF Glasgow Cardiovascular Research Centre, University of Glasgow, 126 University Place, Glasgow G12 8TA,, UK; 2 Instituto de Investigacion Sanitaria IDIPAZ, Madrid 28046, Spain; 3 REDINREN, 28040 Madrid, Spain; 4 Biomedical Research Foundation,, cademy of Athens, 4 Soranou Ephessiou, 115 27 Athens, Greece; 5 IIS-Fundación Jiménez Díaz, Universidad Autónoma de Madrid and Fundación Renal Iñigo Alvarez de Toledo-IRSIN, 28003 Madrid, Spain; 6 Mosaiques diagnostics GmbH, 30625 Hannover, Germany

**Keywords:** Acute kidney injury, NMDA receptor, Pathways, Proteomics, Systems biology

## Abstract

**Background:**

Acute kidney injury (AKI) is a frequent condition in hospitalised patients undergoing major surgery or the critically ill and is associated with increased mortality. Based on the volume of the published literature addressing this condition, reporting both supporting as well as conflicting molecular evidence, it is apparent that a comprehensive analysis strategy is required to understand and fully delineate molecular events and pathways which can be used to describe disease induction and progression as well as lead to a more targeted approach in intervention therapies.

**Results:**

We used a Systems Biology approach coupled with a *de-novo* high-resolution proteomic analysis of kidney cortex samples from a mouse model of folic acid-induced AKI (12 animals in total) and show comprehensive mapping of signalling cascades, gene activation events and metabolite interference by mapping high-resolution proteomic datasets onto a de-novo hypothesis-free dataspace. The findings support the involvement of the glutamatergic signalling system in AKI, induced by over-activation of the N-methyl-D-aspartate (NMDA)-receptor leading to apoptosis and necrosis by Ca^2+^-influx, calpain and caspase activation, and co-occurring reactive oxygen species (ROS) production to DNA fragmentation and NAD-rundown. The specific over-activation of the NMDA receptor may be triggered by the p53-induced protein kinase Dapk1, which is a known non-reversible cell death inducer in a neurological context. The pathway mapping is consistent with the involvement of the Renin-Angiotensin Aldosterone System (RAAS), corticoid and TNFα signalling, leading to ROS production and gene activation through NFκB, PPARγ, SMAD and HIF1α trans-activation, as well as p53 signalling cascade activation. Key elements of the RAAS-glutamatergic axis were assembled as a novel hypothetical pathway and validated by immunohistochemistry.

**Conclusions:**

This study shows to our knowledge for the first time in a molecular signal transduction pathway map how AKI is induced, progresses through specific signalling cascades that may lead to end-effects such as apoptosis and necrosis by uncoupling of the NMDA receptor. Our results can potentially pave the way for a targeted pharmacological intervention in disease progression or induction.

## Background

A number of tightly controlled and complex processes are performed by heterogeneous cell populations of the kidney, including blood filtration by microvascular endothelial cells and podocytes and secretion and re-absorption by proximal tubular epithelial cells. Acute kidney injury (AKI) is a frequent clinical event associated with a rapid loss of kidney function, leading to high morbidity and mortality [1] in up to 22% of hospitalised patients [2]. However, it is estimated that one-fifth of AKI that occurs after hospital admission is predictable and avoidable [3]. In light of the economic burden of 0.4% to 0.6% of the total healthcare costs, corresponding to £400 m - £600 m, annually spent on treatment for acute kidney injury in the UK alone [4], a clear need for development of means to predict and/or early detect and prevent/treat AKI arises.

It has been by now widely accepted that AKI represents a continuum or spectrum of diseases that could be identified at an early stage, rather than the previous terminology of acute renal failure describing an “all or nothing” condition [5]. The recognition of renal impairment at an early stage would allow for an immediate course of action to alleviate symptoms and disrupt the process of functional decline [6], however this implies that this condition is comprehensively understood on a molecular level to allow for targeted intervention therapies.

AKI can be caused by many different events such as ischemia, vasoconstrictive drugs, exposure to toxins, hypotension linked to sepsis, and obstruction of the urinary tract, and leads to a number of complications, including metabolic acidosis, high potassium levels, uremia, changes in body fluid balance, and damage to and failure of other organs [7]. Molecular hallmarks of AKI are accumulation of free and esterified cholesterol, inflammation and inflammatory response, altered tubule dynamics leading to increased luminal sodium, hypoxia, cellular ATP depletion, renal cell apoptosis and necrosis, and hyperglycaemia, which is also a contributor to AKI (see Table 1).

**Table 1:** Hallmarks and causes defining AKI

**Event**	**Modulated associated event**	**Modulation in AKI**	**Molecular cause**
RAAS activation	Angiotensin signalling	up	Cathepsin/kallikrein/kininogen activity, blood pressure, pH
Vasoconstriction	Vasoconstrictors (endothelin, angiotensin, MMP2)	up	RAAS pathway induction, endothelial obstruction
	Vasodilators (NO)	down	NOS inhibition, reduced NO bioavailability by ROS activity
Hyperglycaemia		up	20-hydroxyeicosatetraenoic acid (20-HETE), Glycogen phosphorylase, PPARγ
Elevated blood pressure	Hypertension	up	Renin, 20-hydroxyeicosatetraenoic acid (20-HETE), mineralocorticoid receptor
Hypoxia	HIF1α	up	Induction by vasoconstriction and ECM accumulation
	NADPH oxidases	up	Induction by RAAS and PLCβ
	ROS levels	up	NAD(P)H oxidases, P450 isoforms, Xanthine dehydrogenase
	NFκB activity	up	ROS-modulated activation
	Inflammation factors (TNFα, TF, PAT1, MCP1)	up	NFκB-mediated gene expression
	Inflammation and inflammatory response	up	JAK/STAT- and NFκB-dependent gene activation, other pathways
	Atherogenesis, fibrinogenesis	up	TGFβ signalling and gene activation pathway
	ATP levels	down	Depletion by PMCA activity and others, and inhibition of de-novo ATP production
	NAD levels	down	Rundown by PARP
	Hypoxanthine levels	up	Metabolic shift from accumulated XMP, IMP and Inosine
	Necrosis	up	ROS, SOD, OH^.^, DNA damage, PARP, NAD rundown
PI3K modulation	PI3Kinase activity	down	Inhibition by Jnk and PKCα
	Insulin signalling	down	Inhibition by RAAS and PPARγ systems
Accumulation of free and esterified cholesterol	Systemic stress response	up	LIPE inhibition by PP1
Na^+^/Cl^-^ retention, increased luminal Na^+^	Aldosterone/cortisol signalling events	up	Mineralocorticoid/Glucocorticoid-receptors, acting on Na^+^/Cl^-^ pumps
Ras activation	Ras signalling	up	Mineralocorticoid/Glucocorticoid-receptor-dependent gene activation, Angiotensin receptor signalling
Cytoskeletal reorganization	ECM remodelling	up	Hsp27, ATP depletion, Rac1 activation, Ras mediated events
Tubular cell dynamics	Infiltration of immature cells	up	Pro-apoptotic signals

Clinical and disease model observations were analysed based on modulated associated events, directionality in terms of in- or decrease, and plausible molecular causes.

The factors causing AKI and their interaction with each other are still incompletely understood, which is surprising in light of the vast amount of scientific reports in this area. However, a systematic and in-depth mining of the literature, as employed by a comprehensive Systems Biology approach, coupled with high-resolution mass spectrometry analysis of diseased tissue could pave the way forward. Systems Biology, or integrative biology, uses descriptive terms to understand complex interactions based on the analysis of network behaviour and dynamic aspects. Here, we demonstrate a comprehensive analysis of AKI induced in a mouse model system to investigate and identify key signalling cascades, inferred metabolic alterations and molecules modulated in this highly complex and clinically relevant disease. Nevertheless it should be noted that animal models of AKI might not reflect this clinically critical condition observed in man, where an unobserved laps of mere hours between onset and catastrophic irreparable kidney failure can mean life or death. An acceptable approximation is the model chosen here, where an single overdose of folic acid causes the same end-effect of kidney damage observed in clinically manifested AKI.

## Results

**Figure 1:**
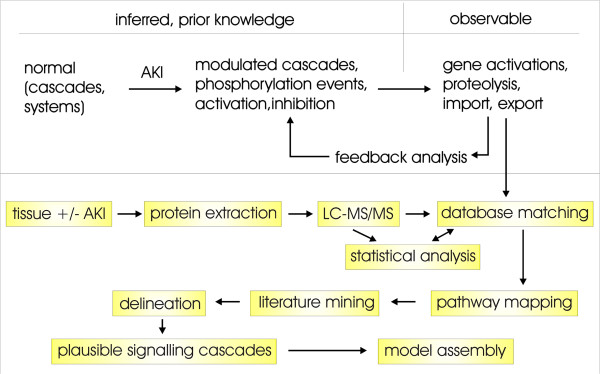
**Workflow of AKI sample analysis.** Systems Biology approaches require prior knowledge and/or inferred pathways from independent sources used to map measured data (top). In this work these sources include an extensive novel proteomic dataset, and that has undergone rigorous statistical analysis. The predicted affected pathways are then mapped further by extensive manual literature mining and delineation to establish plausible cascades and pathway maps, followed by integration of all data to assemble a final model. Re-iterations between each and all steps between pathway mapping and model assembly ensure a best-fit model for both the de-novo data as well as prior knowledge.

We first established a high-resolution proteomic map of protein expression profiles using 6 sham injected controls and 6 folic acid overdosed mice. Folic acid nephropathy is a well established murine model of AKI using a single intraperitoneal injection overdose of folic acid and harvesting of kidneys after 24 hours [8]. Kidney cortex proteins were extracted, trypsin-digested, and analysed by high-resolution liquid chromatography mass spectrometry (LC-MS/MS). After merging the individual independent mass-spectrometric analyses, 6564 non-redundant proteins were detected (Additional file 1: Table S1, and detailed information of data processing can be found in Additional file 2). 14 of these proteins were detected only in control samples, 1050 only in AKI, and 5500 in both sets of conditions. Statistical testing using Mann–Whitney showed that 2521 proteins were statistically significant (p-values <0.05), and when also taking fold-change calculations into considerations then the dataset indicated 1480 molecules to be significantly changed in AKI (p-values <0.05 and fold changes >2). 420 of these proteins were found in both control and AKI samples, 14 only in controls, and 1046 molecules only in AKI samples. After initial database matching and employing gene ontology clustering to assess data quality and overlapping characteristics, the data were mapped onto existing general pathway maps to identify affected cascades and modulatory events. More than 50 signalling cascades (see Additional file 2), mainly due to overlapping features in the existing maps, were initially identified to be of interest in further downstream analysis based on their potential functional relevance. In parallel, we assembled potential pathway maps derived from the pertinent AKI-focussed literature. Through a manual iterative feedback analysis and deep-mining of inferred and prior knowledge (Figure 1), extending the pathway analysis beyond the reported AKI-signalling cascades, and molecule-by-molecule pathway delineation and manual feature look-up, we assembled plausible signalling cascades which led to a combined pathway map (Additional file 3: Figure S1). The same approach was also used to map the metabolic pathways modulated in AKI, as well as probable gene activation cascades based on reported modulation of transcription factors such as NFκB, which were integrated into the AKI model.

**Figure 2:**
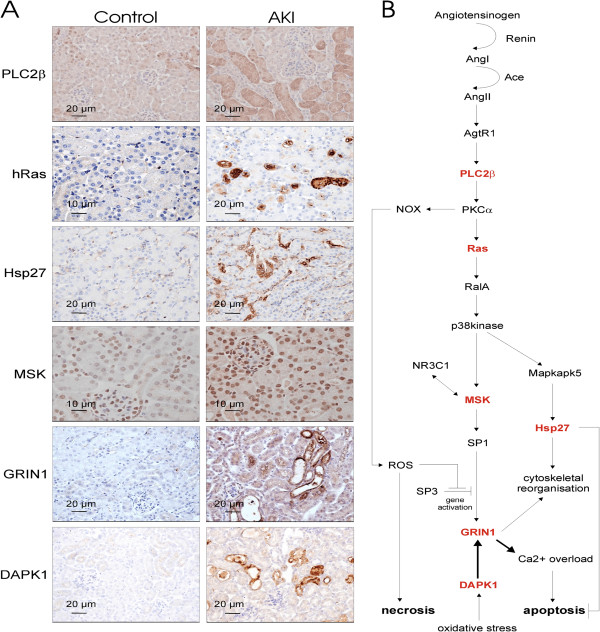
**Immunohistochemistry and pathway analysis of AKI modulated pathways along the RAAS/glutamatergic axes. (A)** Molecules of interest found and/or predicted to be differentially expressed based on the proteomics data and subsequent pathway analysis, were verified by immunohistochemistry of kidney tissue. **(B)** These proteins were delineated into specific signalling cascade involving the angiotensin to p38kinase downstream signalling and NMDA-R1 (Grin1) pathway. All six molecules tested (highlighted in red) showed an up-regulation as measured by mass spectrometry experimentation in AKI samples and potentially validates the proposed signalling cascade. Original magnification of immunohistochemical microscopy is ×20 (MSK and hRas control ×40 in order to observe the intense nuclear staining of kidney epithelial cells), black bars in the panels are 10 or 20 micron as indicated.

Key pathways which were previously reported to be main events in AKI induction, such as the Renin-Angiotensin Aldosterone System (RAAS) and involvement of the TNFα-signalling cascade, could be confirmed in our analysis to be up-regulated. Upstream activators, such as the kallikrein and cathepsin systems, were also induced, suggesting a specific activation of the aforementioned RAAS axis. Additional up-regulated modulatory events, which were *per se* not associated with the currently perceived molecular model of AKI are the glutamatergic signalling cascades and associated calcium-flux pathways, which have a major detrimental effect on both apoptosis and necrosis. A substantial amount of information is available about glutamate-dependent pathways and signalling events in a non-renal, specifically neurological context, and it was surprising to encounter a considerable level of glutamatergic pathway elements associated with renal dysfunction. The specific involvement under physiological conditions of ionotropic as well as metabotropic glutamate receptors in kidney is currently unknown, however a dysfunction, such as over-stimulation and –activation is expected to lead to the same effects observed in other systems, e.g. uncontrollable calcium-influx and ultimately cell death. This observation is further acerbated by an apparent simultaneous induction of the calcium-flux machinery, involving the calcium-import and –export channels, such as calcium pumps (SERCA and PMCA) as well as ryanodine receptors and calcium-sensitive modulators. A potential assembly of signalling events originating from the RAAS axis and involving the most prominent glutamate-sensitive calcium-channel NMDA receptor is depicted in Figure 2B. As shown, signalling from the renin-induced angiotensin receptor leads to a cascade of known signalling and induction events involving PLC2β, PKCα, Ras, RalA, p38kinase, MSK and activation of the transcription factor SP1. The latter promotes gene activation of the NMDA receptor GRIN1 which however is also dependent on SP3 inhibition. SP3 inhibition can be induced by oxidative stress, reflected in this scheme in the stimulation of NADPH oxidase NOX, predicted to result from the activation of the up-stream kinase PKCα. Oxidative stress also stimulates the death-associated protein kinase Dapk1, which can target the NMDA receptor and leads to a permanent-open state of the channel, allowing calcification of the intracellular environment. This in turn triggers the mitochondrial- as well as nuclear-based apoptotic and necrotic molecular machinery.

To enhance these hypotheses and predictions, we validated specific key elements in this delineated pathway by immunohistochemistry, as shown in Figure 2A. We also tested whether the potentially anti-apoptotic protein Hsp27 is regulated according to the MS-results. This protein is involved in cytoskeletal remodelling, which might also be an involvement of the NMDA receptor under physiological conditions, however based on our pathway model this would imply that apoptosis is to some extent inhibited, whereas necrosis is not. All of the proteins tested, namely PLC2β, Ras, MSK, Grin1, Dapk1 and Hsp27, could be confirmed to be up-regulated, as measured by the proteomic mass-spectrometry approach, thereby further emphasizing the importance of this downstream pathway in the development of the AKI phenotype. Unsurprisingly we could not detect any indication of the involvement of the insulin pathway, either up- or down-stream of the insulin receptor, but instead observed an up-regulation of known inhibitors of insulin-signalling such as Socs3 and Ptprf.

Additionally, our analysis of the glycolysis and glycogenolysis pathways, which are modulatory targets of insulin-signalling, shows a significant up-regulation of enzymes involved in glucose and fructose release, and no indication of an equally activated down-stream metabolism from Fructose-1,6-bisphosphate to pyruvate or lactate. This suggests an accumulation of Fructose-1,6-bisphosphate, which is a known allosteric inhibitor of Adss, an enzyme in the *de novo* pathway of AMP production. This inhibition would lead to an accumulation of IMP, and in conjunction with the observed up-regulation of 5′(3′)-deoxyribonucleotidase Nt5c, to inosine and ultimately hypoxanthine and xanthine build-up. Those last two stages are a characteristic hallmark of AKI, thereby explaining the previously described observations of an accumulation of the latter two chemical compounds on a molecular level (see Table 1).

## Discussion

AKI has a major impact on survival in hospitalised patients undergoing extensive surgery [9], and a large number of studies have been undertaken to understand the molecular causes leading to and being involved in this complex disease [10,11]. Here, we employed a non-biased systematic approach using a combination of Systems Biology, proteomics and *de-novo* pathway mapping and immunohistochemical validation to elucidate major pathways and signalling cascades in order to contextualise previous findings and uncover novel potential targets for pharmacological intervention. The analysis focuses mainly on very significant changes observed in AKI covering all the known hallmarks using the folic acid-overdose animal model and can be regarded as a first attempt to describe this condition in a molecular mechanistic way.

An initial finding of this mapping effort was the apparent complexity of AKI and associated pathways, where a plethora of signalling cascades appears to be modulated simultaneously. This could be due to many kidney cell types being in an active state of inflammation signalling, apoptosis induction, stress, and other modulatory events, as also supported by the literature [12]. As many cascades can be activated in more than one way, and induction of AKI can occur via multiple stimulation or entry points [13], it appears plausible that these pathways are merging to similar down-stream targets leading to the observed deleterious events in kidney injury.

Our analysis revealed that a major pathway involved in AKI is the RAAS axis, which has been reported multiple times before [14,15], further confirming the validity of the approach. Renin activation can occur in several ways, either by kallikrein, cathepsin or other stimuli as indicated in Additional file 3: Figure S1. Many of those activating proteins were markedly up-regulated in AKI.

**Figure 3:**
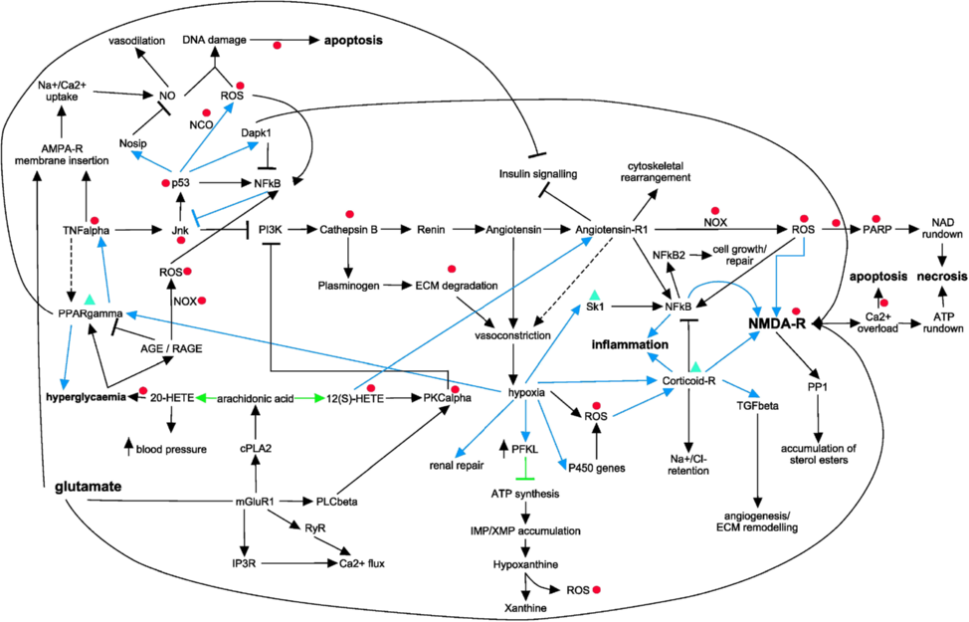
**Global molecular axes invoked during AKI.** Many interactions exist between endothelial cells, epithelial cells, and blood cells in the pathophysiology of AKI. These interactions are bidirectional between the cells involved, and result in specific functional and structural alterations. Inflammatory mediators released from proximal tubular cells influence endothelial cell processes (e.g. increased vasoconstriction and expression of cell adhesion molecules) that in turn interfere with and modulate endothelial cells, leading to reduced microvascular flow and continued hypoxia within the local environment. Co-occurring augmented ROS production within the cells and cross-signalling induces additional signalling events leading to oxidative stress. The impairment to replenish the intracellular ATP pool, as well as NAD depletion, of vascular and tubular cells is one of the major contributors to cell injury and tissue damage. Primary pathways which are involved in multifaceted AKI are delineated in gene activation cascades (blue arrows), signalling events (black arrows and inhibitions) and metabolic pathways (green arrows and inhibitions). Modulations are indicated as arrows (positive signalling events) or t-bars (inhibitions). Ultimate clinically observed end-points are marked in bold. Pharmacological intervention studies in inhibiting AKI or alleviate AKI-dependent symptoms by targeting specific molecules are indicated by inhibition of targets (red circles) or stimulation (green triangles).

A further main crucial initiation step leading towards AKI is based on up-stream activation via TNFα, where inhibition of this molecule prevents apoptotic cell death [16]. Mapping of modulated proteins found in this study clearly indicates the involvement of this cascade in AKI. TNF signals via Jnk to inhibit phosphoinositide 3-kinases (PI3Ks), which in turn leads to the release of cathepsin B-containing lysosomes and to renin activation [17], as well as activation of the pro-apoptotic protein p53 [18]. TNFα may also potentially contribute to RAAS activation as shown in Figure 3. Mounting evidence suggests that the RAAS plays a major role in kidney injury and inflammatory processes [19], whereas TNFα and TNF-like cytokines are involved in induction of cellular responses such as inflammation as well as induction and progression of apoptotic cell death [20].

Along these lines, the data obtained also suggest up-regulation of inflammatory pathways, indicated in our analysis to involve activation by the RAAS axis through the NFκB pathway, leading to downstream signalling by interleukins (see Figure 3). These results are supported by an observed role for TLRs in promoting inflammation and tissue injury in AKI, which synchronise their inflammatory signal activations via NFκB [21]. The activation of the RAAS axis also leads to the activation of a number of transcription factors [22], and our results indicate gene activation cascades involving among others NFκB, PPARγ, SMAD and HIF1α.

The significant changes in levels of e.g. collagens, Ena/VASP, Hsp27/Hspb1 and proteases such as MMP2 were interpreted by the bioinformatic tools to be correlated with cytoskeletal remodelling, extracellular matrix (ECM) degradation, and associated cell detachment, all linked to kidney injury [23]. The cytoskeletal reorganisation by Hsp27 has been shown to inhibit fibrogenesis in obstructive nephropathy [24].

The marked up-regulation of the molecular clusters associated with apoptotic pathways, including NADPH oxidases (NOX) and P450 monooxygenases, nitric oxide synthase Nos1, calpains Capn3 and 11, caspase Casp8, poly-[ADP-ribose]-polymerase Parp3, 6, and 12 and Serine-protein kinase ATM, indicates activated cell-death cascades linked to prolonged hypoxia. Vasoconstriction, one of the hallmarks of AKI, is directly responsible for hypoxia, which stimulates several proteins such as NOX and P450 monooxygenases and leads to ROS generation, including superoxide and hydrogen peroxide production by xanthine oxidase as a result of metabolic hypoxanthine accumulation [25]. This is acerbated by the notion that kidney injury can also induce the over-production of nitric oxide (NO), which, with superoxide, forms peroxynitrite, and causes cell damage via DNA fragmentation [26]. Over-production of ROS is linked to the induction of apoptosis and necrosis, and a number of pro-apoptotic and necrotic pathways have been shown to exist, some of which also require the activation of the mitochondrial calpain system through sustained Ca^2+^-influx [27]. The classical apoptotic pathway involves the release of mitochondrial proteins, leading ultimately to activation of caspases [26]. Regulated necrosis also contributes to cell loss [28] (Figure 3, Additional file 3: Figure S1 and Table 1).

A major novel finding is the modulation of proteins involved in glutamatergic signalling. This is exemplified by the up-regulation of key components of the glutamate response system, such as the metabotropic glutamate receptor Grm1 (also known as mGluR1), and ionotropic glutamate receptors Grin1 (NMDA receptor NR1), Gria2 (AMPA receptor GluR2), and specific scaffolders of these receptors, as well as known down-stream signalling molecules (e.g. CamK2b, PP2B, and Nos1) (Additional file 3: Figure S1). Activation of glutamatergic signalling components is one possible route to activate Ca^2+^-flux pathways [29]. Podocytes and tubular cells express glutamate receptors [30] including functional Grin1 NMDA receptors [31], raising the possibility that neuron-like signalling contributes to glomerular function [32]. Glutamatergic signalling driven by podocytes contributes to the integrity of the glomerular filtration barrier and may regulate glomerular filtration [33]. Additionally, one of the metabotropic glutamate receptors, Grm1, is expressed in glomerular podocytes [34]. Although the stimulus for increased NMDA receptor expression in AKI has not been characterised, hypoxia is known to induce the transcription of glutamatergic signalling components such as the NMDA receptors in non-renal cells through inhibition of the transcription factor SP3. SP3 inhibits transcription by occupying the genomic translation initiation site, and activation of SP1 which in turn occupies the freed site and promotes the transcription of target genes [35].

Collectively, based on the presented data, we could hypothesize that excessive activation or sustained activation of NMDA receptors in tubular cells or podocytes promotes oxidative stress, mobilization of cation channels, disproportionate Ca^2+^ influx and overloading, excessive ROS generation, and apoptotic cell death [36] similar to what was shown to occur in the neurological environment [37]. Indeed tissue glutamate is high in AKI [38] and pharmacologically blocking this receptor with D-AP5, a synthetic glutamate analogue which binds to the glutamate-binding site of the NMDA receptor and suppresses pore opening, prevented tissue injury [39]. Shown in Additional file 3: Figure S1 are proteins and signalling events, assembled to depict probable cascades driving both, apoptosis and necrosis in kidney tissue after injury. The glutamate receptor Grin1/NMDA-R1, which is responsible for cellular Ca^2+^-overload leading to irreversible caspase activation and apoptosis, in conjunction with sustained ROS production, and ultimately energy-depletion and necrosis, may be a major contributor to these events.

Dapk1, a gene activation target of p53, can mediate the pro-apoptotic activity of TNFα by inhibition of NFκB signalling [40]. Additionally, the NMDA receptor is a known target of Dapk1, where Dapk1-mediated channel modulation results in a permanently open NMDA receptor, leading to cell death [41]. Remarkably, we also observed an orchestrated up-regulation of relevant scaffolders (Homer1/3, Dlg1, and Dlgap4) which link the glutamate receptors directly to downstream signalling cascades ranging from Ca^2+^-signalling to phospholipase and adenylate cyclase cascades and interlinking pathways. This further supports the importance of these receptors in AKI.

Other Ca^2+^ channels, which we observed to be up-regulated in AKI, and that could potentially contribute to Ca^2+^-overload are Ca^2+^-import channels such as voltage-sensitive calcium channels VSCC (isoforms Cacna1g/h and Cacnb1) and transient receptor potential channels TRP (isoforms Trpc4, Trpm1/3), intracellular release channels sarcoplasmic/endoplasmic reticulum calcium ATPases SRCA (isoforms Atp2a1/2) which are dependent on ATP hydrolysis, and others (Slc8a3, Ryr1). Simultaneously we also detected up-regulation of Ca^2+^-efflux channels plasma membrane calcium-transporting ATPases PMCA (isoforms Atp2b1/2/3), which are dependent on available ATP to function. The depletion of intracellular ATP pools could therefore lead to an asymmetric Ca^2+^-flux and exacerbate the intracellular calcification even further.

We also extracted supportive information from the literature, where pharmacological or other intervention studies reported modulatory effects on kidney injury. The results are summarised in Additional file 4: Table S2, and also indicated in Figure 3. Inhibition of molecules downstream of TNFα and involved in ROS production and hyperglycaemia, as well as specific activation of NFκB cascades, all had an improving or attenuated outcome on AKI prevention. Of particular interest is the inhibition of TNFα signalling by using TNFα antibodies which resulted in prevention of AKI [16]. Inhibition of glutamate signalling via the NMDA receptor, by using the channel blocker D-AP5, has been shown to significantly reduce ischemia/reperfusion injury (I/RI) -induced glomerular and tubular dysfunction [39], and NMDA-induced injury, which leads to calcification of the intracellular space, was shown to be reduced by using calcium antagonists nimodipine and dantrolene in an animal model of retinal injury [42]. Nimodipine targets and blocks L-type calcium channels, whereas dantrolene inhibits ryanodine receptors. Nimodipine was shown to alleviate tubular cell necrosis in transplantation-induced AKI [43]. Dantrolene in particular gained interest as it was shown to be beneficial in protecting against I/RI in animal models of heart-, brain- and potentially liver-induced I/RI [44]. However, dantrolene was shown to be ineffective in protecting renal function [45]. The same study investigated two other calcium-channel inhibitors, namely nicardipine, which blocks L-type calcium channels such as CACNB1, and TMB-8, blocking inositol 1,4,5-trisphosphate receptors. They could demonstrate that both compounds applied in pre-conditioning prior to I/RI had a positive effect both *in vitro* on tubular cells by decreasing the apoptotic effect, and *in vivo* in a rat renal model in kidney functional integrity. The results of those pharmacological studies listed in Additional file 4: Table S2 suggest that AKI in principle can be prevented or its effect diminished, and a combination of various drugs, targeting specific AKI-induced pathways and molecules, could be a way forward in disease prevention or therapeutic intervention.

## Conclusions

**Figure 4:**
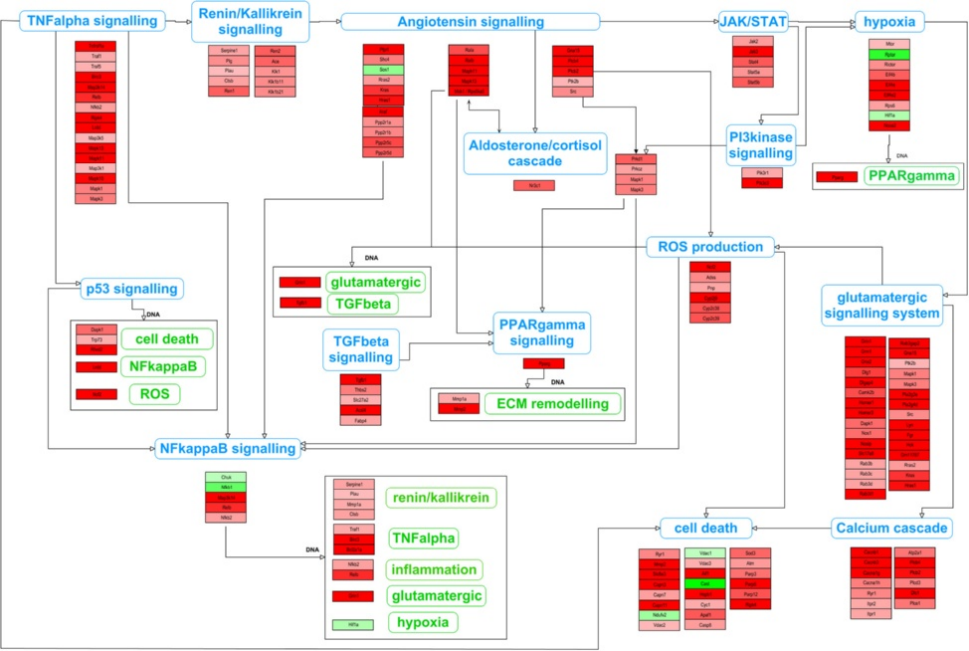
**Clustered view of pathways involved in AKI.** A summarised overview of signalling cascades triggered by AKI leading ultimately to apoptosis and necrosis is shown. Based on the bioinformatics approach the Angiotensin-Aldosterone system, ROS, NFκB, glutamatergic signalling and cell death appear to be key targets for therapeutic intervention. Of these, ROS targeting approaches have failed to date and efforts should concentrate in selective ROS targeting. In this respect recent successful attempts have focused on mitochondrial ROS targeting. There is also evidence that targeting cell death and NFκB might be beneficial, although the complexity of the NFκB system requires exploration of more selective approaches. By contrast the role of glutamatergic signalling in AKI remains largely unexplored. The initial entry-point was selected by activating the RAAS axis, though it is known that different AKI-conditions can have other starting points within and outside the sequence of events depicted. Since pathway names can be ambiguous, the proteins involved and found in the most significant dataset of 1480 entries are listed as well. The protein coloration ranges from green (down-regulated) to red (up-regulated). Pathway names in green are evoked after transcriptional and translational events.

A potential synopsis of pathways and signalling events evoked during AKI leading to Ca^2+^-overload and apoptosis as well as necrosis is shown in Figure 4, and a focus centred around the NMDA receptor is shown in Figure 2B. The relevant endogenous NMDA receptor ligand in AKI has not been characterised, however kidney glutamate levels are increased in AKI [38]. To further substantiate this cascade, we validated the up-regulation of key molecules involved in this axis as by immunohistochemistry shown in Figure 2A. The signalling cascade downstream of angiotensin-receptor 1 activates PLC2β which signals to PKCα. Both these proteins were up-regulated in AKI based on the mass-spectrometric analysis and confirmed for PLC2β by immunohistochemistry. PKCα activation leads to ROS production via NOX [46], and induces gene activation of the NMDA receptor, which we found to be up-regulated by both techniques used. This gene induction step also requires activation of MSK, which we also found to be up-regulated. Additionally, we could show that Hsp27 is up-regulated, indicative of cytoskeletal reorganisation, and we could demonstrate that Dapk1 is also up-regulated. Hsp27 up-regulation may protect from angiotensin II- and high-glucose-induced apoptosis [47]. All these events are potentially linked to both apoptosis and necrosis, and through this up-regulated pathway could be demonstrating a probable route of tubular epithelial fate in AKI.

The tissue proteomics approach identified here may eventually be optimized for its use in diagnostic/staging clinical pathology and the molecular fingerprint identified may be used to explore different molecular types of clinical AKI that may require different therapeutic approaches.

In summary we assembled a global pathway model for AKI based on proteomic changes, and further support the concept that the approach of in-depth Systems Biology coupled with high-resolution proteomic analysis represents a valuable approach to elucidate complex pathways and systems, especially in multifaceted diseases such as AKI. This combined methodology can be applied to search for druggable targets and identify key nodes where molecular/pharmacological interventions are most likely to have an impact on the system as a whole, as well as predictions for disease biomarkers. One such target could be the glutamatergic system, however the NMDA receptor itself might not be an ideal lead due to its involvement in neurotransmission in other areas like cardiac function, but a renally localised combinatorial pharmacological intervention, blocking both glutamatergic signalling and Ca^2+^-flux could be of beneficial effect.

## Methods

### Animal model

Folic acid nephropathy is a classical model of AKI [48]. C57/BL6 mice from IFFA-CREDO (Barcelona, Spain) (12- to 14-week-old) received a single i.p. injection of folic acid (Sigma) 250 mg/kg in 0.3 mol/L sodium bicarbonate (AKI animals) or vehicle alone (controls) and mice were killed 24 h later [8]. Kidneys were cold saline perfused in situ before removal. One kidney from each mouse was fixed in buffered formalin, embedded in paraffin and stained with hematoxylin-eosin or used for immunohistochemistry. The kidney cortex from other kidney was snap-frozen in liquid nitrogen for protein studies. The study was approved by the IIS-FJD animal ethics committee and followed Directive 2010/63/EU on the protection of animals used for scientific purposes.

### Sample preparation

Tissue samples were weighed out and extracted using the Filter Aided Sample Preparation (FASP) method [49]. Briefly, tissue samples were homogenised in SDS-lysis buffer (1:10 sample to buffer ratio) (0.1 M Tris–HCl pH 7.6 supplemented with 4% SDS and 0.1 M DTT) using an Ultra-Turrax T 25 (IKA, Staufen, Germany), incubated at 95°C for 3 minutes and clarified by centrifugation at 16,000 g for 5 min at room temperature. An aliquot of the supernatant was taken and placed in a Micron YM-30 filter device (Millipore, Watford UK). 8 M Urea buffer (UA) was added to the protein extract and then centrifuged at 14,000 g for 15 minutes. This step was repeated twice after which the protein extract was mixed gently for 1 minute with 0.05 M iodoacetamide buffer (IAA) and incubated for a further 20 minutes prior to centrifugation. UA buffer was again added and centrifuged (twice). Ammonium bicarbonate buffer (50 mM of NH_4_HCO_3_, pH 8) (ABC) was then added and centrifuged (twice) before incubating overnight with trypsin. The trypsin homogenate was centrifuged and washed with ABC buffer prior to acidification with 10% formic acid. Sample volumes were adjusted to match final concentration of protein prior to analysis by LC-MS/MS.

### LC-MS/MS mass spectrometry analysis

Tissue extracts were separated on a Dionex Ultimate 3000 RSLS nano flow system (Dionex, Camberly UK). A 5 μl sample was loaded in 0.1% formic acid and acetonitrile (98:2) onto a Dionex 100 μm × 2 cm, 5 μm C18 nano trap column at a flowrate of 5 μl/min. Elution was performed on an Acclaim PepMap C18 nano column 75 μm × 50 cm, 2 μm, 100 Å with a linear gradient of solvent A, 0.1% formic acid and acetonitrile (98:2) against solvent B, 0.1% formic acid and acetonitrile (20:80) starting at 1% B for 5 minutes rising to 30% at 400 minutes then to 50%B at 480 minutes. The sample was ionized in positive ion mode using a Proxeon nano spray ESI source (Thermo Fisher Hemel UK) and analyzed in an Orbitrap Velos FTMS (Thermo Finnigan, Bremen, Germany). The MS was operated in a data-dependent mode (top 40) to switch between MS and MS/MS acquisition and parent ions were fragmented by collision-induced dissociation (CID). Data files were searched against the IPI mouse non-redundant database (version 3.8.5) using SEQUEST with enzyme specified as trypsin. A fixed modification of carbamidomethylation was set and oxidation of methionine and proline as variable modifications were selected. Mass error windows of 20 ppm and 0.8 Da were allowed for MS and MS/MS, respectively. In SEQUEST, only peptides that showed mass deviation of less than 10 ppm were passed, the peptide data were extracted using high peptide confidence and top one peptide rank filters. Statistical p-value analysis was performed using the Mann Whitney test (implemented in in-house software). Normalisation of the mass spectrometry data was done using Histone H2B. Similar results were obtained when the normalisation was performed against actin (skeletal muscle isoform).

### Systems Biology analysis

Data merging was done by Blast-searching the SwissProt database with all 9930 identified molecules individually, or by batch-transfer using the UniProt online tool, to transfer all IPI accession numbers to the SwissProt identifiers, followed by combining all duplicated entries. Mass spectrometry measurement values were adjusted using the average or, in case where the protein was only found in one instance in a sample, by taking only the measured value. This dataset of 6564 entries was then used in the downstream analysis (Additional file 1: Table S1). The p-values were re-calculated using the Mann Whitney test, and the fold changes calculated by using the averages where missing values were ignored. 1480 molecules were significantly changed in AKI using high-stringency cut-off values of p-values <0.05 and fold changes >2. Of those, 1046 were only found in the AKI kidney samples, 14 only in controls, and 420 in both. Gene ontology analysis was performed using the CytoScape plug-in ClueGO. Initial pathway mapping was done using KEGG (online) as well as WikiPathway maps (using PathVisio software), where 28 partially overlapping metabolic and signalling cascades could be identified. Data for molecular properties were obtained from UniProt and the published literature, as well as other databases (PADB, BRENDA, Reactome, IntAct, GeneCards, NCBI RefSeq and Entrez). *De-novo* pathway maps were constructed by extended manual literature searches. Gene activation cascades were put together by manual literature mining and use of on-line resources such as GeneCards. AKI-specific metabolic maps were created based on information from KEGG, Reactome, BRENDA and UniProt, as well as the published literature. The delineated pathways were then combined into plausible signalling cascades and initial sub-models were manually assembled using the PathVisio software. A final model was established after several re-iterations and literature cross-checks.

### Immunohistochemistry

Immunohistochemistry was carried out in 5 μm thick paraffin-embedded tissue sections [50]. The primary antibodies were rabbit polyclonal anti-PLCβ2, goat polyclonal anti-HSP27, rat monoclonal anti-H-Ras, rabbit polyclonal anti-MSK1 (1:100, Santa Cruz biotechnology), rabbit polyclonal anti-NMDAR1 (1:100, Abcam) and rabbit polyclonal anti-DAP Kinase 1 (1:100, Sigma). Sections were counterstained with Carazzi’s hematoxylin. Negative controls included incubation with a non-specific immunoglobulin of the same isotype as the primary antibody. These sections were mounted in 90% glycerol/PBS and examined in a blinded manner.

## Abbreviations

AKI: Acute kidney injury; LC-MS/MS: Liquid chromatography mass spectrometry; NMDA: N-methyl-D-aspartate; NOX: NADPH oxidases; RAAS: Renin-Angiotensin Aldosterone System; ROS: Reactive oxygen species.

## Competing interests

HM is the owner and CEO of Mosaiques GmbH, Hannover, Germany. No other competing interests are declared.

## Authors’ contributions

HH wrote the draft of the manuscript and did the Systems Biology analysis, WM did the mass spectrometry measurements, MDSN did the animal model and the immunohistochemistry work, CD contributed to the clinical relevance of modelling and correctness, AV initiated the project, and both AO and HM led the research. All authors contributed to the writing of the manuscript. All authors read and approved the final manuscript.

## Supplementary Material

Additional file 1: Table S1 Proteomic and bioinformatic analysis of the AKI dataset. Data entries were matched to various databases by accession numbers such as SwissProt, UniProt, EnsEMBL, TrEMBL, PIR, Unigene, Entrez (GI), IPI and NCBI-GeneIndex. Raw data from the six control and six AKI samples, as measured by LC-MS, and derived values such as fold changes and p-values are included. Click here for file

Additional file 2 **This file contains in-depth methods and results used to generate the molecular pathway map.** It also contains the results of GO, interactome and KEGG pathway mapping analysis, as well as the original assembly of pathway cascades used to establish the global pathway shown in Additional file 3: Figure S1. Click here for file

Additional file 3: Figure S1 Pathway map in AKI. Molecules implicated in AKI were mapped onto pathways based on prior knowledge of signalling cascades as well as merged de-novo pathways. Fold changes of individual proteins range from green (down-regulation) to white (unchanged) to red (up-regulation). Grey denotes proteins and metabolites without fold-change information. ROS are highlighted with a green surrounding box. The legend is included in the top-left corner. Click here for file

Additional file 4: Table S2 Reported therapeutic agents employed in acute kidney injury models. Abbreviated names are indicated in round brackets, and gene names in square brackets. Click here for file
