# Supplementary figures and images for: A combinatorial approach of Proteomics and Systems Biology in unravelling the mechanisms of acute kidney injury (AKI): involvement of NMDA receptor GRIN1 in murine AKI
Source: BMC Syst Biol. 2013 Oct 30;7:110. doi: 10.1186/1752-0509-7-110 (PMC3827826; doi:10.1186/1752-0509-7-110)

species: mouse [download \(columbus\)](#)

degradation/cleavage  
of cell adhesion molecules

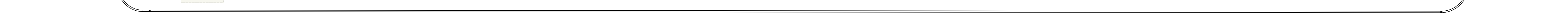

Supplement: Additional file 3: Figure S1 — Pathway map in AKI. Molecules implicated in AKI were mapped onto pathways based on prior knowledge of signalling cascades as well as merged de-novo pathways. Fold changes of individual proteins range from green (down-regulation) to white (unchanged) to red (up-regulation). Grey denotes proteins and metabolites without fold-change information. ROS are highlighted with a green surrounding box. The legend is included in the top-left corner. [file 1752-0509-7-110-S3.pdf]
