# Supplementary material for: A combinatorial approach of Proteomics and Systems Biology in unravelling the mechanisms of acute kidney injury (AKI): involvement of NMDA receptor GRIN1 in murine AKI
Source: BMC Syst Biol. 2013 Oct 30;7:110. doi: 10.1186/1752-0509-7-110 (PMC3827826; doi:10.1186/1752-0509-7-110)
Supplement: Additional file 4: Table S2 — Reported therapeutic agents employed in acute kidney injury models. Abbreviated names are indicated in round brackets, and gene names in square brackets. [file 1752-0509-7-110-S4.doc]

**Table S2. Reported therapeutic agents employed in AKI models.** Abbreviated names are indicated in round brackets, and gene names in square brackets.

| **Agent** | **Mechanism** | **Target** | **Kidney injury induction model** | **Global outcome** | **Reference** |
| --- | --- | --- | --- | --- | --- |
| 1,25-dihydroxyvitamin D(3) [1,25(OH)(2)D(3)] | Suppressing the RAAS, with renin and angiotensinogen (AGT) | NF-κB inhibition | High glucose | AGT induction in diabetic mice was suppressed by treatment with a vitamin D analogue | 1 |
| Adenosine receptor agonist (2- chlorocyclopentyladenosine) | Anti-inflammatory, antiapoptosis | A(1) adenosine receptor [ADORA1] | I/RI in rats | Protects by reducing inflammation, necrosis, and apoptosis | 2 |
| Adrenocorticotropic hormone (ACTH) | Steroidogenic-dependent and -independent mechanisms | Adrenocorticotropic hormone receptor | TNF-induced AKI | ACTH has additive renoprotective actions achieved by both steroid-dependent mechanisms and MC1R-directed anti-apoptosis | 3 |
| Alpha-1-acid glycoprotein | Anti-inflammatory, antiapoptosis | Inhibiting neutrophil infiltration | I/RI | Protects by preservation of tubular epithelial structure and inhibition of apoptosis and subsequent inflammation | 4 |
| Alpha-Melanocyte stimulating hormone, and AP214 | Anti-inflammatory, anti-apoptosis | Melanocortin receptor | I/RI, sepsis-induced AKI | Prevent I/RI such as urinary-concentrating defects and down-regulation of renal AQPs and sodium transporters. Improves hemodynamic failure, AKI, mortality and splenocyte apoptosis attenuating pro- and anti-inflammatory actions due to sepsis | ^5^, ^6^ |
| Amifostine | Anti-tubular damage | Scavenging oxygen free radicals | I/RI | Decreased the degree and severity of tubular damage | 7 |
| anti-TNF-α antibody | Anti-inflammatory, anti-apoptosis | TNF-α | FA AKI | markedly blocked the apoptotic death | 8 |
| Apocynin | ROS-production inhibition | NADPH-oxidase | I/RI | Ameliorated extensive tubular necrosis, glomerular damage, and apoptosis in the histological evaluation | 9 |
| Apotransferrin | Iron chelation | Free iron ions | I/RI | Protects against renal I/RI, inhibiting oxidative stress, inflammation, and loss of function | 10 |
| Atorvastatin | Anti- tubulointerstitial damage, anti-apoptosis, anti-necrosis | Caspase-3 inhibition | I/RI | Improves reperfusion tolerance | 11 |
| Benzyloxicarbonyl-L-phenylalanyl-alanine-fluoromethylketone (Z-FA.FMK) | Anti-degenerative changes, anti-apoptosis | Cathepsin B inhibition | D-GalN/TNF-α-induced AKI | Markedly lessens the degree of impairment seen in D-GalN/TNF-α-induced AKI | 12 |
| Beta-1-integrin antagonist | Anti-inflammatory | Beta-1-integrin antagonist | I/RI | Preventing tubular epithelial cell detachment | 13 |
| C5a receptor antagonist | Anti-inflammatory | Complement receptor | I/RI | Significantly reduced loss of renal function, no influence on renal apoptosis. Preventing C5 activation abrogates late apoptosis and inflammation, being strongly protective against renal function loss. Improves early graft function following cadaveric kidney transplantation | ^14^, ^15^, ^16^ |
| Carbon monoxide | Reduced acute tubular necrosis, anti-apoptosis | Unknown | I/RI | Blocks anoxia-reoxygenation-induced cell death while promoting proliferation | 17 |
| CO-releasing compounds | Vasodilation | Unknown | I/RI | Significant protective effects | ^18^, ^19^ |
| Cyclosporin A (CsA) | Anti-necrosis | Cyclophilin D (CypD-mediated mitochondrial pore transition) | FA-induced AKI | A single dose of CsA significantly protects mice from FA-induced AKI, presumably through inhibition of cell death, inflammatory reaction, interstitial cell infiltration and fibrosis | 20 |
| D-AP5 | Anti-Ca2+-overload by glutamate (NMDA) receptors | NMDA-glutamate receptor channel blocker | I/RI | Treatment significantly ameliorated I/RI -induced glomerular and tubular dysfunction by restoring decreased GFR, UV, and U(Na)V levels | 21 |
| Dexamethasone | Anti-apoptosis, anti-necrosis | Glucocorticoid receptor | I/RI | Protects against kidney injury, stimulates rapid and transient phosphorylation of ERK 1/2, which requires the presence of the glucocorticoid receptor and was independent of transcriptional activity | 22 |
| Dexpanthenol | Anti-tubular necrosis and glomerular damage, anti-apoptosis | Unknown | I/RI | Ameliorated extensive tubular necrosis, glomerular damage and apoptosis | 23 |
| Edaravone | ROS scavenger | ROS | I/RI | Ameliorates renal ischemia/reperfusion injury by scavenging free radicals produced in renal tubular cells and inhibiting lipid peroxidation | 24 |
| Endothelin receptor antagonist | Vasodilation | Endothelin | I/RI | Effective in prophylaxis | 25 |
| Erythropoietin | Anti-inflammatory, anti-apoptosis | Erythropoietin receptor | I/RI AKI, unilateral ureteral obstruction I/RI , LPS-induced AKI | Significantly prevent AKI. Significantly reduced the amount of cell death. Exerts renoprotective effects, not preventing the occurrence of tubular necrosis but attenuated. Renoprotective effects against the inflammatory process and cell apoptosis during endotoxemia | ^5^, ^26^, ^27^, ^28^, ^29^ |
| Etanercept | Anti-inflammatory, anti-apoptotic | TNF-α blocker (by enhancing the activation of ERK and increasing the Bcl-2/Bax) | I/RI | Etanercept improved resistance to renal injury during IR by enhancing the activation of ERK and increasing the Bcl-2/Bax ratio | 30 |
| Fidarestat | Anti-inflammatory | Aldose reductase (inhibition) | LPS-induced AKI | Ameliorated vacuolar degeneration and apoptosis of renal tubular cells as well as infiltration of neutrophils and macrophages | 31 |
| Geranylgeranylacetone | Induction of cytoprotective HSP | Induces Hsp70 | I/RI | Protects tubular epithelial cells from apoptosis | 32 |
| Hepatocyte growth factor [HGF] | Growth factor, anti-apoptosis | Hepatocyte growth factor receptor [MET] | I/RI, glycerol-induced AKI | HGF overexpression results in dramatic protection from ischemia-induced AKI. Endogenous HGF attenuates the renal inflammatory response, leukocyte infiltration and Th1 polarization after glycerol injection | ^33^, ^34^ |
| Hesperidin/Hesperadin | Decreasing oxidative stress, inflammation and DNA damage, reduced cholesterol and blood pressure | Aurora kinase B | cisplatin-induced AKI | Co-administration may prevent cisplatin-induced AKI | 35 |
| Hydrogen sulphide (H_2_S) | Anti-inflammatory, anti-apoptosis | Unknown, but naturally produced by cystathionine gamma-lyase [CSE] | I/RI | Synthesis of endogenous H_2_S by CSE is essential to protect the kidney against I/RI and dysfunction and aids in the recovery of renal function following I/RI, H_2_S generated by sodium hydrosulfide reduces I/RI and dysfunction, and morphological changes of the kidney, and the observed protective effects of H_2_S are due to both anti-apoptotic and anti-inflammatory effects. Supplemental H_2_S can mitigate renal graft I/RI incurred during transplantation and prolonged cold storage, improving early graft function and recipient survival | ^36^, ^37^ |
| Hyperbaric oxygen | Anti-apoptosis, anti-necrosis | Hypoxia mechanism | Myoglobinuric AKI | Inhibited apoptosis, did not increase regeneration | 38 |
| IL-10 | Anti-inflammatory | IL-10 receptor | Cisplatin, I/RI and rat renal transplantation | Protects against I/RI and cisplatin-induced AKI | 39 |
| IL-11 | Anti-necrosis, anti-inflammatory, anti-apoptosis | Sphingosine kinase-1 [SK1] induction via HIF-1α | I/RI | Powerful renal protective effects by reducing necrosis, inflammation, and apoptosis through induction of SK1 via HIF-1α | 40 |
| IL-6 antagonist | Anti-inflammatory | IL-6 | I/RI | Endogenous IL-6 enhances the degree of renal injury, dysfunction, and inflammation caused by I/R by promoting the expression of adhesion molecules and subsequent oxidative and nitrosative stress | 41 |
| Indomethacin | Anti-fibrosis | Unknown | I/RI | Beneficial effect on proximal tubule cell survival | 42 |
| Inhibitors of matrix metalloproteinases (minocycline, synthetic peptide MMP inhibitor) | Anti-acute tubular injury, anti-apoptosis, anti-necrosis | MMP-2, MMP-9 | I/RI | Protect against I/RI AKI, improved renal dysfunction | 43 |
| Isoflurane | Anti-apoptosis | Sphingosine kinase-1 [SK1] induction via Erk1/2 | I/RI | Protects against endothelial apoptosis most likely via SK1 and ERK MAPK activation | 44 |
| Leflunomide | Anti-inflammatory | Aryl hydrocarbon receptor | I/RI | Increased mobilization of stems cells subsets (i.e., mesenchymal and hematopoietic stem cells and endothelial progenitor cells) in the peripheral blood and promoted their recruitment into the I/RI kidney | 45 |
| Ligustrazine | Reduction of oxidative stress, anti-apoptosis | Unknown | I/RI | Protects murine kidney from warm I/RI, probably via reducing oxidative stress, inhibiting cell apoptosis, decreasing neutrophils infiltration, and suppressing the overexpression of TNF-α and ICAM-1 levels | 46 |
| low-molecular-weight fucoidan (LMWF) | Anti-apoptosis | Unknown | I/RI | Ameliorates acute renal IRI via inhibiting MAPK signaling pathways | 47 |
| Melatonin | Anti-apoptosis, anti-necrosis | Endothelial progenitor cells, inhibition of NF-κB | I/RI | Protected kidney donor grafts from IRI-induced renal dysfunction and tubular injury most likely through its anti-oxidative, anti-apoptotic and NF-κB inhibitory capacity. Causes "early outgrowth" endothelial progenitor cells | ^48^, ^49^ |
| Mesenchymal stem cells | Anti-inflammatory, anti-apoptosis (partial) | Paracrine effects | I/RI AKI, gentamicin induced AKI | Highly significant renoprotection. The subcapsular transplantation of MMCs ameliorated renal function and repair kidney injury. Minimizes renal damage | ^50^, ^51^, ^52^ |
| Milrinone | Vasodilation, anti-inflammatory | Phosphodiesterase [PDE] 3 inhibitor | I/RI | Maintained renal tissue blood flow by its vasodilatory effect, suppressed expression of TNF-α mRNA by increasing intracellular cyclic adenosine monophosphate, and ultimately decreased tubular cell apoptosis | 53 |
| MnTMPyP | Reduces oxidative stress, anti-apoptosis | SOD mimetic | I/RI | Effective in reducing apoptosis | 54 |
| Neutrophil gelatinase-associated lipocalin (NGAL) | Iron chelation, growth factor, anti-apoptosis | Organic cation transporter [SLC22A17] | I/RI | Reduced apoptotic tubule cells and increased proliferating proximal tubule cells | ^55^, ^56^ |
| Nicardipine | Anti-apoptosis | L-type calcium channel [CACNB1] | I/RI | Inhibition of cytochrome C release and caspase 3 activation, decrease of apoptotic cell number and in vivo protects renal function | 57 |
| Nimodipine | Anti-necrotic | L-type calcium channel [CACNB1] | I/RI, CsA-induced nephrotoxicity | Useful in renal transplantation for protection against cyclosporin toxicity and post-transplant acute tubular necrosis | 58 |
| PARP inhibitor 5-aminoisoquinolinone (5-AIQ) and PJ-34 | Anti-apoptosis | Poly(adenosine diphosphate-ribose) polymerase [PARP] | I/RI | Significantly reduce cellular injury and death caused to primary cultures of rat proximal tubular cells by oxidative stress in vitro, and renal injury and dysfunction. Decreased systemic levels of TNFα and IL6, attenuated apoptosis | ^59^, ^60^ |
| PD-81723 | Anti-necrosis, anti-apoptosis, Anti-inflammatory | A1 adenosine receptor (allosteric enhancer) | I/RI | Reduces necrosis, inflammation, and apoptosis through the induction of renal tubular SK1 and activation of proximal tubule S1P(1)Rs | 61 |
| Pentoxifylline | Anti-inflammatory and anti-oxidant, anti-drug-induced nephrotoxicity | Downregulating TNF-α | I/RI | Attenuated renal tissue injury and number of apoptotic cells. Protective effects against renal toxicity of some antimicrobial and cytotoxic agents | ^62^, ^63^ |
| Pituitary adenylate cyclase-activating polypeptide (PACAP)38 | Anti-inflammatory, anti-tubular injury, reduced oxidative injury | Adenylate cyclase in CsA induced nephrotoxicity | I/RI, CsA-induced nephrotoxicity | Renoprotective effect of PACAP in AKI involves both MyD88-dependent and -independent pathways. Ameliorated renal tubular injury, reduced oxidative injury, and inhibited the expression of TGF-β1 in CsA-exposed murine kidneys | ^64^, ^65^ |
| PPAR-β/δ agonist L-165041 | Anti-inflammatory, anti-apoptosis | PPAR-β/δ | I/RI | Target for preconditioning strategies | 66 |
| Propofol | Anti-LPS-induced kidney injury, anti-oxidative stress | Bone morphogenetic protein-7 induction | AKI during sepsis, rat LPS-induced AKI | Protect kidney from sepsis-induced AKI by increasing BMP-7 expression, decreasing inflammatory cytokines and inhibiting oxidative stress. Pretreatment protected renal function in a rat model of endotoxaemia | ^67^, ^68^ |
| Quercetin, Silymarin, Luteolin, Cilastatin, Rosiglitazone | Anti- oxidative stress, anti-inflammation, anti- apoptosis, tubular protection | Renoprotective agent in cisplatin-induced nephrotoxicity, through down-regulation of p53-dependent apoptotic pathway, dehydrodipeptidase I inhibition | Cisplatin nephrotoxicity | Pre-treatment with rosiglitazone attenuates cisplatin-induced renal damage through the suppression of TNF-α overproduction and NF-κB activation. Cilastatin attenuates cisplatin-induced cell death in proximal tubular cells without reducing the cytotoxic activity of cisplatin in tumor cells. Luteolin ameliorates the cisplatin-mediated nephrotoxicity through down-regulation of p53-dependent apoptotic pathway. Silymarin protects renal cells from cisplatin-induced cell death. Co-treatment with quercetin partially prevented all the renal effects of cisplatin | ^69^, ^70^, ^71^, ^72^, ^73^ |
| Rapamycin | Anti-apoptosis, anti-necrosis | Immunosuppression, FKBP12 inhibition, ryanodine receptor activation, mTOR pathway | I/RI | Preconditioning donor animals in a rat syngenic kidney transplantation model improves clinical outcomes and reduce necrosis and apoptosis by decreasing acute tubular necrosis significantly | 74 |
| Renalase | Anti-inflammatory, reduction in blood pressure, vasodilation | Catecholamines, e.g. dopamine (degradation) | I/RI | Protects against I/RI AKI by reducing renal tubular necrosis, apoptosis, and inflammation | 75 |
| Rolipram | Decrease oxidative renal tissue damage | Phosphodiesterase-4 | I/RI | Decreases oxidative renal tissue damage | 76 |
| S1P(1)R agonist (sphingolipid sphingosine-1-phosphate (S1P)) | Anti-inflammatory, anti-apoptosis | Sphingosine 1-phosphate receptor 1 [S1P(1)R] | I/RI | Pretreatment with S1P resulted in an attenuation of systemic inflammation and kidney injury. SK1 is renoprotective by S1P(1) activation and perhaps HSP27 induction | ^77^, ^78^ |
| S1P(2)R antagonist JTE013 | Anti-inflammatory, anti-apoptosis | Sphingosine 1-phosphate receptor 2 [S1P(2)R] | I/RI | S1P(2)R antagonist selectively upregulated SK1 and attenuated both H_2_O_2_-induced necrosis and TNF-α/cycloheximide-induced apoptosis | 79 |
| Sotrastaurin | Enhanced immediate transplant function, attenuated epithelial injury, and accelerated renal function recovery | PKC inhibition | Pre-transplantation treatment of recipients of kidneys in rat | The PKC inhibitor sotrastaurin effectively ameliorated ischemia-reperfusion organ damage and promoted cytoprotection in a clinically relevant model of extended renal cold preservation followed by transplantation. | 80 |
| SP600125 | Anti-apoptosis | JNK [Mapk8/9/10] inhibition | I/RI | Inhibits the activation of the JNK-c-Jun-FasL pathway and protect renal tubular epithelial cells against apoptosis | 81 |
| Splenectomy | Anti-inflammatory, anti-apoptosis | Removal of spleen | I/RI | Splenectomy reduces renal I/RI. This effect may occur by an anti-inflammatory pathway and inhibition of cell apoptosis | 82 |
| Statins (Cerivastatin, Atorvastatin) | Anti-inflammatory | Inflammatory mechanisms (Hydroxy-3-methylglutaryl coenzyme A reductase inhibitors) | I/RI AKI | Protects renal tissue, involves anti-inflammatory effects, with inhibition of mitogen-activated protein kinase activation and the redox-sensitive transcription factors NF-κB and activator protein-1 (AP-1). Induction of protective molecules such as IL-6 may underlie this effect. Low-dose treatment with atorvastatin enhances NO availability in aging rats, improving renal dynamics and conferring a peculiar histologic protection at tubular level after ischemia | ^83^, ^84^, ^85^ |
| Tacrolimus | Anti-apoptosis, anti-necrosis | Immunosuppression, FKBP12 inhibition, ryanodine receptor activation | I/RI | Preconditioning donor animals in a rat syngenic kidney transplantation model improves clinical outcomes and reduce necrosis and apoptosis by decreasing acute tubular necrosis significantly | 74 |
| Tauroursodeoxycholic acid (TUCDA) | Anti-endoplasmic reticulum stress, anti-apoptosis | blocking GRP78 and CHOP expression, reducing Caspase 12 activation | I/RI | Nephroprotective effect on I/RI-induced AKI by inhibiting endoplasmic reticulum stress and by blocking GRP78 and CHOP expression, reducing Caspase 12 activation and inhibiting cell apoptosis | 86 |
| TDZD-8 | Anti-tubular necrosis, anti-apoptosis | Glycogen synthase kinase-3β | LPS-induced AKI, nonsteroidal anti-inflammatory drugs (NSAIDs) induced AKI | GSK-3 inhibition protects against endotoxaemic acute renal failure mainly by down-regulating pro-inflammatory TNF-α and RANTES. Ameliorates NSAID-induced acute kidney injury by induction of renal cortical COX-2 and direct inhibition of the mitochondrial permeability transition | ^87^, ^88^ |
| Telmisartan | Antioxidant, anti-inflammatory, anti-apoptotic | angiotensin II receptor antagonist | I/RI | Pre-treatment markedly ameliorated I/RI -induced renal tissue damage | 89 |
| Tetramethylpyrazine | Anti-oxidation, anti-inflammation | Heme oxygenase-1 | Gentamicin-induced AKI | Protect against gentamicin-induced nephrotoxicity through antiapoptotic and anti-inflammatory mechanisms | 90 |
| TMB-8 | Anti-apoptosis | Inositol 1,4,5-trisphosphate receptor (InsP3R) | I/RI | Inhibition of cytochrome C release and caspase 3 activation, decrease of apoptotic cell number and in vivo protects renal function | 57 |

I/RI: ischemia/reperfusion injury; RAAS: Renin-Angiotensin-Aldostrone system; LPS: Lipopolysaccharide, FA: folic acid.

Reference List

1. Deb,D.K. *et al.* 1,25-Dihydroxyvitamin D3 suppresses high glucose-induced angiotensinogen expression in kidney cells by blocking the NF-{kappa}B pathway. *Am. J. Physiol Renal Physiol* **296**, F1212-F1218 (2009).

2. Lee,H.T., Gallos,G., Nasr,S.H., & Emala,C.W. A1 adenosine receptor activation inhibits inflammation, necrosis, and apoptosis after renal ischemia-reperfusion injury in mice. *J. Am. Soc. Nephrol.* **15**, 102-111 (2004).

3. Si,J. *et al.* Adrenocorticotropic hormone ameliorates acute kidney injury by steroidogenic-dependent and -independent mechanisms. *Kidney Int.* **83**, 635-646 (2013).

4. de,V.B. *et al.* Exogenous alpha-1-acid glycoprotein protects against renal ischemia-reperfusion injury by inhibition of inflammation and apoptosis. *Transplantation* **78**, 1116-1124 (2004).

5. Gong,H. *et al.* EPO and alpha-MSH prevent ischemia/reperfusion-induced down-regulation of AQPs and sodium transporters in rat kidney. *Kidney Int.* **66**, 683-695 (2004).

6. Doi,K. *et al.* AP214, an analogue of alpha-melanocyte-stimulating hormone, ameliorates sepsis-induced acute kidney injury and mortality. *Kidney Int.* **73**, 1266-1274 (2008).

7. Chok,M.K. *et al.* Renoprotective potency of amifostine in rat renal ischaemia-reperfusion. *Nephrol. Dial. Transplant.* **25**, 3845-3851 (2010).

8. Wan,B. *et al.* Blocking tumor necrosis factor-alpha inhibits folic acid-induced acute renal failure. *Exp. Mol. Pathol.* **81**, 211-216 (2006).

9. Altintas,R. *et al.* The Protective Effects of Apocynin on Kidney Damage Caused by Renal Ischemia/Reperfusion. *J. Endourol.*(2013).

10. de,V.B. *et al.* Reduction of circulating redox-active iron by apotransferrin protects against renal ischemia-reperfusion injury. *Transplantation* **77**, 669-675 (2004).

11. Haylor,J.L. *et al.* Atorvastatin improving renal ischemia reperfusion injury via direct inhibition of active caspase-3 in rats. *Exp. Biol. Med. (Maywood. )* **236**, 755-763 (2011).

12. Gezginci-Oktayoglu,S., Tunali,S., Yanardag,R., & Bolkent,S. Effects of Z-FA.FMK on D-galactosamine/tumor necrosis factor-alpha-induced kidney injury and oxidative stress in mice : effects of Z-FA.FMK on TNF-alpha-mediated kidney injury. *Mol. Cell Biochem.* **309**, 9-20 (2008).

13. Molina,A. *et al.* Renal ischemia/reperfusion injury: functional tissue preservation by anti-activated {beta}1 integrin therapy. *J. Am. Soc. Nephrol.* **16**, 374-382 (2005).

14. de,V.B. *et al.* Complement factor C5a mediates renal ischemia-reperfusion injury independent from neutrophils. *J. Immunol.* **170**, 3883-3889 (2003).

15. de,V.B. *et al.* Inhibition of complement factor C5 protects against renal ischemia-reperfusion injury: inhibition of late apoptosis and inflammation. *Transplantation* **75**, 375-382 (2003).

16. Lewis,A.G., Kohl,G., Ma,Q., Devarajan,P., & Kohl,J. Pharmacological targeting of C5a receptors during organ preservation improves kidney graft survival. *Clin. Exp. Immunol.* **153**, 117-126 (2008).

17. Hanto,D.W. *et al.* Intraoperative administration of inhaled carbon monoxide reduces delayed graft function in kidney allografts in Swine. *Am. J. Transplant.* **10**, 2421-2430 (2010).

18. Neto,J.S. *et al.* Protection of transplant-induced renal ischemia-reperfusion injury with carbon monoxide. *Am. J. Physiol Renal Physiol* **287**, F979-F989 (2004).

19. Vera,T., Henegar,J.R., Drummond,H.A., Rimoldi,J.M., & Stec,D.E. Protective effect of carbon monoxide-releasing compounds in ischemia-induced acute renal failure. *J. Am. Soc. Nephrol.* **16**, 950-958 (2005).

20. Wen,X. *et al.* One dose of cyclosporine A is protective at initiation of folic acid-induced acute kidney injury in mice. *Nephrol. Dial. Transplant.* **27**, 3100-3109 (2012).

21. Yang,C.C., Chien,C.T., Wu,M.H., Ma,M.C., & Chen,C.F. NMDA receptor blocker ameliorates ischemia-reperfusion-induced renal dysfunction in rat kidneys. *Am. J. Physiol Renal Physiol* **294**, F1433-F1440 (2008).

22. Kumar,S. *et al.* Dexamethasone ameliorates renal ischemia-reperfusion injury. *J. Am. Soc. Nephrol.* **20**, 2412-2425 (2009).

23. Altintas,R. *et al.* Protective effect of dexpanthenol on ischemia-reperfusion-induced renal injury in rats. *Kidney Blood Press Res.* **36**, 220-230 (2012).

24. Doi,K., Suzuki,Y., Nakao,A., Fujita,T., & Noiri,E. Radical scavenger edaravone developed for clinical use ameliorates ischemia/reperfusion injury in rat kidney. *Kidney Int.* **65**, 1714-1723 (2004).

25. Jerkic,M. *et al.* Relative roles of endothelin-1 and angiotensin II in experimental post-ischaemic acute renal failure. *Nephrol. Dial. Transplant.* **19**, 83-94 (2004).

26. Forman,C.J., Johnson,D.W., & Nicol,D.L. Erythropoietin administration protects against functional impairment and cell death after ischaemic renal injury in pigs. *BJU. Int.* **99**, 162-165 (2007).

27. Chang,Y.K. *et al.* Erythropoietin attenuates renal injury in an experimental model of rat unilateral ureteral obstruction via anti-inflammatory and anti-apoptotic effects. *J. Urol.* **181**, 1434-1443 (2009).

28. Caetano,A.M. *et al.* Erythropoietin attenuates apoptosis after ischemia-reperfusion-induced renal injury in transiently hyperglycemic Wister rats. *Transplant. Proc.* **43**, 3618-3621 (2011).

29. Eren,Z., Coban,J., Ekinci,I.D., Kaspar,C., & Kantarci,G. Evaluation of the effects of a high dose of erythropoietin-beta on early endotoxemia using a rat model. *Adv. Clin. Exp. Med.* **21**, 321-329 (2012).

30. Choi,D.E. *et al.* Pretreatment with the tumor nerosis factor-alpha blocker etanercept attenuated ischemia-reperfusion renal injury. *Transplant. Proc.* **41**, 3590-3596 (2009).

31. Takahashi,K. *et al.* Amelioration of acute kidney injury in lipopolysaccharide-induced systemic inflammatory response syndrome by an aldose reductase inhibitor, fidarestat. *PLoS. One.* **7**, e30134 (2012).

32. Suzuki,S. *et al.* Geranylgeranylacetone ameliorates ischemic acute renal failure via induction of Hsp70. *Kidney Int.* **67**, 2210-2220 (2005).

33. Fiaschi-Taesch,N.M. *et al.* Prevention of acute ischemic renal failure by targeted delivery of growth factors to the proximal tubule in transgenic mice: the efficacy of parathyroid hormone-related protein and hepatocyte growth factor. *J. Am. Soc. Nephrol.* **15**, 112-125 (2004).

34. Homsi,E., Janino,P., Amano,M., & Saraiva Camara,N.O. Endogenous hepatocyte growth factor attenuates inflammatory response in glycerol-induced acute kidney injury. *Am. J. Nephrol.* **29**, 283-291 (2009).

35. Sahu,B.D., Kuncha,M., Sindhura,G.J., & Sistla,R. Hesperidin attenuates cisplatin-induced acute renal injury by decreasing oxidative stress, inflammation and DNA damage. *Phytomedicine.* **20**, 453-460 (2013).

36. Tripatara,P. *et al.* Generation of endogenous hydrogen sulfide by cystathionine gamma-lyase limits renal ischemia/reperfusion injury and dysfunction. *Lab Invest* **88**, 1038-1048 (2008).

37. Lobb,I. *et al.* Supplemental hydrogen sulphide protects transplant kidney function and prolongs recipient survival after prolonged cold ischaemia-reperfusion injury by mitigating renal graft apoptosis and inflammation. *BJU. Int.* **110**, E1187-E1195 (2012).

38. Ayvaz,S. *et al.* Preventive effects of hyperbaric oxygen treatment on glycerol-induced myoglobinuric acute renal failure in rats. *J. Mol. Histol.* **43**, 161-170 (2012).

39. Deng,J. *et al.* Interleukin-10 inhibits ischemic and cisplatin-induced acute renal injury. *Kidney Int.* **60**, 2118-2128 (2001).

40. Lee,H.T. *et al.* Interleukin-11 protects against renal ischemia and reperfusion injury. *Am. J. Physiol Renal Physiol* **303**, F1216-F1224 (2012).

41. Patel,N.S. *et al.* Endogenous interleukin-6 enhances the renal injury, dysfunction, and inflammation caused by ischemia/reperfusion. *J. Pharmacol. Exp. Ther.* **312**, 1170-1178 (2005).

42. Sauvant,C. *et al.* Indomethacin corrects alterations associated with ischemia/reperfusion in an in vitro proximal tubular model. *Am. J. Nephrol.* **32**, 57-65 (2010).

43. Kunugi,S. *et al.* Inhibition of matrix metalloproteinases reduces ischemia-reperfusion acute kidney injury. *Lab Invest* **91**, 170-180 (2011).

44. Bakar,A.M., Park,S.W., Kim,M., & Lee,H.T. Isoflurane Protects Against Human Endothelial Cell Apoptosis by Inducing Sphingosine Kinase-1 via ERK MAPK. *Int. J. Mol. Sci.* **13**, 977-993 (2012).

45. Baban,B., Liu,J.Y., & Mozaffari,M.S. Aryl hydrocarbon receptor agonist, leflunomide, protects the ischemic-reperfused kidney: role of Tregs and stem cells. *Am. J. Physiol Regul. Integr. Comp Physiol* **303**, R1136-R1146 (2012).

46. Feng,L. *et al.* The protective mechanism of ligustrazine against renal ischemia/reperfusion injury. *J. Surg. Res.* **166**, 298-305 (2011).

47. Chen,J. *et al.* Low molecular weight fucoidan against renal ischemia-reperfusion injury via inhibition of the MAPK signaling pathway. *PLoS. One.* **8**, e56224 (2013).

48. Li,Z. *et al.* Melatonin protects kidney grafts from ischemia/reperfusion injury through inhibition of NF-kB and apoptosis after experimental kidney transplantation. *J. Pineal Res.* **46**, 365-372 (2009).

49. Patschan,D. *et al.* The hormone melatonin stimulates renoprotective effects of "early outgrowth" endothelial progenitor cells in acute ischemic kidney injury. *Am. J. Physiol Renal Physiol* **302**, F1305-F1312 (2012).

50. Togel,F. *et al.* Administered mesenchymal stem cells protect against ischemic acute renal failure through differentiation-independent mechanisms. *Am. J. Physiol Renal Physiol* **289**, F31-F42 (2005).

51. Liu,L. *et al.* Nephroprotective effects of subcapsular transplantation of metanephric mesenchymal cells on gentamicin-induced acute tubular necrosis in rats. *World J. Pediatr.* **8**, 156-163 (2012).

52. Reis,L.A. *et al.* Bone marrow-derived mesenchymal stem cells repaired but did not prevent gentamicin-induced acute kidney injury through paracrine effects in rats. *PLoS. One.* **7**, e44092 (2012).

53. Nishiki,T. *et al.* Effect of milrinone on ischemia-reperfusion injury in the rat kidney. *Transplant. Proc.* **43**, 1489-1494 (2011).

54. Liang,H.L. *et al.* MnTMPyP, a cell-permeant SOD mimetic, reduces oxidative stress and apoptosis following renal ischemia-reperfusion. *Am. J. Physiol Renal Physiol* **296**, F266-F276 (2009).

55. Mishra,J. *et al.* Amelioration of ischemic acute renal injury by neutrophil gelatinase-associated lipocalin. *J. Am. Soc. Nephrol.* **15**, 3073-3082 (2004).

56. Mori,K. *et al.* Endocytic delivery of lipocalin-siderophore-iron complex rescues the kidney from ischemia-reperfusion injury. *J. Clin. Invest* **115**, 610-621 (2005).

57. Wu,D. *et al.* Ischemia/reperfusion induce renal tubule apoptosis by inositol 1,4,5-trisphosphate receptor and L-type Ca2+ channel opening. *Am. J. Nephrol.* **28**, 487-499 (2008).

58. Fisher,M. & Grotta,J. New uses for calcium channel blockers. Therapeutic implications. *Drugs* **46**, 961-975 (1993).

59. Chatterjee,P.K. *et al.* 5-Aminoisoquinolinone reduces renal injury and dysfunction caused by experimental ischemia/reperfusion. *Kidney Int.* **65**, 499-509 (2004).

60. Vaschetto,R. *et al.* Inhibition of poly(adenosine diphosphate-ribose) polymerase attenuates ventilator-induced lung injury. *Anesthesiology* **108**, 261-268 (2008).

61. Park,S.W. *et al.* A1 adenosine receptor allosteric enhancer PD-81723 protects against renal ischemia-reperfusion injury. *Am. J. Physiol Renal Physiol* **303**, F721-F732 (2012).

62. Asvadi,I. *et al.* Protective effect of pentoxyfilline in renal toxicity after methotrexate administration. *Eur. Rev. Med. Pharmacol. Sci.* **15**, 1003-1009 (2011).

63. Nasiri-Toosi,Z., Dashti-Khavidaki,S., Khalili,H., & Lessan-Pezeshki,M. A review of the potential protective effects of pentoxifylline against drug-induced nephrotoxicity. *Eur. J. Clin. Pharmacol.*(2012).

64. Li,M., Khan,A.M., Maderdrut,J.L., Simon,E.E., & Batuman,V. The effect of PACAP38 on MyD88-mediated signal transduction in ischemia-/hypoxia-induced acute kidney injury. *Am. J. Nephrol.* **32**, 522-532 (2010).

65. Khan,A.M. *et al.* Renoprotection with pituitary adenylate cyclase-activating polypeptide in cyclosporine A-induced nephrotoxicity. *J. Investig. Med.* **59**, 793-802 (2011).

66. Letavernier,E. *et al.* Peroxisome proliferator-activated receptor beta/delta exerts a strong protection from ischemic acute renal failure. *J. Am. Soc. Nephrol.* **16**, 2395-2402 (2005).

67. Hsing,C.H., Chou,W., Wang,J.J., Chen,H.W., & Yeh,C.H. Propofol increases bone morphogenetic protein-7 and decreases oxidative stress in sepsis-induced acute kidney injury. *Nephrol. Dial. Transplant.* **26**, 1162-1172 (2011).

68. Cui,W.Y., Tian,A.Y., & Bai,T. Protective effects of propofol on endotoxemia-induced acute kidney injury in rats. *Clin. Exp. Pharmacol. Physiol* **38**, 747-754 (2011).

69. Lee,S. *et al.* Rosiglitazone ameliorates cisplatin-induced renal injury in mice. *Nephrol. Dial. Transplant.* **21**, 2096-2105 (2006).

70. Camano,S. *et al.* Cilastatin attenuates cisplatin-induced proximal tubular cell damage. *J. Pharmacol. Exp. Ther.* **334**, 419-429 (2010).

71. Kang,K.P. *et al.* Luteolin ameliorates cisplatin-induced acute kidney injury in mice by regulation of p53-dependent renal tubular apoptosis. *Nephrol. Dial. Transplant.* **26**, 814-822 (2011).

72. Ninsontia,C., Pongjit,K., Chaotham,C., & Chanvorachote,P. Silymarin selectively protects human renal cells from cisplatin-induced cell death. *Pharm. Biol.* **49**, 1082-1090 (2011).

73. Sanchez-Gonzalez,P.D., Lopez-Hernandez,F.J., Perez-Barriocanal,F., Morales,A.I., & Lopez-Novoa,J.M. Quercetin reduces cisplatin nephrotoxicity in rats without compromising its anti-tumour activity. *Nephrol. Dial. Transplant.* **26**, 3484-3495 (2011).

74. Cicora,F. *et al.* Preconditioning donor with a combination of tacrolimus and rapamacyn to decrease ischaemia-reperfusion injury in a rat syngenic kidney transplantation model. *Clin. Exp. Immunol.* **167**, 169-177 (2012).

75. Lee,H.T. *et al.* Renalase protects against ischemic AKI. *J. Am. Soc. Nephrol.* **24**, 445-455 (2013).

76. Mammadov,E. *et al.* Protective effects of phosphodiesterase-4-specific inhibitor rolipram on acute ischemia-reperfusion injury in rat kidney. *Urology* **80**, 1390-1396 (2012).

77. Lee,S.Y. *et al.* Sphingosine-1-phosphate reduces hepatic ischaemia/reperfusion-induced acute kidney injury through attenuation of endothelial injury in mice. *Nephrology. (Carlton. )* **16**, 163-173 (2011).

78. Park,S.W., Kim,M., Kim,M., D'Agati,V.D., & Lee,H.T. Sphingosine kinase 1 protects against renal ischemia-reperfusion injury in mice by sphingosine-1-phosphate1 receptor activation. *Kidney Int.* **80**, 1315-1327 (2011).

79. Park,S.W., Kim,M., Brown,K.M., D'Agati,V.D., & Lee,H.T. Inhibition of sphingosine 1-phosphate receptor 2 protects against renal ischemia-reperfusion injury. *J. Am. Soc. Nephrol.* **23**, 266-280 (2012).

80. Fuller,T.F. *et al.* Protein kinase C inhibition ameliorates posttransplantation preservation injury in rat renal transplants. *Transplantation* **94**, 679-686 (2012).

81. Wang,Y., Ji,H.X., Xing,S.H., Pei,D.S., & Guan,Q.H. SP600125, a selective JNK inhibitor, protects ischemic renal injury via suppressing the extrinsic pathways of apoptosis. *Life Sci.* **80**, 2067-2075 (2007).

82. Hiroyoshi,T. *et al.* Splenectomy protects the kidneys against ischemic reperfusion injury in the rat. *Transpl. Immunol.* **27**, 8-11 (2012).

83. Gueler,F. *et al.* Postischemic acute renal failure is reduced by short-term statin treatment in a rat model. *J. Am. Soc. Nephrol.* **13**, 2288-2298 (2002).

84. Yokota,N. *et al.* Protective effect of HMG-CoA reductase inhibitor on experimental renal ischemia-reperfusion injury. *Am. J. Nephrol.* **23**, 13-17 (2003).

85. Sabbatini,M. *et al.* Atorvastatin improves the course of ischemic acute renal failure in aging rats. *J. Am. Soc. Nephrol.* **15**, 901-909 (2004).

86. Gao,X. *et al.* The nephroprotective effect of tauroursodeoxycholic acid on ischaemia/reperfusion-induced acute kidney injury by inhibiting endoplasmic reticulum stress. *Basic Clin. Pharmacol. Toxicol.* **111**, 14-23 (2012).

87. Wang,Y. *et al.* Inhibiting glycogen synthase kinase-3 reduces endotoxaemic acute renal failure by down-regulating inflammation and renal cell apoptosis. *Br. J. Pharmacol.* **157**, 1004-1013 (2009).

88. Bao,H. *et al.* Inhibition of glycogen synthase kinase-3beta prevents NSAID-induced acute kidney injury. *Kidney Int.* **81**, 662-673 (2012).

89. Fouad,A.A., Qureshi,H.A., Al-Sultan,A.I., Yacoubi,M.T., & Al-Melhim,W.N. Nephroprotective effect of telmisartan in rats with ischemia/reperfusion renal injury. *Pharmacology* **85**, 158-167 (2010).

90. Sue,Y.M. *et al.* Antioxidation and anti-inflammation by haem oxygenase-1 contribute to protection by tetramethylpyrazine against gentamicin-induced apoptosis in murine renal tubular cells. *Nephrol. Dial. Transplant.* **24**, 769-777 (2009).
