# Supplementary material for: A combinatorial approach of Proteomics and Systems Biology in unravelling the mechanisms of acute kidney injury (AKI): involvement of NMDA receptor GRIN1 in murine AKI
Source: BMC Syst Biol. 2013 Oct 30;7:110. doi: 10.1186/1752-0509-7-110 (PMC3827826; doi:10.1186/1752-0509-7-110)

Supplementary Methods and Extended Analysis

Initial application of significance levels

The initial mass-spectrometric data the 12 datasets (6 control, 6 AKI samples) was assigned to 9930 molecules and was subsequently combined after normalisation, using Histone H2B as the reference molecule, to generate a non-redundant protein-list of 6564 entries (see Figure E1A) by a batch process (~3400 entries) or by manual Blast searching (~6500 entries). This was followed by cross-mapping to other databases (EnsEMBL, KEGG, gene names, etc.) to enable the use of specific analysis tools. Application of a statistical significance-cut-off value of 0.05 or less resulted in a dataset of 2521 proteins (Figure E1B), and an additional cut-off of a fold-change of at least 2 reduced the dataset to 1480 entries (Figure E1C). All further analyses were performed using this last dataset.

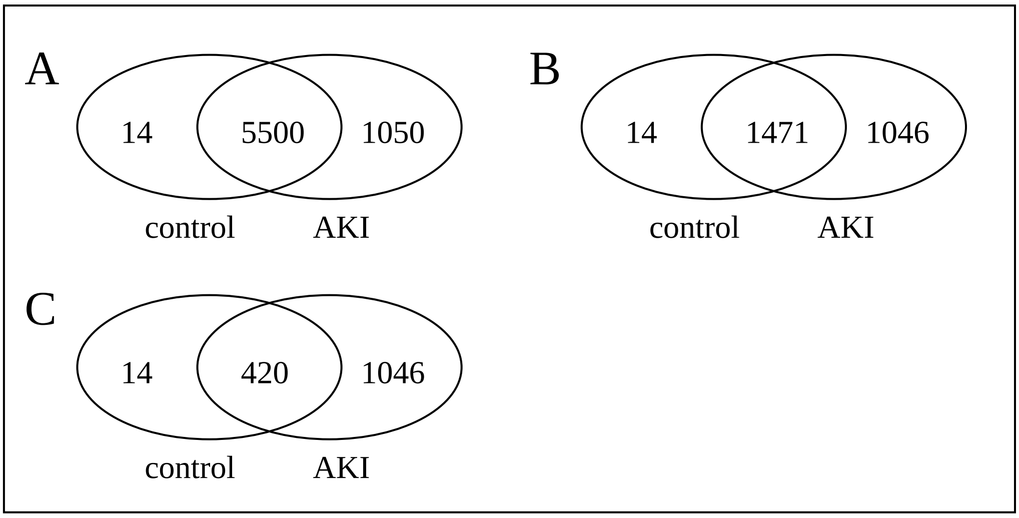

Figure E1. Distribution of observed proteins in control and AKI samples. The Venn diagrams depict the division of molecules within the control and AKI groups in (A) the whole dataset, (B) after applying a statistical significance cut-off level of 0.05, and (C) a further restriction to only include molecules which show altered expression levels of at least 2 between the two groups.

GO analysis

The dataset containing the 1480 proteins was split into two groups, up- (1449 molecules) and down-regulated (31 proteins), and both were individually analysed using Gene Ontology (GO) terms. Several different analyses were performed, each one using a different algorithm and procedure to generate a list of enriched/over-

represented GO-terms. Each of these methods have their own strengths and weaknesses, and an optimised way to extract as much information from the AKI datasets is by executing all strategies, and incorporating the results further downstream in the pathway assembly step.

<u>GO analysis using ClueGO</u>

The first analysis was performed using the CytoScape plug-in ClueGO (http://apps.cytoscape.org/apps/cluego). A network is created with kappa statistics and reflects the relationships between the terms based on the similarity of their associated molecules. The graphical results of the GO classes ‘molecular function’ and ‘biological process’ of the 1449 up-regulated proteins are shown in Figure E2, and the tabulated version is shown in Table E1.

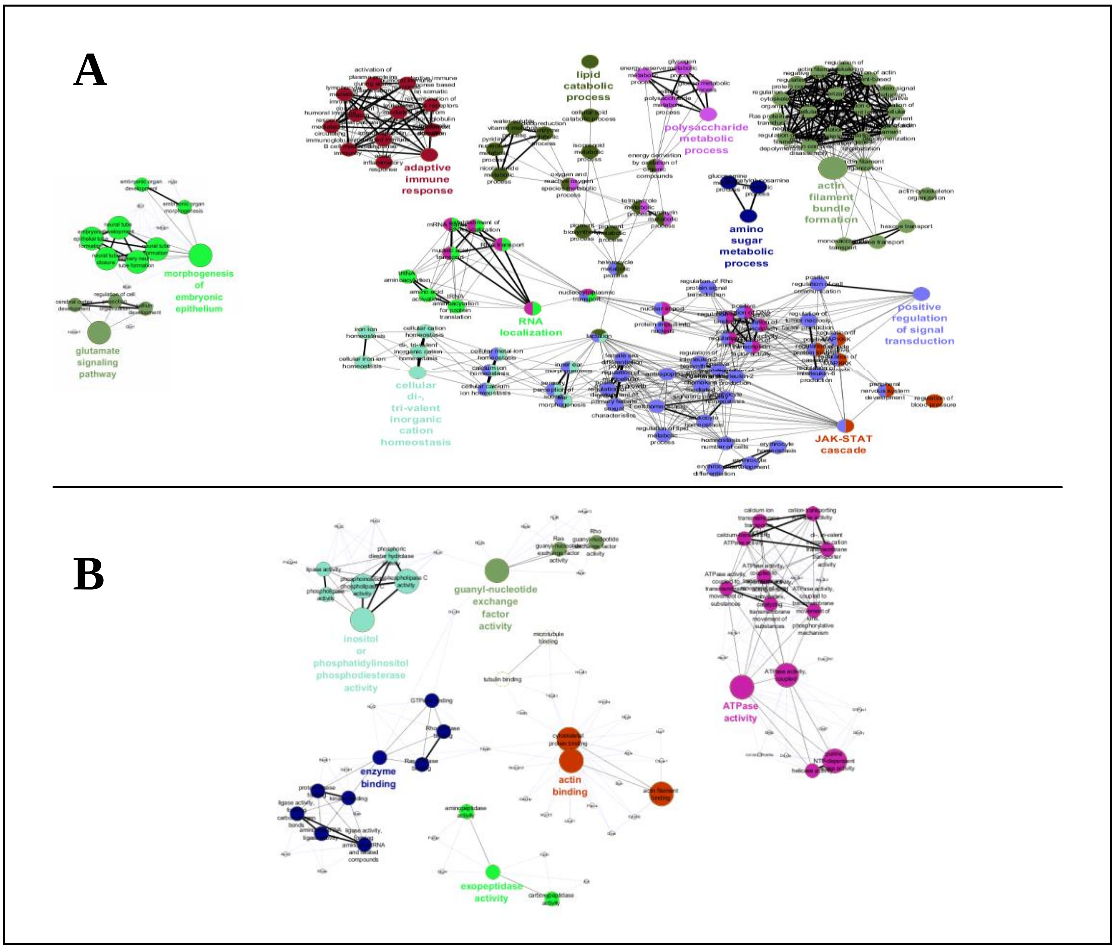

Figure E2. Gene ontology analysis. The enrichment analysis of GO-terms of all statistically and fold-change significant up-regulated proteins was done using ClueGO and querying either for (A) biological process or (B) molecular function GO-subgroups.

Additionally, ClueGO also allows mapping using pathway terms derived from KEGG as well as Reactome, and these results are also included in Table E1.

Table E1. GO analysis using ClueGO. AKI-modulated enriched GO/Reactome/KEGG-terms for either the up- or down-regulated groups are listed by source, term-ID and description, the number of associated entries found in the qury-list, p-value and the list of molecules mapped to the term. The cut-off value was 0.05.

GO-ID	GOTerm	Nr. Genes	% Associated Proteins	Term PValue	Associated Proteins Found
GO_BiologicalProcess –up-regulated dataset					
GO:0044267	cellular protein metabolic process	205	8.983348	3.03E-08	[Aars, Aars2, Ace, Adam26a, Adamts2, Adrbk1, Aebp1, Afg3l1, Agbl1, Araf, Axl, B3gnt7, Capn3, Cct8, Cdk2, Cdk3, Cdk4, Cdk6, Cdkl1, Cfb, Cfh, Chek1, Cit, Cts8, Ctsw, Cul1, Cul2, Cul5, Dapk1, Dhcr24, Dnaja1, Dnaja2, Dnaja4, Dnajb5, Dnajc16, Dnajc17, Dpep2, Dpp4, Dpp7, Dpp8, Dub1, Dub2a, Dync2h1, Dzip3, EG317677, Ece2, Egfr, Eif2s1, Eif2s3x, Eif2s3y, Eif3l, Eif3m, Eif4b, Eif4e, Eif4e2, Eif4g1, Eif6, Epha7, Ephb6, F10, F7, Fbxo43, Fbxo47, Fert2, Fgfr1, Fgfr2, Fgfr4, Fgr, Fkbp2, Folh1, Fyn, Gna15, Grk4, Grm1, Gspt1, Hck, Hectd2, Hecw2, Hsph1, Ick, Ide, Ift172, Irak4, Jak3, Kalrn, Khsrp, Ksr2, Lck, Lonrf3, Lyn, Madd, Mak, Man1b1, Man2a1, Map3k13, Map3k14, Map3k15, Map3k2, Map3k9, Map4k3, Mapk11, Mapk13, Mapk8ip3, Mark4, Mbd3, Mgat3, Mlkl, Mmp2, Mmp20, Mrpl2, Myo3a, Naalad2, Nckap1, Ndst2, Nedd4l, Nek5, Nfx1, Nosip, Npepl1, Osm, Pa2g4, Pan3, Pdk2, Pepd, Pik3c3, Pkdcc, Pkn2, Pmpca, Ppic, Ppil2, Ppm1a, Ppm1d, Ppp1ca, Ppp1cc, Ppp2r5c, Ppp3cb, Prkd1, Prkg2, Prmt1, Psma1, Psmd8, Ptprg, Ptprj, Ptprt, Ptprv, Pxn, Rabgef1, Rabggta, Rars2, Rbm3, Rbp3, Ren1, Rfwd2, Ripk4, Rnf123, Rnf6, Rnf7, Rngtt, Ror1, Rpl28, Rps6ka5, Senp6, Sgsh, Slmap, Sltm, Smok3a, Socs3, Ssh2, St6galnac6, Stambp, Stat4, Tars2, Tarsl2, Tcfe2a, Tec, Tesk2, Tgfb1, Tmprss12, Tmprss7, Tpp1, Trappc10, Trim33, Trip12, Tsfm, Ttc9, Ttll8, Ube2m, Ube2o, Ube3a, Ublcp1, Ubqlnl, Ubr1, Uchl3, Ufd1l, Usp14, Usp16, Usp26, Usp42, Usp47, Usp54, Vprbp, Wars, Wnk2, Wnk4, Yes1]
GO:0019538	protein metabolic process	206	8.91389	5.99E-08	[Aars, Aars2, Ace, Adam26a, Adamts2, Adrbk1, Aebp1, Afg3l1, Agbl1, Araf, Axl, B3gnt7, Capn3, Cct8, Cdk2, Cdk3, Cdk4, Cdk6, Cdkl1, Cfb, Cfh, Chek1, Cit, Cts8, Ctsw, Cul1, Cul2, Cul5, Dapk1, Dhcr24, Dnaja1, Dnaja2, Dnaja4, Dnajb5, Dnajc16, Dnajc17, Dpep2, Dpp4, Dpp7, Dpp8, Dub1, Dub2a, Dync2h1, Dzip3, EG317677, Ece2, Egfr, Eif2s1, Eif2s3x, Eif2s3y, Eif3l, Eif3m, Eif4b, Eif4e,

					Eif4e2, Eif4g1, Eif6, Epha7, Ephb6, F10, F7, Fbxo43, Fbxo47, Fert2, Fgfr1, Fgfr2, Fgfr4, Fgr, Fkbp2, Folh1, Fyn, Gna15, Grk4, Grm1, Gspt1, Hck, Hectd2, Hecw2, Hsph1, Ick, Ide, Ift172, Irak4, Jak3, Kalrn, Khsrp, Ksr2, Lck, Lonrf3, Lyn, Madd, Mak, Man1b1, Man2a1, Map3k13, Map3k14, Map3k15, Map3k2, Map3k9, Map4k3, Mapk11, Mapk13, Mapk8ip3, Mark4, Mbd3, Mgat3, Mlkl, Mmp2, Mmp20, Mrpl2, Myo3a, Naalad2, Nckap1, Ndst2, Nedd4l, Nek5, Nfx1, Nosip, Npepl1, Osm, Pa2g4, Pan3, Pdk2, Pepd, Pik3c3, Pkdcc, Pkn2, Plod3, Pmpca, Ppic, Ppil2, Ppm1a, Ppm1d, Ppp1ca, Ppp1cc, Ppp2r5c, Ppp3cb, Prkd1, Prkg2, Prmt1, Psma1, Psmd8, Ptprg, Ptprj, Ptprt, Ptprv, Pxn, Rabgef1, Rabggta, Rars2, Rbm3, Rbp3, Ren1, Rfwd2, Ripk4, Rnf123, Rnf6, Rnf7, Rngtt, Ror1, Rpl28, Rps6ka5, Senp6, Sgsh, Slmap, Sltm, Smok3a, Socs3, Ssh2, St6galnac6, Stambp, Stat4, Tars2, Tarsl2, Tcfe2a, Tec, Tesk2, Tgfb1, Tmprss12, Tmprss7, Tpp1, Trappc10, Trim33, Trip12, Tsfm, Ttc9, Ttll8, Ube2m, Ube2o, Ube3a, Ublcp1, Ubqlnl, Ubr1, Uchl3, Ufd1l, Usp14, Usp16, Usp26, Usp42, Usp47, Usp54, Vprbp, Wars, Wnk2, Wnk4, Yes1]
GO:0044260	cellular macromolecule metabolic process	348	7.990815	8.76E-08	[Aars, Aars2, Abra, Ace, Actr5, Adam26a, Adamts2, Adnp2, Adrbk1, Aebp1, Afg3l1, Agbl1, Aire, Apoa4, Aptx, Araf, Arhgap22, Asxl2, Atoh8, Axl, B3gnt7, Baz2a, Bnc1, Bnc2, Brip1, Btf3, Capn3, Ccno, Cct8, Cdk2, Cdk3, Cdk4, Cdk6, Cdkl1, Cebpz, Cfb, Cfh, Chd7, Chek1, Chid1, Cic, Cit, Cpsf2, Cpsf6, Creb3l2, Cts8, Ctsw, Cul1, Cul2, Cul5, Cux2, Dapk1, Dhcr24, Dicer1, Dis3, Dlg1, Dmrt1, Dnaja1, Dnaja2, Dnaja4, Dnajb5, Dnajc16, Dnajc17, Dpep2, Dpp4, Dpp7, Dpp8, Dub1, Dub2a, Dync2h1, Dzip3, E2f8, EG317677, Ece2, Egfr, Eif2s1, Eif2s3x, Eif2s3y, Eif3l, Eif3m, Eif4a3, Eif4b, Eif4e, Eif4e2, Eif4g1, Eif6, Epc2, Epha7, Ephb6, Ercc2, Esco1, Esr2, F10, F7, Fancm, Fbxo43, Fbxo47, Fert2, Fgfr1, Fgfr2, Fgfr4, Fgr, Fhl2, Fip1l1, Fkbp2, Folh1, Foxj3, Foxl2, Foxn3, Fyn, G6pc, Gaa, Gabpb2, Gbe1, Gfi1, Gli3, Glis1, Gna15, Grhl2, Grk4, Grm1, Gspt1, Gtf2h1, Hck, Hdgf, Hectd2, Hecw2, Hivep3, Hltf, Hoxb13, Hsf1, Hsph1, Ick, Ide, Ift172, Ikzf2, Ikzf4, Irak4, Jak3, Kalrn, Khsrp, Ksr2, Larp7, Lcat, Lck, Lig1, Lig3, Lin54, Lonrf3, Lyn, Madd, Mak, Man1b1, Man2a1, Map3k13, Map3k14, Map3k15, Map3k2, Map3k9, Map4k3, Mapk11, Mapk13, Mapk8ip3, Mark4, Mbd3, Mcm2, Mcm7, Med12l, Med15, Med27, Mgat3, Mkl2, Mlh3, Mlkl, Mmp2, Mmp20, Mrpl2, Msh2, Mus81, Mutyh, Mybl1, Mycbp, Myef2, Myo3a, Naalad2, Nckap1, Ncoa2, Ndst2, Nedd4l, Nek5, Nfx1, Nono, Nosip, Notch2, Npat, Npepl1, Nr1h3, Nr2c2, Nr3c1, Nr3c2, Orc3l, Osm, Pa2g4, Pan3, Papolg, Pawr, Pdk2, Pelp1, Pepd, Per3, Pgr, Phf17, Phrf1, Phtf1, Pik3c3, Pkdcc, Pkn2, Pmf1, Pmpca, Pms1, Polg, Polr2b, Pparg, Ppfibp1, Ppic, Ppil2, Ppm1a, Ppm1d, Ppp1ca, Ppp1cc, Ppp1r3a, Ppp2r5c, Ppp3cb, Prdm5, Prkd1, Prkg2, Prmt1, Prpf6, Psip1, Psma1, Psmd8, Ptprg, Ptprj, Ptprt, Ptprv, Purb, Pus1, Pxn, Pygb, Pygl, Rabgef1, Rabggta, Rars2, Rbm28, Rbm3, Rbm39, Rbp3, Relb, Ren1, Rfc5, Rfwd2, Ripk4, Rnf123, Rnf6, Rnf7, Rngtt, Ror1, Rpl28, Rpp38, Rps6ka5, Ruvbl1, Senp6, Sf1, Sgsh, Sim1, Sin3b, Slmap, Sltm, Smok3a, Snrpa, Snrpb, Socs3, Sox6, Sox7, Ssh2, St6galnac6, Stambp, Stat4, Stat5b, Swap70, Taf1a, Tars2, Tarsl2, Tcfe2a, Tcof1, Tec, Tesk2, Tfip11, Tgfb1, Thrb, Tmprss12, Tmprss7, Top1, Top2b, Top3a, Tpp1, Trappc10, Trdmt1, Trim28, Trim33, Trip12, Trps1, Trrap, Tsfm, Tshz1, Ttc9, Ttll8, Ube2m, Ube2o, Ube3a, Ublcp1, Ubqlnl, Ubr1, Uchl3, Ufd1l,

					Unc13a, Usp14, Usp16, Usp26, Usp42, Usp47, Usp54, Vprbp, Vps36, Wars, Whsc1l1, Wnk2, Wnk4, Wrn, Xrcc3, Yes1, Zbtb17, Zfp28, Zfp287, Zfp446, Zfy1, Zfy2, Zhx3]
GO:0006793	phosphorus metabolic process	81	10.72848	1.50E-06	[Adrbk1, Araf, Atp5g2, Axl, Cdk2, Cdk3, Cdk4, Cdk6, Cdkl1, Chek1, Cit, Dapk1, Egfr, Epha7, Ephb6, Fert2, Fgfr1, Fgfr2, Fgfr4, Fgr, Fyn, Gnptab, Grk4, Grm1, Hck, Ick, Irak4, Jak3, Kalrn, Ksr2, Lck, Lyn, Madd, Mak, Map3k13, Map3k14, Map3k15, Map3k2, Map3k9, Map4k3, Mapk11, Mapk13, Mapk8ip3, Mark4, Mlkl, Msh2, Myo3a, Nek5, Osm, Pan3, Pdk2, Pik3c3, Pkdcc, Pkn2, Ppm1a, Ppm1d, Ppp1ca, Ppp1cc, Ppp3cb, Prkd1, Prkg2, Ptprg, Ptprj, Ptprt, Ptprv, Pxn, Ripk4, Rngtt, Ror1, Rps6ka5, Sbf1, Sltm, Smok3a, Ssh2, Stat4, Tec, Tesk2, Tgfb1, Wnk2, Wnk4, Yes1]
GO:0006796	phosphate metabolic process	81	10.72848	1.50E-06	[Adrbk1, Araf, Atp5g2, Axl, Cdk2, Cdk3, Cdk4, Cdk6, Cdkl1, Chek1, Cit, Dapk1, Egfr, Epha7, Ephb6, Fert2, Fgfr1, Fgfr2, Fgfr4, Fgr, Fyn, Gnptab, Grk4, Grm1, Hck, Ick, Irak4, Jak3, Kalrn, Ksr2, Lck, Lyn, Madd, Mak, Map3k13, Map3k14, Map3k15, Map3k2, Map3k9, Map4k3, Mapk11, Mapk13, Mapk8ip3, Mark4, Mlkl, Msh2, Myo3a, Nek5, Osm, Pan3, Pdk2, Pik3c3, Pkdcc, Pkn2, Ppm1a, Ppm1d, Ppp1ca, Ppp1cc, Ppp3cb, Prkd1, Prkg2, Ptprg, Ptprj, Ptprt, Ptprv, Pxn, Ripk4, Rngtt, Ror1, Rps6ka5, Sbf1, Sltm, Smok3a, Ssh2, Stat4, Tec, Tesk2, Tgfb1, Wnk2, Wnk4, Yes1]
GO:0043283	biopolymer metabolic process	299	7.918432	2.95E-06	[Aars, Aars2, Abra, Actr5, Adnp2, Adrbk1, Aebp1, Afg3l1, Aire, Aptx, Araf, Arhgap22, Asxl2, Atoh8, Axl, B3gnt7, Baz2a, Bnc1, Bnc2, Brip1, Btf3, Ccno, Cdk2, Cdk3, Cdk4, Cdk6, Cdkl1, Cebpz, Chd7, Chek1, Chid1, Cic, Cit, Cpsf2, Cpsf6, Creb3l2, Cul1, Cul2, Cul5, Cux2, Dapk1, Dhcr24, Dicer1, Dis3, Dmrt1, Dub1, Dub2a, Dync2h1, Dzip3, E2f8, Ece2, Egfr, Eif2s1, Eif2s3x, Eif2s3y, Eif3l, Eif3m, Eif4a3, Eif4b, Eif4e, Eif4e2, Eif4g1, Eif6, Epc2, Epha7, Ephb6, Ercc2, Esco1, Esr2, Fancm, Fbxo43, Fbxo47, Fert2, Fgfr1, Fgfr2, Fgfr4, Fgr, Fhl2, Fip1l1, Foxj3, Foxl2, Foxn3, Fyn, G6pc, Gaa, Gabpb2, Gbe1, Gfi1, Gli3, Glis1, Gna15, Grhl2, Grk4, Grm1, Gspt1, Gtf2h1, Hck, Hdgf, Hectd2, Hecw2, Hivep3, Hltf, Hoxb13, Hsf1, Ick, Ift172, Ikzf2, Ikzf4, Irak4, Jak3, Kalrn, Khsrp, Ksr2, Larp7, Lck, Lig1, Lig3, Lin54, Lyn, Madd, Mak, Man1b1, Man2a1, Map3k13, Map3k14, Map3k15, Map3k2, Map3k9, Map4k3, Mapk11, Mapk13, Mapk8ip3, Mark4, Mbd3, Mcm2, Mcm7, Med12l, Med15, Med27, Mgat3, Mkl2, Mlh3, Mlkl, Mrpl2, Msh2, Mus81, Mutyh, Mybl1, Mycbp, Myef2, Myo3a, Ncoa2, Nedd4l, Nek5, Nfx1, Nono, Nosip, Notch2, Npat, Nr1h3, Nr2c2, Nr3c1, Nr3c2, Orc3l, Osm, Pa2g4, Pan3, Papolg, Pawr, Pdk2, Pelp1, Per3, Pgr, Phf17, Phrf1, Phtf1, Pik3c3, Pkdcc, Pkn2, Pmf1, Pms1, Polg, Polr2b, Pparg, Ppfibp1, Ppil2, Ppm1a, Ppm1d, Ppp1ca, Ppp1cc, Ppp1r3a, Ppp2r5c, Ppp3cb, Prdm5, Prkd1, Prkg2, Prmt1, Prpf6, Psip1, Psma1, Ptprg, Ptprj, Ptprt, Ptprv, Purb, Pus1, Pxn, Pygb, Pygl, Rabgef1, Rabggta, Rars2, Rbm28, Rbm3, Rbm39, Relb, Ren1, Rfc5, Rfwd2, Ripk4, Rnf123, Rnf6, Rnf7, Rngtt, Ror1, Rpl28, Rpp38, Rps6ka5, Ruvbl1, Senp6, Sf1, Sim1, Sin3b, Sltm, Smok3a, Snrpa, Snrpb, Socs3, Sox6, Sox7, Ssh2, St6galnac6, Stambp, Stat4, Stat5b, Swap70, Taf1a, Tars2, Tarsl2, Tcfe2a, Tcof1, Tec, Tesk2, Tfip11, Tgfb1, Thrb, Top1, Top2b, Top3a, Trappc10, Trdmt1, Trim28, Trim33, Trip12, Trps1, Trrap, Tsfm, Tshz1, Ttll8, Ube2m, Ube2o, Ube3a, Ublcp1, Ubqlnl, Ubr1, Uchl3, Ufd1l, Usp14, Usp16, Usp26, Usp42, Usp47,

					Usp54, Vprbp, Vps36, Wars, Whsc1l1, Wnk2, Wnk4, Wrn, Xrcc3, Yes1, Zbtb17, Zfp28, Zfp287, Zfp446, Zfy1, Zfy2, Zhx3]
GO:0043687	post-translational protein modification	92	10.15453	3.84E-06	[Adrbk1, Araf, Axl, Cdk2, Cdk3, Cdk4, Cdk6, Cdkl1, Chek1, Cit, Dapk1, Dhcr24, Dub1, Dub2a, Dync2h1, Ece2, Egfr, Epha7, Ephb6, Fert2, Fgfr1, Fgfr2, Fgfr4, Fgr, Fyn, Gna15, Grk4, Grm1, Hck, Ick, Ift172, Irak4, Jak3, Kalrn, Ksr2, Lck, Lyn, Madd, Mak, Map3k13, Map3k14, Map3k15, Map3k2, Map3k9, Map4k3, Mapk11, Mapk13, Mapk8ip3, Mark4, Mbd3, Mlkl, Myo3a, Nek5, Nosip, Osm, Pan3, Pdk2, Pik3c3, Pkdcc, Pkn2, Ppil2, Ppm1a, Ppm1d, Ppp1ca, Ppp1cc, Ppp3cb, Prkd1, Prkg2, Prmt1, Ptprg, Ptprj, Ptprt, Ptprv, Pxn, Ren1, Ripk4, Rngtt, Ror1, Rps6ka5, Sltm, Smok3a, Ssh2, Stat4, Tec, Tesk2, Tgfb1, Ube2m, Ube2o, Uchl3, Wnk2, Wnk4, Yes1]
GO:0006468	protein amino acid phosphorylation	66	11.07383	5.84E-06	[Adrbk1, Araf, Axl, Cdk2, Cdk3, Cdk4, Cdk6, Cdkl1, Chek1, Cit, Dapk1, Egfr, Epha7, Ephb6, Fert2, Fgfr1, Fgfr2, Fgfr4, Fgr, Fyn, Grk4, Grm1, Hck, Ick, Irak4, Jak3, Kalrn, Ksr2, Lck, Lyn, Madd, Mak, Map3k13, Map3k14, Map3k15, Map3k2, Map3k9, Map4k3, Mapk11, Mapk13, Mapk8ip3, Mark4, Mlkl, Myo3a, Nek5, Osm, Pan3, Pdk2, Pik3c3, Pkdcc, Pkn2, Prkd1, Prkg2, Pxn, Ripk4, Ror1, Rps6ka5, Sltm, Smok3a, Stat4, Tec, Tesk2, Tgfb1, Wnk2, Wnk4, Yes1]
GO:0016310	phosphorylation	69	10.73095	9.08E-06	[Adrbk1, Araf, Atp5g2, Axl, Cdk2, Cdk3, Cdk4, Cdk6, Cdkl1, Chek1, Cit, Dapk1, Egfr, Epha7, Ephb6, Fert2, Fgfr1, Fgfr2, Fgfr4, Fgr, Fyn, Gnptab, Grk4, Grm1, Hck, Ick, Irak4, Jak3, Kalrn, Ksr2, Lck, Lyn, Madd, Mak, Map3k13, Map3k14, Map3k15, Map3k2, Map3k9, Map4k3, Mapk11, Mapk13, Mapk8ip3, Mark4, Mlkl, Msh2, Myo3a, Nek5, Osm, Pan3, Pdk2, Pik3c3, Pkdcc, Pkn2, Prkd1, Prkg2, Pxn, Ripk4, Ror1, Rps6ka5, Sltm, Smok3a, Stat4, Tec, Tesk2, Tgfb1, Wnk2, Wnk4, Yes1]
GO:0043412	biopolymer modification	108	9.254499	3.27E-05	[Aars, Adrbk1, Araf, Axl, B3gnt7, Cdk2, Cdk3, Cdk4, Cdk6, Cdkl1, Chek1, Cit, Dapk1, Dhcr24, Dub1, Dub2a, Dync2h1, Ece2, Egfr, Epha7, Ephb6, Fert2, Fgfr1, Fgfr2, Fgfr4, Fgr, Fyn, Gna15, Grk4, Grm1, Hck, Hectd2, Hecw2, Ick, Ift172, Irak4, Jak3, Kalrn, Ksr2, Lck, Lyn, Madd, Mak, Man2a1, Map3k13, Map3k14, Map3k15, Map3k2, Map3k9, Map4k3, Mapk11, Mapk13, Mapk8ip3, Mark4, Mbd3, Mgat3, Mlkl, Myo3a, Nedd4l, Nek5, Nosip, Osm, Pan3, Pdk2, Pik3c3, Pkdcc, Pkn2, Ppil2, Ppm1a, Ppm1d, Ppp1ca, Ppp1cc, Ppp3cb, Prkd1, Prkg2, Prmt1, Ptprg, Ptprj, Ptprt, Ptprv, Pus1, Pxn, Rabggta, Ren1, Ripk4, Rngtt, Ror1, Rps6ka5, Sltm, Smok3a, Ssh2, St6galnac6, Stat4, Tec, Tesk2, Tgfb1, Trdmt1, Ttll8, Ube2m, Ube2o, Ube3a, Ublcp1, Ubqlnl, Uchl3, Usp14, Wnk2, Wnk4, Yes1]
GO:0006464	protein modification process	105	9.267431	3.47E-05	[Adrbk1, Araf, Axl, B3gnt7, Cdk2, Cdk3, Cdk4, Cdk6, Cdkl1, Chek1, Cit, Dapk1, Dhcr24, Dub1, Dub2a, Dync2h1, Ece2, Egfr, Epha7, Ephb6, Fert2, Fgfr1, Fgfr2, Fgfr4, Fgr, Fyn, Gna15, Grk4, Grm1, Hck, Hectd2, Hecw2, Ick, Ift172, Irak4, Jak3, Kalrn, Ksr2, Lck, Lyn, Madd, Mak, Man2a1, Map3k13, Map3k14, Map3k15, Map3k2, Map3k9, Map4k3, Mapk11, Mapk13, Mapk8ip3, Mark4, Mbd3, Mgat3, Mlkl, Myo3a, Nedd4l, Nek5, Nosip, Osm, Pan3, Pdk2, Pik3c3, Pkdcc, Pkn2, Ppil2, Ppm1a, Ppm1d, Ppp1ca, Ppp1cc, Ppp3cb, Prkd1, Prkg2, Prmt1, Ptprg, Ptprj, Ptprt, Ptprv, Pxn, Rabggta, Ren1, Ripk4, Rngtt, Ror1, Rps6ka5, Sltm, Smok3a, Ssh2, St6galnac6, Stat4, Tec, Tesk2, Tgfb1, Ttll8, Ube2m, Ube2o, Ube3a, Ublcp1, Ubqlnl, Uchl3, Usp14, Wnk2, Wnk4, Yes1]

GO:0030518	steroid hormone receptor signaling pathway	5	50	1.81E-04	[Dnaja1, Ncoa2, Nr3c1, Pgr, Safb]
GO:0005977	glycogen metabolic process	8	26.66667	3.80E-04	[G6pc, Gaa, Gbe1, Ppp1ca, Ppp1cc, Ppp1r3a, Pygb, Pygl]
GO:0006073	glucan metabolic process	8	26.66667	3.80E-04	[G6pc, Gaa, Gbe1, Ppp1ca, Ppp1cc, Ppp1r3a, Pygb, Pygl]
GO:0006112	energy reserve metabolic process	8	24.24242	7.63E-04	[G6pc, Gaa, Gbe1, Ppp1ca, Ppp1cc, Ppp1r3a, Pygb, Pygl]
GO:0030036	actin cytoskeleton organization	19	14.28571	7.78E-04	[2610204M08Rik, Aif1, Avil, Capn3, Dbnl, Enah, Fhdc1, Fmn2, Fmnl3, Grid2ip, Kras, Lcp1, Mybpc3, Nckap1, Nisch, Shroom3, Sorbs1, Tmod1, Xirp1]
GO:0007216	metabotropic glutamate receptor signaling pathway	3	75	9.23E-04	[Grid2ip, Homer1, Homer3]
GO:0046578	regulation of Ras protein signal transduction	19	13.66906	0.00115	[Abra, Arap2, Arhgef12, Arhgef3, Arhgef6, Asap1, Evi5, Fgd5, Kalrn, Kras, Ngef, Rasgrf2, Rgnef, Sgsm1, Sgsm2, Tbc1d1, Tbc1d2b, Tbc1d9b, Usp6nl]
GO:0008105	asymmetric protein localization	4	44.44444	0.001471	[Dync2h1, Napa, Nckap1, Vangl2]
GO:0030522	intracellular receptor-mediated signaling pathway	5	33.33333	0.001657	[Dnaja1, Ncoa2, Nr3c1, Pgr, Safb]
GO:0005976	polysaccharide metabolic process	9	19.56522	0.001874	[Chid1, G6pc, Gaa, Gbe1, Ppp1ca, Ppp1cc, Ppp1r3a, Pygb, Pygl]
GO:0044264	cellular polysaccharide metabolic process	9	19.56522	0.001874	[Chid1, G6pc, Gaa, Gbe1, Ppp1ca, Ppp1cc, Ppp1r3a, Pygb, Pygl]
GO:0051056	regulation of small GTPase mediated signal transduction	21	12.42604	0.00218	[Abra, Arap2, Arhgef12, Arhgef3, Arhgef6, Asap1, Evi5, Fgd5, Kalrn, Kras, Ngef, Rapgef5, Rasgrf2, Rgnef, Sgsm1, Sgsm2, Sipa1l1, Tbc1d1, Tbc1d2b, Tbc1d9b, Usp6nl]
GO:0006265	DNA topological change	3	60	0.002199	[Top1, Top2b, Top3a]
GO:0009612	response to mechanical stimulus	6	25	0.002953	[Atp2b2, Chrna10, Fyn, Gfi1, Pkd1, Strbp]
GO:0007215	glutamate signaling pathway	4	36.36364	0.003483	[Grid2ip, Grin1, Homer1, Homer3]
GO:0006261	DNA-dependent DNA replication	7	21.21212	0.003675	[Mcm2, Mcm7, Mlh3, Msh2, Pms1, Polg, Top3a]
GO:0003009	skeletal muscle contraction	2	100	0.003886	[Gaa, Homer1]
GO:0021775	smoothened signaling pathway involved in ventral spinal cord interneuron specification	2	100	0.003886	[Gli2, Gli3]
GO:0021776	smoothened signaling pathway involved in spinal cord motor neuron cell fate specification	2	100	0.003886	[Gli2, Gli3]
GO:0021910	smoothened signaling pathway in ventral spinal	2	100	0.003886	[Gli2, Gli3]

	cord patterning				
GO:0031033	myosin filament assembly or disassembly	2	100	0.003886	[Mybpc3, Tmod1]
GO:0031034	myosin filament assembly	2	100	0.003886	[Mybpc3, Tmod1]
GO:0016485	protein processing	11	15.49296	0.0043	[Dhcr24, Dync2h1, Ece2, Egfr, Fert2, Fyn, Ift172, Lck, Lyn, Ren1, Sltm]
GO:0032313	regulation of Rab GTPase activity	7	20.58824	0.004383	[Evi5, Sgsm1, Sgsm2, Tbc1d1, Tbc1d2b, Tbc1d9b, Usp6nl]
GO:0032483	regulation of Rab protein signal transduction	7	20.58824	0.004383	[Evi5, Sgsm1, Sgsm2, Tbc1d1, Tbc1d2b, Tbc1d9b, Usp6nl]
GO:0043588	skin development	5	26.31579	0.005209	[Aars, Adamts2, Dhcr24, Ercc2, Fras1]
GO:0007165	signal transduction	165	5.161089	0.005292	[Aars, Adam26a, Adam30, Adamts2, Adcy1, Adcy6, Adcyap1r1, Adrbk1, Angptl4, Ank1, Apc2, Araf, Arap2, Arhgap19, Arhgap22, Arhgef12, Arhgef3, Arhgef6, Arl3, Arl5b, Baiap2l1, Bambi, Bmp8a, C3ar1, Ccr8, Cdk4, Chek1, Chn2, Cit, Cnih2, Col1a2, Col4a3, Dapk1, Dbnl, Dgka, Dnaja1, Dtx2, Egfr, Eif6, Epha7, Ephb6, Eps8l2, Fert2, Fgfr1, Fgfr2, Fgg, Frzb, Fyn, Gabra1, Gigyf1, Gli2, Gli3, Glmn, Gmip, Gna15, Gpr125, Gpr176, Gpr19, Grid2ip, Grin1, Grk4, Grm1, Homer1, Homer3, Hras1, Hrh4, Ick, Ift172, Il1rl1, Irak4, Irs2, Itga1, Itgam, Jak3, Kcnh8, Kras, Krt18, Ksr2, Lck, Lepr, Lifr, Lphn1, Lrdd, Lyn, Madd, Mapk11, Mapk8ip3, Msh2, Myo7a, Ncoa2, Ngef, Nisch, Nle1, Notch2, Nr3c1, Ntsr1, Olfr1033, Olfr1211, Olfr1289, Olfr134, Olfr1495, Olfr181, Olfr632, Olfr642, Olfr695, Olfr780, Olfr806, Olfr867, Osm, Pacsin1, Pde5a, Pdk2, Per3, Pgr, Phip, Pik3c3, Pkd1, Pkn2, Plcb2, Plce1, Plch2, Plcl1, Plekhm1, Plxna4, Ppp2r5c, Prkd1, Ptprv, Pxn, Qrfpr, Rala, Ralb, Rapgef5, Rasgrf2, Rassf2, Ren1, Rgnef, Rhot2, Rnf7, Rps6ka5, Rsu1, Rxfp1, Safb, Sema3c, Sema6a, Shc4, Sim1, Sltm, Socs3, Sorbs1, Stambp, Stat4, Stat5b, Tas1r2, Tas2r136, Tas2r143, Tec, Tgfb1, Tnfrsf11b, Tnfrsf1a, Unc13a, Unc5d, Vmn2r42, Vmn2r43, Vmn2r61, Wnt6]
GO:0006418	tRNA aminoacylation for protein translation	8	18.18182	0.005301	[Aars, Aars2, Khsrp, Rars2, Tars2, Tarsl2, Trappc10, Wars]
GO:0043038	amino acid activation	8	18.18182	0.005301	[Aars, Aars2, Khsrp, Rars2, Tars2, Tarsl2, Trappc10, Wars]
GO:0043039	tRNA aminoacylation	8	18.18182	0.005301	[Aars, Aars2, Khsrp, Rars2, Tars2, Tarsl2, Trappc10, Wars]
GO:0019216	regulation of lipid metabolic process	6	22.22222	0.005535	[Acsl4, Angptl4, Bbs4, Nr3c1, Sf1, Stat5b]
GO:0035239	tube morphogenesis	15	12.93103	0.006191	[Bbs4, Dicer1, Dlg1, Enah, Fgfr1, Gli2, Gli3, Gzf1, Hoxb13, Ift172, Nckap1, Ppp1ca, Pxn, Shroom3, Vangl2]
GO:0006268	DNA unwinding during replication	3	42.85714	0.007001	[Mcm2, Mcm7, Top3a]
GO:0007616	long-term memory	3	42.85714	0.007001	[Adcy1, Chst10, Grin1]
GO:0032392	DNA geometric change	3	42.85714	0.007001	[Mcm2, Mcm7, Top3a]
GO:0032508	DNA duplex unwinding	3	42.85714	0.007001	[Mcm2, Mcm7, Top3a]
GO:0045176	apical protein localization	3	42.85714	0.007001	[Napa, Nckap1, Vangl2]

GO:0051017	actin filament bundle formation	4	28.57143	0.009093	[Aif1, Dbnl, Lcp1, Sorbs1]
GO:0044262	cellular carbohydrate metabolic process	23	10.74766	0.009904	[Chid1, Fn3k, G6pc, Gaa, Gbe1, Gnpda2, Itih2, Khsrp, Man2a1, Man2b1, Ndst2, Otog, Pck2, Pdk2, Pgm3, Ppp1ca, Ppp1cc, Ppp1r3a, Pygb, Pygl, Renbp, Sgsh, Treh]
GO:0007530	sex determination	5	22.72727	0.0101	[Dmrt1, Foxl2, Sf1, Srd5a1, Zfp429]
GO:0006508	proteolysis	74	8.323959	0.010324	[Ace, Adam26a, Adamts2, Aebp1, Afg3l1, Agbl1, Capn3, Cfb, Cfh, Cts8, Ctsw, Cul1, Cul2, Cul5, Dpep2, Dpp4, Dpp7, Dpp8, Dub1, Dub2a, Dzip3, EG317677, Ece2, F10, F7, Fbxo43, Fbxo47, Folh1, Hectd2, Hecw2, Ide, Lonrf3, Man1b1, Mmp2, Mmp20, Naalad2, Nedd4l, Nfx1, Npepl1, Pa2g4, Pepd, Pmpca, Ppp2r5c, Psma1, Psmd8, Rabgef1, Rbp3, Ren1, Rfwd2, Rnf123, Rnf6, Rnf7, Senp6, Socs3, Stambp, Tcfe2a, Tmprss12, Tmprss7, Tpp1, Trim33, Trip12, Ube2m, Ube2o, Ube3a, Ubr1, Uchl3, Ufd1l, Usp14, Usp16, Usp26, Usp42, Usp47, Usp54, Vprbp]
GO:0032318	regulation of Ras GTPase activity	9	15.25424	0.010373	[Arap2, Asap1, Evi5, Sgsm1, Sgsm2, Tbc1d1, Tbc1d2b, Tbc1d9b, Usp6nl]
GO:0016579	protein deubiquitination	3	37.5	0.010686	[Dub1, Dub2a, Uchl3]
GO:0030517	negative regulation of axon extension	3	37.5	0.010686	[Rnf6, Rtn4, Sema3a]
GO:0048488	synaptic vesicle endocytosis	3	37.5	0.010686	[Amph, Stxbp1, Unc13a]
GO:0000086	G2/M transition of mitotic cell cycle	2	66.66666	0.011174	[Chek1, Ppm1d]
GO:0005980	glycogen catabolic process	2	66.66666	0.011174	[G6pc, Gaa]
GO:0006419	alanyl-tRNA aminoacylation	2	66.66666	0.011174	[Aars, Aars2]
GO:0006435	threonyl-tRNA aminoacylation	2	66.66666	0.011174	[Tars2, Tarsl2]
GO:0009251	glucan catabolic process	2	66.66666	0.011174	[G6pc, Gaa]
GO:0018894	dibenzo-p-dioxin metabolic process	2	66.66666	0.011174	[Cyp1a1, Cyp1a2]
GO:0018904	organic ether metabolic process	2	66.66666	0.011174	[Cyp1a1, Cyp1a2]
GO:0031050	dsRNA fragmentation	2	66.66666	0.011174	[Dicer1, Rbm3]
GO:0035195	gene silencing by miRNA	2	66.66666	0.011174	[Dicer1, Rbm3]
GO:0035196	gene silencing by miRNA, production of miRNAs	2	66.66666	0.011174	[Dicer1, Rbm3]
GO:0042982	amyloid precursor protein metabolic process	2	66.66666	0.011174	[Dhcr24, Dlg1]
GO:0050665	hydrogen peroxide biosynthetic process	2	66.66666	0.011174	[Cyp1a1, Cyp1a2]
GO:0051928	positive regulation of	2	66.66666	0.011174	[Atp2b2, Homer1]

	calcium ion transport				
GO:0030239	myofibril assembly	4	26.66667	0.011796	[Capn3, Mybpc3, Tmod1, Xirp1]
GO:0055002	striated muscle cell development	4	26.66667	0.011796	[Capn3, Mybpc3, Tmod1, Xirp1]
GO:0007276	gamete generation	28	9.964413	0.012701	[Adam26a, Adamts2, Adcyap1r1, Asz1, Bbs4, Bmp8a, Cit, Crtap, D1Pas1, Dazap1, Dnaja1, Fmn2, Hsf1, Micalcl, Morc1, Msh2, Nlrp14, Nr2c2, Pgm3, Pgr, Rnf114, Rnf17, Sbf1, Spata6, Strbp, Svs2, Tgfb1, Tial1]
GO:0035295	tube development	20	10.92896	0.013181	[Adamts2, Bbs4, Cyp1a2, Dicer1, Dlg1, Enah, Fgfr1, Fgfr2, Fgfr4, Gli2, Gli3, Gzf1, Hoxb13, Ift172, Man2a1, Nckap1, Ppp1ca, Pxn, Shroom3, Vangl2]
GO:0015980	energy derivation by oxidation of organic compounds	9	14.51613	0.01419	[Cyp1a2, G6pc, Gaa, Gbe1, Ppp1ca, Ppp1cc, Ppp1r3a, Pygb, Pygl]
GO:0019221	cytokine and chemokine mediated signaling pathway	7	16.66667	0.014269	[Irak4, Jak3, Krt18, Lifr, Stat4, Stat5b, Tnfrsf1a]
GO:0006040	amino sugar metabolic process	5	20.83333	0.014704	[Chid1, Fn3k, Gnpda2, Pgm3, Renbp]
GO:0042743	hydrogen peroxide metabolic process	4	25	0.014966	[Cyp1a1, Cyp1a2, Mpo, Tpo]
GO:0055001	muscle cell development	4	25	0.014966	[Capn3, Mybpc3, Tmod1, Xirp1]
GO:0030879	mammary gland development	6	18.18182	0.01507	[Gli2, Gli3, Irs2, Pgr, Rxfp1, Tgfb1]
GO:0016601	Rac protein signal transduction	3	33.33333	0.015293	[Dbnl, Eps8l2, Nisch]
GO:0006457	protein folding	14	12.06897	0.018042	[Aars, Cct8, Dnaja1, Dnaja2, Dnaja4, Dnajb5, Dnajc16, Dnajc17, Fkbp2, Hsph1, Ppic, Ppil2, Slmap, Ttc9]
GO:0006310	DNA recombination	9	13.84615	0.018957	[Lig1, Lig3, Msh2, Mus81, Nono, Ruvbl1, Swap70, Wrn, Xrcc3]
GO:0035023	regulation of Rho protein signal transduction	9	13.84615	0.018957	[Abra, Arhgef12, Arhgef3, Arhgef6, Fgd5, Kalrn, Ngef, Rasgrf2, Rgnef]
GO:0006259	DNA metabolic process	31	9.567902	0.019464	[Aptx, Brip1, Ccno, Chek1, Epc2, Ercc2, Esco1, Fancm, Gtf2h1, Lig1, Lig3, Mcm2, Mcm7, Mlh3, Msh2, Mus81, Mutyh, Nono, Orc3l, Pms1, Polg, Ppfibp1, Rfc5, Ruvbl1, Swap70, Top1, Top2b, Top3a, Trdmt1, Wrn, Xrcc3]
GO:0002009	morphogenesis of an epithelium	14	11.86441	0.019488	[Bbs4, Enah, F11r, Fgfr2, Fras1, Gli2, Hoxb13, Ift172, Nckap1, Notch2, Pgr, Pparg, Shroom3, Vangl2]
GO:0051129	negative regulation of cellular component organization	9	13.63636	0.020777	[Avil, Cit, Dbnl, Pacsin1, Rnf6, Rtn4, Scin, Sema3a, Tmod3]
GO:0006013	mannose metabolic process	3	30	0.020848	[Man2a1, Man2b1, Renbp]
GO:0007439	ectodermal gut development	3	30	0.020848	[Gli2, Gli3, Nckap1]
GO:0048567	ectodermal gut morphogenesis	3	30	0.020848	[Gli2, Gli3, Nckap1]

GO:0009404	toxin metabolic process	2	50	0.021425	[Cyp1a1, Cyp1a2]
GO:0016441	posttranscriptional gene silencing	2	50	0.021425	[Dicer1, Rbm3]
GO:0032720	negative regulation of tumor necrosis factor production	2	50	0.021425	[H47, Hsf1]
GO:0035194	posttranscriptional gene silencing by RNA	2	50	0.021425	[Dicer1, Rbm3]
GO:0042255	ribosome assembly	2	50	0.021425	[Eif6, Surf6]
GO:0048570	notochord morphogenesis	2	50	0.021425	[Gli2, Nckap1]
GO:0060045	positive regulation of cardiac muscle cell proliferation	2	50	0.021425	[Fgfr1, Fgfr2]
GO:0046777	protein amino acid autophosphorylation	6	16.66667	0.022634	[Egfr, Fert2, Fyn, Lck, Lyn, Sltm]
GO:0031345	negative regulation of cell projection organization	4	22.22222	0.02279	[Cit, Rnf6, Rtn4, Sema3a]
GO:0050982	detection of mechanical stimulus	4	22.22222	0.02279	[Atp2b2, Chrna10, Fyn, Pkd1]
GO:0007283	spermatogenesis	22	10.09174	0.023531	[Adam26a, Adamts2, Adcyap1r1, Bbs4, Bmp8a, Cit, Crtap, D1Pas1, Dazap1, Dnaja1, Hsf1, Micalcl, Morc1, Nlrp14, Nr2c2, Pgm3, Rnf114, Rnf17, Sbf1, Spata6, Strbp, Svs2]
GO:0048232	male gamete generation	22	10.09174	0.023531	[Adam26a, Adamts2, Adcyap1r1, Bbs4, Bmp8a, Cit, Crtap, D1Pas1, Dazap1, Dnaja1, Hsf1, Micalcl, Morc1, Nlrp14, Nr2c2, Pgm3, Rnf114, Rnf17, Sbf1, Spata6, Strbp, Svs2]
GO:0048562	embryonic organ morphogenesis	5	18.51852	0.023945	[Fgfr2, Foxl2, Gli2, Gli3, Nckap1]
GO:0009611	response to wounding	27	9.540636	0.025378	[Abhd2, Cd163, Cfb, Cfh, Crip2, EG317677, F10, F7, Fga, Fgg, Ggt5, Gp5, H47, Hrh4, Kng1, Lamc1, Lcp1, Mefv, Nfkbid, Notch2, Otog, Pparg, Psma1, Tgfb1, Tnfrsf1a, Tnfsf4, Vnn1]
GO:0001763	morphogenesis of a branching structure	10	13.33333	0.025588	[Dicer1, Dlg1, Epha7, Fgfr1, Gli2, Gli3, Gzf1, Hoxb13, Ppp1ca, Pxn]
GO:0006800	oxygen and reactive oxygen species metabolic process	6	16.21622	0.025635	[Cyp1a1, Cyp1a2, Mpo, Ncf2, Sod3, Tpo]
GO:0007015	actin filament organization	6	16.21622	0.025635	[Aif1, Avil, Dbnl, Enah, Lcp1, Sorbs1]
GO:0034961	cellular biopolymer biosynthetic process	139	7.441114	0.025933	[Aars, Aars2, Abra, Actr5, Adnp2, Aebp1, Aire, Arhgap22, Asxl2, Atoh8, Baz2a, Bnc1, Bnc2, Brip1, Btf3, Cebpz, Chd7, Cic, Creb3l2, Cux2, Dmrt1, E2f8, Eif2s1, Eif2s3x, Eif2s3y, Eif3l, Eif3m, Eif4b, Eif4e, Eif4e2, Eif4g1, Eif6, Epc2, Ercc2, Esr2, Fhl2, Foxj3, Foxl2, Foxn3, Gabpb2, Gbe1, Gfi1, Gli3, Glis1, Grhl2, Gspt1, Gtf2h1, Hdgf, Hivep3, Hltf, Hoxb13, Hsf1, Ikzf2, Ikzf4, Khsrp, Lig1, Lig3, Lin54, Mbd3, Mcm2, Mcm7, Med12l, Med15, Med27, Mkl2, Mlh3, Mrpl2, Msh2, Mybl1, Mycbp, Myef2, Ncoa2, Nfx1, Nono, Notch2, Npat, Nr1h3, Nr2c2, Nr3c1, Nr3c2,

					Orc3l, Pa2g4, Papolg, Pawr, Pelp1, Per3, Pgr, Phf17, Phrf1, Phtf1, Pmf1, Pms1, Polg, Polr2b, Pparg, Prdm5, Psip1, Purb, Rars2, Rbm3, Rbm39, Relb, Rfc5, Rpl28, Ruvbl1, Sf1, Sim1, Sin3b, Sltm, Sox6, Sox7, Stat4, Stat5b, Taf1a, Tars2, Tarsl2, Tcfe2a, Tcof1, Thrb, Top1, Top3a, Trappc10, Trim28, Trim33, Trps1, Trrap, Tsfm, Tshz1, Vps36, Wars, Whsc1l1, Wrn, Zbtb17, Zfp28, Zfp287, Zfp446, Zfy1, Zfy2, Zhx3]
GO:0043284	biopolymer biosynthetic process	139	7.437132	0.025992	[Aars, Aars2, Abra, Actr5, Adnp2, Aebp1, Aire, Arhgap22, Asxl2, Atoh8, Baz2a, Bnc1, Bnc2, Brip1, Btf3, Cebpz, Chd7, Cic, Creb3l2, Cux2, Dmrt1, E2f8, Eif2s1, Eif2s3x, Eif2s3y, Eif3l, Eif3m, Eif4b, Eif4e, Eif4e2, Eif4g1, Eif6, Epc2, Ercc2, Esr2, Fhl2, Foxj3, Foxl2, Foxn3, Gabpb2, Gbe1, Gfi1, Gli3, Glis1, Grhl2, Gspt1, Gtf2h1, Hdgf, Hivep3, Hltf, Hoxb13, Hsf1, Ikzf2, Ikzf4, Khsrp, Lig1, Lig3, Lin54, Mbd3, Mcm2, Mcm7, Med12l, Med15, Med27, Mkl2, Mlh3, Mrpl2, Msh2, Mybl1, Mycbp, Myef2, Ncoa2, Nfx1, Nono, Notch2, Npat, Nr1h3, Nr2c2, Nr3c1, Nr3c2, Orc3l, Pa2g4, Papolg, Pawr, Pelp1, Per3, Pgr, Phf17, Phrf1, Phtf1, Pmf1, Pms1, Polg, Polr2b, Pparg, Prdm5, Psip1, Purb, Rars2, Rbm3, Rbm39, Relb, Rfc5, Rpl28, Ruvbl1, Sf1, Sim1, Sin3b, Sltm, Sox6, Sox7, Stat4, Stat5b, Taf1a, Tars2, Tarsl2, Tcfe2a, Tcof1, Thrb, Top1, Top3a, Trappc10, Trim28, Trim33, Trps1, Trrap, Tsfm, Tshz1, Vps36, Wars, Whsc1l1, Wrn, Zbtb17, Zfp28, Zfp287, Zfp446, Zfy1, Zfy2, Zhx3]
GO:0034960	cellular biopolymer metabolic process	175	7.267442	0.026332	[Aars, Aars2, Abra, Actr5, Adnp2, Aebp1, Aire, Aptx, Arhgap22, Asxl2, Atoh8, Baz2a, Bnc1, Bnc2, Brip1, Btf3, Ccno, Cebpz, Chd7, Chek1, Chid1, Cic, Cpsf2, Cpsf6, Creb3l2, Cux2, Dicer1, Dis3, Dmrt1, E2f8, Eif2s1, Eif2s3x, Eif2s3y, Eif3l, Eif3m, Eif4a3, Eif4b, Eif4e, Eif4e2, Eif4g1, Eif6, Epc2, Ercc2, Esco1, Esr2, Fancm, Fhl2, Fip1l1, Foxj3, Foxl2, Foxn3, G6pc, Gaa, Gabpb2, Gbe1, Gfi1, Gli3, Glis1, Grhl2, Gspt1, Gtf2h1, Hdgf, Hivep3, Hltf, Hoxb13, Hsf1, Ikzf2, Ikzf4, Khsrp, Larp7, Lig1, Lig3, Lin54, Mbd3, Mcm2, Mcm7, Med12l, Med15, Med27, Mkl2, Mlh3, Mrpl2, Msh2, Mus81, Mutyh, Mybl1, Mycbp, Myef2, Ncoa2, Nfx1, Nono, Notch2, Npat, Nr1h3, Nr2c2, Nr3c1, Nr3c2, Orc3l, Pa2g4, Pan3, Papolg, Pawr, Pelp1, Per3, Pgr, Phf17, Phrf1, Phtf1, Pmf1, Pms1, Polg, Polr2b, Pparg, Ppfibp1, Ppp1ca, Ppp1cc, Ppp1r3a, Prdm5, Prpf6, Psip1, Purb, Pus1, Pygb, Pygl, Rars2, Rbm28, Rbm3, Rbm39, Relb, Rfc5, Rngtt, Rpl28, Rpp38, Ruvbl1, Sf1, Sim1, Sin3b, Sltm, Snrpa, Snrpb, Sox6, Sox7, Stat4, Stat5b, Swap70, Taf1a, Tars2, Tarsl2, Tcfe2a, Tcof1, Tfip11, Thrb, Top1, Top2b, Top3a, Trappc10, Trdmt1, Trim28, Trim33, Trps1, Trrap, Tsfm, Tshz1, Vps36, Wars, Whsc1l1, Wrn, Xrcc3, Zbtb17, Zfp28, Zfp287, Zfp446, Zfy1, Zfy2, Zhx3]
GO:0000272	polysaccharide catabolic process	3	27.27273	0.02736	[Chid1, G6pc, Gaa]
GO:0006298	mismatch repair	3	27.27273	0.02736	[Mlh3, Msh2, Pms1]
GO:0044247	cellular polysaccharide catabolic process	3	27.27273	0.02736	[Chid1, G6pc, Gaa]
GO:0045214	sarcomere organization	3	27.27273	0.02736	[Capn3, Mybpc3, Xirp1]
GO:0006041	glucosamine metabolic process	4	21.05263	0.027478	[Chid1, Gnpda2, Pgm3, Renbp]

GO:0006044	N-acetylglucosamine metabolic process	4	21.05263	0.027478	[Chid1, Gnpda2, Pgm3, Renbp]
GO:0042384	cilium biogenesis	4	21.05263	0.027478	[Bbs4, Dync2h1, Ift172, Vangl2]
GO:0060021	palate development	4	21.05263	0.027478	[Chd7, Dlg1, Meox2, Tshz1]
GO:0030324	lung development	10	12.98701	0.027889	[Adamts2, Cyp1a2, Dicer1, Fgfr1, Fgfr2, Fgfr4, Gli2, Gli3, Man2a1, Ppp1ca]
GO:0016540	protein autoprocessing	6	15.78947	0.028889	[Egfr, Fert2, Fyn, Lck, Lyn, Sltm]
GO:0050954	sensory perception of mechanical stimulus	10	12.82051	0.029239	[Atp2b2, Chd7, Chrna10, Fyn, Myo15, Myo7a, Otog, Slc17a8, Strc, Thrb]
GO:0042472	inner ear morphogenesis	8	13.55932	0.02928	[Atp2b2, Chd7, Chrna10, Fgfr1, Fgfr2, Gfi1, Myo15, Myo7a]
GO:0010627	regulation of protein kinase cascade	10	12.65823	0.030724	[Card9, Cenpj, Grm1, Il1rl1, Lyn, Mapk8ip3, Nlrp12, Osm, Pdcd11, Sh3rf1]
GO:0030323	respiratory tube development	10	12.65823	0.030724	[Adamts2, Cyp1a2, Dicer1, Fgfr1, Fgfr2, Fgfr4, Gli2, Gli3, Man2a1, Ppp1ca]
GO:0031032	actomyosin structure organization	4	20	0.032699	[Capn3, Mybpc3, Tmod1, Xirp1]
GO:0051693	actin filament capping	4	20	0.032699	[Avil, Dbnl, Scin, Tmod3]
GO:0048731	system development	124	7.456404	0.033087	[Aars, Adamts2, Add2, Adrbk1, Ank1, Arhgap22, Atoh8, Atp2b2, Baiap2l1, Bbs4, Bmp8a, Capn3, Cdk6, Chd7, Chrna10, Cit, Crip2, Ctf1, Ctnna1, Ctnna2, Cul1, Cyp1a2, Dbh, Dhcr24, Dicer1, Dlg1, Dock7, Dsg4, Dync2h1, Enah, Epha7, Ercc2, Esr2, Fam83h, Fgfr1, Fgfr2, Fgfr4, Fmr1, Foxl2, Fras1, Fyn, Gaa, Gfi1, Gli2, Gli3, Grin1, Gzf1, Homer1, Hoxb13, Hsf1, Ift172, Irs2, Isl2, Jak3, Kirrel3, Kras, Lamc1, Lsr, Lyn, Man2a1, Mapk8ip3, Mbd3, Meox2, Mkl2, Mkx, Mmp2, Msh2, Mybpc3, Myo15, Myo7a, Napa, Nckap1, Ngef, Notch2, Nr3c1, Ntng2, Osm, Pcdhb17, Pgm3, Pgr, Pkd1, Plxna4, Postn, Pparg, Ppp1ca, Ppp1r13b, Ppp3cb, Ptprj, Relb, Ren1, Rgnef, Rnf6, Rtn4, Rxfp1, Scel, Sema3a, Sema3b, Sema3c, Sema6a, Sf1, Shroom3, Sim1, Slit3, Slitrk2, Slitrk4, Sltm, Sox6, Stat5b, Stxbp1, Swap70, Tcra, Tfip11, Tgfb1, Thrb, Tmod1, Tnfaip2, Top2b, Tpp1, Tshz1, Vangl2, Vcan, Wnt6, Xirp1, Zbtb17]
GO:0007442	hindgut morphogenesis	2	40	0.034242	[Gli2, Gli3]
GO:0007638	mechanosensory behavior	2	40	0.034242	[Gfi1, Strbp]
GO:0016188	synaptic vesicle maturation	2	40	0.034242	[Stxbp1, Unc13a]
GO:0021513	spinal cord dorsal/ventral patterning	2	40	0.034242	[Gli2, Gli3]
GO:0042537	benzene and derivative metabolic process	2	40	0.034242	[Cyp1a1, Cyp1a2]
GO:0045586	regulation of gamma-delta T cell differentiation	2	40	0.034242	[Lck, Stat5b]
GO:0045588	positive regulation of gamma-delta T cell differentiation	2	40	0.034242	[Lck, Stat5b]
GO:0046643	regulation of gamma-delta	2	40	0.034242	[Lck, Stat5b]

	T cell activation				
GO:0046645	positive regulation of gamma-delta T cell activation	2	40	0.034242	[Lck, Stat5b]
GO:0006139	nucleobase, nucleoside, nucleotide and nucleic acid metabolic process	169	7.243892	0.034487	[Aars, Aars2, Abra, Actr5, Adcy1, Adcy6, Adnp2, Adsl, Aebp1, Aire, Aptx, Arhgap22, Asxl2, Atic, Atoh8, Atp2b2, Atp5g2, Baz2a, Bnc1, Bnc2, Brip1, Btf3, Cbr2, Ccno, Cebpz, Chd7, Chek1, Cic, Cpsf2, Cpsf6, Creb3l2, Cux2, Dicer1, Dis3, Dmrt1, E2f8, Eif4a3, Eif4g1, Epc2, Ercc2, Esco1, Esr2, Fancm, Fhl2, Fip1l1, Foxj3, Foxl2, Foxn3, Gabpb2, Gfi1, Gli3, Glis1, Grhl2, Gtf2h1, Hdgf, Hivep3, Hltf, Hoxb13, Hsf1, Ikzf2, Ikzf4, Itpa, Khsrp, Larp7, Lig1, Lig3, Lin54, Mbd3, Mcm2, Mcm7, Med12l, Med15, Med27, Mkl2, Mlh3, Msh2, Mus81, Mutyh, Mybl1, Mycbp, Myef2, Ncf2, Ncoa2, Nfx1, Nme3, Nono, Notch2, Npat, Nr1h3, Nr2c2, Nr3c1, Nr3c2, Nt5c, Nt5c2, Nt5c3, Orc3l, Pa2g4, Pan3, Papolg, Pawr, Pde5a, Pelp1, Per3, Pgm3, Pgr, Phf17, Phrf1, Phtf1, Pmf1, Pms1, Polg, Polr2b, Pparg, Ppfibp1, Prdm5, Prpf6, Psip1, Purb, Pus1, Rars2, Rbm28, Rbm3, Rbm39, Relb, Rfc5, Rngtt, Rpp38, Ruvbl1, Sf1, Sim1, Sin3b, Sltm, Snrpa, Snrpb, Sox6, Sox7, Stat4, Stat5b, Swap70, Taf1a, Tars2, Tarsl2, Tcfe2a, Tcof1, Tfip11, Thrb, Top1, Top2b, Top3a, Trappc10, Trdmt1, Trim28, Trim33, Trps1, Trrap, Tshz1, Umps, Vps36, Wars, Whsc1l1, Wrn, Xrcc3, Zbtb17, Zfp28, Zfp287, Zfp446, Zfy1, Zfy2, Zhx3]
GO:0006692	prostanoid metabolic process	3	25	0.034824	[Ptgis, Tbxas1, Tnfrsf1a]
GO:0006693	prostaglandin metabolic process	3	25	0.034824	[Ptgis, Tbxas1, Tnfrsf1a]
GO:0006904	vesicle docking during exocytosis	3	25	0.034824	[Scfd2, Stxbp1, Stxbp2]
GO:0007143	female meiosis	3	25	0.034824	[Fmn2, Mlh3, Ppp1ca]
GO:0045005	maintenance of fidelity during DNA-dependent DNA replication	3	25	0.034824	[Mlh3, Msh2, Pms1]
GO:0050830	defense response to Gram-positive bacterium	3	25	0.034824	[Card9, Fgr, Hck]
GO:0000226	microtubule cytoskeleton organization	9	13.43284	0.035985	[Bbs4, Cenpj, Fmn2, Hook2, Hook3, Nuf2, Ranbp1, Sac3d1, Tekt4]
GO:0048754	branching morphogenesis of a tube	9	13.43284	0.035985	[Dicer1, Dlg1, Fgfr1, Gli2, Gli3, Gzf1, Hoxb13, Ppp1ca, Pxn]
GO:0042471	ear morphogenesis	9	13.23529	0.037224	[Atp2b2, Chd7, Chrna10, Fgfr1, Fgfr2, Gfi1, Myo15, Myo7a, Tshz1]
GO:0030835	negative regulation of actin filament depolymerization	4	19.04762	0.038459	[Avil, Dbnl, Scin, Tmod3]
GO:0007605	sensory perception of sound	9	13.04348	0.038645	[Atp2b2, Chd7, Chrna10, Myo15, Myo7a, Otog, Slc17a8, Strc, Thrb]
GO:0043087	regulation of GTPase activity	9	12.85714	0.040248	[Arap2, Asap1, Evi5, Sgsm1, Sgsm2, Tbc1d1, Tbc1d2b, Tbc1d9b, Usp6nl]

GO:0001701	in utero embryonic development	18	10.11236	0.041096	[Apba2, Btf3, Chd7, Ercc2, Fgfr1, Fgfr2, Hsf1, Man2a1, Mbd3, Mkl2, Msh2, Ncapg2, Nckap1, Nle1, Pkd1, Prmt1, Sbds, Tjp1]
GO:0051146	striated muscle cell differentiation	5	16.12903	0.041107	[Capn3, Kras, Mybpc3, Tmod1, Xirp1]
GO:0006753	nucleoside phosphate metabolic process	16	10.38961	0.042488	[Adcy1, Adcy6, Adsl, Atic, Atp2b2, Atp5g2, Cbr2, Itpa, Msh2, Ncf2, Nme3, Nt5c, Nt5c2, Nt5c3, Pde5a, Umps]
GO:0009117	nucleotide metabolic process	16	10.38961	0.042488	[Adcy1, Adcy6, Adsl, Atic, Atp2b2, Atp5g2, Cbr2, Itpa, Msh2, Ncf2, Nme3, Nt5c, Nt5c2, Nt5c3, Pde5a, Umps]
GO:0019218	regulation of steroid metabolic process	3	23.07692	0.043223	[Nr3c1, Sf1, Stat5b]
GO:0021522	spinal cord motor neuron differentiation	3	23.07692	0.043223	[Gli2, Gli3, Isl2]
GO:0043331	response to dsRNA	3	23.07692	0.043223	[Card9, Dicer1, Rbm3]
GO:0045076	regulation of interleukin-2 biosynthetic process	3	23.07692	0.043223	[Card9, Glmn, Stat5b]
GO:0048278	vesicle docking	3	23.07692	0.043223	[Scfd2, Stxbp1, Stxbp2]
GO:0001841	neural tube formation	6	14.28571	0.044563	[Bbs4, Enah, Ift172, Nckap1, Shroom3, Vangl2]
GO:0007596	blood coagulation	7	13.20755	0.045343	[F10, F7, Fga, Fgg, Gp5, Kng1, Otog]
GO:0030030	cell projection organization	26	9.187279	0.046378	[Atp2b2, Baiap2l1, Bbs4, Cit, Dync2h1, Enah, Epha7, Gli2, Ift172, Isl2, Lamc1, Mapk8ip3, Myo7a, Nckap1, Ntng2, Pxn, Rgnef, Sema3a, Sema6a, Slit3, Slitrk2, Slitrk4, Stxbp1, Tekt4, Top2b, Vangl2]
GO:0021514	ventral spinal cord interneuron differentiation	2	33.33333	0.049266	[Gli2, Gli3]
GO:0021520	spinal cord motor neuron cell fate specification	2	33.33333	0.049266	[Gli2, Gli3]
GO:0021521	ventral spinal cord interneuron specification	2	33.33333	0.049266	[Gli2, Gli3]
GO:0030903	notochord development	2	33.33333	0.049266	[Gli2, Nckap1]
GO:0032228	regulation of synaptic transmission, GABAergic	2	33.33333	0.049266	[Kras, Stxbp1]
GO:0033057	reproductive behavior in a multicellular organism	2	33.33333	0.049266	[Dbh, Grin1]
GO:0035058	sensory cilium biogenesis	2	33.33333	0.049266	[Bbs4, Vangl2]
GO:0040016	embryonic cleavage	2	33.33333	0.049266	[Ercc2, Top1]
GO:0043270	positive regulation of ion transport	2	33.33333	0.049266	[Atp2b2, Homer1]
GO:0045123	cellular extravasation	2	33.33333	0.049266	[Itga1, Itgam]
GO:0048041	focal adhesion formation	2	33.33333	0.049266	[Pxn, Sorbs1]
GO:0060043	regulation of cardiac muscle cell proliferation	2	33.33333	0.049266	[Fgfr1, Fgfr2]
GO_CellularComponent –up-regulated dataset					

GO:0031224	intrinsic to membrane	263	4.693077	3.87E-09	[Abca2, Abca8a, Abcb10, Abcb11, Abcb7, Abcc3, Abcg5, Abhd2, Ace, Acsl4, Adam26a, Adam3, Adam30, Adcy1, Adcy6, Adcyap1r1, Afg3l1, Agpat3, Alg5, Amn, Atp10b, Atp2a1, Atp2b1, Atp2b2, Atp2c1, Atp5g2, Atp6ap2, Atp8a1, Axl, B3gnt7, Bambi, Bcam, Btbd11, C2cd2l, C3ar1, Cacna1h, Cacnb1, Ccdc90a, Ccdc90b, Ccr8, Cd163, Cd33, Cdan1, Cdh20, Cdipt, Ceacam1, Ceacam2, Chst10, Chsy3, Clcn3, Cldn8, Clec1a, Cnih2, Cntn3, Comtd1, Cox7c, Creb3l2, Csf2ra, Csgalnact1, Dbh, Dhcr24, Dnajc16, Dpcr1, Dpep2, Dpp4, Dsg1a, Dsg1b, Dsg3, Dsg4, Ece2, Egfr, Epha7, Ephb6, Extl3, F11r, F830045P16Rik, Fcrl6, Fgfr1, Fgfr2, Fgfr4, Flot1, Fmo4, Folh1, Fras1, Fyco1, G6pc, Gabra1, Gabrd, Galnt3, Galnt5, Ggt5, Gimap1, Glt8d2, Gnptab, Golga5, Gosr1, Gp5, Gpc6, Gpr111, Gpr125, Gpr176, Gpr19, Gramd1b, Gria2, Grin1, Grm1, H2-D1, H47, Has2, Hrh4, Hs6st2, Ifitm7, Igsf1, Igsf8, Il1rl1, Il28ra, Ipo5, Itga1, Itgam, Jph3, Kcna1, Kcnh8, Kcnn1, Kcnq5, Kctd12, Kctd21, Kirrel3, Klra4, Klra7, Kpnb1, Lepr, Lifr, Lphn1, Lrrn1, Lrrtm3, Lsr, Ly75, Madd, Man2a1, Marco, Mctp2, Megf8, Mfap3, Mgat3, Mtx1, Naalad2, Nalcn, Nckap1, Ncln, Ncr1, Ndst2, Ndst3, Nlgn2, Notch2, Npc1l1, Nphs2, Ntng2, Ntsr1, Nup155, Nup210l, Ocln, Olfr1033, Olfr1211, Olfr1289, Olfr134, Olfr1495, Olfr181, Olfr632, Olfr642, Olfr695, Olfr780, Olfr806, Olfr867, Paqr8, Pcdh17, Pcdhb17, Pcnxl3, Pgrmc1, Pigr, Pkd1, Plxna1, Plxna4, Plxnb3, Plxnc1, Ppp1r3a, Ptgis, Ptprg, Ptprj, Ptprt, Ptprv, Pvrl2, Qrfpr, Qsox2, Rdh10, Reep2, Rhbdf2, Rhot2, Ror1, Rtn4, Rxfp1, Sacm1l, Scn7a, Sdk1, Sec22b, Sema6a, Siglecg, Skint5, Slc12a6, Slc16a7, Slc17a1, Slc17a8, Slc22a12, Slc25a2, Slc25a30, Slc25a35, Slc25a41, Slc27a3, Slc34a2, Slc35a3, Slc38a8, Slitrk2, Slitrk4, Slmap, Sltm, Sorcs1, Sptlc1, Srd5a1, St6galnac6, Stim1, Syt1, Syt2, Syt6, Tas1r2, Tas2r136, Tas2r143, Tbc1d9b, Tbxas1, Teddm1, Tm7sf2, Tm9sf3, Tmco7, Tmem120a, Tmem126a, Tmem173, Tmem201, Tmem70, Tmprss12, Tmtc3, Tnfrsf1a, Tnfsf4, Tomm20l, Tomm70a, Tpo, Treh, Trpc4, Trpm1, Unc5d, Ust, Vangl2, Vmn2r42, Vmn2r43, Vmn2r61, Vnn1]
GO:0005622	intracellular	638	7.310645	6.10E-09	[Aars, Aars2, Abca2, Abcb10, Abcb7, Abcc4, Abra, Acot12, Acot7, Acoxl, Acsf3, Acsl4, Actl7a, Actr1a, Add2, Adnp2, Aebp1, Afg3l1, Agbl1, Ahsa1, Aif1, Aire, Akap9, Alg5, Amacr, Amph, Ank1, Ankrd34b, Ap2s1, Apc2, Apon, Appbp2, Aptx, Araf, Arap2, Arhgap19, Arhgap22, Arhgef12, Arhgef3, Arhgef6, Arl3, Arl5b, Arpp19, Asap1, Asxl2, Asz1, Atoh8, Atp2a1, Atp2b2, Atp2c1, Atp5g2, Atp8a1, Avil, B3gnt7, Baz2a, Bbs4, Bcl2a1a, Becn1, Bhmt, Bnc1, Bnc2, Brip1, Btf3, Bub3, C330016O10Rik, Cacnb1, Capn11, Capn3, Cbr2, Cbr4, Cbx5, Ccdc50, Ccdc90a, Ccdc90b, Ccnb3, Ccno, Cct8, Cdipt, Cdk2, Cdk4, Cdkl1, Cebpz, Cenpb, Cenpj, Chd7, Chek1, Chid1, Chn2, Chst10, Chsy3, Cic, Cidec, Cit, Clcn3, Cog3, Cog6, Cops4, Cops7b, Coro1b, Cox6a1, Cox7c, Cps1, Cpsf2, Cpsf6, Cramp1l, Creb3l2, Csgalnact1, Ctnna1, Ctnna2, Ctr9, Cul1, Cul2, Cul5, Cux2, Cyfip2, Cyp1a1, Cyp1a2, Cyp27a1, Cyp2a5, Cyp2c38, Cyp2c39, Cyp2c54, Cyp2j5, Cyp4b1, Dapk1, Dazap1, Dbh, Dbil5, Dbnl, Ddhd2, Ddx21, Ddx27, Ddx3x, Ddx3y, Ddx50, Dgka, Dhcr24, Dicer1, Dis3, Dlg1, Dmrt1, Dnm2, Dpp7, Dpp8, Dtnb, Dtx2, Dync2h1, Dzip3, E2f8, Ece2, Echdc3, Efr3a, Egfr, Ehd1, Ehmt1, Eif2s1, Eif3l, Eif4a3, Eif4e, Eif4e2, Eif4g1, Eif6, Elmo3, Elmod1, Emid1, Eml1, Enah, Epc2, Eps8l2, Ercc2, Esco1, Esr2, Etaa1, Evi5, Exoc8,

Extl3, Fabp9, Faf2, Fam50a, Fam53a, Fancm, Fdx1, Fermt3, Fert2, Fga, Fgd5, Fgfr2, Fgg, Fhl2, Fip1l1, Fkbp2, Fmn2, Fmo4, Fmr1, Fn3k, Fnip2, Foxj3, Foxl2, Foxn3, Frmd4a, Frmd4b, Ftmt, Fxr2, G6pc, Gaa, Gabpb2, Galnt3, Galnt5, Gfer, Gfi1, Gimap1, Gimap8, Gli2, Gli3, Glis1, Gmfb, Gmfg, Gmip, Gnpda2, Gnptab, Golga5, Gosr1, Grhl2, Grin1, Grm1, Gtf2h1, Gvin1, Gzf1, H47, Hdgf, Hdgfrp3, Hdhd3, Hectd2, Hecw2, Hivep3, Hltf, Hnrnpa0, Hnrnpul2, Homer1, Homer3, Hook2, Hook3, Hoxb13, Hras1, Hsd17b11, Hsf1, Hspb1, Hsph1, Ick, Ide, Ik, Ikzf2, Ikzf4, Inpp5j, Ipo5, Isl2, Itgam, Itpa, Jak3, Jakmip1, Jph3, Kalrn, Khsrp, Kif12, Kif26a, Kif2c, Kif5b, Kif9, Kifc1, Klc1, Klc2, Klc4, Klhdc1, Kntc1, Kpnb1, Kras, Krt18, Krt19, Krt26, Krt32, Ksr2, Larp7, Lck, Lcp1, Lig1, Lig3, Lima1, Lin54, Lrdd, Lyn, Magi3, Man2a1, Man2b1, Map3k13, Map3k14, Map3k2, Mapk8ip3, Mbd3, Mcm2, Mcm3ap, Mcm7, Mecr, Med12l, Med15, Med27, Mefv, Meox2, Mgat3, Micalcl, Mkl2, Mkx, Mlh3, Mmp2, Morc1, Mpo, Mrpl2, Mrps26, Mrps27, Mrps34, Msh2, Mta1, Mta3, Mterfd1, Mtfr1, Mtx1, Mus81, Mutyh, Mvp, Mybl1, Mybpc3, Mycbp, Myef2, Myo15, Myo16, Myo1e, Myo3a, Myo7a, Mypn, Naga, Nags, Napa, Napb, Narg2, Ncapg2, Ncf2, Nckap1, Ncln, Ncoa2, Ndst2, Ndst3, Nedd4l, Nfkbid, Nfrkb, Nfx1, Ngef, Nipbl, Nisch, Nle1, Nlrp12, Nlrp9b, Nlrp9c, Nlrx1, Nol12, Nono, Nop56, Nosip, Notch2, Npat, Npepl1, Nr1h3, Nr2c2, Nr3c1, Nr3c2, Nsl1, Nt5c, Nt5c2, Nt5c3, Nub1, Nudc, Nuf2, Nup155, Optn, Orc3l, Otog, Pa2g4, Pacsin1, Pafah2, Pan3, Papolg, Parp12, Parp6, Parva, Pawr, Pck2, Pdcd11, Pdcd6ip, Pdia5, Pdk2, Pelp1, Per3, Pgr, Pgrmc1, Pgs1, Phf17, Phldb2, Phrf1, Phtf1, Pik3c3, Pitpnm3, Pkd1, Pkn2, Pla2g4d, Plce1, Plch2, Plcl1, Plekhh1, Plekhm1, Plod3, Pmf1, Pmpca, Polg, Polr2b, Pparg, Ppfibp1, Ppic, Ppil2, Ppp1ca, Ppp1cc, Ppp1r12a, Ppp1r13b, Ppp2r5c, Prdm5, Prdm6, Prickle1, Prkg2, Prmt1, Prpf6, Psip1, Psma1, Psmd1, Psmd14, Psmd6, Psmd8, Psme1, Psme2, Ptgis, Ptma, Purb, Purg, Pus1, Pwp2, Pxn, R3hdm2, Rab11fip5, Rab3gap2, Rab3ip, Rabgef1, Rai14, Rala, Ralb, Ranbp1, Rapgef5, Rars2, Rasgrf2, Rbm28, Rbm39, Rbm6, Rdh10, Relb, Retsat, Rexo1, Rfc5, Rfwd2, Rgnef, Rhbdf2, Rhot2, Ric8b, Rilpl1, Rint1, Ripk4, Rnaseh2b, Rnf114, Rnf123, Rnf17, Rnf6, Rnf7, Rngtt, Rpl28, Rpl34, Rpl3l, Rpp38, Rps6ka5, Rrs1, Rtel1, Rtn4, Ruvbl1, S100pbp, Sac3d1, Sacm1l, Sbds, Sbf1, Sbf2, Scel, Scin, Sdad1, Sec22b, Sec23ip, Senp6, Serpina3g, Setd5, Sf1, Sgsh, Sgsm1, Sgsm2, Sh3kbp1, Sh3pxd2a, Sh3rf1, Shroom3, Sim1, Sin3b, Sipa1l1, Slc17a8, Slc25a30, Slc25a35, Slc25a41, Slc27a3, Slc35a3, Slfn9, Slmap, Sltm, Smchd1, Smtn, Snrpa, Snrpb, Sod3, Sorbs1, Sox6, Sox7, Spag1, Spata4, Sptlc1, Srd5a1, Ssh2, St6galnac6, Stambp, Stat4, Stat5b, Stim1, Strbp, Stxbp1, Surf6, Svs2, Swap70, Syce1, Synpo2, Syt1, Syt2, Syt6, Taf1a, Tars2, Tarsl2, Tbc1d1, Tbc1d2b, Tbc1d9b, Tbca, Tcfe2a, Tcof1, Tec, Tekt4, Tesk2, Tfip11, Thrb, Tial1, Tjp1, Tm7sf2, Tmod1, Tmod3, Tom1, Tomm20l, Tomm70a, Top1, Top2b, Top3a, Tpo, Tpp1, Trappc10, Trdmt1, Trim16, Trim28, Trim29, Trim33, Trim37, Trim8, Trps1, Trrap, Tsfm, Tshz1, Ttc35, Ube3a, Ublcp1, Ubr1, Ubxn2b, Uchl3, Unc13a, Urod, Usp6nl, Ust, Utp20, Vprbp, Vps11, Vps18, Vps26b, Vps36, Wars, Wasf1, Wbp2nl, Whsc1l1, Wnk4, Wrn, Xrcc3, Yeats2, Yes1, Zbtb17, Zfp28, Zfp287, Zfp300, Zfp386, Zfp429, Zfp442, Zfp446, Zfp709, Zfp71-rs1, Zfy1, Zfy2, Zhx3, Zmym3, Zmym4, Zranb3]

GO:0016021	integral to membrane	259	4.724553	1.29E-08	[Abca2, Abca8a, Abcb10, Abcb11, Abcb7, Abcc3, Abcg5, Abhd2, Ace, Acsl4, Adam26a, Adam3, Adam30, Adcy1, Adcy6, Adcyap1r1, Afg3l1, Agpat3, Alg5, Amn, Atp10b, Atp2a1, Atp2b1, Atp2b2, Atp2c1, Atp5g2, Atp6ap2, Atp8a1, Axl, B3gnt7, Bambi, Bcam, Btbd11, C2cd2l, C3ar1, Cacna1h, Cacnb1, Ccdc90a, Ccdc90b, Ccr8, Cd163, Cd33, Cdan1, Cdh20, Cdipt, Ceacam1, Ceacam2, Chst10, Chsy3, Clcn3, Cldn8, Clec1a, Cnih2, Comtd1, Cox7c, Creb3l2, Csf2ra, Csgalnact1, Dbh, Dhcr24, Dnajc16, Dpcr1, Dpp4, Dsg1a, Dsg1b, Dsg3, Dsg4, Ece2, Egfr, Epha7, Ephb6, Extl3, F11r, F830045P16Rik, Fcrl6, Fgfr1, Fgfr2, Fgfr4, Flot1, Fmo4, Folh1, Fras1, Fyco1, G6pc, Gabra1, Gabrd, Galnt3, Galnt5, Ggt5, Gimap1, Glt8d2, Gnptab, Golga5, Gosr1, Gp5, Gpc6, Gpr111, Gpr125, Gpr176, Gpr19, Gramd1b, Gria2, Grin1, Grm1, H2-D1, H47, Has2, Hrh4, Hs6st2, Ifitm7, Igsf1, Igsf8, Il1rl1, Il28ra, Ipo5, Itga1, Itgam, Jph3, Kcna1, Kcnh8, Kcnn1, Kcnq5, Kctd12, Kctd21, Kirrel3, Klra4, Klra7, Kpnb1, Lepr, Lifr, Lphn1, Lrrn1, Lrrtm3, Lsr, Ly75, Madd, Man2a1, Marco, Mctp2, Megf8, Mfap3, Mgat3, Mtx1, Naalad2, Nalcn, Nckap1, Ncln, Ncr1, Ndst2, Ndst3, Nlgn2, Notch2, Npc1l1, Nphs2, Ntsr1, Nup155, Nup210l, Ocln, Olfr1033, Olfr1211, Olfr1289, Olfr134, Olfr1495, Olfr181, Olfr632, Olfr642, Olfr695, Olfr780, Olfr806, Olfr867, Paqr8, Pcdh17, Pcdhb17, Pcnxl3, Pgrmc1, Pigr, Pkd1, Plxna1, Plxna4, Plxnb3, Plxnc1, Ppp1r3a, Ptgis, Ptprg, Ptprj, Ptprt, Ptprv, Pvrl2, Qrfpr, Qsox2, Rdh10, Reep2, Rhbdf2, Rhot2, Ror1, Rtn4, Rxfp1, Sacm1l, Scn7a, Sdk1, Sec22b, Sema6a, Siglecg, Skint5, Slc12a6, Slc16a7, Slc17a1, Slc17a8, Slc22a12, Slc25a2, Slc25a30, Slc25a35, Slc25a41, Slc27a3, Slc34a2, Slc35a3, Slc38a8, Slitrk2, Slitrk4, Slmap, Sltm, Sorcs1, Sptlc1, Srd5a1, St6galnac6, Stim1, Syt1, Syt2, Syt6, Tas1r2, Tas2r136, Tas2r143, Tbc1d9b, Tbxas1, Teddm1, Tm7sf2, Tm9sf3, Tmco7, Tmem120a, Tmem126a, Tmem173, Tmem201, Tmem70, Tmprss12, Tmtc3, Tnfrsf1a, Tnfsf4, Tomm20l, Tomm70a, Tpo, Trpc4, Trpm1, Unc5d, Ust, Vangl2, Vmn2r42, Vmn2r43, Vmn2r61, Vnn1]
GO:0044424	intracellular part	616	7.279603	4.36E-08	[Aars, Aars2, Abca2, Abcb10, Abcb7, Abcc4, Abra, Acot12, Acot7, Acoxl, Acsf3, Acsl4, Actl7a, Actr1a, Add2, Adnp2, Aebp1, Afg3l1, Agbl1, Ahsa1, Aif1, Aire, Akap9, Alg5, Amacr, Amph, Ank1, Ankrd34b, Ap2s1, Apc2, Apon, Appbp2, Aptx, Araf, Arap2, Arhgap22, Arhgef12, Arhgef3, Arpp19, Asap1, Asxl2, Asz1, Atoh8, Atp2a1, Atp2b2, Atp2c1, Atp5g2, Atp8a1, Avil, B3gnt7, Baz2a, Bbs4, Bcl2a1a, Becn1, Bhmt, Bnc1, Bnc2, Brip1, Btf3, Bub3, C330016O10Rik, Cacnb1, Capn11, Capn3, Cbr2, Cbr4, Cbx5, Ccdc50, Ccdc90a, Ccdc90b, Ccnb3, Ccno, Cct8, Cdipt, Cdk2, Cdk4, Cdkl1, Cebpz, Cenpb, Cenpj, Chd7, Chek1, Chid1, Chst10, Chsy3, Cic, Cidec, Cit, Clcn3, Cog3, Cog6, Cops4, Cops7b, Coro1b, Cox6a1, Cox7c, Cps1, Cpsf2, Cpsf6, Cramp1l, Creb3l2, Csgalnact1, Ctnna1, Ctnna2, Ctr9, Cul1, Cul2, Cul5, Cux2, Cyfip2, Cyp1a1, Cyp1a2, Cyp27a1, Cyp2a5, Cyp2c38, Cyp2c39, Cyp2c54, Cyp2j5, Cyp4b1, Dapk1, Dazap1, Dbh, Dbil5, Dbnl, Ddhd2, Ddx21, Ddx27, Ddx3x, Ddx3y, Ddx50, Dgka, Dhcr24, Dis3, Dlg1, Dmrt1, Dnm2, Dpp7, Dpp8, Dtnb, Dtx2, Dync2h1, Dzip3, E2f8, Ece2, Echdc3, Efr3a, Egfr, Ehd1, Ehmt1, Eif2s1, Eif3l, Eif4a3, Eif4e, Eif4e2, Eif4g1, Eif6, Elmo3, Elmod1, Emid1, Eml1, Enah, Epc2, Eps8l2, Ercc2, Esco1, Esr2, Etaa1, Evi5, Exoc8, Extl3, Fabp9, Faf2, Fam50a, Fam53a, Fancm, Fdx1, Fermt3, Fert2, Fga, Fgd5, Fgfr2, Fgg, Fhl2, Fip1l1, Fkbp2, Fmn2, Fmo4, Fmr1, Fn3k, Fnip2, Foxj3,

					Foxl2, Foxn3, Frmd4a, Frmd4b, Ftmt, Fxr2, G6pc, Gaa, Gabpb2, Galnt3, Galnt5, Gfer, Gfi1, Gimap1, Gimap8, Gli2, Gli3, Glis1, Gnpda2, Gnptab, Golga5, Gosr1, Grhl2, Grin1, Grm1, Gtf2h1, Gvin1, Gzf1, H47, Hdgf, Hdgfrp3, Hdhd3, Hecw2, Hivep3, Hltf, Hnrnpa0, Hnrnpul2, Homer1, Homer3, Hook2, Hook3, Hoxb13, Hras1, Hsd17b11, Hsf1, Hspb1, Hsph1, Ick, Ide, Ik, Ikzf2, Ikzf4, Inpp5j, Ipo5, Isl2, Itgam, Itpa, Jak3, Jakmip1, Jph3, Kalrn, Khsrp, Kif12, Kif26a, Kif2c, Kif5b, Kif9, Kifc1, Klc1, Klc2, Klc4, Klhdc1, Kntc1, Kpnb1, Krt18, Krt19, Krt26, Krt32, Ksr2, Larp7, Lck, Lcp1, Lig1, Lig3, Lima1, Lin54, Lrdd, Lyn, Magi3, Man2a1, Man2b1, Map3k13, Map3k14, Map3k2, Mapk8ip3, Mbd3, Mcm2, Mcm3ap, Mcm7, Mecr, Med12l, Med15, Med27, Mefv, Meox2, Mgat3, Micalcl, Mkl2, Mkx, Mlh3, Mmp2, Morc1, Mpo, Mrpl2, Mrps26, Mrps27, Mrps34, Msh2, Mta1, Mta3, Mterfd1, Mtfr1, Mtx1, Mus81, Mutyh, Mvp, Mybl1, Mybpc3, Mycbp, Myef2, Myo15, Myo16, Myo1e, Myo3a, Myo7a, Mypn, Naga, Nags, Napa, Napb, Narg2, Ncapg2, Ncf2, Nckap1, Ncln, Ncoa2, Ndst2, Ndst3, Nedd4l, Nfkbid, Nfrkb, Nfx1, Ngef, Nipbl, Nisch, Nle1, Nlrp12, Nlrp9b, Nlrp9c, Nlrx1, Nol12, Nono, Nop56, Nosip, Notch2, Npat, Nr1h3, Nr2c2, Nr3c1, Nr3c2, Nsl1, Nt5c, Nt5c2, Nt5c3, Nub1, Nudc, Nuf2, Nup155, Optn, Orc3l, Otog, Pa2g4, Pacsin1, Pafah2, Pan3, Papolg, Parp12, Parp6, Parva, Pawr, Pck2, Pdcd11, Pdcd6ip, Pdia5, Pdk2, Pelp1, Per3, Pgr, Pgrmc1, Pgs1, Phf17, Phldb2, Phrf1, Phtf1, Pik3c3, Pkd1, Pkn2, Pla2g4d, Plce1, Plch2, Plcl1, Plekhh1, Plekhm1, Plod3, Pmf1, Pmpca, Polg, Polr2b, Pparg, Ppfibp1, Ppic, Ppil2, Ppp1ca, Ppp1cc, Ppp1r12a, Ppp1r13b, Ppp2r5c, Prdm5, Prdm6, Prickle1, Prkg2, Prmt1, Prpf6, Psip1, Psma1, Psmd1, Psmd14, Psmd6, Psmd8, Psme1, Psme2, Ptgis, Ptma, Purb, Purg, Pus1, Pwp2, Pxn, R3hdm2, Rab11fip5, Rab3gap2, Rab3ip, Rabgef1, Rai14, Ranbp1, Rapgef5, Rars2, Rasgrf2, Rbm28, Rbm39, Rbm6, Rdh10, Relb, Retsat, Rexo1, Rfc5, Rfwd2, Rgnef, Rhbdf2, Rhot2, Ric8b, Rilpl1, Rint1, Ripk4, Rnaseh2b, Rnf123, Rnf17, Rnf6, Rnf7, Rngtt, Rpl28, Rpl34, Rpl3l, Rpp38, Rps6ka5, Rrs1, Rtel1, Rtn4, Ruvbl1, S100pbp, Sac3d1, Sacm1l, Sbds, Sbf1, Sbf2, Scel, Scin, Sdad1, Sec22b, Sec23ip, Senp6, Serpina3g, Setd5, Sf1, Sgsh, Sgsm1, Sh3kbp1, Sh3pxd2a, Sh3rf1, Shroom3, Sim1, Sin3b, Slc17a8, Slc25a30, Slc25a35, Slc25a41, Slc27a3, Slc35a3, Slfn9, Slmap, Sltm, Smchd1, Smtn, Snrpa, Snrpb, Sod3, Sorbs1, Sox6, Sox7, Spag1, Spata4, Sptlc1, Srd5a1, Ssh2, St6galnac6, Stambp, Stat4, Stat5b, Stim1, Strbp, Stxbp1, Surf6, Svs2, Swap70, Syce1, Synpo2, Syt1, Syt2, Syt6, Taf1a, Tars2, Tarsl2, Tbc1d1, Tbca, Tcfe2a, Tcof1, Tec, Tekt4, Tesk2, Tfip11, Thrb, Tial1, Tjp1, Tm7sf2, Tmod1, Tmod3, Tom1, Tomm20l, Tomm70a, Top1, Top2b, Top3a, Tpo, Tpp1, Trappc10, Trdmt1, Trim16, Trim28, Trim29, Trim33, Trim37, Trim8, Trps1, Trrap, Tsfm, Tshz1, Ttc35, Ube3a, Ublcp1, Ubr1, Ubxn2b, Uchl3, Unc13a, Urod, Ust, Utp20, Vprbp, Vps11, Vps18, Vps26b, Vps36, Wars, Wasf1, Wbp2nl, Whsc1l1, Wnk4, Wrn, Xrcc3, Yeats2, Yes1, Zbtb17, Zfp28, Zfp287, Zfp300, Zfp386, Zfp429, Zfp442, Zfp446, Zfp709, Zfp71-rs1, Zfy1, Zfy2, Zhx3, Zmym3, Zmym4]
GO:0044425	membrane part	300	4.930966	1.35E-07	[Abca2, Abca8a, Abcb10, Abcb11, Abcb7, Abcc3, Abcg5, Abhd2, Ace, Acsl4, Adam26a, Adam3, Adam30, Adcy1, Adcy6, Adcyap1r1, Afg3l1, Agpat3, Aif1, Alg5, Amn, Amph, Ap2s1, Apc2, Atp10b, Atp2a1, Atp2b1, Atp2b2, Atp2c1, Atp5g2, Atp6ap2, Atp8a1, Axl, B2m, B3gnt7, Bambi, Bcam, Btbd11, C2cd2l,

					C3ar1, Cacna1h, Cacnb1, Ccdc90a, Ccdc90b, Ccr8, Cd163, Cd33, Cdan1, Cdh20, Cdipt, Ceacam1, Ceacam2, Chst10, Chsy3, Clcn3, Cldn8, Clec1a, Cnih2, Cntn3, Comtd1, Cox7c, Creb3l2, Csf2ra, Csgalnact1, Ctnna1, Ctnna2, Cyfip2, Dbh, Dhcr24, Dlg1, Dnajc16, Dnm2, Dpcr1, Dpep2, Dpp4, Dsg1a, Dsg1b, Dsg3, Dsg4, Ece2, Egfr, Enah, Epha7, Ephb6, Extl3, F11r, F830045P16Rik, Fcrl6, Fgfr1, Fgfr2, Fgfr4, Flot1, Fmo4, Folh1, Fras1, Frmd4b, Fyco1, G6pc, Gabra1, Gabrd, Galnt3, Galnt5, Ggt5, Gimap1, Glt8d2, Gna15, Gnptab, Golga5, Gosr1, Gp5, Gpc6, Gpr111, Gpr125, Gpr176, Gpr19, Gramd1b, Gria2, Grid2ip, Grin1, Grm1, H2-D1, H47, Has2, Homer1, Homer3, Hrh4, Hs6st2, Ifitm7, Igsf1, Igsf8, Il1rl1, Il28ra, Ipo5, Itga1, Itgam, Jph3, Kcna1, Kcnh8, Kcnn1, Kcnq5, Kctd12, Kctd21, Kirrel3, Klhl17, Klra4, Klra7, Kpnb1, Lcp1, Lepr, Lifr, Lphn1, Lrrn1, Lrrtm3, Lsr, Ly75, Madd, Magi3, Man2a1, Mapk8ip3, Marco, Mctp2, Megf8, Mfap3, Mgat3, Mpdz, Mtx1, Myo7a, Naalad2, Nalcn, Napa, Nckap1, Ncln, Ncr1, Ndst2, Ndst3, Nlgn2, Notch2, Npc1l1, Nphs2, Ntng2, Ntsr1, Nup155, Nup210l, Ocln, Olfr1033, Olfr1211, Olfr1289, Olfr134, Olfr1495, Olfr181, Olfr632, Olfr642, Olfr695, Olfr780, Olfr806, Olfr867, Paqr8, Parva, Pcdh17, Pcdhb17, Pcnxl3, Pgrmc1, Pigr, Pkd1, Plxna1, Plxna4, Plxnb3, Plxnc1, Ppp1r3a, Ptgis, Ptprg, Ptprj, Ptprt, Ptprv, Pvrl2, Pxn, Qrfpr, Qsox2, Rdh10, Reep2, Retsat, Rhbdf2, Rhot2, Ror1, Rtn4, Rxfp1, Sacm1l, Scn7a, Sdk1, Sec22b, Sema6a, Sh3kbp1, Shc4, Shroom3, Siglecg, Skint5, Slc12a6, Slc16a7, Slc17a1, Slc17a8, Slc22a12, Slc25a2, Slc25a30, Slc25a35, Slc25a41, Slc27a3, Slc34a2, Slc35a3, Slc38a8, Slitrk2, Slitrk4, Slmap, Sltm, Sorbs1, Sorcs1, Sptlc1, Srd5a1, Ssh2, St6galnac6, Stim1, Syt1, Syt2, Syt6, Tas1r2, Tas2r136, Tas2r143, Tbc1d9b, Tbxas1, Tcra, Teddm1, Tjp1, Tm7sf2, Tm9sf3, Tmco7, Tmem120a, Tmem126a, Tmem173, Tmem201, Tmem70, Tmprss12, Tmtc3, Tnfrsf1a, Tnfsf4, Tomm20l, Tomm70a, Tpo, Treh, Trpc4, Trpm1, Unc13a, Unc5d, Ust, Vangl2, Vmn2r42, Vmn2r43, Vmn2r61, Vnn1, Vps26b, Wnk4, Xirp1]
GO:0005737	cytoplasm	419	7.598839	7.89E-07	[Aars, Aars2, Abca2, Abcb10, Abcb7, Abcc4, Abra, Acot12, Acot7, Acoxl, Acsf3, Acsl4, Actl7a, Actr1a, Add2, Aebp1, Afg3l1, Agbl1, Ahsa1, Aif1, Aire, Akap9, Alg5, Amacr, Amph, Ank1, Ankrd34b, Ap2s1, Apc2, Apon, Appbp2, Araf, Arap2, Arhgap22, Arhgef12, Arhgef3, Arpp19, Asap1, Asz1, Atp2a1, Atp2b2, Atp2c1, Atp5g2, Atp8a1, Avil, B3gnt7, Bbs4, Bcl2a1a, Becn1, Bhmt, Bnc1, Brip1, C330016O10Rik, Cacnb1, Capn11, Capn3, Cbr2, Cbr4, Ccdc50, Ccdc90a, Ccdc90b, Cct8, Cdipt, Cdkl1, Cenpj, Chek1, Chid1, Chst10, Chsy3, Cidec, Cit, Clcn3, Cog3, Cog6, Cops4, Cops7b, Coro1b, Cox6a1, Cox7c, Cps1, Creb3l2, Csgalnact1, Ctnna1, Ctnna2, Cyfip2, Cyp1a1, Cyp1a2, Cyp27a1, Cyp2a5, Cyp2c38, Cyp2c39, Cyp2c54, Cyp2j5, Cyp4b1, Dapk1, Dazap1, Dbh, Dbil5, Dbnl, Ddhd2, Ddx3x, Ddx3y, Dgka, Dhcr24, Dis3, Dlg1, Dnm2, Dpp7, Dpp8, Dtnb, Dtx2, Dync2h1, Dzip3, Ece2, Echdc3, Egfr, Ehd1, Eif2s1, Eif4e, Eif4e2, Eif4g1, Eif6, Elmo3, Emid1, Eml1, Enah, Eps8l2, Esr2, Etaa1, Exoc8, Extl3, Fabp9, Faf2, Fdx1, Fermt3, Fert2, Fga, Fgd5, Fgg, Fhl2, Fkbp2, Fmo4, Fmr1, Fn3k, Fnip2, Frmd4a, Frmd4b, Ftmt, Fxr2, G6pc, Gaa, Galnt3, Galnt5, Gfer, Gimap1, Gimap8, Gli2, Gli3, Gnpda2, Gnptab, Golga5, Gosr1, Grin1, Gvin1, H47, Hdgf, Hdgfrp3, Hdhd3, Hecw2, Hivep3, Homer1, Homer3, Hook2, Hook3, Hras1, Hsd17b11, Hsf1, Hspb1, Hsph1, Ick, Ide, Inpp5j, Ipo5, Itpa, Jakmip1,

					Jph3, Kalrn, Khsrp, Kif2c, Kif5b, Kifc1, Klc1, Klc2, Klhdc1, Kntc1, Kpnb1, Krt19, Ksr2, Lck, Lcp1, Lima1, Lrdd, Lyn, Man2a1, Man2b1, Map3k13, Map3k14, Map3k2, Mapk8ip3, Mbd3, Mcm3ap, Mecr, Med15, Mefv, Mgat3, Micalcl, Mkl2, Mmp2, Mpo, Mrpl2, Mrps26, Mrps27, Mrps34, Mta1, Mta3, Mterfd1, Mtfr1, Mtx1, Mvp, Mybpc3, Mycbp, Myo15, Myo16, Myo3a, Myo7a, Mypn, Naga, Nags, Napa, Napb, Ncf2, Nckap1, Ncln, Ncoa2, Ndst2, Ndst3, Nedd4l, Ngef, Nisch, Nlrp12, Nlrp9b, Nlrp9c, Nlrx1, Nosip, Nr3c1, Nr3c2, Nt5c, Nt5c2, Nt5c3, Nudc, Optn, Otog, Pa2g4, Pacsin1, Pafah2, Pan3, Parva, Pawr, Pck2, Pdcd11, Pdcd6ip, Pdia5, Pdk2, Pelp1, Per3, Pgrmc1, Pgs1, Phf17, Phldb2, Pik3c3, Pkn2, Pla2g4d, Plce1, Plch2, Plcl1, Plekhm1, Plod3, Pmpca, Polg, Pparg, Ppic, Ppp1ca, Ppp1cc, Ppp1r12a, Ppp1r13b, Ppp2r5c, Prickle1, Psma1, Psmd1, Psmd14, Psmd6, Psmd8, Psme1, Psme2, Ptgis, Pxn, Rab11fip5, Rab3gap2, Rab3ip, Rabgef1, Rai14, Ranbp1, Rars2, Rasgrf2, Rdh10, Retsat, Rfwd2, Rgnef, Rhbdf2, Rhot2, Ric8b, Rilpl1, Rint1, Ripk4, Rnf123, Rnf17, Rnf6, Rnf7, Rpl28, Rpl34, Rpl3l, Rps6ka5, Rtn4, Sac3d1, Sacm1l, Sbds, Sbf2, Scel, Scin, Sec22b, Sec23ip, Serpina3g, Sf1, Sgsh, Sgsm1, Sh3kbp1, Sh3pxd2a, Sh3rf1, Shroom3, Sin3b, Slc17a8, Slc25a30, Slc25a35, Slc25a41, Slc27a3, Slc35a3, Slmap, Smtn, Snrpb, Sod3, Sorbs1, Spag1, Spata4, Sptlc1, Srd5a1, Ssh2, St6galnac6, Stambp, Stat4, Stat5b, Stim1, Strbp, Stxbp1, Svs2, Swap70, Synpo2, Syt1, Syt2, Syt6, Tars2, Tarsl2, Tbca, Tcfe2a, Tec, Tfip11, Tial1, Tjp1, Tm7sf2, Tmod1, Tmod3, Tom1, Tomm20l, Tomm70a, Top1, Tpo, Tpp1, Trappc10, Trim16, Trim29, Trim37, Tsfm, Ube3a, Ubr1, Ubxn2b, Uchl3, Unc13a, Urod, Ust, Vprbp, Vps11, Vps18, Vps26b, Vps36, Wars, Wasf1, Wbp2nl, Wnk4, Yes1, Zhx3]
GO:0031252	cell leading edge	16	20.25316	2.43E-05	[Aif1, Cit, Ctnna1, Ctnna2, Dbnl, Enah, Eps8l2, Inpp5j, Lcp1, Mapk8ip3, Nckap1, Parva, Pxn, Sh3rf1, Tmod3, Wasf1]
GO:0005856	cytoskeleton	84	9.655172	6.93E-05	[Abra, Actl7a, Actr1a, Add2, Aif1, Akap9, Amph, Ank1, Apc2, Appbp2, Avil, Bbs4, Becn1, Cenpj, Cit, Coro1b, Ctnna1, Ctnna2, Dbnl, Dlg1, Dnm2, Dync2h1, Efr3a, Eif6, Elmo3, Elmod1, Eml1, Enah, Fermt3, Fgd5, Fmn2, Frmd4a, Frmd4b, Grin1, Grm1, Hook2, Hook3, Jak3, Jakmip1, Kalrn, Kif12, Kif26a, Kif2c, Kif5b, Kif9, Kifc1, Klc1, Klc2, Klc4, Krt18, Krt19, Krt26, Krt32, Lcp1, Lima1, Mefv, Mybpc3, Myo15, Myo16, Myo1e, Myo3a, Myo7a, Nudc, Parva, Plekhh1, Pxn, Rai14, Ranbp1, Sac3d1, Scin, Sh3kbp1, Shroom3, Smtn, Sorbs1, Ssh2, Strbp, Tbca, Tekt4, Tmod1, Tmod3, Vps11, Vps18, Wasf1, Wbp2nl]
GO:0043229	intracellular organelle	502	7.076403	1.84E-04	[Aars2, Abca2, Abcb10, Abcb7, Abcc4, Abra, Acoxl, Acsf3, Acsl4, Actl7a, Actr1a, Add2, Adnp2, Aebp1, Afg3l1, Ahsa1, Aif1, Aire, Akap9, Alg5, Amacr, Amph, Ank1, Ankrd34b, Ap2s1, Apc2, Appbp2, Aptx, Araf, Arhgap22, Asxl2, Atoh8, Atp2a1, Atp2b2, Atp2c1, Atp5g2, Atp8a1, Avil, B3gnt7, Baz2a, Bbs4, Becn1, Bnc1, Bnc2, Brip1, Btf3, Bub3, C330016O10Rik, Cacnb1, Capn11, Cbr2, Cbx5, Ccdc90a, Ccdc90b, Ccnb3, Ccno, Cdipt, Cdk2, Cdk4, Cdkl1, Cebpz, Cenpb, Cenpj, Chd7, Chek1, Chid1, Chst10, Chsy3, Cic, Cit, Clcn3, Cog3, Cog6, Cops4, Cops7b, Coro1b, Cox6a1, Cox7c, Cps1, Cpsf2, Cpsf6, Cramp1l, Creb3l2, Csgalnact1, Ctnna1, Ctnna2, Ctr9, Cux2, Cyp1a1, Cyp1a2, Cyp27a1, Cyp2a5, Cyp2c38, Cyp2c39, Cyp2c54, Cyp2j5, Cyp4b1, Dazap1, Dbh, Dbnl, Ddx21, Ddx27, Ddx3x, Ddx3y, Ddx50, Dhcr24, Dis3, Dlg1, Dmrt1, Dnm2, Dpp7, Dtx2, Dync2h1, E2f8, Ece2, Echdc3, Efr3a, Egfr, Ehd1, Ehmt1, Eif2s1, Eif3l, Eif4a3,

					Eif6, Elmo3, Elmod1, Emid1, Eml1, Enah, Epc2, Ercc2, Esco1, Esr2, Evi5, Extl3, Fam50a, Fam53a, Fancm, Fdx1, Fermt3, Fert2, Fgd5, Fgfr2, Fhl2, Fip1l1, Fkbp2, Fmn2, Fmo4, Fmr1, Foxj3, Foxl2, Foxn3, Frmd4a, Frmd4b, Ftmt, G6pc, Gaa, Gabpb2, Galnt3, Galnt5, Gfer, Gfi1, Gimap1, Gimap8, Gli2, Gli3, Glis1, Gnptab, Golga5, Gosr1, Grhl2, Grin1, Grm1, Gtf2h1, Gvin1, Gzf1, H47, Hdgf, Hdgfrp3, Hdhd3, Hivep3, Hltf, Hnrnpul2, Hook2, Hook3, Hoxb13, Hras1, Hsf1, Hspb1, Hsph1, Ik, Ikzf2, Ikzf4, Ipo5, Isl2, Itgam, Jak3, Jakmip1, Jph3, Kalrn, Khsrp, Kif12, Kif26a, Kif2c, Kif5b, Kif9, Kifc1, Klc1, Klc2, Klc4, Kntc1, Kpnb1, Krt18, Krt19, Krt26, Krt32, Larp7, Lcp1, Lig1, Lig3, Lima1, Lin54, Lrdd, Lyn, Magi3, Man2a1, Man2b1, Map3k2, Mapk8ip3, Mbd3, Mcm2, Mcm3ap, Mcm7, Mecr, Med12l, Med15, Med27, Mefv, Meox2, Mgat3, Mkl2, Mkx, Mlh3, Morc1, Mpo, Mrpl2, Mrps26, Mrps27, Mrps34, Msh2, Mta1, Mta3, Mterfd1, Mtfr1, Mtx1, Mus81, Mutyh, Mybl1, Mybpc3, Mycbp, Myef2, Myo15, Myo16, Myo1e, Myo3a, Myo7a, Mypn, Naga, Nags, Napa, Napb, Narg2, Ncapg2, Ncf2, Ncln, Ncoa2, Ndst2, Ndst3, Nfkbid, Nfrkb, Nfx1, Nipbl, Nisch, Nle1, Nlrp12, Nol12, Nono, Nop56, Nosip, Notch2, Npat, Nr1h3, Nr2c2, Nr3c1, Nr3c2, Nsl1, Nub1, Nudc, Nuf2, Nup155, Optn, Orc3l, Pa2g4, Pacsin1, Papolg, Parp12, Parp6, Parva, Pawr, Pck2, Pdia5, Pdk2, Pelp1, Per3, Pgr, Pgrmc1, Pgs1, Phf17, Phrf1, Phtf1, Pkd1, Pkn2, Pla2g4d, Plce1, Plekhh1, Plod3, Pmf1, Pmpca, Polg, Polr2b, Pparg, Ppfibp1, Ppil2, Ppp1ca, Ppp1cc, Ppp1r13b, Ppp2r5c, Prdm5, Prdm6, Prickle1, Prmt1, Prpf6, Psip1, Psma1, Ptgis, Ptma, Purb, Purg, Pus1, Pwp2, Pxn, R3hdm2, Rab11fip5, Rab3ip, Rabgef1, Rai14, Ranbp1, Rapgef5, Rars2, Rasgrf2, Rbm28, Rbm39, Rbm6, Rdh10, Relb, Retsat, Rexo1, Rfc5, Rfwd2, Rhbdf2, Rhot2, Rint1, Rnaseh2b, Rnf17, Rnf6, Rnf7, Rngtt, Rpl28, Rpl34, Rpl3l, Rpp38, Rps6ka5, Rrs1, Rtel1, Rtn4, Ruvbl1, S100pbp, Sac3d1, Sacm1l, Sbf1, Sbf2, Scin, Sdad1, Sec22b, Sec23ip, Senp6, Serpina3g, Setd5, Sf1, Sgsh, Sgsm1, Sh3kbp1, Sh3rf1, Shroom3, Sim1, Sin3b, Slc17a8, Slc25a30, Slc25a35, Slc25a41, Slc27a3, Slc35a3, Slfn9, Sltm, Smchd1, Smtn, Snrpa, Snrpb, Sod3, Sorbs1, Sox6, Sox7, Sptlc1, Srd5a1, Ssh2, St6galnac6, Stambp, Stat4, Stat5b, Stim1, Strbp, Stxbp1, Surf6, Svs2, Swap70, Syce1, Synpo2, Syt1, Syt2, Syt6, Taf1a, Tars2, Tbc1d1, Tbca, Tcfe2a, Tcof1, Tekt4, Tesk2, Tfip11, Thrb, Tial1, Tjp1, Tm7sf2, Tmod1, Tmod3, Tomm20l, Tomm70a, Top1, Top2b, Top3a, Tpo, Tpp1, Trappc10, Trdmt1, Trim28, Trim33, Trim37, Trim8, Trps1, Trrap, Tsfm, Tshz1, Ttc35, Ube3a, Ublcp1, Ubxn2b, Ust, Utp20, Vps11, Vps18, Vps36, Wasf1, Wbp2nl, Whsc1l1, Wrn, Xrcc3, Yeats2, Zbtb17, Zfp28, Zfp287, Zfp300, Zfp386, Zfp429, Zfp442, Zfp446, Zfp709, Zfp71-rs1, Zfy1, Zfy2, Zhx3, Zmym3, Zmym4]
GO:0016020	membrane	387	5.459162	4.88E-04	[Abca2, Abca8a, Abcb10, Abcb11, Abcb7, Abcc3, Abcc4, Abcg5, Abhd2, Ace, Acsl4, Adam26a, Adam3, Adam30, Adcy1, Adcy6, Adcyap1r1, Add2, Adrbk1, Afg3l1, Agpat3, Aif1, Alg5, Amn, Amph, Ank1, Ap2s1, Apc2, Appbp2, Arhgef12, Asap1, Atp10b, Atp2a1, Atp2b1, Atp2b2, Atp2c1, Atp5g2, Atp6ap2, Atp8a1, Axl, B2m, B3gnt7, Bambi, Bcam, Becn1, Btbd11, C2cd2l, C330016O10Rik, C3ar1, Cacna1h, Cacnb1, Ccdc90a, Ccdc90b, Ccr8, Cd163, Cd33, Cdan1, Cdh20, Cdipt, Ceacam1, Ceacam2, Chn2, Chst10, Chsy3, Clcn3, Cldn8, Clec1a, Cnih2, Cntn3, Cog3, Cog6, Comtd1, Cox6a1, Cox7c, Creb3l2, Csf2ra, Csgalnact1,

					Ctnna1, Ctnna2, Cyfip2, Cyp1a1, Cyp1a2, Cyp27a1, Cyp2a5, Cyp2c38, Cyp2c39, Cyp2c54, Cyp2j5, Cyp4b1, Dbh, Dgka, Dhcr24, Dlg1, Dlgap4, Dnaja1, Dnaja2, Dnaja4, Dnajc16, Dnm2, Dpcr1, Dpep2, Dpp4, Dpp8, Dsg1a, Dsg1b, Dsg3, Dsg4, Dync2h1, Ece2, Efr3a, Egfr, Ehd1, Eif6, Enah, Epha7, Ephb6, Extl3, F11r, F830045P16Rik, Fcrl6, Fgfr1, Fgfr2, Fgfr4, Fkbp2, Flot1, Fmo4, Folh1, Fras1, Frmd4b, Fyco1, Fyn, G6pc, Gabra1, Gabrd, Galnt3, Galnt5, Ggt5, Gimap1, Glt8d2, Gna15, Gnptab, Golga5, Gosr1, Gp5, Gpc6, Gpr111, Gpr125, Gpr176, Gpr19, Gramd1b, Gria2, Grid2ip, Grin1, Grm1, H2-D1, H47, Has2, Hck, Homer1, Homer3, Hras1, Hrh4, Hs6st2, Hspb1, Ifitm7, Igsf1, Igsf8, Il1rl1, Il28ra, Ipo5, Itga1, Itgam, Jak3, Jakmip1, Jph3, Kcna1, Kcnh8, Kcnip1, Kcnn1, Kcnq5, Kctd12, Kctd21, Kirrel3, Klhl17, Klra13, Klra18, Klra4, Klra7, Kpnb1, Kras, Krt19, Ksr2, Lck, Lcp1, Lepr, Lifr, Lphn1, Lrrn1, Lrrtm3, Lsr, Ly75, Madd, Magi3, Man2a1, Map3k13, Mapk8ip3, Marco, Mctp2, Megf8, Mfap3, Mgat3, Mmp2, Mpdz, Mtx1, Myo7a, Naalad2, Nalcn, Napa, Napb, Nckap1, Ncln, Ncr1, Ndst2, Ndst3, Neu3, Ngef, Nisch, Nlgn2, Notch2, Npc1l1, Nphs2, Nr3c1, Nr3c2, Ntng2, Ntsr1, Nup155, Nup210l, Ocln, Olfr1033, Olfr1211, Olfr1289, Olfr134, Olfr1495, Olfr181, Olfr632, Olfr642, Olfr695, Olfr780, Olfr806, Olfr867, Otog, Paqr8, Parva, Pcdh17, Pcdhb17, Pcnxl3, Pgrmc1, Phldb2, Pigr, Pitpnm3, Pkd1, Pla2g4d, Plce1, Plch2, Plod3, Plxna1, Plxna4, Plxnb3, Plxnc1, Polg, Ppp1cc, Ppp1r16b, Ppp1r3a, Prickle1, Prmt8, Ptgis, Ptprg, Ptprj, Ptprt, Ptprv, Pvrl2, Pxn, Qrfpr, Qsox2, Rab11fip5, Rala, Ralb, Rasgrf2, Rdh10, Reep2, Ren1, Retsat, Rftn1, Rgnef, Rhbdf2, Rhot2, Rint1, Ripk4, Ror1, Rtn4, Rxfp1, Sacm1l, Sbf2, Scel, Scn7a, Sdk1, Sec22b, Sec23ip, Sema3b, Sema6a, Sh3kbp1, Shc4, Shroom3, Siglecg, Skint5, Slc12a6, Slc16a7, Slc17a1, Slc17a8, Slc22a12, Slc25a2, Slc25a30, Slc25a35, Slc25a41, Slc27a3, Slc34a2, Slc35a3, Slc38a8, Slitrk2, Slitrk4, Slmap, Sltm, Sorbs1, Sorcs1, Sptlc1, Srd5a1, Ssh2, St6galnac6, Stambp, Stim1, Swap70, Syt1, Syt2, Syt6, Tas1r2, Tas2r136, Tas2r143, Tbc1d9b, Tbxas1, Tcra, Teddm1, Tjp1, Tm7sf2, Tm9sf3, Tmco7, Tmem120a, Tmem126a, Tmem173, Tmem201, Tmem70, Tmprss12, Tmprss7, Tmtc3, Tnfrsf1a, Tnfsf4, Tom1, Tomm20l, Tomm70a, Tpo, Treh, Trpc4, Trpm1, Unc13a, Unc5d, Ust, Vangl2, Veph1, Vmn2r42, Vmn2r43, Vmn2r61, Vnn1, Vps11, Vps18, Vps26b, Wasf1, Wnk4, Xirp1]
GO:0005871	kinesin complex	5	41.66667	5.12E-04	[Kif5b, Kifc1, Klc1, Klc2, Klc4]
GO:0030027	lamellipodium	10	20.40816	7.54E-04	[Ctnna1, Ctnna2, Dbnl, Enah, Nckap1, Parva, Pxn, Sh3rf1, Tmod3, Wasf1]
GO:0005913	cell-cell adherens junction	7	25.92593	0.001059	[Ctnna1, Ctnna2, Dlg1, Dsg3, Sorbs1, Tjp1, Xirp1]
GO:0005886	plasma membrane	170	5.062537	0.001517	[Abca8a, Abcb11, Abcc3, Abcc4, Ace, Adcy1, Adcy6, Adcyap1r1, Add2, Aif1, Amph, Ap2s1, Apc2, Atp2b1, Atp2b2, B2m, Bcam, C3ar1, Cacna1h, Cacnb1, Ccr8, Cd163, Cd33, Cdh20, Cdipt, Cldn8, Cntn3, Ctnna1, Ctnna2, Cyfip2, Dgka, Dlg1, Dnm2, Dpp4, Dsg1a, Dsg1b, Dsg3, Dsg4, Dync2h1, Efr3a, Egfr, Ehd1, Enah, Ephb6, F11r, Flot1, Folh1, Fras1, Fyn, Gabra1, Gabrd, Ggt5, Gna15, Gpc6, Gpr111, Gpr125, Gpr176, Gpr19, Gria2, Grid2ip, Grin1, Grm1, H2-D1, Homer1, Homer3, Hras1, Hrh4, Hspb1, Il1rl1, Itga1, Itgam, Jph3, Kcna1, Kcnip1, Kcnq5, Kctd12, Kctd21, Kirrel3, Klhl17, Klra13, Klra18, Klra4, Klra7, Kras, Krt19,

					Lck, Lcp1, Lepr, Lifr, Lphn1, Lsr, Ly75, Magi3, Mapk8ip3, Mfap3, Mmp2, Mpdz, Myo7a, Nckap1, Ncr1, Neu3, Nisch, Notch2, Npc1l1, Nphs2, Ntng2, Ntsr1, Ocln, Olfr1211, Olfr1289, Olfr1495, Olfr181, Olfr632, Olfr642, Olfr695, Olfr806, Olfr867, Otog, Parva, Pcdh17, Pigr, Pkd1, Plce1, Plch2, Plxna1, Plxna4, Ppp1r16b, Prmt8, Pvrl2, Pxn, Qrfpr, Rala, Ralb, Rasgrf2, Rftn1, Rgnef, Ror1, Rxfp1, Sh3kbp1, Shc4, Shroom3, Slc12a6, Slc16a7, Slc17a8, Slc22a12, Slc34a2, Slmap, Sltm, Sorbs1, Ssh2, Stim1, Swap70, Syt1, Syt2, Syt6, Tas1r2, Tcra, Tjp1, Tnfrsf1a, Treh, Trpc4, Trpm1, Unc13a, Vangl2, Veph1, Vmn2r42, Vmn2r43, Vnn1, Wnk4, Xirp1]
GO:0005912	adherens junction	11	17.46032	0.001628	[Ctnna1, Ctnna2, Dlg1, Dsg3, Enah, Parva, Pxn, Shroom3, Sorbs1, Tjp1, Xirp1]
GO:0005911	cell-cell junction	20	13.33333	0.001727	[Abcb11, Cldn8, Ctnna1, Ctnna2, Dlg1, Dpp4, Dsg1a, Dsg1b, Dsg3, Dsg4, F11r, Magi3, Mpdz, Ocln, Pvrl2, Shroom3, Sorbs1, Tjp1, Wnk4, Xirp1]
GO:0042995	cell projection	44	10.02278	0.001824	[Actl7a, Aif1, Atp2b2, Cit, Ctnna1, Ctnna2, Dbnl, Dock7, Dync2h1, Enah, Eps8l2, Exoc8, Grin1, Grm1, Ift172, Inpp5j, Kif5b, Klc1, Klc2, Lcp1, Mapk8ip3, Mpdz, Myo15, Myo7a, Nckap1, Ngef, Nphp4, Parva, Pkd1, Pxn, Pygb, Rnf6, Rtn4, Sema6a, Sh3pxd2a, Sh3rf1, Slc12a6, Slc22a12, Swap70, Syt1, Tekt4, Tmod3, Treh, Wasf1]
GO:0000502	proteasome complex	7	23.33333	0.002062	[Psma1, Psmd1, Psmd14, Psmd8, Psme1, Psme2, Ubr1]
GO:0022624	proteasome accessory complex	3	60	0.002199	[Psmd8, Psme1, Psme2]
GO:0043232	intracellular non-membrane-bounded organelle	120	8.064516	0.00352	[Abra, Actl7a, Actr1a, Add2, Aif1, Akap9, Amph, Ank1, Apc2, Appbp2, Avil, Bbs4, Becn1, Cbx5, Cdk2, Cenpb, Cenpj, Chd7, Chek1, Cit, Coro1b, Ctnna1, Ctnna2, Dbnl, Ddx21, Dlg1, Dnm2, Dync2h1, Efr3a, Eif3l, Eif6, Elmo3, Elmod1, Eml1, Enah, Fermt3, Fgd5, Fmn2, Frmd4a, Frmd4b, Grin1, Grm1, Hook2, Hook3, Jak3, Jakmip1, Kalrn, Kif12, Kif26a, Kif2c, Kif5b, Kif9, Kifc1, Klc1, Klc2, Klc4, Krt18, Krt19, Krt26, Krt32, Lcp1, Lima1, Lin54, Mbd3, Mcm2, Mefv, Mlh3, Mrpl2, Mrps26, Mrps27, Mrps34, Mybpc3, Myo15, Myo16, Myo1e, Myo3a, Myo7a, Nol12, Nudc, Nuf2, Orc3l, Parva, Plekhh1, Purb, Pus1, Pxn, Rai14, Ranbp1, Rfc5, Rpl28, Rpl34, Rpl3l, Rps6ka5, Sac3d1, Scin, Sh3kbp1, Shroom3, Sin3b, Smchd1, Smtn, Sorbs1, Ssh2, Strbp, Surf6, Syce1, Taf1a, Tbca, Tcof1, Tekt4, Tmod1, Tmod3, Top1, Top2b, Top3a, Trim28, Vps11, Vps18, Wasf1, Wbp2nl, Wrn]
GO:0042598	vesicular fraction	19	12.4183	0.003725	[Acsl4, Atp2b2, Cyp1a1, Cyp1a2, Cyp2a5, Cyp2c38, Cyp2c39, Cyp2c54, Cyp2j5, Cyp4b1, Fmo4, G6pc, Grm1, Jph3, Pgrmc1, Rdh10, Srd5a1, Syt2, Tbxas1]
GO:0043296	apical junction complex	13	14.44445	0.00375	[Cldn8, Ctnna1, Dsg1a, Dsg1b, Dsg3, Dsg4, F11r, Magi3, Mpdz, Ocln, Shroom3, Tjp1, Wnk4]
GO:0046581	intercellular canaliculus	3	50	0.004194	[Abcb11, Dpp4, Tjp1]
GO:0001726	ruffle	7	20.58824	0.004383	[Aif1, Cit, Dbnl, Eps8l2, Inpp5j, Lcp1, Tmod3]
GO:0030054	cell junction	42	9.767442	0.004402	[Abcb11, Amph, Cldn8, Ctnna1, Ctnna2, Cyfip2, Dlg1, Dnm2, Dpp4, Dsg1a, Dsg1b, Dsg3, Dsg4, Enah, F11r, Gabra1, Gabrd, Gria2, Grid2ip, Grin1, Homer1, Homer3, Klhl17, Magi3, Mpdz, Ocln, Parva, Pvrl2, Pxn, Sh3kbp1, Shc4, Shroom3, Slc17a8, Sorbs1, Ssh2, Syt1, Syt2, Syt6, Tjp1, Unc13a, Wnk4, Xirp1]
GO:0016327	apicolateral plasma	13	14.13044	0.007176	[Cldn8, Ctnna1, Dsg1a, Dsg1b, Dsg3, Dsg4, F11r, Magi3, Mpdz, Ocln, Shroom3,

	membrane				Tjp1, Wnk4]
GO:0005874	microtubule	23	11.16505	0.008129	[Apc2, Appbp2, Cenpj, Dnm2, Dync2h1, Eml1, Hook2, Hook3, Jakmip1, Kif12, Kif26a, Kif2c, Kif5b, Kif9, Kifc1, Klc1, Klc2, Klc4, Mefv, Nudc, Shroom3, Tbca, Tekt4]
GO:0005884	actin filament	5	23.80953	0.008219	[Aif1, Lcp1, Tmod3, Vps11, Vps18]
GO:0030057	desmosome	4	28.57143	0.009093	[Dsg1a, Dsg1b, Dsg3, Dsg4]
GO:0005792	microsome	18	12	0.009491	[Acsl4, Atp2b2, Cyp1a1, Cyp1a2, Cyp2a5, Cyp2c38, Cyp2c39, Cyp2c54, Cyp2j5, Cyp4b1, Fmo4, G6pc, Grm1, Jph3, Pgrmc1, Rdh10, Srd5a1, Tbxas1]
GO:0015629	actin cytoskeleton	20	11.42857	0.010613	[Abra, Aif1, Cit, Ctnna1, Ctnna2, Enah, Fermt3, Fmn2, Lcp1, Lima1, Mybpc3, Myo15, Myo16, Myo1e, Myo3a, Myo7a, Sorbs1, Tmod3, Vps11, Vps18]
GO:0000806	Y chromosome	2	66.66666	0.011174	[Cdk2, Sin3b]
GO:0001891	phagocytic cup	2	66.66666	0.011174	[Aif1, Lcp1]
GO:0008537	proteasome activator complex	2	66.66666	0.011174	[Psme1, Psme2]
GO:0044430	cytoskeletal part	49	8.990826	0.011359	[Aif1, Akap9, Apc2, Appbp2, Bbs4, Cenpj, Dlg1, Dnm2, Dync2h1, Eif6, Eml1, Enah, Fermt3, Grin1, Grm1, Hook2, Hook3, Jakmip1, Kif12, Kif26a, Kif2c, Kif5b, Kif9, Kifc1, Klc1, Klc2, Klc4, Krt18, Krt19, Krt26, Krt32, Lcp1, Mefv, Mybpc3, Myo15, Myo16, Myo1e, Myo3a, Myo7a, Nudc, Ranbp1, Sac3d1, Shroom3, Sorbs1, Tbca, Tekt4, Tmod3, Vps11, Vps18]
GO:0043231	intracellular membrane-bounded organelle	429	6.861804	0.012343	[Aars2, Abca2, Abcb10, Abcb7, Abcc4, Acoxl, Acsf3, Acsl4, Actl7a, Adnp2, Aebp1, Afg3l1, Ahsa1, Aire, Alg5, Amacr, Amph, Ank1, Ankrd34b, Ap2s1, Apc2, Appbp2, Aptx, Araf, Arhgap22, Asxl2, Atoh8, Atp2a1, Atp2b2, Atp2c1, Atp5g2, B3gnt7, Baz2a, Becn1, Bnc1, Bnc2, Brip1, Btf3, Bub3, Cacnb1, Capn11, Cbr2, Cbx5, Ccdc90a, Ccdc90b, Ccnb3, Ccno, Cdipt, Cdk2, Cdk4, Cdkl1, Cebpz, Cenpb, Chd7, Chek1, Chid1, Chst10, Chsy3, Cic, Clcn3, Cog3, Cog6, Cops4, Cops7b, Cox6a1, Cox7c, Cps1, Cpsf2, Cpsf6, Cramp1l, Creb3l2, Csgalnact1, Ctr9, Cux2, Cyp1a1, Cyp1a2, Cyp27a1, Cyp2a5, Cyp2c38, Cyp2c39, Cyp2c54, Cyp2j5, Cyp4b1, Dazap1, Ddx21, Ddx27, Ddx3x, Ddx3y, Ddx50, Dhcr24, Dis3, Dlg1, Dmrt1, Dpp7, Dtx2, E2f8, Ece2, Echdc3, Egfr, Ehd1, Ehmt1, Eif2s1, Eif3l, Eif4a3, Eif6, Emid1, Epc2, Ercc2, Esco1, Esr2, Evi5, Extl3, Fam50a, Fam53a, Fancm, Fdx1, Fert2, Fgfr2, Fhl2, Fip1l1, Fkbp2, Fmo4, Fmr1, Foxj3, Foxl2, Foxn3, Ftmt, G6pc, Gaa, Gabpb2, Galnt3, Galnt5, Gfer, Gfi1, Gimap1, Gimap8, Gli2, Gli3, Glis1, Gnptab, Golga5, Gosr1, Grhl2, Grin1, Grm1, Gtf2h1, Gvin1, Gzf1, H47, Hdgf, Hdgfrp3, Hdhd3, Hivep3, Hltf, Hnrnpul2, Hook3, Hoxb13, Hras1, Hsf1, Hspb1, Hsph1, Ik, Ikzf2, Ikzf4, Ipo5, Isl2, Itgam, Jph3, Khsrp, Kif2c, Kifc1, Kntc1, Kpnb1, Larp7, Lig1, Lig3, Lin54, Lrdd, Lyn, Magi3, Man2a1, Man2b1, Map3k2, Mapk8ip3, Mbd3, Mcm2, Mcm3ap, Mcm7, Mecr, Med12l, Med15, Med27, Meox2, Mgat3, Mkl2, Mkx, Mlh3, Morc1, Mpo, Mrpl2, Mrps26, Mrps27, Mrps34, Msh2, Mta1, Mta3, Mterfd1, Mtfr1, Mtx1, Mus81, Mutyh, Mybl1, Mycbp, Myef2, Myo7a, Mypn, Naga, Nags, Napa, Napb, Narg2, Ncapg2, Ncf2, Ncln, Ncoa2, Ndst2, Ndst3, Nfkbid, Nfrkb, Nfx1, Nipbl, Nisch, Nle1, Nlrp12, Nol12, Nono, Nop56, Nosip, Notch2, Npat, Nr1h3, Nr2c2, Nr3c1, Nr3c2, Nsl1, Nub1, Nudc, Nuf2, Nup155, Optn, Orc3l, Pa2g4, Pacsin1, Papolg, Parp12,

					Parp6, Parva, Pawr, Pck2, Pdia5, Pdk2, Pelp1, Per3, Pgr, Pgrmc1, Pgs1, Phf17, Phrf1, Phtf1, Pkd1, Pkn2, Plce1, Plod3, Pmf1, Pmpca, Polg, Polr2b, Pparg, Ppfibp1, Ppil2, Ppp1ca, Ppp1cc, Ppp1r13b, Ppp2r5c, Prdm5, Prdm6, Prickle1, Prmt1, Prpf6, Psip1, Psma1, Ptgis, Ptma, Purb, Purg, Pus1, Pwp2, R3hdm2, Rab11fip5, Rab3ip, Rabgef1, Rai14, Rapgef5, Rars2, Rasgrf2, Rbm28, Rbm39, Rbm6, Rdh10, Relb, Retsat, Rexo1, Rfc5, Rfwd2, Rhbdf2, Rhot2, Rint1, Rnaseh2b, Rnf17, Rnf6, Rnf7, Rngtt, Rpp38, Rps6ka5, Rrs1, Rtel1, Rtn4, Ruvbl1, S100pbp, Sacm1l, Sbf1, Sbf2, Sdad1, Sec22b, Sec23ip, Senp6, Serpina3g, Setd5, Sf1, Sgsh, Sgsm1, Sh3rf1, Sim1, Sin3b, Slc17a8, Slc25a30, Slc25a35, Slc25a41, Slc27a3, Slc35a3, Slfn9, Sltm, Snrpa, Snrpb, Sod3, Sox6, Sox7, Sptlc1, Srd5a1, St6galnac6, Stambp, Stat4, Stat5b, Stim1, Stxbp1, Surf6, Svs2, Swap70, Syce1, Synpo2, Syt1, Syt2, Syt6, Taf1a, Tars2, Tbc1d1, Tcfe2a, Tcof1, Tesk2, Tfip11, Thrb, Tial1, Tjp1, Tm7sf2, Tomm20l, Tomm70a, Top1, Top2b, Tpo, Tpp1, Trappc10, Trdmt1, Trim28, Trim33, Trim37, Trim8, Trps1, Trrap, Tsfm, Tshz1, Ttc35, Ube3a, Ublcp1, Ubxn2b, Ust, Utp20, Vps11, Vps18, Vps36, Wasf1, Whsc1l1, Wrn, Xrcc3, Yeats2, Zbtb17, Zfp28, Zfp287, Zfp300, Zfp386, Zfp429, Zfp442, Zfp446, Zfp709, Zfp71-rs1, Zfy1, Zfy2, Zhx3, Zmym3, Zmym4]
GO:0005578	proteinaceous extracellular matrix	27	10.18868	0.014035	[Adam26a, Adamts2, Angptl4, Col1a2, Col4a1, Col4a3, Col9a1, Crtap, Emid1, Fras1, Gpc6, Lamc1, Lamc3, Mmp2, Mmp20, Ntng2, Omd, Otog, Papln, Podn, Postn, Rbp3, Tfip11, Tgfb1, Tnfrsf11b, Vcan, Wnt6]
GO:0031012	extracellular matrix	27	10.03718	0.014905	[Adam26a, Adamts2, Angptl4, Col1a2, Col4a1, Col4a3, Col9a1, Crtap, Emid1, Fras1, Gpc6, Lamc1, Lamc3, Mmp2, Mmp20, Ntng2, Omd, Otog, Papln, Podn, Postn, Rbp3, Tfip11, Tgfb1, Tnfrsf11b, Vcan, Wnt6]
GO:0035253	ciliary rootlet	3	33.33333	0.015293	[Kif5b, Klc1, Klc2]
GO:0000808	origin recognition complex	2	50	0.021425	[Mcm2, Orc3l]
GO:0005664	nuclear origin of replication recognition complex	2	50	0.021425	[Mcm2, Orc3l]
GO:0000267	cell fraction	43	8.865979	0.021679	[Abcb11, Abcc4, Abcg5, Acsl4, Atp2b2, Chn2, Cyfip2, Cyp1a1, Cyp1a2, Cyp2a5, Cyp2c38, Cyp2c39, Cyp2c54, Cyp2j5, Cyp4b1, Fmo4, Fn3k, G6pc, Gria2, Grid2ip, Grin1, Grm1, Homer1, Hrh4, Hspb1, Inpp5j, Jakmip1, Jph3, Kras, Mapk8ip3, Mpdz, Pgrmc1, Plxna1, Pygl, Rdh10, Sh3kbp1, Slc17a8, Sltm, Srd5a1, Syt2, Tbxas1, Tpp1, Usp14]
GO:0019717	synaptosome	8	13.7931	0.026716	[Chn2, Cyfip2, Grid2ip, Grin1, Mpdz, Sh3kbp1, Slc17a8, Usp14]
GO:0015630	microtubule cytoskeleton	28	9.523809	0.027406	[Akap9, Apc2, Appbp2, Bbs4, Cenpj, Dnm2, Dync2h1, Eml1, Hook2, Hook3, Jakmip1, Kif12, Kif26a, Kif2c, Kif5b, Kif9, Kifc1, Klc1, Klc2, Klc4, Mefv, Nudc, Ranbp1, Sac3d1, Shroom3, Strbp, Tbca, Tekt4]
GO:0005634	nucleus	274	6.986231	0.030003	[Actl7a, Adnp2, Aebp1, Aire, Ankrd34b, Appbp2, Aptx, Arhgap22, Asxl2, Atoh8, Baz2a, Bnc1, Bnc2, Brip1, Btf3, Bub3, Cbx5, Ccnb3, Ccno, Cdk2, Cdk4, Cdkl1, Cebpz, Cenpb, Chd7, Chek1, Cic, Cops4, Cops7b, Cpsf2, Cpsf6, Cramp1l, Creb3l2, Ctr9, Cux2, Dazap1, Ddx21, Ddx27, Ddx3x, Ddx3y, Ddx50, Dis3, Dmrt1, Dtx2, E2f8, Ehmt1, Eif2s1, Eif3l, Eif4a3, Eif6, Epc2, Ercc2, Esco1, Esr2, Evi5, Fam50a, Fam53a, Fancm, Fert2, Fgfr2, Fhl2, Fip1l1, Fmr1, Foxj3, Foxl2,

					Foxn3, Gabpb2, Gfi1, Gli2, Gli3, Glis1, Gnptab, Grhl2, Grm1, Gtf2h1, Gvin1, Gzf1, Hdgf, Hdgfrp3, Hivep3, Hltf, Hnrnpul2, Hoxb13, Hsf1, Hspb1, Hsph1, Ik, Ikzf2, Ikzf4, Ipo5, Isl2, Itgam, Khsrp, Kif2c, Kifc1, Kntc1, Kpnb1, Larp7, Lig1, Lig3, Lin54, Lrdd, Lyn, Magi3, Map3k2, Mbd3, Mcm2, Mcm3ap, Mcm7, Mecr, Med12l, Med15, Med27, Meox2, Mkl2, Mkx, Mlh3, Morc1, Msh2, Mta1, Mta3, Mus81, Mutyh, Mybl1, Mycbp, Myef2, Mypn, Narg2, Ncapg2, Ncoa2, Nfkbid, Nfrkb, Nfx1, Nipbl, Nle1, Nlrp12, Nol12, Nono, Nop56, Nosip, Notch2, Npat, Nr1h3, Nr2c2, Nr3c1, Nr3c2, Nsl1, Nub1, Nudc, Nuf2, Nup155, Orc3l, Pa2g4, Papolg, Parp12, Parp6, Parva, Pawr, Pelp1, Per3, Pgr, Phf17, Phrf1, Phtf1, Pkd1, Pkn2, Pmf1, Polr2b, Pparg, Ppfibp1, Ppil2, Ppp1ca, Ppp1cc, Ppp1r13b, Ppp2r5c, Prdm5, Prdm6, Prickle1, Prmt1, Prpf6, Psip1, Psma1, Ptma, Purb, Purg, Pus1, Pwp2, R3hdm2, Rab3ip, Rapgef5, Rbm28, Rbm39, Rbm6, Relb, Retsat, Rexo1, Rfc5, Rfwd2, Rnaseh2b, Rnf17, Rnf6, Rnf7, Rngtt, Rpp38, Rps6ka5, Rrs1, Rtel1, Rtn4, Ruvbl1, S100pbp, Sbf1, Sdad1, Senp6, Serpina3g, Setd5, Sf1, Sim1, Sin3b, Slfn9, Sltm, Snrpa, Snrpb, Sox6, Sox7, Stambp, Stat4, Stat5b, Surf6, Swap70, Syce1, Synpo2, Taf1a, Tbc1d1, Tcfe2a, Tcof1, Tesk2, Tfip11, Thrb, Tial1, Tjp1, Top1, Top2b, Trdmt1, Trim28, Trim33, Trim8, Trps1, Trrap, Tshz1, Ttc35, Ube3a, Ublcp1, Ubxn2b, Utp20, Vps36, Whsc1l1, Wrn, Xrcc3, Yeats2, Zbtb17, Zfp28, Zfp287, Zfp300, Zfp386, Zfp429, Zfp442, Zfp446, Zfp709, Zfp71-rs1, Zfy1, Zfy2, Zhx3, Zmym3, Zmym4]
GO:0016323	basolateral plasma membrane	10	12.34568	0.034104	[Ctnna2, Dlg1, Egfr, Enah, Parva, Pxn, Slc12a6, Sltm, Sorbs1, Tjp1]
GO:0031461	cullin-RING ubiquitin ligase complex	3	25	0.034824	[Cul1, Cul2, Cul5]
GO:0005875	microtubule associated complex	10	12.19512	0.036002	[Dync2h1, Kif12, Kif26a, Kif2c, Kif5b, Kif9, Kifc1, Klc1, Klc2, Klc4]
GO:0005626	insoluble fraction	39	8.666667	0.037463	[Abcb11, Abcc4, Abcg5, Acsl4, Atp2b2, Chn2, Cyfip2, Cyp1a1, Cyp1a2, Cyp2a5, Cyp2c38, Cyp2c39, Cyp2c54, Cyp2j5, Cyp4b1, Fmo4, G6pc, Gria2, Grid2ip, Grin1, Grm1, Homer1, Hrh4, Hspb1, Inpp5j, Jakmip1, Jph3, Kras, Mpdz, Pgrmc1, Plxna1, Rdh10, Sh3kbp1, Slc17a8, Sltm, Srd5a1, Syt2, Tbxas1, Usp14]
GO:0005624	membrane fraction	38	8.656036	0.044651	[Abcb11, Abcc4, Abcg5, Acsl4, Atp2b2, Chn2, Cyfip2, Cyp1a1, Cyp1a2, Cyp2a5, Cyp2c38, Cyp2c39, Cyp2c54, Cyp2j5, Cyp4b1, Fmo4, G6pc, Gria2, Grid2ip, Grin1, Grm1, Homer1, Hrh4, Inpp5j, Jakmip1, Jph3, Kras, Mpdz, Pgrmc1, Plxna1, Rdh10, Sh3kbp1, Slc17a8, Sltm, Srd5a1, Syt2, Tbxas1, Usp14]
GO:0005887	integral to plasma membrane	18	4.026846	0.047662	[Abcc3, Atp2b1, Bcam, Dsg3, Folh1, Gpc6, Gria2, Grin1, Hrh4, Itga1, Itgam, Kcna1, Kcnq5, Kctd12, Kctd21, Lepr, Notch2, Ror1]
GO:0005587	collagen type IV	2	33.33333	0.049266	[Col4a1, Col4a3]
GO:0030935	sheet-forming collagen	2	33.33333	0.049266	[Col4a1, Col4a3]
GO_MolecularFunction –up-regulated dataset					
GO:0005524	ATP binding	150	12.15559	7.80E-16	[Aars, Aars2, Abca2, Abca8a, Abcb10, Abcb11, Abcb7, Abcb8, Abcc3, Abcc4, Abcg5, Acot12, Actr1a, Actr5, Adrbk1, Afg3l1, Araf, Atp10b, Atp2a1, Atp2b1, Atp2b2, Atp2c1, Atp8a1, Axl, Brip1, Cct8, Cdk2, Cdk3, Cdk4, Cdk6, Cdkl1, Chd7, Chek1, Cit, Clpb, Cps1, D1Pas1, Dapk1, Ddx1, Ddx10, Ddx18, Ddx21, Ddx24,

					Ddx27, Ddx3x, Ddx3y, Ddx50, Dicer1, Dync2h1, Egfr, Ehd1, Eif4a3, Eif4b, Epha7, Ephb6, Ercc2, Fancm, Fastkd1, Fastkd2, Fert2, Fgfr1, Fgfr2, Fgfr4, Fgr, Fyn, Grk4, Hck, Hltf, Hsph1, Ick, Irak4, Jak3, Kalrn, Khsrp, Kif12, Kif26a, Kif2c, Kif5b, Kif9, Kifc1, Ksr2, Lck, Lig1, Lig3, Lyn, Magi3, Mak, Map3k13, Map3k14, Map3k15, Map3k2, Map3k9, Map4k3, Mapk11, Mapk13, Mark4, Mcm2, Mcm7, Mlkl, Morc1, Morc2a, Morc2b, Msh2, Myo15, Myo16, Myo1e, Myo3a, Myo7a, Nek5, Nlrp12, Nlrp14, Nlrp1b, Nlrp1c, Nlrp9b, Nlrp9c, Nlrx1, Nme3, Pan3, Pdk2, Pgs1, Pik3c3, Pkdcc, Pkn2, Prkd1, Prkg2, Rars2, Rfc5, Ripk4, Ror1, Rps6ka5, Ruvbl1, Sltm, Smchd1, Smok3a, Swap70, Tars2, Tarsl2, Tec, Tesk2, Top2b, Tpk1, Trappc10, Ttll8, Wars, Wnk2, Wnk4, Wrn, Xrcc3, Yes1, Zranb3]
GO:0032559	adenyl ribonucleotide binding	150	12.00961	2.89E-15	[Aars, Aars2, Abca2, Abca8a, Abcb10, Abcb11, Abcb7, Abcb8, Abcc3, Abcc4, Abcg5, Acot12, Actr1a, Actr5, Adrbk1, Afg3l1, Araf, Atp10b, Atp2a1, Atp2b1, Atp2b2, Atp2c1, Atp8a1, Axl, Brip1, Cct8, Cdk2, Cdk3, Cdk4, Cdk6, Cdkl1, Chd7, Chek1, Cit, Clpb, Cps1, D1Pas1, Dapk1, Ddx1, Ddx10, Ddx18, Ddx21, Ddx24, Ddx27, Ddx3x, Ddx3y, Ddx50, Dicer1, Dync2h1, Egfr, Ehd1, Eif4a3, Eif4b, Epha7, Ephb6, Ercc2, Fancm, Fastkd1, Fastkd2, Fert2, Fgfr1, Fgfr2, Fgfr4, Fgr, Fyn, Grk4, Hck, Hltf, Hsph1, Ick, Irak4, Jak3, Kalrn, Khsrp, Kif12, Kif26a, Kif2c, Kif5b, Kif9, Kifc1, Ksr2, Lck, Lig1, Lig3, Lyn, Magi3, Mak, Map3k13, Map3k14, Map3k15, Map3k2, Map3k9, Map4k3, Mapk11, Mapk13, Mark4, Mcm2, Mcm7, Mlkl, Morc1, Morc2a, Morc2b, Msh2, Myo15, Myo16, Myo1e, Myo3a, Myo7a, Nek5, Nlrp12, Nlrp14, Nlrp1b, Nlrp1c, Nlrp9b, Nlrp9c, Nlrx1, Nme3, Pan3, Pdk2, Pgs1, Pik3c3, Pkdcc, Pkn2, Prkd1, Prkg2, Rars2, Rfc5, Ripk4, Ror1, Rps6ka5, Ruvbl1, Sltm, Smchd1, Smok3a, Swap70, Tars2, Tarsl2, Tec, Tesk2, Top2b, Tpk1, Trappc10, Ttll8, Wars, Wnk2, Wnk4, Wrn, Xrcc3, Yes1, Zranb3]
GO:0030554	adenyl nucleotide binding	153	11.67939	1.32E-14	[Aars, Aars2, Abca2, Abca8a, Abcb10, Abcb11, Abcb7, Abcb8, Abcc3, Abcc4, Abcg5, Acot12, Acoxl, Actr1a, Actr5, Adrbk1, Afg3l1, Araf, Atp10b, Atp2a1, Atp2b1, Atp2b2, Atp2c1, Atp8a1, Axl, Brip1, Cct8, Cdk2, Cdk3, Cdk4, Cdk6, Cdkl1, Chd7, Chek1, Cit, Clpb, Cps1, D1Pas1, Dapk1, Ddx1, Ddx10, Ddx18, Ddx21, Ddx24, Ddx27, Ddx3x, Ddx3y, Ddx50, Dhcr24, Dicer1, Dync2h1, Egfr, Ehd1, Eif4a3, Eif4b, Epha7, Ephb6, Ercc2, Fancm, Fastkd1, Fastkd2, Fert2, Fgfr1, Fgfr2, Fgfr4, Fgr, Fmo4, Fyn, Grk4, Hck, Hltf, Hsph1, Ick, Irak4, Jak3, Kalrn, Khsrp, Kif12, Kif26a, Kif2c, Kif5b, Kif9, Kifc1, Ksr2, Lck, Lig1, Lig3, Lyn, Magi3, Mak, Map3k13, Map3k14, Map3k15, Map3k2, Map3k9, Map4k3, Mapk11, Mapk13, Mark4, Mcm2, Mcm7, Mlkl, Morc1, Morc2a, Morc2b, Msh2, Myo15, Myo16, Myo1e, Myo3a, Myo7a, Nek5, Nlrp12, Nlrp14, Nlrp1b, Nlrp1c, Nlrp9b, Nlrp9c, Nlrx1, Nme3, Pan3, Pdk2, Pgs1, Pik3c3, Pkdcc, Pkn2, Prkd1, Prkg2, Rars2, Rfc5, Ripk4, Ror1, Rps6ka5, Ruvbl1, Sltm, Smchd1, Smok3a, Swap70, Tars2, Tarsl2, Tec, Tesk2, Top2b, Tpk1, Trappc10, Ttll8, Wars, Wnk2, Wnk4, Wrn, Xrcc3, Yes1, Zranb3]
GO:0032553	ribonucleotide binding	171	11.00386	6.77E-14	[Aars, Aars2, Abca2, Abca8a, Abcb10, Abcb11, Abcb7, Abcb8, Abcc3, Abcc4, Abcg5, Acot12, Actr1a, Actr5, Adrbk1, Afg3l1, Araf, Arl3, Arl5b, Atp10b, Atp2a1, Atp2b1, Atp2b2, Atp2c1, Atp8a1, Axl, Brip1, Cct8, Cdk2, Cdk3, Cdk4, Cdk6, Cdkl1, Chd7, Chek1, Cit, Clpb, Cps1, D1Pas1, Dapk1, Ddx1, Ddx10, Ddx18, Ddx21, Ddx24, Ddx27, Ddx3x, Ddx3y, Ddx50, Dicer1, Dnm2, Dock6, Dock7, Dync2h1, Egfr, Ehd1, Eif2s3x, Eif2s3y, Eif4a3, Eif4b, Epha7, Ephb6, Ercc2,

					Fancm, Fastkd1, Fastkd2, Fert2, Fgfr1, Fgfr2, Fgfr4, Fgr, Fyn, Gimap1, Gimap8, Gna15, Grk4, Gspt1, Gtpbp2, Gvin1, Hck, Hltf, Hras1, Hsph1, Ick, Irak4, Jak3, Kalrn, Khsrp, Kif12, Kif26a, Kif2c, Kif5b, Kif9, Kifc1, Kras, Ksr2, Lck, Lig1, Lig3, Lyn, Magi3, Mak, Map3k13, Map3k14, Map3k15, Map3k2, Map3k9, Map4k3, Mapk11, Mapk13, Mark4, Mcm2, Mcm7, Mlkl, Morc1, Morc2a, Morc2b, Msh2, Myo15, Myo16, Myo1e, Myo3a, Myo7a, Nek5, Nlrp12, Nlrp14, Nlrp1b, Nlrp1c, Nlrp9b, Nlrp9c, Nlrx1, Nme3, Pan3, Pck2, Pde5a, Pdk2, Pgs1, Pik3c3, Pkdcc, Pkn2, Prkd1, Prkg2, Rala, Ralb, Rars2, Rfc5, Rhot2, Ripk4, Ror1, Rps6ka5, Ruvbl1, Sltm, Smchd1, Smok3a, Spag1, Swap70, Tars2, Tarsl2, Tec, Tesk2, Top2b, Tpk1, Trappc10, Ttll8, Wars, Wnk2, Wnk4, Wrn, Xrcc3, Yes1, Zranb3]
GO:0032555	purine ribonucleotide binding	171	11.00386	6.77E-14	[Aars, Aars2, Abca2, Abca8a, Abcb10, Abcb11, Abcb7, Abcb8, Abcc3, Abcc4, Abcg5, Acot12, Actr1a, Actr5, Adrbk1, Afg3l1, Araf, Arl3, Arl5b, Atp10b, Atp2a1, Atp2b1, Atp2b2, Atp2c1, Atp8a1, Axl, Brip1, Cct8, Cdk2, Cdk3, Cdk4, Cdk6, Cdkl1, Chd7, Chek1, Cit, Clpb, Cps1, D1Pas1, Dapk1, Ddx1, Ddx10, Ddx18, Ddx21, Ddx24, Ddx27, Ddx3x, Ddx3y, Ddx50, Dicer1, Dnm2, Dock6, Dock7, Dync2h1, Egfr, Ehd1, Eif2s3x, Eif2s3y, Eif4a3, Eif4b, Epha7, Ephb6, Ercc2, Fancm, Fastkd1, Fastkd2, Fert2, Fgfr1, Fgfr2, Fgfr4, Fgr, Fyn, Gimap1, Gimap8, Gna15, Grk4, Gspt1, Gtpbp2, Gvin1, Hck, Hltf, Hras1, Hsph1, Ick, Irak4, Jak3, Kalrn, Khsrp, Kif12, Kif26a, Kif2c, Kif5b, Kif9, Kifc1, Kras, Ksr2, Lck, Lig1, Lig3, Lyn, Magi3, Mak, Map3k13, Map3k14, Map3k15, Map3k2, Map3k9, Map4k3, Mapk11, Mapk13, Mark4, Mcm2, Mcm7, Mlkl, Morc1, Morc2a, Morc2b, Msh2, Myo15, Myo16, Myo1e, Myo3a, Myo7a, Nek5, Nlrp12, Nlrp14, Nlrp1b, Nlrp1c, Nlrp9b, Nlrp9c, Nlrx1, Nme3, Pan3, Pck2, Pde5a, Pdk2, Pgs1, Pik3c3, Pkdcc, Pkn2, Prkd1, Prkg2, Rala, Ralb, Rars2, Rfc5, Rhot2, Ripk4, Ror1, Rps6ka5, Ruvbl1, Sltm, Smchd1, Smok3a, Spag1, Swap70, Tars2, Tarsl2, Tec, Tesk2, Top2b, Tpk1, Trappc10, Ttll8, Wars, Wnk2, Wnk4, Wrn, Xrcc3, Yes1, Zranb3]
GO:0017076	purine nucleotide binding	174	10.77399	2.77E-13	[Aars, Aars2, Abca2, Abca8a, Abcb10, Abcb11, Abcb7, Abcb8, Abcc3, Abcc4, Abcg5, Acot12, Acoxl, Actr1a, Actr5, Adrbk1, Afg3l1, Araf, Arl3, Arl5b, Atp10b, Atp2a1, Atp2b1, Atp2b2, Atp2c1, Atp8a1, Axl, Brip1, Cct8, Cdk2, Cdk3, Cdk4, Cdk6, Cdkl1, Chd7, Chek1, Cit, Clpb, Cps1, D1Pas1, Dapk1, Ddx1, Ddx10, Ddx18, Ddx21, Ddx24, Ddx27, Ddx3x, Ddx3y, Ddx50, Dhcr24, Dicer1, Dnm2, Dock6, Dock7, Dync2h1, Egfr, Ehd1, Eif2s3x, Eif2s3y, Eif4a3, Eif4b, Epha7, Ephb6, Ercc2, Fancm, Fastkd1, Fastkd2, Fert2, Fgfr1, Fgfr2, Fgfr4, Fgr, Fmo4, Fyn, Gimap1, Gimap8, Gna15, Grk4, Gspt1, Gtpbp2, Gvin1, Hck, Hltf, Hras1, Hsph1, Ick, Irak4, Jak3, Kalrn, Khsrp, Kif12, Kif26a, Kif2c, Kif5b, Kif9, Kifc1, Kras, Ksr2, Lck, Lig1, Lig3, Lyn, Magi3, Mak, Map3k13, Map3k14, Map3k15, Map3k2, Map3k9, Map4k3, Mapk11, Mapk13, Mark4, Mcm2, Mcm7, Mlkl, Morc1, Morc2a, Morc2b, Msh2, Myo15, Myo16, Myo1e, Myo3a, Myo7a, Nek5, Nlrp12, Nlrp14, Nlrp1b, Nlrp1c, Nlrp9b, Nlrp9c, Nlrx1, Nme3, Pan3, Pck2, Pde5a, Pdk2, Pgs1, Pik3c3, Pkdcc, Pkn2, Prkd1, Prkg2, Rala, Ralb, Rars2, Rfc5, Rhot2, Ripk4, Ror1, Rps6ka5, Ruvbl1, Sltm, Smchd1, Smok3a, Spag1, Swap70, Tars2, Tarsl2, Tec, Tesk2, Top2b, Tpk1, Trappc10, Ttll8, Wars, Wnk2, Wnk4, Wrn, Xrcc3, Yes1, Zranb3]
GO:0070035	purine NTP-dependent helicase activity	18	27.27273	6.95E-08	[Brip1, Chd7, D1Pas1, Ddx1, Ddx10, Ddx18, Ddx21, Ddx24, Ddx27, Ddx3x, Ddx3y, Ddx50, Dicer1, Eif4a3, Eif4b, Ercc2, Fancm, Wrn]

GO:0016887	ATPase activity	36	16.28959	1.07E-07	[Abca2, Abca8a, Abcb10, Abcb11, Abcb7, Abcc3, Abcg5, Atp2a1, Atp2b1, Atp2b2, Atp2c1, Atp5g2, Atp8a1, Brip1, Chd7, D1Pas1, Ddx1, Ddx10, Ddx18, Ddx21, Ddx24, Ddx27, Ddx3x, Ddx3y, Ddx50, Dicer1, Dync2h1, Eif4a3, Eif4b, Ercc2, Fancm, Hltf, Msh2, Rfc5, Wrn, Xrcc3]
GO:0046872	metal ion binding	287	8.151094	3.93E-07	[Ace, Acsl4, Adam26a, Adamts2, Adcy1, Adcy6, Add2, Adnp2, Aebp1, Afg3l1, Agbl1, Aif1, Aire, Alad, Aptx, Araf, Arap2, Asap1, Asxl2, Atp10b, Atp2a1, Atp2b2, Atp2c1, Atp8a1, Avil, Baz2a, Bhmt, Bnc1, Bnc2, C330005M16Rik, Cacna1h, Cacnb1, Capn3, Ccbe1, Cdh20, Cdipt, Chn2, Chsy3, Cit, Col9a1, Crip2, Csgalnact1, Cyp1a1, Cyp1a2, Cyp27a1, Cyp2a5, Cyp2c38, Cyp2c39, Cyp2c44, Cyp2c54, Cyp2j5, Cyp2j9, Cyp4b1, Dbh, Ddhd2, Dgka, Dmrt1, Dnaja1, Dnaja2, Dnaja4, Dpep2, Dpys, Dsg1a, Dsg1b, Dsg3, Dsg4, Dtnb, Dtx2, Dzip3, EG317677, Ece2, Efhd2, Ehd1, Ercc2, Esco1, Esr2, Extl3, F10, F7, Fam20c, Fbxo43, Fdx1, Fgd5, Fgg, Fhl2, Fhl4, Flg2, Folh1, Fras1, Fstl4, Fstl5, Ftmt, Fyco1, Fyn, Galnt3, Galnt5, Gfi1, Gli3, Glis1, Gmip, Gnptab, Grin1, Gzf1, Hivep3, Hltf, Ick, Ide, Ikzf2, Ikzf4, Impa2, Irak4, Isl2, Itga1, Itgam, Itpa, Kalrn, Kcna1, Kcnh8, Kcnip1, Kcnq5, Ksr2, Lcp1, Lig3, Lima1, Lonrf3, Man2a1, Man2b1, Map3k13, Map3k15, Map3k2, Mcm2, Mctp2, Mecr, Mefv, Megf8, Mmp2, Mmp20, Mocos, Morc1, Morc2a, Morc2b, Mpo, Mta1, Mta3, Mus81, Mutyh, Naalad2, Nalcn, Nebl, Nek5, Nfx1, Nme3, Notch2, Npepl1, Nr1h3, Nr2c2, Nr3c1, Nr3c2, Nt5c, Nt5c2, Nt5c3, Papln, Parp12, Pcdh17, Pcdhb17, Pck2, Pde5a, Pdzd8, Pepd, Pgm3, Pgr, Pgrmc1, Phf17, Phf8, Phrf1, Pik3c3, Pitpnm3, Pla2g2e, Pla2g4d, Plcb2, Plce1, Plch2, Plcl1, Plekhm1, Plod3, Pmpca, Polg, Polr2b, Pparg, Ppef1, Ppm1a, Ppm1d, Ppp1ca, Ppp1cc, Ppp3cb, Prdm5, Prickle1, Prickle3, Prkd1, Ptgis, Ptprg, Pxn, Rabgef1, Rabggta, Rasgrf2, Reps1, Rfwd2, Rgnef, Rhot2, Rnf114, Rnf123, Rnf17, Rnf7, Rps6ka5, Rxfp1, S100a13, Scel, Scin, Sec23ip, Senp6, Sf1, Sh3rf1, Slc12a6, Slc17a1, Slc17a8, Slc34a2, Slc38a8, Slit3, Sod3, Stambp, Stat4, Stat5b, Stim1, Syt1, Syt2, Syt6, Tbc1d9b, Tbxas1, Tec, Tesk2, Thbs2, Thrb, Top3a, Tpo, Trim16, Trim28, Trim29, Trim31, Trim33, Trim37, Trim8, Trpc4, Trps1, Tshz1, Ubr1, Unc13a, Vcan, Vps11, Vps18, Whsc1l1, Zbtb17, Zc3h12c, Zc3h13, Zc3h4, Zcwpw1, Zfp101, Zfp119, Zfp28, Zfp287, Zfp300, Zfp385a, Zfp386, Zfp429, Zfp442, Zfp446, Zfp454, Zfp709, Zfp71-rs1, Zfp94, Zfy1, Zfy2, Zhx3, Zmym3, Zmym4, Zranb3, Zswim4, Zswim5]
GO:0003779	actin binding	38	14.61539	8.73E-07	[2610204M08Rik, Abra, Add2, Avil, Baiap2l1, Coro1b, Ctnna1, Dbnl, Enah, Eps8l2, Fhdc1, Fmn2, Fmnl3, Gmfb, Gmfg, Grid2ip, Klhl17, Lcp1, Lima1, Mefv, Mkl2, Mybpc3, Myo15, Myo16, Myo3a, Myo7a, Mypn, Parva, Pof1b, Scin, Shroom3, Ssh2, Synpo2, Tmod1, Tmod3, Vps18, Wasf1, Xirp1]
GO:0008092	cytoskeletal protein binding	47	13.42857	8.74E-07	[2610204M08Rik, Abra, Add2, Apc2, Appbp2, Avil, Baiap2l1, Cenpj, Coro1b, Ctnna1, Dbnl, Enah, Eps8l2, Fhdc1, Fmn2, Fmnl3, Frmd4b, Fyn, Gmfb, Gmfg, Grid2ip, Hook2, Hook3, Klhl17, Lcp1, Lima1, Mapk8ip3, Mefv, Mkl2, Mybpc3, Myo15, Myo16, Myo3a, Myo7a, Mypn, Pacsin1, Parva, Pof1b, Scin, Shroom3, Ssh2, Synpo2, Tmod1, Tmod3, Vps18, Wasf1, Xirp1]
GO:0042623	ATPase activity, coupled	30	16.30435	1.19E-06	[Abcb10, Abcb11, Abcb7, Abcc3, Atp2a1, Atp2b1, Atp2b2, Atp2c1, Atp5g2, Atp8a1, Brip1, Chd7, D1Pas1, Ddx1, Ddx10, Ddx18, Ddx21, Ddx24, Ddx27, Ddx3x, Ddx3y, Ddx50, Dicer1, Eif4a3, Eif4b, Ercc2, Fancm, Rfc5, Wrn, Xrcc3]

GO:0004386	helicase activity	23	19.16667	1.22E-06	[Brip1, Chd7, D1Pas1, Ddx1, Ddx10, Ddx18, Ddx21, Ddx24, Ddx27, Ddx3x, Ddx3y, Ddx50, Dicer1, Eif4a3, Eif4b, Ercc2, Fancm, Hltf, Mcm7, Rtel1, Ruvbl1, Wrn, Zranb3]
GO:0043169	cation binding	264	8.125577	1.72E-06	[Ace, Adam26a, Adamts2, Adnp2, Aebp1, Afg3l1, Agbl1, Aif1, Aire, Alad, Aptx, Araf, Arap2, Asap1, Asxl2, Atp2a1, Atp2b2, Atp2c1, Avil, Baz2a, Bhmt, Bnc1, Bnc2, C330005M16Rik, Cacna1h, Cacnb1, Capn3, Ccbe1, Cdh20, Cdipt, Chid1, Chn2, Cit, Col9a1, Crip2, Cyp1a1, Cyp1a2, Cyp27a1, Cyp2a5, Cyp2c38, Cyp2c39, Cyp2c44, Cyp2c54, Cyp2j5, Cyp2j9, Cyp4b1, Dbh, Dgka, Dmrt1, Dnaja1, Dnaja2, Dnaja4, Dpep2, Dpys, Dsg1a, Dsg1b, Dsg3, Dsg4, Dtnb, Dtx2, Dzip3, EG317677, Ece2, Efhd2, Ehd1, Esco1, Esr2, Extl3, F10, F7, Fam20c, Fbxo43, Fdx1, Fgd5, Fgg, Fhl2, Fhl4, Flg2, Folh1, Fras1, Fstl4, Fstl5, Ftmt, Fyco1, Fyn, Galnt3, Galnt5, Gbe1, Gfi1, Glb1l2, Gli3, Glis1, Gmip, Gnptab, Grin1, Gzf1, Hivep3, Hltf, Ide, Ikzf2, Ikzf4, Impa2, Isl2, Itga1, Itgam, Kcna1, Kcnh8, Kcnip1, Kcnq5, Ksr2, Lcp1, Lig3, Lima1, Lonrf3, Man2a1, Man2b1, Mcm2, Mctp2, Mecr, Mefv, Megf8, Mmp2, Mmp20, Mocos, Morc1, Morc2a, Morc2b, Mpo, Mta1, Mta3, Mutyh, Naalad2, Naga, Nalcn, Nebl, Nfx1, Notch2, Npepl1, Nr1h3, Nr2c2, Nr3c1, Nr3c2, Papln, Parp12, Pcdh17, Pcdhb17, Pck2, Pde5a, Pdzd8, Pepd, Pgr, Pgrmc1, Phf17, Phf8, Phrf1, Pik3c3, Pitpnm3, Pla2g2e, Pla2g4d, Plcb2, Plce1, Plch2, Plcl1, Plekhm1, Plod3, Pmpca, Polr2b, Pparg, Ppef1, Ppm1a, Ppm1d, Ppp1ca, Ppp1cc, Ppp3cb, Prdm5, Prickle1, Prickle3, Prkd1, Ptgis, Ptprg, Pxn, Rabgef1, Rabggta, Rasgrf2, Reps1, Rfwd2, Rgnef, Rhot2, Rnf114, Rnf123, Rnf17, Rnf7, Rxfp1, S100a13, Scel, Scin, Senp6, Sf1, Sh3rf1, Slc12a6, Slc17a1, Slc17a8, Slc34a2, Slc38a8, Slit3, Sod3, Stambp, Stat4, Stat5b, Stim1, Syt1, Syt2, Syt6, Tbc1d9b, Tbxas1, Tec, Tesk2, Thbs2, Thrb, Top3a, Tpo, Trim16, Trim28, Trim29, Trim31, Trim33, Trim37, Trim8, Trpc4, Trps1, Tshz1, Ubr1, Unc13a, Vcan, Vps11, Vps18, Whsc1l1, Zbtb17, Zc3h12c, Zc3h13, Zc3h4, Zcwpw1, Zfp101, Zfp119, Zfp28, Zfp287, Zfp300, Zfp385a, Zfp386, Zfp429, Zfp442, Zfp446, Zfp454, Zfp709, Zfp71-rs1, Zfp94, Zfy1, Zfy2, Zhx3, Zmym3, Zmym4, Zranb3, Zswim4, Zswim5]
GO:0004872	receptor activity	112	4.260175	2.76E-06	[Adcyap1r1, Adrbk1, Atp6ap2, Axl, Bcam, C130060K24Rik, C3ar1, Ccr8, Cd163, Ceacam1, Ceacam2, Chrna10, Clec1a, Csf2ra, Cul5, Egfr, Epha7, Ephb6, Esr2, F830045P16Rik, Fgfr1, Fgfr2, Fgfr4, Gabra1, Gabrd, Gosr1, Gpr111, Gpr125, Gpr176, Gpr19, Gria2, Grin1, Grk4, Grm1, Hrh4, Igsf1, Il1rl1, Il28ra, Irak4, Irs2, Itga1, Itgam, Klra13, Klra18, Klra4, Klra7, Lepr, Lifr, Lphn1, Lsr, Ly75, Marco, Ncoa2, Ncr1, Nisch, Notch2, Npc1l1, Nr1h3, Nr2c2, Nr3c1, Nr3c2, Ntsr1, Olfr1033, Olfr1211, Olfr1289, Olfr134, Olfr1495, Olfr181, Olfr632, Olfr642, Olfr695, Olfr780, Olfr806, Olfr867, Paqr8, Pgr, Pgrmc1, Pigr, Plxna1, Plxna4, Plxnb3, Plxnc1, Pparg, Prpf6, Ptprb, Ptprf, Ptprg, Ptprj, Ptprt, Pvrl2, Qrfpr, Ripk4, Ror1, Rxfp1, Sltm, Sorcs1, Tas1r2, Tas2r136, Tas2r143, Tcp10c, Thrb, Tnfrsf11b, Tnfrsf1a, Tomm70a, Trip12, Trpc4, Trpm1, Unc5d, Vmn2r29, Vmn2r42, Vmn2r43, Vmn2r61]
GO:0016788	hydrolase activity, acting on ester bonds	66	11	6.48E-06	[Abhd2, Acot12, Acot7, Aptx, Aspa, C330005M16Rik, Dicer1, Dis3, Dis3l, Dub1, Dub2a, Fancm, G6pc, Hdhd3, Impa2, Inpp5j, Khsrp, Mus81, Mutyh, Nt5c, Nt5c2, Nt5c3, Pafah2, Pde5a, Pde8b, Pfkfb3, Pla2g2e, Pla2g4d, Plcb2, Plce1, Plch2, Plcl1, Polg, Ppef1, Ppm1a, Ppm1d, Ppp1ca, Ppp1cc, Ppp1r12a, Ppp1r3a,

					Ppp3cb, Ptprb, Ptprf, Ptprg, Ptprj, Ptprt, Ptprv, Rexo1, Rngtt, Rpp38, Sbf1, Sgsh, Smpdl3a, Ssh2, Stambp, Tcfe2a, Ublcp1, Uchl3, Usp14, Usp16, Usp26, Usp42, Usp47, Usp54, Wrn, Zranb3]
GO:0016462	pyrophosphatase activity	52	11.25541	3.48E-05	[Abca2, Abca8a, Abcb10, Abcb11, Abcb7, Abcc3, Abcg5, Afg3l1, Atp2a1, Atp2b1, Atp2b2, Atp2c1, Atp5g2, Atp8a1, Brip1, Chd7, Clpb, D1Pas1, Ddx1, Ddx10, Ddx18, Ddx21, Ddx24, Ddx27, Ddx3x, Ddx3y, Ddx50, Dicer1, Dnm2, Dync2h1, Ehd1, Eif2s3x, Eif2s3y, Eif4a3, Eif4b, Ercc2, Fancm, Gna15, Gspt1, Gtpbp2, Hltf, Itpa, Kras, Mcm7, Msh2, Nudt2, Rfc5, Rtel1, Ruvbl1, Wrn, Xrcc3, Zranb3]
GO:0017111	nucleoside-triphosphatase activity	50	11.36364	3.60E-05	[Abca2, Abca8a, Abcb10, Abcb11, Abcb7, Abcc3, Abcg5, Afg3l1, Atp2a1, Atp2b1, Atp2b2, Atp2c1, Atp5g2, Atp8a1, Brip1, Chd7, Clpb, D1Pas1, Ddx1, Ddx10, Ddx18, Ddx21, Ddx24, Ddx27, Ddx3x, Ddx3y, Ddx50, Dicer1, Dnm2, Dync2h1, Ehd1, Eif2s3x, Eif2s3y, Eif4a3, Eif4b, Ercc2, Fancm, Gna15, Gspt1, Gtpbp2, Hltf, Kras, Mcm7, Msh2, Rfc5, Rtel1, Ruvbl1, Wrn, Xrcc3, Zranb3]
GO:0016818	hydrolase activity, acting on acid anhydrides, in phosphorus-containing anhydrides	52	11.1828	3.87E-05	[Abca2, Abca8a, Abcb10, Abcb11, Abcb7, Abcc3, Abcg5, Afg3l1, Atp2a1, Atp2b1, Atp2b2, Atp2c1, Atp5g2, Atp8a1, Brip1, Chd7, Clpb, D1Pas1, Ddx1, Ddx10, Ddx18, Ddx21, Ddx24, Ddx27, Ddx3x, Ddx3y, Ddx50, Dicer1, Dnm2, Dync2h1, Ehd1, Eif2s3x, Eif2s3y, Eif4a3, Eif4b, Ercc2, Fancm, Gna15, Gspt1, Gtpbp2, Hltf, Itpa, Kras, Mcm7, Msh2, Nudt2, Rfc5, Rtel1, Ruvbl1, Wrn, Xrcc3, Zranb3]
GO:0016773	phosphotransferase activity, alcohol group as acceptor	68	10.24096	5.37E-05	[Adrbk1, Araf, Axl, Cdk2, Cdk3, Cdk4, Cdk6, Cdkl1, Chek1, Cit, Dapk1, Dgka, Egfr, Epha7, Ephb6, Fastkd1, Fastkd2, Fert2, Fgfr1, Fgfr2, Fgfr4, Fgr, Fyn, G6pc, Grk4, Hck, Ick, Irak4, Jak3, Kalrn, Kcnh8, Ksr2, Lck, Lyn, Mak, Map3k13, Map3k14, Map3k15, Map3k2, Map3k9, Map4k3, Mapk11, Mapk13, Mark4, Mlkl, Myo3a, Nek5, Pan3, Pdk2, Per3, Pfkfb3, Pik3c3, Pkdcc, Pkn2, Prkd1, Prkg2, Ripk4, Ror1, Rps6ka5, Sim1, Sltm, Smok3a, Tec, Tesk2, Trrap, Wnk2, Wnk4, Yes1]
GO:0016817	hydrolase activity, acting on acid anhydrides	52	11.1588	5.62E-05	[Abca2, Abca8a, Abcb10, Abcb11, Abcb7, Abcc3, Abcg5, Afg3l1, Atp2a1, Atp2b1, Atp2b2, Atp2c1, Atp5g2, Atp8a1, Brip1, Chd7, Clpb, D1Pas1, Ddx1, Ddx10, Ddx18, Ddx21, Ddx24, Ddx27, Ddx3x, Ddx3y, Ddx50, Dicer1, Dnm2, Dync2h1, Ehd1, Eif2s3x, Eif2s3y, Eif4a3, Eif4b, Ercc2, Fancm, Gna15, Gspt1, Gtpbp2, Hltf, Itpa, Kras, Mcm7, Msh2, Nudt2, Rfc5, Rtel1, Ruvbl1, Wrn, Xrcc3, Zranb3]
GO:0046914	transition metal ion binding	190	8.126604	9.26E-05	[Ace, Adam26a, Adamts2, Adnp2, Aebp1, Afg3l1, Agbl1, Aire, Alad, Aptx, Araf, Arap2, Asap1, Asxl2, Baz2a, Bhmt, Bnc1, Bnc2, C330005M16Rik, Cdipt, Chn2, Cit, Col9a1, Crip2, Cyp1a1, Cyp1a2, Cyp27a1, Cyp2a5, Cyp2c38, Cyp2c39, Cyp2c44, Cyp2c54, Cyp2j5, Cyp2j9, Cyp4b1, Dbh, Dgka, Dmrt1, Dnaja1, Dnaja2, Dnaja4, Dpep2, Dpys, Dtnb, Dtx2, Dzip3, Ece2, Esco1, Esr2, Extl3, Fbxo43, Fdx1, Fgd5, Fhl2, Fhl4, Folh1, Ftmt, Fyco1, Fyn, Galnt3, Galnt5, Gfi1, Gli3, Glis1, Gmip, Gzf1, Hivep3, Hltf, Ide, Ikzf2, Ikzf4, Isl2, Ksr2, Lig3, Lima1, Lonrf3, Man2a1, Man2b1, Mcm2, Mecr, Mefv, Mmp2, Mmp20, Mocos, Morc1, Morc2a, Morc2b, Mpo, Mta1, Mta3, Mutyh, Naalad2, Nebl, Nfx1, Npepl1, Nr1h3, Nr2c2, Nr3c1, Nr3c2, Papln, Parp12, Pck2, Pde5a, Pdzd8, Pepd, Pgr, Pgrmc1, Phf17, Phf8, Phrf1, Pik3c3, Plekhm1, Plod3, Pmpca, Polr2b, Pparg, Ppef1, Ppm1a, Ppm1d, Ppp1ca, Ppp1cc, Ppp3cb, Prdm5, Prickle1, Prickle3, Prkd1,

					Ptgis, Ptprg, Pxn, Rabgef1, Rabggta, Rfwd2, Rgnef, Rnf114, Rnf123, Rnf17, Rnf7, Scel, Senp6, Sf1, Sh3rf1, Sod3, Stambp, Tbxas1, Tec, Tesk2, Thrb, Top3a, Tpo, Trim16, Trim28, Trim29, Trim31, Trim33, Trim37, Trim8, Trps1, Tshz1, Ubr1, Unc13a, Vps11, Vps18, Whsc1l1, Zbtb17, Zc3h12c, Zc3h13, Zc3h4, Zcwpw1, Zfp101, Zfp119, Zfp28, Zfp287, Zfp300, Zfp385a, Zfp386, Zfp429, Zfp442, Zfp446, Zfp454, Zfp709, Zfp71-rs1, Zfp94, Zfy1, Zfy2, Zhx3, Zmym3, Zmym4, Zranb3, Zswim4, Zswim5]
GO:0016712	oxidoreductase activity, acting on paired donors, with incorporation or reduction of molecular oxygen, reduced flavin or flavoprotein as one donor, and incorporation of one atom of oxygen	10	25	1.30E-04	[Cyp1a1, Cyp1a2, Cyp2a5, Cyp2c38, Cyp2c39, Cyp2c44, Cyp2c54, Cyp2j5, Cyp4b1, Tbxas1]
GO:0042578	phosphoric ester hydrolase activity	35	11.9863	2.02E-04	[C330005M16Rik, G6pc, Hdhd3, Impa2, Inpp5j, Khsrp, Nt5c, Nt5c2, Nt5c3, Pde5a, Pde8b, Pfkfb3, Plcb2, Plce1, Plch2, Plcl1, Ppef1, Ppm1a, Ppm1d, Ppp1ca, Ppp1cc, Ppp1r12a, Ppp1r3a, Ppp3cb, Ptprb, Ptprf, Ptprg, Ptprj, Ptprt, Ptprv, Rngtt, Sbf1, Smpdl3a, Ssh2, Ublcp1]
GO:0050381	unspecific monooxygenase activity	9	25	2.81E-04	[Cyp1a1, Cyp1a2, Cyp2a5, Cyp2c38, Cyp2c39, Cyp2c44, Cyp2c54, Cyp2j5, Cyp4b1]
GO:0016301	kinase activity	77	9.322034	3.82E-04	[Adrbk1, Akap9, Araf, Axl, Cdk2, Cdk3, Cdk4, Cdk6, Cdkl1, Chek1, Cit, Dapk1, Dgka, Egfr, Epha7, Ephb6, Fastkd1, Fastkd2, Fert2, Fgfr1, Fgfr2, Fgfr4, Fgr, Fn3k, Fyn, Grk4, Hck, Ick, Irak4, Jak3, Jakmip1, Kalrn, Kcnh8, Ksr2, Lck, Lyn, Madd, Mak, Map3k13, Map3k14, Map3k15, Map3k2, Map3k9, Map4k3, Mapk11, Mapk13, Mapk8ip3, Mark4, Mlkl, Myo3a, Nags, Nek5, Nme3, Pacsin1, Pan3, Pck2, Pdk2, Per3, Pfkfb3, Pik3c3, Pkdcc, Pkn2, Prkd1, Prkg2, Ripk4, Ror1, Rps6ka5, Sh3kbp1, Sim1, Sltm, Smok3a, Tec, Tesk2, Tpk1, Wnk2, Wnk4, Yes1]
GO:0016772	transferase activity, transferring phosphorus-containing groups	86	9.081309	4.07E-04	[Adrbk1, Akap9, Araf, Axl, Cdipt, Cdk2, Cdk3, Cdk4, Cdk6, Cdkl1, Chek1, Cit, Dapk1, Dgka, Egfr, Epha7, Ephb6, Fastkd1, Fastkd2, Fert2, Fgfr1, Fgfr2, Fgfr4, Fgr, Fn3k, Fyn, G6pc, Gnptab, Grk4, Hck, Ick, Irak4, Jak3, Jakmip1, Kalrn, Kcnh8, Ksr2, Lck, Lyn, Madd, Mak, Map3k13, Map3k14, Map3k15, Map3k2, Map3k9, Map4k3, Mapk11, Mapk13, Mapk8ip3, Mark4, Mlkl, Myo3a, Nags, Nek5, Nme3, Pacsin1, Pan3, Papolg, Pck2, Pdk2, Per3, Pfkfb3, Pgs1, Pik3c3, Pkdcc, Pkn2, Polg, Polr2b, Prkd1, Prkg2, Ripk4, Rngtt, Ror1, Rps6ka5, Sh3kbp1, Sim1, Sltm, Smok3a, Tec, Tesk2, Tpk1, Trrap, Wnk2, Wnk4, Yes1]
GO:0008270	zinc ion binding	154	8.161102	4.13E-04	[Ace, Adam26a, Adamts2, Adnp2, Aebp1, Afg3l1, Agbl1, Aire, Alad, Aptx, Araf, Arap2, Asap1, Asxl2, Baz2a, Bhmt, Bnc1, Bnc2, C330005M16Rik, Chn2, Cit, Col9a1, Crip2, Dgka, Dmrt1, Dnaja1, Dnaja2, Dnaja4, Dpep2, Dpys, Dtnb, Dtx2, Dzip3, Ece2, Esco1, Esr2, Fbxo43, Fgd5, Fhl2, Fhl4, Folh1, Fyco1, Gfi1, Gli3, Glis1, Gmip, Gzf1, Hivep3, Hltf, Ide, Ikzf2, Ikzf4, Isl2, Ksr2, Lig3, Lima1, Lonrf3, Man2a1, Man2b1, Mcm2, Mecr, Mefv, Mmp2, Mmp20, Morc1, Morc2a, Morc2b, Mta1, Mta3, Naalad2, Nebl, Nfx1, Npepl1, Nr1h3, Nr2c2, Nr3c1, Nr3c2, Papln, Parp12, Pde5a, Pdzd8, Pgr, Phf17, Phf8, Phrf1, Plekhm1, Pmpca, Polr2b, Pparg, Ppp3cb, Prdm5, Prickle1, Prickle3, Prkd1, Ptprg, Pxn, Rabgef1, Rabggta,

					Rfwd2, Rgnef, Rnf114, Rnf123, Rnf17, Rnf7, Scel, Senp6, Sf1, Sh3rf1, Sod3, Stambp, Tec, Thrb, Top3a, Trim16, Trim28, Trim29, Trim31, Trim33, Trim37, Trim8, Trps1, Tshz1, Ubr1, Unc13a, Vps11, Vps18, Whsc1l1, Zbtb17, Zc3h12c, Zc3h13, Zc3h4, Zcwpw1, Zfp101, Zfp119, Zfp28, Zfp287, Zfp300, Zfp385a, Zfp386, Zfp429, Zfp442, Zfp446, Zfp454, Zfp709, Zfp71-rs1, Zfp94, Zfy1, Zfy2, Zhx3, Zmym3, Zmym4, Zranb3, Zswim4, Zswim5]
GO:0016791	phosphatase activity	28	12.5	4.14E-04	[C330005M16Rik, G6pc, Hdhd3, Impa2, Inpp5j, Khsrp, Nt5c, Nt5c2, Nt5c3, Pfkfb3, Ppef1, Ppm1a, Ppm1d, Ppp1ca, Ppp1cc, Ppp1r12a, Ppp1r3a, Ppp3cb, Ptprb, Ptprf, Ptprg, Ptprj, Ptprt, Ptprv, Rngtt, Sbf1, Ssh2, Ublcp1]
GO:0004715	non-membrane spanning protein tyrosine kinase activity	9	23.68421	4.34E-04	[Fert2, Fgr, Fyn, Hck, Jak3, Lck, Lyn, Tec, Yes1]
GO:0005388	calcium-transporting ATPase activity	4	50	8.60E-04	[Atp2a1, Atp2b1, Atp2b2, Atp2c1]
GO:0015085	calcium ion transmembrane transporter activity	4	50	8.60E-04	[Atp2a1, Atp2b1, Atp2b2, Atp2c1]
GO:0032947	protein complex scaffold	4	50	8.60E-04	[Dlg1, Mapk8ip3, Pxn, Sh3rf1]
GO:0008238	exopeptidase activity	13	16.88312	8.85E-04	[Ace, Aebp1, Agbl1, Dpep2, Dpp4, Dpp7, Dpp8, Folh1, Naalad2, Npepl1, Pa2g4, Pepd, Tpp1]
GO:0016798	hydrolase activity, acting on glycosyl bonds	16	14.95327	9.49E-04	[Ace, Amy2, Ccno, Chid1, Gaa, Gbe1, Glb1l2, Man1b1, Man2a1, Man2b1, Mutyh, Naga, Neu3, Otog, Smpdl3a, Treh]
GO:0004221	ubiquitin thiolesterase activity	11	17.74194	0.001422	[Dub1, Dub2a, Stambp, Tcfe2a, Uchl3, Usp14, Usp16, Usp26, Usp42, Usp47, Usp54]
GO:0004843	ubiquitin-specific protease activity	11	17.46032	0.001628	[Dub1, Dub2a, Stambp, Tcfe2a, Uchl3, Usp14, Usp16, Usp26, Usp42, Usp47, Usp54]
GO:0019783	small conjugating protein-specific protease activity	11	17.1875	0.001857	[Dub1, Dub2a, Stambp, Tcfe2a, Uchl3, Usp14, Usp16, Usp26, Usp42, Usp47, Usp54]
GO:0019992	diacylglycerol binding	10	18.18182	0.001911	[Araf, Chn2, Cit, Dgka, Gmip, Ksr2, Plekhm1, Prkd1, Rgnef, Unc13a]
GO:0004693	cyclin-dependent protein kinase activity	7	23.33333	0.002062	[Cdk2, Cdk3, Cdk4, Cdk6, Cdkl1, Ick, Mak]
GO:0016790	thiolester hydrolase activity	13	15.11628	0.002498	[Acot12, Acot7, Dub1, Dub2a, Stambp, Tcfe2a, Uchl3, Usp14, Usp16, Usp26, Usp42, Usp47, Usp54]
GO:0003678	DNA helicase activity	5	29.41176	0.003076	[Brip1, Ercc2, Mcm7, Ruvbl1, Wrn]
GO:0004709	MAP kinase kinase kinase activity	5	29.41176	0.003076	[Map3k13, Map3k14, Map3k15, Map3k2, Map3k9]
GO:0030215	semaphorin receptor binding	2	100	0.003886	[Sema3c, Sema6a]
GO:0003916	DNA topoisomerase activity	3	50	0.004194	[Top1, Top2b, Top3a]
GO:0030159	receptor signaling complex scaffold activity	3	50	0.004194	[Mapk8ip3, Pxn, Sh3rf1]
GO:0005509	calcium ion binding	70	8.75	0.004319	[Aif1, Atp2a1, Atp2b2, Atp2c1, Avil, Cacna1h, Cacnb1, Capn3, Ccbe1, Cdh20,

					Crip2, Dgka, Dsg1a, Dsg1b, Dsg3, Dsg4, Dtnb, EG317677, Efhd2, Ehd1, F10, F7, Fam20c, Fgg, Flg2, Fras1, Fstl4, Fstl5, Galnt3, Galnt5, Gnptab, Grin1, Itga1, Itgam, Kcnip1, Lcp1, Mctp2, Megf8, Mmp2, Mmp20, Mpo, Notch2, Pcdh17, Pcdhb17, Pitpnm3, Pla2g2e, Pla2g4d, Plcb2, Plce1, Plch2, Plcl1, Ppef1, Rasgrf2, Reps1, Rhot2, Rxfp1, S100a13, Scin, Slit3, Stat4, Stat5b, Stim1, Syt1, Syt2, Syt6, Tbc1d9b, Thbs2, Tpo, Trpc4, Vcan]
GO:0016805	dipeptidase activity	4	33.33333	0.004969	[Dpep2, Folh1, Naalad2, Pepd]
GO:0003713	transcription coactivator activity	10	15.87302	0.005324	[Abra, Cenpj, Mkl2, Ncoa2, Pmf1, Prpf6, Pus1, Rbm39, Trim28, Trrap]
GO:0020037	heme binding	14	13.72549	0.00569	[Cyp1a1, Cyp1a2, Cyp27a1, Cyp2a5, Cyp2c38, Cyp2c39, Cyp2c54, Cyp2j5, Cyp4b1, Mpo, Pgrmc1, Ptgis, Tbxas1, Tpo]
GO:0004553	hydrolase activity, hydrolyzing O-glycosyl compounds	12	14.28571	0.005741	[Amy2, Chid1, Gaa, Gbe1, Glb1l2, Man1b1, Man2a1, Man2b1, Naga, Neu3, Otog, Treh]
GO:0003723	RNA binding	49	9.315589	0.005752	[Alkbh8, Cpsf2, Cpsf6, D1Pas1, Dazap1, Ddx1, Ddx10, Ddx18, Ddx21, Ddx24, Ddx3x, Ddx3y, Ddx50, Dicer1, Dis3, Dis3l, Dnajc17, Dzip3, Eif2s1, Eif4a3, Eif4b, Eif4e, Eif4e2, Eif4g1, Fip1l1, Fmr1, Fxr2, Jakmip1, Khsrp, Larp7, Myef2, Nol12, Nono, Pa2g4, Papolg, Purb, Rbm28, Rbm3, Rbm39, Rgnef, Sf1, Sltm, Snrpa, Snrpb, Strbp, Surf6, Tial1, Trdmt1, Ttc14]
GO:0005085	guanyl-nucleotide exchange factor activity	16	12.69841	0.007926	[Arhgef12, Arhgef3, Arhgef6, Dock6, Dock7, Fgd5, Kalrn, Madd, Ngef, Plce1, Rab3il1, Rab3ip, Rapgef5, Rasgrf2, Rgnef, Ric8b]
GO:0004812	aminoacyl-tRNA ligase activity	8	17.02128	0.007978	[Aars, Aars2, Khsrp, Rars2, Tars2, Tarsl2, Trappc10, Wars]
GO:0016875	ligase activity, forming carbon-oxygen bonds	8	17.02128	0.007978	[Aars, Aars2, Khsrp, Rars2, Tars2, Tarsl2, Trappc10, Wars]
GO:0016876	ligase activity, forming aminoacyl-tRNA and related compounds	8	17.02128	0.007978	[Aars, Aars2, Khsrp, Rars2, Tars2, Tarsl2, Trappc10, Wars]
GO:0019904	protein domain specific binding	16	12.59843	0.008185	[Cit, Ctr9, Elmo3, Enah, Homer3, Inpp5j, Kirrel3, Myo7a, Phrf1, Ppp1r13b, Pxn, Sh3bgrl, Sh3kbp1, Shc4, Tjp1, Wbp2nl]
GO:0005097	Rab GTPase activator activity	7	18.42105	0.00828	[Evi5, Sgsm1, Sgsm2, Tbc1d1, Tbc1d2b, Tbc1d9b, Usp6nl]
GO:0005083	small GTPase regulator activity	21	11.35135	0.008295	[Arap2, Arhgef12, Arhgef3, Arhgef6, Asap1, Cit, Evi5, Fgd5, Kalrn, Madd, Map4k3, Ngef, Rab3il1, Rasgrf2, Rgnef, Sgsm1, Sgsm2, Tbc1d1, Tbc1d2b, Tbc1d9b, Usp6nl]
GO:0004714	transmembrane receptor protein tyrosine kinase activity	9	15.78947	0.008295	[Axl, Egfr, Epha7, Ephb6, Fgfr1, Fgfr2, Fgfr4, Ror1, Sltm]
GO:0003707	steroid hormone receptor activity	8	16.66667	0.009064	[Esr2, Nr1h3, Nr2c2, Nr3c1, Nr3c2, Pgr, Pparg, Thrb]
GO:0004434	inositol or phosphatidylinositol phosphodiesterase activity	4	28.57143	0.009093	[Plcb2, Plce1, Plch2, Plcl1]
GO:0004435	phosphoinositide	4	28.57143	0.009093	[Plcb2, Plce1, Plch2, Plcl1]

	phospholipase C activity				
GO:0005088	Ras guanyl-nucleotide exchange factor activity	10	14.70588	0.009157	[Arhgef12, Arhgef3, Arhgef6, Fgd5, Kalrn, Madd, Ngef, Rab3il1, Rasgrf2, Rgnef]
GO:0051082	unfolded protein binding	9	15.51724	0.00929	[Cct8, Dnaja1, Dnaja2, Dnaja4, Dnajb5, Dnajc16, Dnajc17, Slmap, Tbca]
GO:0004177	aminopeptidase activity	6	20	0.009467	[Dpp4, Dpp7, Dpp8, Npepl1, Pa2g4, Pepd]
GO:0004879	ligand-dependent nuclear receptor activity	8	16.32653	0.010256	[Esr2, Nr1h3, Nr2c2, Nr3c1, Nr3c2, Pgr, Pparg, Thrb]
GO:0001846	opsonin binding	2	66.66666	0.011174	[Cfh, Itgam]
GO:0003909	DNA ligase activity	2	66.66666	0.011174	[Lig1, Lig3]
GO:0003910	DNA ligase (ATP) activity	2	66.66666	0.011174	[Lig1, Lig3]
GO:0003918	DNA topoisomerase (ATP-hydrolyzing) activity	2	66.66666	0.011174	[Top1, Top2b]
GO:0004813	alanine-tRNA ligase activity	2	66.66666	0.011174	[Aars, Aars2]
GO:0004829	threonine-tRNA ligase activity	2	66.66666	0.011174	[Tars2, Tarsl2]
GO:0005078	MAP-kinase scaffold activity	2	66.66666	0.011174	[Mapk8ip3, Sh3rf1]
GO:0008538	proteasome activator activity	2	66.66666	0.011174	[Psme1, Psme2]
GO:0016972	thiol oxidase activity	2	66.66666	0.011174	[Gfer, Qsox2]
GO:0017112	Rab guanyl-nucleotide exchange factor activity	2	66.66666	0.011174	[Madd, Rab3il1]
GO:0019237	centromeric DNA binding	2	66.66666	0.011174	[Mlh3, Msh2]
GO:0048365	Rac GTPase binding	2	66.66666	0.011174	[Dock7, Ncf2]
GO:0003743	translation initiation factor activity	10	14.28571	0.011175	[Eif2s1, Eif2s3x, Eif2s3y, Eif3m, Eif4a3, Eif4b, Eif4e, Eif4e2, Eif4g1, Eif6]
GO:0019905	syntaxin binding	4	26.66667	0.011796	[Napa, Napb, Stxbp1, Stxbp2]
GO:0004497	monooxygenase activity	15	12.19512	0.012859	[Cyp1a1, Cyp1a2, Cyp27a1, Cyp2a5, Cyp2c38, Cyp2c39, Cyp2c44, Cyp2c54, Cyp2j5, Cyp2j9, Cyp4b1, Dbh, Fmo4, Ptgis, Tbxas1]
GO:0004629	phospholipase C activity	4	25	0.014966	[Plcb2, Plce1, Plch2, Plcl1]
GO:0008237	metallopeptidase activity	19	10.98266	0.016289	[Ace, Adam26a, Adam30, Adamts2, Aebp1, Afg3l1, Agbl1, Dpep2, Ece2, Folh1, Ide, Mmp2, Mmp20, Naalad2, Pa2g4, Papln, Pepd, Pmpca, Stambp]
GO:0000149	SNARE binding	4	23.52941	0.018624	[Napa, Napb, Stxbp1, Stxbp2]
GO:0005496	steroid binding	7	15.55556	0.020434	[Esr2, Nr1h3, Nr3c1, Nr3c2, Paqr8, Pgr, Pgrmc1]
GO:0008253	5'-nucleotidase activity	3	30	0.020848	[Nt5c, Nt5c2, Nt5c3]
GO:0019203	carbohydrate phosphatase activity	3	30	0.020848	[G6pc, Khsrp, Pfkfb3]
GO:0031625	ubiquitin protein ligase	3	30	0.020848	[Cul1, Cul2, Cul5]

	binding				
GO:0003917	DNA topoisomerase type I activity	2	50	0.021425	[Top1, Top3a]
GO:0004645	phosphorylase activity	2	50	0.021425	[Pygb, Pygl]
GO:0015016	[heparan sulfate]-glucosamine N-sulfotransferase activity	2	50	0.021425	[Ndst2, Ndst3]
GO:0050811	GABA receptor binding	2	50	0.021425	[Jakmip1, Plcl1]
GO:0000287	magnesium ion binding	34	9.16442	0.022492	[Acsl4, Adcy1, Adcy6, Atp10b, Atp2a1, Atp2b2, Atp2c1, Atp8a1, Ercc2, Grin1, Ick, Impa2, Irak4, Itga1, Itpa, Kalrn, Map3k13, Map3k15, Map3k2, Mus81, Nek5, Nme3, Nt5c, Nt5c2, Nt5c3, Pde5a, Pgm3, Polg, Polr2b, Ppef1, Ppm1a, Ppm1d, Rps6ka5, Tesk2]
GO:0004702	receptor signaling protein serine/threonine kinase activity	7	15.21739	0.022847	[Map3k13, Map3k14, Map3k15, Map3k2, Map3k9, Mapk11, Mapk13]
GO:0035091	phosphoinositide binding	8	14.03509	0.024313	[Exoc8, Nisch, Sbf2, Sh3pxd2a, Snx2, Snx30, Snx5, Vnn1]
GO:0070011	peptidase activity, acting on L-amino acid peptides	48	8.633094	0.025315	[Ace, Adam26a, Adam30, Adamts2, Aebp1, Afg3l1, Agbl1, Capn3, Cfb, Ctsw, Dpep2, Dpp4, Dpp7, Dpp8, Dub1, Dub2a, EG317677, Ece2, F10, F7, Folh1, Ide, Lonrf3, Mmp2, Mmp20, Naalad2, Npepl1, Pa2g4, Papln, Pepd, Pmpca, Psma1, Psmd14, Rbp3, Ren1, Senp6, Stambp, Tcfe2a, Tmprss12, Tmprss7, Tpp1, Uchl3, Usp14, Usp16, Usp26, Usp42, Usp47, Usp54]
GO:0019899	enzyme binding	15	11.45038	0.026605	[2610204M08Rik, Bank1, Cul1, Cul2, Cul5, Dock6, Dock7, Exoc8, Fmnl3, Irs2, Mapk8ip3, Micalcl, Ncf2, Sbf2, Sorbs1]
GO:0017048	Rho GTPase binding	4	21.05263	0.027478	[2610204M08Rik, Dock7, Fmnl3, Ncf2]
GO:0004527	exonuclease activity	7	14.58333	0.028247	[Aptx, Dis3, Dis3l, Polg, Rexo1, Tcfe2a, Wrn]
GO:0030145	manganese ion binding	15	11.02941	0.030273	[Cdipt, Extl3, Fyn, Galnt3, Galnt5, Npepl1, Pck2, Pepd, Pik3c3, Ppef1, Ppm1a, Ppm1d, Ppp1ca, Ppp1cc, Tesk2]
GO:0005543	phospholipid binding	11	11.95652	0.030405	[Dgka, Exoc8, Nisch, Sbf2, Sh3pxd2a, Snx2, Snx30, Snx5, Syt1, Syt2, Vnn1]
GO:0005089	Rho guanyl-nucleotide exchange factor activity	8	13.33333	0.032008	[Arhgef12, Arhgef3, Arhgef6, Fgd5, Kalrn, Ngef, Rasgrf2, Rgnef]
GO:0005523	tropomyosin binding	2	40	0.034242	[Tmod1, Tmod3]
GO:0016886	ligase activity, forming phosphoric ester bonds	2	40	0.034242	[Lig1, Lig3]
GO:0008252	nucleotidase activity	3	25	0.034824	[Nt5c, Nt5c2, Nt5c3]
GO:0019199	transmembrane receptor protein kinase activity	9	13.23529	0.037224	[Axl, Egfr, Epha7, Ephb6, Fgfr1, Fgfr2, Fgfr4, Ror1, Sltm]
GO:0004674	protein serine/threonine kinase activity	36	8.801956	0.037708	[Adrbk1, Araf, Cdk2, Cdk3, Cdk4, Cdk6, Cdkl1, Chek1, Cit, Dapk1, Grk4, Ick, Irak4, Kalrn, Ksr2, Mak, Map3k13, Map3k14, Map3k15, Map3k2, Map3k9, Map4k3, Mapk11, Mapk13, Mark4, Myo3a, Nek5, Pkn2, Prkd1, Prkg2, Ripk4, Rps6ka5, Smok3a, Tesk2, Wnk2, Wnk4]
GO:0005099	Ras GTPase activator	7	13.46154	0.041495	[Evi5, Sgsm1, Sgsm2, Tbc1d1, Tbc1d2b, Tbc1d9b, Usp6nl]

	activity				
GO:0015923	mannosidase activity	3	23.07692	0.043223	[Man1b1, Man2a1, Man2b1]
GO:0016705	oxidoreductase activity, acting on paired donors, with incorporation or reduction of molecular oxygen	14	10.68702	0.044036	[Cyp1a1, Cyp1a2, Cyp27a1, Cyp2a5, Cyp2c38, Cyp2c39, Cyp2c44, Cyp2c54, Cyp2j5, Cyp4b1, Dbh, Fmo4, Plod3, Tbxas1]
GO:0004620	phospholipase activity	7	13.20755	0.045343	[Pla2g2e, Pla2g4d, Plcb2, Plce1, Plch2, Plcl1, Smpdl3a]
GO:0008235	metalloexopeptidase activity	5	15.625	0.046312	[Aebp1, Agbl1, Dpep2, Pa2g4, Pepd]
GO:0004703	G-protein coupled receptor kinase activity	2	33.33333	0.049266	[Adrbk1, Grk4]
GO:0016670	oxidoreductase activity, acting on sulfur group of donors, oxygen as acceptor	2	33.33333	0.049266	[Gfer, Qsox2]
GO:0017154	semaphorin receptor activity	2	33.33333	0.049266	[Plxna1, Plxna4]
GO:0017169	CDP-alcohol phosphatidyltransferase activity	2	33.33333	0.049266	[Cdipt, Pgs1]
GO:0030374	ligand-dependent nuclear receptor transcription coactivator activity	2	33.33333	0.049266	[Ncoa2, Pus1]
GO:0043499	eukaryotic cell surface binding	2	33.33333	0.049266	[Cfh, Svs2]
GO:0008233	peptidase activity	50	8.196721	0.049405	[Ace, Adam26a, Adam30, Adamts2, Aebp1, Afg3l1, Agbl1, Capn3, Cfb, Cts8, Ctsw, Dpep2, Dpp4, Dpp7, Dpp8, Dub1, Dub2a, EG317677, Ece2, F10, F7, Folh1, Fstl4, Ide, Lonrf3, Mmp2, Mmp20, Naalad2, Npepl1, Pa2g4, Papln, Pepd, Pmpca, Psma1, Psmd14, Rbp3, Ren1, Senp6, Stambp, Tcfe2a, Tmprss12, Tmprss7, Tpp1, Uchl3, Usp14, Usp16, Usp26, Usp42, Usp47, Usp54]
KEGG –up-regulated dataset					
KEGG:04520	Adherens junction	14	18.66667	1.94E-04	[Ctnna1, Ctnna2, Egfr, Fert2, Fgfr1, Fyn, Ptprb, Ptprf, Ptprj, Pvrl2, Sorbs1, Tjp1, Wasf1, Yes1]
KEGG:04720	Long-term potentiation	12	17.3913	0.001054	[Adcy1, Araf, Gria2, Grin1, Grm1, Hras1, Kras, Plcb2, Ppp1ca, Ppp1cc, Ppp1r12a, Ppp3cb]
KEGG:04910	Insulin signaling pathway	19	13.57143	0.001235	[Araf, Eif4e, Eif4e2, Flot1, G6pc, Hras1, Irs2, Kras, Lipe, Pck2, Ppp1ca, Ppp1cc, Ppp1r3a, Ptprf, Pygb, Pygl, Shc4, Socs3, Sorbs1]
KEGG:04360	Axon guidance	18	13.74046	0.001503	[Arhgef12, Epha7, Ephb6, Fyn, Hras1, Kras, Ngef, Plxna1, Plxna4, Plxnb3, Plxnc1, Ppp3cb, Sema3a, Sema3b, Sema3c, Sema6a, Slit3, Unc5d]
KEGG:02010	ABC transporters	9	20	0.001594	[Abca2, Abca8a, Abcb10, Abcb11, Abcb7, Abcb8, Abcc3, Abcc4, Abcg5]
KEGG:00591	Linoleic acid metabolism	9	18.36735	0.002955	[Cyp1a2, Cyp2c38, Cyp2c39, Cyp2c44, Cyp2c54, Cyp2j5, Cyp2j9, Pla2g2e, Pla2g4d]

KEGG:05219	Bladder cancer	8	18.60465	0.004583	[Araf, Cdk4, Dapk1, Egfr, Hras1, Kras, Mmp2, Rps6ka5]
KEGG:04380	Osteoclast differentiation	15	12.29508	0.01238	[Fhl2, Fyn, Lck, Map3k14, Mapk11, Mapk13, Ncf2, Pparg, Ppp3cb, Relb, Socs3, Tec, Tgfb1, Tnfrsf11b, Tnfrsf1a]
KEGG:04730	Long-term depression	9	14.51613	0.01419	[Araf, Gria2, Grm1, Hras1, Kras, Lyn, Pla2g4d, Plcb2, Prkg2]
KEGG:05150	Staphylococcus aureus infection	8	15.38462	0.014529	[C3ar1, Cfb, Cfh, Dsg1a, Dsg1b, EG317677, Fgg, Itgam]
KEGG:00590	Arachidonic acid metabolism	12	12.5	0.018125	[Cbr2, Cyp2c38, Cyp2c39, Cyp2c44, Cyp2c54, Cyp2j5, Cyp2j9, Ggt5, Pla2g2e, Pla2g4d, Ptgis, Tbxas1]
KEGG:03050	Proteasome	7	15.90909	0.018205	[Psma1, Psmd1, Psmd14, Psmd6, Psmd8, Psme1, Psme2]
KEGG:03015	mRNA surveillance pathway	12	12.2449	0.020483	[Cpsf2, Cpsf6, Dazap1, Eif4a3, Fip1l1, Gspt1, Papolg, Ppp1ca, Ppp1cc, Ppp2r5c, Rngtt, Wibg]
KEGG:04810	Regulation of actin cytoskeleton	22	10.13825	0.022959	[Apc2, Araf, Arhgef12, Arhgef6, Cyfip2, Egfr, Enah, Fgfr1, Fgfr2, Fgfr4, Hras1, Itga1, Itgam, Kras, Nckap1, Ppp1ca, Ppp1cc, Ppp1r12a, Pxn, Scin, Ssh2, Wasf1]
KEGG:04912	GnRH signaling pathway	11	12.35955	0.0255	[Adcy1, Adcy6, Egfr, Hras1, Kras, Map3k2, Mapk11, Mapk13, Mmp2, Pla2g4d, Plcb2]
KEGG:05200	Pathways in cancer	30	9.287926	0.026992	[Apc2, Araf, Birc3, Cdk2, Cdk4, Cdk6, Col4a1, Csf2ra, Ctnna1, Ctnna2, Cul2, Dapk1, Egfr, Fgfr1, Fgfr2, Gli2, Gli3, Hras1, Kras, Lamb2, Lamc1, Lamc3, Mmp2, Msh2, Pparg, Rala, Ralb, Stat5b, Tgfb1, Wnt6]
KEGG:05213	Endometrial cancer	7	13.46154	0.041495	[Apc2, Araf, Ctnna1, Ctnna2, Egfr, Hras1, Kras]
KEGG:05212	Pancreatic cancer	9	12.67606	0.042034	[Araf, Arhgef6, Cdk4, Cdk6, Egfr, Kras, Rala, Ralb, Tgfb1]
KEGG:04320	Dorso-ventral axis formation	4	18.18182	0.044761	[Egfr, Fmn2, Kras, Notch2]
REACTOME_BioCarta –up-regulated dataset					
BioCarta:361	Roles of ß-arrestin-dependent Recruitment of Src Kinases in GPCR Signaling	5	29.41176	0.003076	[Adrbk1, Fgr, Hck, Hras1, Kcna1]
BioCarta:415	Regulation of eIF2	3	30	0.020848	[Eif2s1, Eif2s3x, Ppp1ca]
BioCarta:569	Lck and Fyn tyrosine kinases in initiation of TCR Activation	3	30	0.020848	[Fyn, Lck, Tcra]
BioCarta:377	CBL mediated ligand-induced downregulation of EGF receptors	3	27.27273	0.02736	[Egfr, Sh3kbp1, Sltm]
BioCarta:546	RB Tumor Suppressor/Checkpoint Signaling in response to DNA damage	3	27.27273	0.02736	[Cdk2, Cdk4, Chek1]
BioCarta:456	Skeletal muscle hypertrophy is regulated via AKT/mTOR pathway	4	21.05263	0.027478	[Eif2s1, Eif2s3x, Eif4e, Pdk2]

BioCarta:497	Downregulated of MTA-3 in ER-negative Breast Tumors	3	25	0.034824	[Mbd3, Mta1, Mta3]
BioCarta:495	Multi-Drug Resistance Factors	2	33.33333	0.049266	[Abcb11, Abcc3]
GO_MolecularFunction –downup-regulated dataset					
GO:0005086	ARF guanyl-nucleotide exchange factor activity	2	11.76471	2.94E-04	[Arfgef1, Arfgef2]
GO:0017022	myosin binding	2	8.695652	5.45E-04	[Arfgef1, Arfgef2]
	GO_BiologicalProcess				
GO:0032011	ARF protein signal transduction	2	5.405406	0.001415	[Arfgef1, Arfgef2]
GO:0032012	regulation of ARF protein signal transduction	2	5.405406	0.001415	[Arfgef1, Arfgef2]
REACTOME_BioCarta –down-regulated dataset					
REACTOME:2522839	Alpha-defensins	2	7.407407	7.53E-04	[Prss1, Prss2]
REACTOME:2522840	Defensins	2	5.405406	0.001415	[Prss1, Prss2]
REACTOME:2522859	Activation of Matrix Metalloproteinases	2	4.166667	0.002372	[Prss1, Prss2]
REACTOME:2522860	Degradation of the extracellular matrix	2	4.166667	0.002372	[Prss1, Prss2]

GO analysis using FuncAssociate

FuncAssociate (http://llama.mshri.on.ca/funcassociate_client/html/) uses a different strategy to derive an over-representation matrix of GO terms that is driven by a pre-mapped namespace linked to the GO-terms, which is used to map the input-query. Table E2 summarises the results using this approach with all up-regulated proteins as input. The down-regulated proteisn showed no significant GO-term enrichment.

Table E2. FuncAssociate output using all significant AKI up-regulated protein entries. Over-represented GO terms (ID and name) are listed by their statistical significance values (p-values, unadjusted and adjusted, and LOD value) and the number of proteins from the query list associated with the GO term.

N<br>number of entities in query having the row's attribute	LOD<br>logarithm (base 10) of the odds ratio	P<br>p-value	P_adj<br>resampling-adjusted p-value	attribute ID	attribute name
6	1.639393	8.16E-07	0.002	GO:0005980	glycogen catabolic process
6	1.639393	8.16E-07	0.002	GO:0009251	glucan catabolic process
6	1.639393	8.16E-07	0.002	GO:0044247	cellular polysaccharide catabolic process
14	0.71073	5.82E-06	0.019	GO:0015980	energy derivation by oxidation of organic compounds
17	0.680888	1.30E-06	0.005	GO:0008026	ATP-dependent helicase activity
17	0.680888	1.30E-06	0.005	GO:0070035	purine NTP-dependent helicase activity
25	0.591483	1.37E-07	0.001	GO:0004386	helicase activity
40	0.501017	5.65E-09	<0.001	GO:0016887	ATPase activity
30	0.468688	1.66E-06	0.005	GO:0042623	ATPase activity, coupled
32	0.449658	1.75E-06	0.005	GO:0030036	actin cytoskeleton organization
34	0.445923	9.99E-07	0.003	GO:0030029	actin filament-based process
39	0.407344	1.20E-06	0.005	GO:0003779	actin binding
66	0.396279	8.99E-10	<0.001	GO:0008092	cytoskeletal protein binding
169	0.388358	5.30E-21	<0.001	GO:0005524	ATP binding

170	0.383225	1.16E-20	<0.001	GO:0032559	adenyl ribonucleotide binding
170	0.380696	1.92E-20	<0.001	GO:0030554	adenyl nucleotide binding
38	0.373069	8.38E-06	0.02	GO:0051301	cell division
65	0.357677	2.49E-08	<0.001	GO:0004672	protein kinase activity
191	0.356392	2.92E-20	<0.001	GO:0035639	purine ribonucleoside triphosphate binding
193	0.352544	4.44E-20	<0.001	GO:0032555	purine ribonucleotide binding
193	0.352244	4.73E-20	<0.001	GO:0032553	ribonucleotide binding
57	0.352018	2.44E-07	0.001	GO:0043005	neuron projection
193	0.350452	6.92E-20	<0.001	GO:0017076	purine nucleotide binding
67	0.347696	3.38E-08	<0.001	GO:0017111	nucleoside-triphosphatase activity
50	0.346045	1.79E-06	0.005	GO:0007010	cytoskeleton organization
82	0.343006	1.85E-09	<0.001	GO:0006468	protein phosphorylation
69	0.337696	4.74E-08	<0.001	GO:0016462	pyrophosphatase activity
69	0.335267	5.73E-08	<0.001	GO:0016818	hydrolase activity, acting on acid anhydrides, in phosphorus-containing anhydrides
69	0.33446	6.10E-08	<0.001	GO:0016817	hydrolase activity, acting on acid anhydrides
70	0.324362	1.09E-07	<0.001	GO:0016788	hydrolase activity, acting on ester bonds
223	0.313769	1.13E-18	<0.001	GO:0000166	nucleotide binding
104	0.313582	4.89E-10	<0.001	GO:0042995	cell projection
837	0.312801	1.84E-39	<0.001	GO:0005488	binding
115	0.302009	2.77E-10	<0.001	GO:0006796	phosphate metabolic process
115	0.301105	3.08E-10	<0.001	GO:0006793	phosphorus metabolic process
69	0.301048	7.57E-07	0.002	GO:0016773	phosphotransferase activity, alcohol group as acceptor
66	0.299269	1.45E-06	0.005	GO:0030054	cell junction
206	0.292642	1.22E-15	<0.001	GO:0016787	hydrolase activity
93	0.283156	6.68E-08	<0.001	GO:0016310	phosphorylation
1012	0.273967	8.32E-28	<0.001	GO:0003674	molecular_function
76	0.269179	2.62E-06	0.008	GO:0016301	kinase activity
235	0.264886	9.69E-15	<0.001	GO:0019538	protein metabolic process
68	0.263297	1.22E-05	0.038	GO:0003723	RNA binding
153	0.26105	2.56E-10	<0.001	GO:0006464	protein modification process
108	0.257403	1.02E-07	<0.001	GO:0000267	cell fraction
186	0.256765	1.05E-11	<0.001	GO:0044267	cellular protein metabolic process

463	0.255917	2.23E-22	<0.001	GO:0005515	protein binding
91	0.255742	1.02E-06	0.003	GO:0005856	cytoskeleton
158	0.254901	3.38E-10	<0.001	GO:0043412	macromolecule modification
85	0.254757	2.37E-06	0.008	GO:0016772	transferase activity, transferring phosphorus-containing groups
78	0.252695	6.76E-06	0.019	GO:0005624	membrane fraction
81	0.246078	7.59E-06	0.02	GO:0005626	insoluble fraction
418	0.246062	1.23E-19	<0.001	GO:0003824	catalytic activity
295	0.244038	2.91E-15	<0.001	GO:0043167	ion binding
293	0.241938	5.69E-15	<0.001	GO:0043169	cation binding
289	0.239981	1.26E-14	<0.001	GO:0046872	metal ion binding
1049	0.231119	2.75E-19	<0.001	GO:0005575	cellular_component
1005	0.230194	3.88E-20	<0.001	GO:0044464	cell part
401	0.225271	1.91E-16	<0.001	GO:0043170	macromolecule metabolic process
362	0.221283	6.15E-15	<0.001	GO:0044260	cellular macromolecule metabolic process
168	0.219691	1.64E-08	<0.001	GO:0071842	cellular component organization at cellular level
759	0.219282	1.79E-20	<0.001	GO:0044424	intracellular part
382	0.219077	3.28E-15	<0.001	GO:0005737	cytoplasm
171	0.213574	2.90E-08	<0.001	GO:0071841	cellular component organization or biogenesis at cellular level
481	0.209052	4.24E-16	<0.001	GO:0044238	primary metabolic process
133	0.20763	1.32E-06	0.005	GO:0046914	transition metal ion binding
211	0.206609	3.71E-09	<0.001	GO:0016043	cellular component organization
115	0.206408	6.63E-06	0.019	GO:0006996	organelle organization
951	0.202199	1.27E-16	<0.001	GO:0008150	biological_process
214	0.202124	6.07E-09	<0.001	GO:0071840	cellular component organization or biogenesis
628	0.19772	2.03E-16	<0.001	GO:0043229	intracellular organelle
628	0.196324	3.24E-16	<0.001	GO:0043226	organelle
542	0.1953	3.45E-15	<0.001	GO:0008152	metabolic process
467	0.191632	1.15E-13	<0.001	GO:0044237	cellular metabolic process
136	0.186432	9.14E-06	0.022	GO:0016740	transferase activity
156	0.183207	3.48E-06	0.011	GO:0043228	non-membrane-bounded organelle
156	0.183207	3.48E-06	0.011	GO:0043232	intracellular non-membrane-bounded organelle
205	0.17786	3.74E-07	0.002	GO:0005886	plasma membrane

348	0.17049	1.66E-09	<0.001	GO:0005634	nucleus
552	0.168001	7.48E-12	<0.001	GO:0043227	membrane-bounded organelle
550	0.16569	1.45E-11	<0.001	GO:0043231	intracellular membrane-bounded organelle
209	0.165323	1.64E-06	0.005	GO:0043234	protein complex
789	0.159647	1.09E-11	<0.001	GO:0009987	cellular process
223	0.156498	2.80E-06	0.009	GO:0032502	developmental process
363	0.140177	3.41E-07	0.001	GO:0044444	cytoplasmic part
290	0.137436	3.91E-06	0.011	GO:0019222	regulation of metabolic process
424	0.123113	1.99E-06	0.006	GO:0016020	membrane
554	0.10645	8.54E-06	0.02	GO:0065007	biological regulation

GO analysis using GOrilla

GOrilla (http://cbl-gorilla.cs.technion.ac.il/) uses yet again a different methodology to map GO-terms to user input list, where the input is sorted according to expression levels and a weighed matrix/mHG statistics is applied favouring entries which are higher up (i.e. show bigger expression differences in AKI compared to control) in the list. Table E3 summarises the result of the GO enrichment analysis using GOrilla.

Table E3. Over-representation of GO terms using GOrilla. The enrtiched GO term and its descriptor is shown together with the associated proteins found in the input list. The P-value is the enrichment p-value computed according to the mHG or HG model, and the FDR q-value is the correction of the p-value for multiple testing using the Benjamini and Hochberg method. Specifically, the FDR q-value was computed for the n-th term (ranked according to p-value) as p-value * number of GO terms) / n.

GO Term	Description	P-value	FDR q-value	Molecules
Biological Process				
GO:1900373	positive regulation of purine nucleotide biosynthetic process	6.10E-05	3.01E-01	[Adcyap1r1, Adcy1, Adcy6]
GO:1900371	regulation of purine nucleotide biosynthetic process	6.10E-05	1.51E-01	[Adcyap1r1, Adcy1, Adcy6]
GO:0045981	positive regulation of nucleotide metabolic process	6.10E-05	1.00E-01	[Adcyap1r1, Adcy1, Adcy6]

GO:1900544	positive regulation of purine nucleotide metabolic process	6.10E-05	7.53E-02	[Adcyap1r1, Adcy1, Adcy6]
GO:0030808	regulation of nucleotide biosynthetic process	6.10E-05	6.03E-02	[Adcyap1r1, Adcy1, Adcy6]
GO:0030802	regulation of cyclic nucleotide biosynthetic process	6.10E-05	5.02E-02	[Adcyap1r1, Adcy1, Adcy6]
GO:0030801	positive regulation of cyclic nucleotide metabolic process	6.10E-05	4.30E-02	[Adcyap1r1, Adcy1, Adcy6]
GO:0030804	positive regulation of cyclic nucleotide biosynthetic process	6.10E-05	3.77E-02	[Adcyap1r1, Adcy1, Adcy6]
GO:0030819	positive regulation of cAMP biosynthetic process	6.10E-05	3.35E-02	[Adcyap1r1, Adcy1, Adcy6]
GO:0030817	regulation of cAMP biosynthetic process	6.10E-05	3.01E-02	[Adcyap1r1, Adcy1, Adcy6]
GO:0030816	positive regulation of cAMP metabolic process	6.10E-05	2.74E-02	[Adcyap1r1, Adcy1, Adcy6]
GO:0030814	regulation of cAMP metabolic process	6.10E-05	2.51E-02	[Adcyap1r1, Adcy1, Adcy6]
GO:0030810	positive regulation of nucleotide biosynthetic process	6.10E-05	2.32E-02	[Adcyap1r1, Adcy1, Adcy6]
GO:0030799	regulation of cyclic nucleotide metabolic process	6.10E-05	2.15E-02	[Adcyap1r1, Adcy1, Adcy6]
GO:0032368	regulation of lipid transport	2.52E-04	8.29E-02	[Apoa4, Abca2, Acsl4, Abcg5]
GO:0015721	bile acid and bile salt transport	3.59E-04	1.11E-01	[Abcb11, Abcc3]
GO:0015722	canalicular bile acid transport	3.59E-04	1.04E-01	[Abcb11, Abcc3]
GO:0006855	drug transmembrane transport	3.87E-04	1.06E-01	[Abcc3, Abcc4]
GO:0009152	purine ribonucleotide biosynthetic process	8.21E-04	2.13E-01	[Adsl, Adcy1, Atic, Adcy6]
GO:0032371	regulation of sterol transport	9.90E-04	2.45E-01	[Abca2, Apoa4, Abcg5]
GO:0032374	regulation of cholesterol transport	9.90E-04	2.33E-01	[Abca2, Apoa4, Abcg5]
Molecular Function				
GO:0004693	cyclin-dependent protein serine/threonine kinase activity	3.01E-05	3.83E-02	[Cdk4, Cdk18, Cdk6, Cdkl1, Cdk1, Cdk17, Cdk2]
GO:0097472	cyclin-dependent protein kinase activity	3.01E-05	1.92E-02	[Cdk18, Cdk4, Cdk6, Cdkl1, Cdk1, Cdk17, Cdk2]
GO:0005319	lipid transporter activity	6.55E-05	2.78E-02	[Abcb11, Apoa4, Abcc3, Abcg5-]
GO:0043492	ATPase activity, coupled to movement of substances	7.40E-05	2.36E-02	[Abcb8, Abcb11, Abcc3, Abcb10-]
GO:0015399	primary active transmembrane transporter activity	7.40E-05	1.88E-02	[Abcb8, Abcb11, Abcc3, Abcb10]
GO:0015405	P-P-bond-hydrolysis-driven transmembrane transporter activity	7.40E-05	1.57E-02	[Abcb8, Abcb11, Abcc3, Abcb10]
GO:0016820	hydrolase activity, acting on acid anhydrides, catalyzing transmembrane movement of substances	7.40E-05	1.35E-02	[Abcb8, Abcb11, Abcc3, Abcb10]
GO:0042626	ATPase activity, coupled to transmembrane movement of substances	7.40E-05	1.18E-02	[Abcb8, Abcb11, Abcc3, Abcb10]
GO:0008047	enzyme activator activity	2.42E-04	3.43E-02	[Arhgap31, Arap2, Apoa4, Arhgap42, Arhgef12, Arhgap22, Asap1, Ahsa1, Arhgap19]
GO:0015125	bile acid transmembrane transporter activity	3.59E-04	4.58E-02	[Abcb11, Abcc3]
GO:0015238	drug transmembrane transporter activity	3.87E-04	4.48E-02	[Abcc3, Abcc4]
GO:0090484	drug transporter activity	3.87E-04	4.11E-02	[Abcc3, Abcc4]

GO:0004888	transmembrane signaling receptor activity	6.52E-04	6.39E-02	[Hrh4, Olfr806, C130060K24Rik, Ntsr1, Tas2r136, Vmn2r96, Vmn2r29, Olfr780, Gria2, Gpr19, Lifr, Vmn2r9, Axl, Vmn2r2, Vmn2r38, Tnfrsf1a, Olfr632, Epha7, Olfr867, Tas2r143, Olfr1033, Olfr134, Grin1, Vmn2r8, Tas1r2, Olfr181, Grm1, Fgfr2, Ifnlr1, Fgfr1, Lphn1, Fgfr4, Il1rl1, Gabrd, Csf2ra, Vmn2r28, Plxna1, Adcyap1r1, Plxna4, Gabra1, Rxfp1, Ptprf, C3ar1, Vmn2r42, Frzb, Olfr642, Ccr8, Sema6a, Vmn2r116, Gpr176, Vmn2r43, Olfr1211, Vmn2r84Olfr1495, Npc1l1, Egfr, Qrfpr, Vmn2r51]
GO:0004672	protein kinase activity	7.27E-04	6.61E-02	[Jak3, Fgfr2, Araf, Fgfr1, Mak, Cdkl1, Gtf2h1, Fgr, Cdk17, Fgfr4, Kcnh8, Mapk11, Hck, Adrbk1, Cdk18, Map3k14, Ick, Fam20c, Map3k15, Axl, Fastkd2, Mark4, Ercc2, Ksr2, Map3k13, Epha7, Cdk1, Mapk13, Cdk4, Fastkd1, Fyn, Cdk6, Lyn, Map4k3, Map3k2, Lck, Irak4, Egfr, Chek1, Cdk2]
GO:0016887	ATPase activity	8.43E-04	7.16E-02	[Abcb8, Abcb11, Abca2, Abca8a, Abcc3, Abcg5, Abcb10]
GO:0052689	carboxylic ester hydrolase activity	9.73E-04	7.74E-02	[Aadacl3, Acot12, Aars, Abhd2]
Cellular Component				
GO:0031224	intrinsic to membrane	3.44E-04	2.17E-01	[Man2a1, Tex10, Abca2, Ptgis, Syt6, Slc9a6, Ntsr1, Adcy1, Vmn2r29, Olfr780, Marco, Tmem70, Extl3, Vmn2r2, Abhd2, Mcur1, Afg3l1, Cystm1, Abcb11, Slc25a35, Tas2r143, Abcg5, Cacna1g, Dbh, Olfr134, Gnptab, Cdhr5, Tmprss7, Notch2, Has2, Paqr8, Ncln, B3gnt7, Tm9sf3, Tmem173, Abcc3, Tmtc3, Il1rl1, Adcy6, Gpr111, Vmn2r28, Tbxas1, Igsf1, Adcyap1r1, Megf8, Pvrl2, Gpc6, Galnt5, Ntng2, Ptprf, Naalad2, Olfr642, Qsox2, Teddm1, Glt8d2, Shisa9, Man1b1, Ptprj, Ncr1, Olfr1495, Npc1l1, Pcnxl3, Jph3, Ptprv, Asic3, C130060K24Rik, Tas2r136, Vmn2r96, Fmo4, Gria2, Slc25a27, Lifr, Tmem126a, Atp10b, Dhcr24, Vmn2r9, Sorcs1, Ece2, Tmem201, Kcnn1, Btbd11, Car12, Aadacl3, Chrna10, Vnn1, Tnfrsf1a, Alg5, Olfr867, Tango6, Abcb10, Cd33, Grin1, Scn7a, Tas1r2, Nup210l, Gramd1b, Olfr181, Grm1, Fgfr2, Ifnlr1, Rhbdf2, Fgfr1, Comtd1, Trpm3, Acsl4, Hs6st2, Kcnh8, Fgfr4, Nckap1, Dsg4, Abca8a, Kirrel3, Syt1, H2-D1, Abcb8, Rdh10, Unc5d, Syt2, Gimap1, Sema6a, Rtn4, Vmn2r43, Vmn2r84, Pex11g, Sel1l3, Gosr1, Cers5, St6galnac6, Dsg1b, Klra7, 8430419L09Rik, Hrh4, Clcn3, Klra4, Olfr806, Nlgn2, Pcdhb17, Slc25a41, Chst10, Reep2, Ceacam2, Axl, Pkd1, Slc34a2, Mettl21a, Ceacam1, Mfap3, Olfr632, Olfr1033, Pigr, Tomm20l, Slc16a7, Vmn2r8, Dsg3, Csgalnact1, Tmco5b, Slc35a3, Dsg1a, Treh, Slc8a3, Galnt3, Sacm1l, Ust, Gabrd, Gm5134, Clec1a, Plxna1, Plxna4, Gabra1, Rxfp1, Vmn2r42, Tbc1d9b, G6pc, Mctp2, Ccr8, C2cd2l, Gpr176, Ifitm7, Abhd16a, Olfr1211, Cnih2, Ndst3, Slc27a3, Nalcn, Fam151a, Slc38a8, Slc17a8, Vmn2r51, Siglecg, Esyt1, F11r, Cldn8, D10Bwg1379e, Cd163, Gpr19, Tpo, Kcna1, Sdk1, Nphs2, Trpm1, Lrrtm3, Vmn2r38, Cox7c, Epha7, Sptlc1, Ndst2, Bambi, Srd5a1, Ppp1r3a, Lphn1, Tnfsf4, Fcrl6, Dpep2, Plxnb3, Igsf8, Csf2ra, Lsr, Tmem120a, Dpcr1, Vangl2, C3ar1, Cdipt, Vmn2r116, Slitrk2, Trpc4, Nipal4, Dnajc16, Tmprss12, Egfr, Qrfpr, Kcnh4]
GO:0016021	integral to membrane	3.70E-04	1.16E-01	[Man2a1, Tex10, Ptgis, Abca2, Syt6, Slc9a6, Ntsr1, Adcy1, Vmn2r29, Olfr780, Marco, Tmem70, Extl3, Vmn2r2, Abhd2, Mcur1, Afg3l1, Cystm1, Abcb11, Slc25a35, Tas2r143, Abcg5, Cacna1g, Dbh, Olfr134, Tmprss7, Cdhr5, Gnptab, Notch2, Has2, Ncln, Paqr8, B3gnt7, Tm9sf3, Tmem173, Abcc3, Il1rl1, Tmtc3, Adcy6, Gpr111, Tbxas1, Vmn2r28, Adcyap1r1, Igsf1, Megf8, Pvrl2, Gpc6, Galnt5, Ptprf, Naalad2, Olfr642, Qsox2, Teddm1, Glt8d2, Shisa9, Man1b1, Ptprj, Ncr1, Olfr1495, Npc1l1, Pcnxl3, Jph3, Ptprv, Asic3, C130060K24Rik, Tas2r136, Vmn2r96, Fmo4, Gria2, Slc25a27, Lifr, Tmem126a,

Atp10b, Dhcr24, Vmn2r9, Sorcs1, Ece2, Tmem201, Kcnn1, Btbd11, Car12, Aadacl3, Chrna10, Vnn1, Tnfrsf1a, Alg5, Olfr867, Tango6, Abcb10, Cd33, Grin1, Scn7a, Tas1r2, Nup210l, Gramd1b, Olfr181, Grm1, Fgfr2, Ifnlr1, Rhbdf2, Fgfr1, Comtd1, Trpm3, Acsl4, Hs6st2, Kcnh8, Fgfr4, Nckap1, Dsg4, Abca8a, Kirrel3, Syt1, H2-D1, Abcb8, Rdh10, Unc5d, Syt2, Gimap1, Sema6a, Rtn4, Vmn2r43, Vmn2r84, Pex11g, Sel1l3, Gosr1, Cers5, St6galnac6, Dsg1b, Klra7, 8430419L09Rik, Hrh4, Clcn3, Klra4, Olfr806, Nlgn2, Pcdhb17, Slc25a41, Chst10, Reep2, Ceacam2, Axl, Pkd1, Slc34a2, Mettl21a, Ceacam1, Mfap3, Olfr632, Olfr1033, Pigr, Tomm20l, Slc16a7, Vmn2r8, Dsg3, Csgalnact1, Tmco5b, bDsg1a, Slc35a3, Galnt3, Slc8a3, Sacm1l, Ust, Gabrd, Gm5134, Clec1a, Plxna1, Plxna4, Gabra1, Rxfp1, Tbc1d9b, Vmn2r42, G6pc, Mctp2, Ccr8, C2cd2l, Abhd16a, Ifitm7, Gpr176, Cnih2, Olfr1211, Ndst3, Slc27a3, Fam151a, Nalcn, Slc17a8, Slc38a8, Vmn2r51, Siglecg, Esyt1, F11r, Cldn8, D10Bwg1379e, Cd163, Gpr19, Tpo, Kcna1, Sdk1, Nphs2, Trpm1, Lrrtm3, Vmn2r38, Cox7c, Epha7, Sptlc1, Ndst2, Bambi, Srd5a1, Lphn1, Ppp1r3a, Tnfsf4, Fcrl6, Plxnb3, Csf2ra, Igsf8, Lsr, Dpcr1, Tmem120a, Vangl2, C3ar1, Cdipt, Vmn2r116, Slitrk2, Trpc4, Nipal4, Dnajc16, Tmprss12, Egfr, Qrfpr, Kcnh4]

In summary, GO analysis indicated several pathways to be enriched in the up-regulated datasets which could be of interest for further analysis, such as the TNFalpha-driven NFkappaB-interleukin pathway, JAK-STAT signalling, MAPK/kinase signalling, and calcium-mediated pathways. There are also indications that the immune-response system is up-regulated, and also that focal adhesions and/or cytoskeletal changes are initiated through actin-bundling. Metabolic pathways which appear to be up-regulated in AKI involve carbohydrate, lipid and glutamate metabolic processes. Biological processes which are up-regulated in AKI include protein processing and translation and differentiation. The cellular component analysis shows that cytoskeletal changes through actin modulation, focal adhesion and cell junction are up-regulated in the AKI samples.

The GO analysis for the molecular function also showed similar results to the biological processes, with the addition of an involvement of inositol/phosphatidyl-inositol pathways, AKI-elevated guanylate exchange factor activity and calcium ATPase modulation. Using keyword-clustering for KEGG terms supports some of the previous findings stated above, and Reactome group-clustering mainly showed an up-regulation of processes involving Rho/Ras/Rac.

Interactome analysis

Another approach to analyse the data is the interactome analysis, where the significant datasets are explored for protein-protein interactions, common MESH term linkages and associations such as co-regulations, etc. amongst themselves as well as through bridging molecules. These molecular clusters are then examined for functional groups, since a common feature of clustered molecules is a shared biological function as well as assembly of signalling cascades. This method has the advantage that it is not based on prior functional tagging such as GO analysis or known pathway maps and signalling cascades, however, it is a requirement that protein-protein interactions for the molecules in question have been demonstrated. The benefit of this type of analysis is also that any cluster of interest can easily be explored further by analysing the specific proteins contained therein.

Molecular interaction networks were assembled using the CytoScape MiMI plug-in (mimi.ncibi.org). All 1480 entries were annotated with their respective gene names in order to enable searching. Full interactome analysis was done by connecting molecules through neighbouring proteins (Figure E3), whereas the condensed analysis was performed by searching for direct associations, followed by stepwise expansion to tie-in other molecular clusters (Figure E4). Simultaneously, the interactome clusters were also analysed by mapping them back onto GO terms (see Tables E4 and E5).

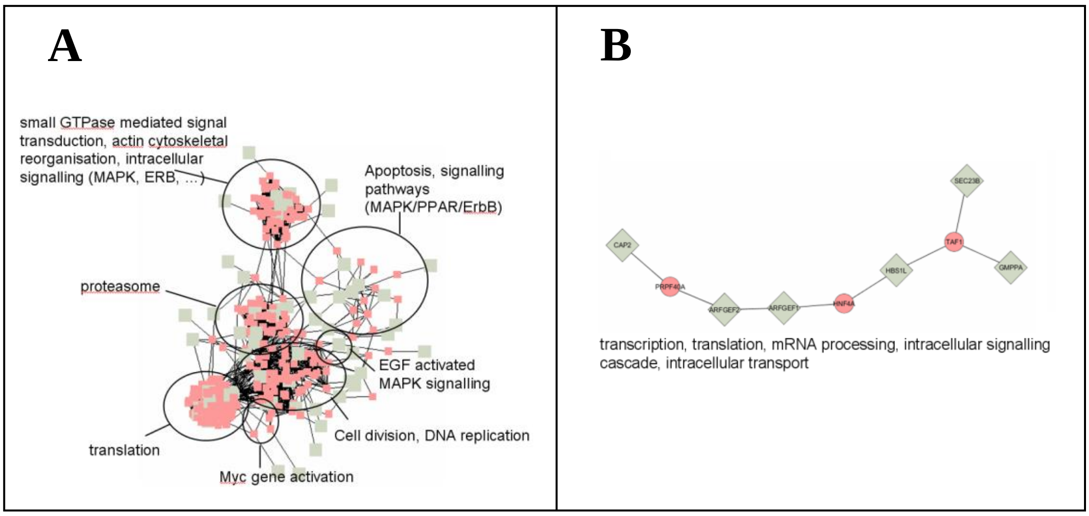

Figure E3. Protein interaction clusters. Protein-protein interactions were mapped using MiMI and allowing for additional shared proteins (red circles) which were not in the query list (grey diamonds) as bridging proteins in order to inter-connect clusters. (A) shows the modular clusters of the up-regulated proteins in AKI, and (B)

the corresponding main cluster in the down-regulated AKI dataset. Specific functional clusters are highlighted and labelled according to their main functional association.

Table E4. GO term associations of protein interaction clusters. The GO analysis of all clustered molecules is shown for both up- and down-regulated sub-groups. The LOD value is the logarithm (base 10) of the odds ratio, and statistical significance for each GO term (listed by attribute ID and name) is provided by the p-value, and the resampling adjusted p-value.

LOD	P	P_adj	attrib ID	attrib name
up-regulated in AKI				
2.469382	5.78E-09	<0.001	GO:0042555	MCM complex
2.396424	1.37E-07	0.001	GO:0000275	mitochondrial proton-transporting ATP synthase complex, catalytic core F(1)
2.396424	1.37E-07	0.001	GO:0045261	proton-transporting ATP synthase complex, catalytic core F(1)
2.308866	3.23E-06	0.01	GO:0005750	mitochondrial respiratory chain complex III
2.308866	3.23E-06	0.01	GO:0045275	respiratory chain complex III
2.056827	2.72E-15	<0.001	GO:0005753	mitochondrial proton-transporting ATP synthase complex
2.056827	2.72E-15	<0.001	GO:0045259	proton-transporting ATP synthase complex
2.005336	1.73E-18	<0.001	GO:0005839	proteasome core complex
1.992243	3.90E-08	0.001	GO:0000276	mitochondrial proton-transporting ATP synthase complex, coupling factor F(o)
1.992243	3.90E-08	0.001	GO:0019773	proteasome core complex, alpha-subunit complex
1.992243	3.90E-08	0.001	GO:0022625	cytosolic large ribosomal subunit
1.919284	7.92E-07	0.002	GO:0005092	GDP-dissociation inhibitor activity
1.919284	7.92E-07	0.002	GO:0006290	pyrimidine dimer repair
1.911734	5.09E-30	<0.001	GO:0022627	cytosolic small ribosomal subunit
1.910681	1.31E-14	<0.001	GO:0015985	energy coupled proton transport, down electrochemical gradient
1.910681	1.31E-14	<0.001	GO:0015986	ATP synthesis coupled proton transport
1.887698	4.29E-10	<0.001	GO:0005675	holo TFIIH complex
1.887698	4.29E-10	<0.001	GO:0009188	ribonucleoside diphosphate biosynthetic process
1.832932	8.15E-09	0.001	GO:0009136	purine nucleoside diphosphate biosynthetic process
1.832932	8.15E-09	0.001	GO:0009180	purine ribonucleoside diphosphate biosynthetic process
1.790266	8.51E-11	<0.001	GO:0005665	DNA-directed RNA polymerase II, core complex
1.770376	1.50E-07	0.001	GO:0005980	glycogen catabolic process
1.770376	1.50E-07	0.001	GO:0006172	ADP biosynthetic process
1.770376	1.50E-07	0.001	GO:0009251	glucan catabolic process
1.770376	1.50E-07	0.001	GO:0044247	cellular polysaccharide catabolic process
1.764	5.87E-18	<0.001	GO:0004298	threonine-type endopeptidase activity
1.764	5.87E-18	<0.001	GO:0070003	threonine-type peptidase activity
1.697418	2.67E-06	0.004	GO:0000109	nucleotide-excision repair complex
1.697418	2.67E-06	0.004	GO:0006283	transcription-coupled nucleotide-excision repair
1.697418	2.67E-06	0.004	GO:0031145	anaphase-promoting complex-dependent proteasomal ubiquitin-dependent protein catabolic process
1.697418	2.67E-06	0.004	GO:0042273	ribosomal large subunit biogenesis
1.697418	2.67E-06	0.004	GO:0044325	ion channel binding

1.686786	2.62E-08	0.001	GO:0043560	insulin receptor substrate binding
1.686786	2.62E-08	0.001	GO:0046031	ADP metabolic process
1.681103	2.66E-10	<0.001	GO:0005852	eukaryotic translation initiation factor 3 complex
1.642041	3.15E-38	<0.001	GO:0000502	proteasome complex
1.641782	4.74E-13	<0.001	GO:0005104	fibroblast growth factor receptor binding
1.62423	4.35E-07	0.002	GO:0005849	mRNA cleavage factor complex
1.577624	6.93E-08	0.001	GO:0045263	proton-transporting ATP synthase complex, coupling factor F(o)
1.565242	1.16E-10	<0.001	GO:0005669	transcription factor TFIID complex
1.565242	1.16E-10	<0.001	GO:0005680	anaphase-promoting complex
1.545222	1.09E-08	0.001	GO:0009133	nucleoside diphosphate biosynthetic process
1.545222	1.09E-08	0.001	GO:0009135	purine nucleoside diphosphate metabolic process
1.545222	1.09E-08	0.001	GO:0009179	purine ribonucleoside diphosphate metabolic process
1.515068	1.05E-06	0.002	GO:0046933	hydrogen ion transporting ATP synthase activity, rotational mechanism
1.442109	1.49E-05	0.041	GO:0016303	1-phosphatidylinositol-3-kinase activity
1.442109	1.49E-05	0.041	GO:0034332	adherens junction organization
1.442109	1.49E-05	0.041	GO:0035004	phosphatidylinositol 3-kinase activity
1.442109	1.49E-05	0.041	GO:0071775	regulation of cell cycle cytokinesis
1.442109	1.49E-05	0.041	GO:0071777	positive regulation of cell cycle cytokinesis
1.433434	1.41E-11	<0.001	GO:0004129	cytochrome-c oxidase activity
1.433434	1.41E-11	<0.001	GO:0015002	heme-copper terminal oxidase activity
1.433434	1.41E-11	<0.001	GO:0016676	oxidoreductase activity, acting on a heme group of donors, oxygen as acceptor
1.427899	2.22E-06	0.003	GO:0006270	DNA-dependent DNA replication initiation
1.427899	2.22E-06	0.003	GO:0032467	positive regulation of cytokinesis
1.427899	2.22E-06	0.003	GO:0042274	ribosomal small subunit biogenesis
1.427742	2.19E-12	<0.001	GO:0005689	U12-type spliceosomal complex
1.427742	2.19E-12	<0.001	GO:0070979	protein K11-linked ubiquitination
1.409762	2.02E-16	<0.001	GO:0008543	fibroblast growth factor receptor signaling pathway
1.393908	2.83E-11	<0.001	GO:0016675	oxidoreductase activity, acting on a heme group of donors
1.363208	2.72E-22	<0.001	GO:0015935	small ribosomal subunit
1.356894	2.29E-09	<0.001	GO:0000152	nuclear ubiquitin ligase complex
1.356502	1.49E-08	0.001	GO:0000428	DNA-directed RNA polymerase complex
1.356502	1.49E-08	0.001	GO:0009185	ribonucleoside diphosphate metabolic process
1.356502	1.49E-08	0.001	GO:0055029	nuclear DNA-directed RNA polymerase complex
1.355721	6.44E-07	0.002	GO:0033276	transcription factor TFTC complex
1.337157	1.44E-15	<0.001	GO:0005977	glycogen metabolic process
1.337157	1.44E-15	<0.001	GO:0006073	cellular glucan metabolic process
1.337157	1.44E-15	<0.001	GO:0044042	glucan metabolic process
1.321055	6.50E-10	<0.001	GO:0009156	ribonucleoside monophosphate biosynthetic process
1.313019	2.73E-08	0.001	GO:0030880	RNA polymerase complex
1.309526	1.07E-13	<0.001	GO:0035254	glutamate receptor binding
1.307789	1.78E-07	0.001	GO:0032465	regulation of cytokinesis
1.307789	1.78E-07	0.001	GO:0046627	negative regulation of insulin receptor signaling pathway
1.293165	7.67E-06	0.026	GO:0031369	translation initiation factor binding
1.287613	1.15E-09	<0.001	GO:0043548	phosphatidylinositol 3-kinase binding
1.264305	3.08E-07	0.002	GO:0009127	purine nucleoside monophosphate biosynthetic process
1.264305	3.08E-07	0.002	GO:0009168	purine ribonucleoside monophosphate biosynthetic process
1.264202	3.11E-10	<0.001	GO:0016469	proton-transporting two-sector ATPase complex

1.261283	1.64E-20	<0.001	GO:0006413	translational initiation
1.253022	1.99E-06	0.003	GO:0043551	regulation of phosphatidylinositol 3-kinase activity
1.245294	8.29E-11	<0.001	GO:0006367	transcription initiation from RNA polymerase II promoter
1.243819	3.75E-15	<0.001	GO:0006112	energy reserve metabolic process
1.238789	1.29E-05	0.036	GO:0043552	positive regulation of phosphatidylinositol 3-kinase activity
1.237262	8.03E-08	0.001	GO:0045309	protein phosphorylated amino acid binding
1.211459	8.58E-10	<0.001	GO:0005100	Rho GTPase activator activity
1.209539	3.26E-06	0.01	GO:0000307	cyclin-dependent protein kinase holoenzyme complex
1.200409	5.36E-09	<0.001	GO:0006289	nucleotide-excision repair
1.198261	2.22E-10	<0.001	GO:0006446	regulation of translational initiation
1.198261	2.22E-10	<0.001	GO:0009161	ribonucleoside monophosphate metabolic process
1.180465	2.62E-14	<0.001	GO:0006352	transcription initiation, DNA-dependent
1.172768	2.07E-07	0.001	GO:0030532	small nuclear ribonucleoprotein complex
1.172185	1.60E-13	<0.001	GO:0000082	G1/S transition of mitotic cell cycle
1.170012	5.14E-06	0.014	GO:0001759	organ induction
1.170012	5.14E-06	0.014	GO:0030071	regulation of mitotic metaphase/anaphase transition
1.170012	5.14E-06	0.014	GO:0043029	T cell homeostasis
1.170012	5.14E-06	0.014	GO:0071407	cellular response to organic cyclic compound
1.164427	2.16E-09	<0.001	GO:0005158	insulin receptor binding
1.159841	7.89E-30	<0.001	GO:0003735	structural constituent of ribosome
1.155107	1.28E-06	0.002	GO:0043550	regulation of lipid kinase activity
1.150685	1.31E-08	0.001	GO:0035591	signaling adaptor activity
1.14545	5.37E-21	<0.001	GO:0009260	ribonucleotide biosynthetic process
1.143786	3.18E-07	0.002	GO:0046875	ephrin receptor binding
1.142694	3.82E-13	<0.001	GO:0003743	translation initiation factor activity
1.134919	7.96E-08	0.001	GO:0009126	purine nucleoside monophosphate metabolic process
1.134919	7.96E-08	0.001	GO:0009167	purine ribonucleoside monophosphate metabolic process
1.133782	7.84E-06	0.028	GO:0016538	cyclin-dependent protein kinase regulator activity
1.133782	7.84E-06	0.028	GO:0033177	proton-transporting two-sector ATPase complex, proton-transporting domain
1.11864	6.71E-18	<0.001	GO:0009152	purine ribonucleotide biosynthetic process
1.116616	4.79E-07	0.002	GO:0046626	regulation of insulin receptor signaling pathway
1.106023	3.59E-15	<0.001	GO:0005089	Rho guanyl-nucleotide exchange factor activity
1.101895	1.29E-12	<0.001	GO:0044264	cellular polysaccharide metabolic process
1.10034	1.16E-05	0.035	GO:0007259	JAK-STAT cascade
1.10034	1.16E-05	0.035	GO:0035255	ionotropic glutamate receptor binding
1.095073	2.85E-06	0.004	GO:0070491	repressing transcription factor binding
1.094109	1.18E-10	<0.001	GO:0006754	ATP biosynthetic process
1.085865	3.06E-14	<0.001	GO:0015980	energy derivation by oxidation of organic compounds
1.085365	4.36E-08	0.001	GO:0045931	positive regulation of mitotic cell cycle
1.074116	1.16E-17	<0.001	GO:0071013	catalytic step 2 spliceosome
1.066892	1.02E-06	0.002	GO:0010907	positive regulation of glucose metabolic process
1.065603	6.29E-08	0.001	GO:0009132	nucleoside diphosphate metabolic process
1.057613	9.49E-17	<0.001	GO:0008135	translation factor activity, nucleic acid binding
1.050367	3.42E-17	<0.001	GO:0015078	hydrogen ion transmembrane transporter activity
1.04233	5.83E-06	0.015	GO:0007215	glutamate signaling pathway
1.039457	4.08E-22	<0.001	GO:0044455	mitochondrial membrane part

1.029306	1.65E-14	<0.001	GO:0035023	regulation of Rho protein signal transduction
1.026163	1.76E-10	<0.001	GO:0009206	purine ribonucleoside triphosphate biosynthetic process
1.023819	6.99E-10	<0.001	GO:0051781	positive regulation of cell division
1.022274	2.01E-06	0.003	GO:0007254	JNK cascade
1.022274	2.01E-06	0.003	GO:0033261	regulation of S phase
1.018179	8.11E-06	0.028	GO:0009112	nucleobase metabolic process
1.01408	2.45E-10	<0.001	GO:0009145	purine nucleoside triphosphate biosynthetic process
1.006781	6.92E-07	0.002	GO:0042169	SH2 domain binding
1.002324	3.37E-10	<0.001	GO:0009201	ribonucleoside triphosphate biosynthetic process
1.001571	2.77E-06	0.004	GO:0030879	mammary gland development
0.995298	1.11E-05	0.03	GO:0003899	DNA-directed RNA polymerase activity
0.995298	1.11E-05	0.03	GO:0019843	rRNA binding
0.995298	1.11E-05	0.03	GO:0034062	RNA polymerase activity
0.994467	2.38E-07	0.001	GO:0004715	non-membrane spanning protein tyrosine kinase activity
0.988665	9.43E-07	0.002	GO:0003684	damaged DNA binding
0.988665	9.43E-07	0.002	GO:0010676	positive regulation of cellular carbohydrate metabolic process
0.988665	9.43E-07	0.002	GO:0015934	large ribosomal subunit
0.988665	9.43E-07	0.002	GO:0045913	positive regulation of carbohydrate metabolic process
0.986336	3.60E-12	<0.001	GO:0015992	proton transport
0.978381	5.50E-11	<0.001	GO:0051302	regulation of cell division
0.978161	4.08E-38	<0.001	GO:0006412	translation
0.977454	4.86E-12	<0.001	GO:0006818	hydrogen transport
0.97476	2.44E-09	<0.001	GO:0018108	peptidyl-tyrosine phosphorylation
0.97476	2.44E-09	<0.001	GO:0018212	peptidyl-tyrosine modification
0.97356	1.50E-05	0.041	GO:0003156	regulation of organ formation
0.968914	5.80E-13	<0.001	GO:0005088	Ras guanyl-nucleotide exchange factor activity
0.968846	8.39E-10	<0.001	GO:0009142	nucleoside triphosphate biosynthetic process
0.968846	8.39E-10	<0.001	GO:0070469	respiratory chain
0.966267	3.77E-19	<0.001	GO:0006366	transcription from RNA polymerase II promoter
0.951551	2.70E-20	<0.001	GO:0005681	spliceosomal complex
0.947876	1.49E-09	<0.001	GO:0000079	regulation of cyclin-dependent protein kinase activity
0.938444	2.23E-06	0.003	GO:0031098	stress-activated protein kinase signaling cascade
0.933341	7.51E-07	0.002	GO:0045727	positive regulation of translation
0.929531	3.00E-17	<0.001	GO:0046578	regulation of Ras protein signal transduction
0.927707	3.50E-18	<0.001	GO:0006260	DNA replication
0.925696	8.57E-08	0.001	GO:0003697	single-stranded DNA binding
0.919319	4.20E-14	<0.001	GO:0003729	mRNA binding
0.9182	3.34E-09	<0.001	GO:0007596	blood coagulation
0.9182	3.34E-09	<0.001	GO:0050817	coagulation
0.912247	4.48E-11	<0.001	GO:0005057	receptor signaling protein activity
0.90874	4.33E-09	<0.001	GO:0007599	hemostasis
0.907919	3.77E-06	0.012	GO:0008286	insulin receptor signaling pathway
0.907919	3.77E-06	0.012	GO:0043525	positive regulation of neuron apoptosis
0.9075	1.47E-09	<0.001	GO:0006919	activation of caspase activity
0.903945	4.26E-07	0.002	GO:0045216	cell-cell junction organization
0.901296	4.86E-08	0.001	GO:0051219	phosphoprotein binding
0.894569	1.45E-05	0.036	GO:0002053	positive regulation of mesenchymal cell proliferation

0.891338	1.84E-07	0.001	GO:0048661	positive regulation of smooth muscle cell proliferation
0.890636	2.11E-08	0.001	GO:0010906	regulation of glucose metabolic process
0.886224	1.33E-22	<0.001	GO:0005840	ribosome
0.879394	6.15E-06	0.02	GO:0043255	regulation of carbohydrate biosynthetic process
0.874476	1.33E-09	<0.001	GO:0009411	response to UV
0.874304	3.07E-13	<0.001	GO:0006164	purine nucleotide biosynthetic process
0.87283	1.16E-08	0.001	GO:2000602	regulation of interphase of mitotic cell cycle
0.867322	2.61E-06	0.003	GO:0032321	positive regulation of Rho GTPase activity
0.867322	2.61E-06	0.003	GO:0043112	receptor metabolic process
0.862851	4.29E-08	0.001	GO:0018105	peptidyl-serine phosphorylation
0.859492	3.74E-07	0.002	GO:0050729	positive regulation of inflammatory response
0.858613	3.61E-11	<0.001	GO:0070851	growth factor receptor binding
0.857504	1.11E-06	0.002	GO:0005901	caveola
0.853812	9.07E-10	<0.001	GO:0019902	phosphatase binding
0.852624	9.74E-06	0.029	GO:0004722	protein serine/threonine phosphatase activity
0.852624	9.74E-06	0.029	GO:0043467	regulation of generation of precursor metabolites and energy
0.852329	4.98E-21	<0.001	GO:0007346	regulation of mitotic cell cycle
0.848467	1.93E-11	<0.001	GO:0045787	positive regulation of cell cycle
0.847713	2.29E-08	0.001	GO:0051028	mRNA transport
0.843284	1.07E-48	<0.001	GO:0030529	ribonucleoprotein complex
0.842543	1.98E-07	0.001	GO:0007088	regulation of mitosis
0.842543	1.98E-07	0.001	GO:0051783	regulation of nuclear division
0.842293	9.74E-09	0.001	GO:0030674	protein binding, bridging
0.83756	4.13E-09	<0.001	GO:0043280	positive regulation of caspase activity
0.833494	1.86E-12	<0.001	GO:0072522	purine-containing compound biosynthetic process
0.832036	5.07E-06	0.014	GO:0017053	transcriptional repressor complex
0.831738	3.54E-08	0.001	GO:0010675	regulation of cellular carbohydrate metabolic process
0.829816	7.22E-07	0.002	GO:0070374	positive regulation of ERK1 and ERK2 cascade
0.826984	3.02E-15	<0.001	GO:0009165	nucleotide biosynthetic process
0.826441	2.11E-21	<0.001	GO:0007169	transmembrane receptor protein tyrosine kinase signaling pathway
0.823964	4.37E-08	0.001	GO:0006109	regulation of carbohydrate metabolic process
0.820356	8.90E-07	0.002	GO:0006606	protein import into nucleus
0.820151	1.85E-08	0.001	GO:0071375	cellular response to peptide hormone stimulus
0.820149	1.28E-07	0.001	GO:0031461	cullin-RING ubiquitin ligase complex
0.817949	2.16E-13	<0.001	GO:0005085	guanyl-nucleotide exchange factor activity
0.815794	5.63E-15	<0.001	GO:0051056	regulation of small GTPase mediated signal transduction
0.814763	5.48E-19	<0.001	GO:0005096	GTPase activator activity
0.812517	6.01E-32	<0.001	GO:0032403	protein complex binding
0.811097	1.09E-06	0.002	GO:0008629	induction of apoptosis by intracellular signals
0.808821	6.60E-08	0.001	GO:0034330	cell junction organization
0.802963	1.77E-24	<0.001	GO:0006397	mRNA processing
0.802031	1.33E-06	0.002	GO:0032319	regulation of Rho GTPase activity
0.802031	1.33E-06	0.002	GO:0051170	nuclear import
0.801581	3.02E-10	<0.001	GO:0000209	protein polyubiquitination
0.801359	4.92E-09	<0.001	GO:0048167	regulation of synaptic plasticity
0.799395	9.36E-06	0.029	GO:0034329	cell junction assembly
0.799079	3.39E-08	0.001	GO:0007265	Ras protein signal transduction
0.794234	1.86E-14	<0.001	GO:0034404	nucleobase, nucleoside and nucleotide biosynthetic process

0.794234	1.86E-14	<0.001	GO:0034654	nucleobase, nucleoside, nucleotide and nucleic acid biosynthetic process
0.792831	2.67E-18	<0.001	GO:0008380	RNA splicing
0.787911	3.06E-09	<0.001	GO:0010952	positive regulation of peptidase activity
0.787047	1.19E-07	0.001	GO:0050657	nucleic acid transport
0.787047	1.19E-07	0.001	GO:0050658	RNA transport
0.787047	1.19E-07	0.001	GO:0051236	establishment of RNA localization
0.782713	8.22E-07	0.002	GO:0019903	protein phosphatase binding
0.782713	8.22E-07	0.002	GO:0032869	cellular response to insulin stimulus
0.782252	3.70E-09	<0.001	GO:0014069	postsynaptic density
0.778917	1.37E-05	0.036	GO:0008013	beta-catenin binding
0.778917	1.37E-05	0.036	GO:0031570	DNA integrity checkpoint
0.77732	1.06E-08	0.001	GO:0047485	protein N-terminus binding
0.775912	2.37E-06	0.003	GO:0051092	positive regulation of NF-kappaB transcription factor activity
0.775632	7.90E-10	<0.001	GO:0006200	ATP catabolic process
0.771939	1.20E-17	<0.001	GO:0006091	generation of precursor metabolites and energy
0.771511	1.28E-08	0.001	GO:0000075	cell cycle checkpoint
0.77058	1.24E-14	<0.001	GO:0008022	protein C-terminus binding
0.770482	4.44E-19	<0.001	GO:0007264	small GTPase mediated signal transduction
0.770076	5.28E-12	<0.001	GO:0046034	ATP metabolic process
0.768197	6.85E-06	0.021	GO:0032320	positive regulation of Ras GTPase activity
0.766328	2.67E-12	<0.001	GO:0006417	regulation of translation
0.766309	2.08E-07	0.001	GO:0045834	positive regulation of lipid metabolic process
0.761674	8.94E-15	<0.001	GO:0045121	membrane raft
0.761513	1.02E-10	<0.001	GO:0046777	protein autophosphorylation
0.760771	1.37E-09	<0.001	GO:0046822	regulation of nucleocytoplasmic transport
0.760542	3.25E-09	<0.001	GO:0019838	growth factor binding
0.759483	5.96E-07	0.002	GO:0018209	peptidyl-serine modification
0.759329	3.42E-06	0.01	GO:0007568	aging
0.759316	8.20E-06	0.029	GO:0090316	positive regulation of intracellular protein transport
0.756931	1.73E-35	<0.001	GO:0051726	regulation of cell cycle
0.756808	2.89E-10	<0.001	GO:0042176	regulation of protein catabolic process
0.755927	1.63E-09	<0.001	GO:0031625	ubiquitin protein ligase binding
0.754147	2.58E-11	<0.001	GO:0043434	response to peptide hormone stimulus
0.754044	5.24E-08	0.001	GO:0010563	negative regulation of phosphorus metabolic process
0.754044	5.24E-08	0.001	GO:0045936	negative regulation of phosphate metabolic process
0.753172	1.34E-26	<0.001	GO:0030695	GTPase regulator activity
0.751926	2.16E-23	<0.001	GO:0019901	protein kinase binding
0.748167	1.29E-24	<0.001	GO:0016071	mRNA metabolic process
0.74651	3.54E-07	0.002	GO:0048660	regulation of smooth muscle cell proliferation
0.746084	8.67E-23	<0.001	GO:0009259	ribonucleotide metabolic process
0.745928	1.78E-23	<0.001	GO:0007167	enzyme linked receptor protein signaling pathway
0.744741	8.37E-17	<0.001	GO:0005083	small GTPase regulator activity
0.743354	4.84E-06	0.014	GO:0010565	regulation of cellular ketone metabolic process
0.742077	1.16E-05	0.033	GO:0001948	glycoprotein binding
0.742077	1.16E-05	0.033	GO:0032388	positive regulation of intracellular transport
0.74171	2.75E-09	<0.001	GO:0071156	regulation of cell cycle arrest
0.741463	5.19E-26	<0.001	GO:0019900	kinase binding
0.741352	1.76E-07	0.001	GO:0010498	proteasomal protein catabolic process
0.740106	4.21E-07	0.002	GO:0002757	immune response-activating signal transduction

0.737246	2.40E-06	0.003	GO:0000302	response to reactive oxygen species
0.737246	2.40E-06	0.003	GO:0019199	transmembrane receptor protein kinase activity
0.735797	3.00E-18	<0.001	GO:0010564	regulation of cell cycle process
0.735769	7.75E-09	0.001	GO:0033157	regulation of intracellular protein transport
0.735722	1.01E-25	<0.001	GO:0060589	nucleoside-triphosphatase regulator activity
0.734121	1.14E-12	<0.001	GO:0006979	response to oxidative stress
0.733793	4.98E-07	0.002	GO:0000165	MAPKKK cascade
0.728646	2.21E-126	<0.001	GO:0032991	macromolecular complex
0.728568	1.07E-20	<0.001	GO:0009150	purine ribonucleotide metabolic process
0.72842	4.04E-10	<0.001	GO:0006913	nucleocytoplasmic transport
0.727627	5.16E-08	0.001	GO:0060090	binding, bridging
0.727571	5.88E-07	0.002	GO:0043547	positive regulation of GTPase activity
0.725447	1.40E-06	0.002	GO:0070372	regulation of ERK1 and ERK2 cascade
0.723689	2.91E-07	0.002	GO:0015931	nucleobase, nucleoside, nucleotide and nucleic acid transport
0.723534	2.10E-11	<0.001	GO:0043566	structure-specific DNA binding
0.721436	6.92E-07	0.002	GO:0042326	negative regulation of phosphorylation
0.716628	6.58E-10	<0.001	GO:0051169	nuclear transport
0.715386	8.13E-07	0.002	GO:0002764	immune response-regulating signaling pathway
0.715386	8.13E-07	0.002	GO:0043161	proteasomal ubiquitin-dependent protein catabolic process
0.715386	8.13E-07	0.002	GO:0051291	protein heterooligomerization
0.706712	4.70E-07	0.002	GO:0014070	response to organic cyclic compound
0.706229	2.26E-06	0.003	GO:0030027	lamellipodium
0.706018	2.22E-10	<0.001	GO:0015077	monovalent inorganic cation transmembrane transporter activity
0.701374	2.69E-19	<0.001	GO:0051301	cell division
0.70016	5.87E-09	<0.001	GO:0032386	regulation of intracellular transport
0.700007	2.64E-06	0.004	GO:0009123	nucleoside monophosphate metabolic process
0.699223	2.58E-17	<0.001	GO:0009205	purine ribonucleoside triphosphate metabolic process
0.697723	1.30E-06	0.002	GO:0042306	regulation of protein import into nucleus
0.696446	8.60E-19	<0.001	GO:0008047	enzyme activator activity
0.69523	5.74E-15	<0.001	GO:0009207	purine ribonucleoside triphosphate catabolic process
0.693576	4.04E-17	<0.001	GO:0009199	ribonucleoside triphosphate metabolic process
0.693251	1.08E-12	<0.001	GO:0018130	heterocycle biosynthetic process
0.69304	6.66E-15	<0.001	GO:0009203	ribonucleoside triphosphate catabolic process
0.693035	8.26E-10	<0.001	GO:0043410	positive regulation of MAPKKK cascade
0.691472	2.02E-10	<0.001	GO:0045786	negative regulation of cell cycle
0.690071	3.78E-08	0.001	GO:0050878	regulation of body fluid levels
0.689572	7.27E-06	0.024	GO:0090068	positive regulation of cell cycle process
0.688693	8.97E-15	<0.001	GO:0009146	purine nucleoside triphosphate catabolic process
0.688152	9.23E-09	0.001	GO:0004713	protein tyrosine kinase activity
0.688001	6.30E-17	<0.001	GO:0009144	purine nucleoside triphosphate metabolic process
0.687748	1.80E-07	0.001	GO:0031349	positive regulation of defense response
0.686023	1.11E-09	<0.001	GO:0043523	regulation of neuron apoptosis
0.684389	1.20E-14	<0.001	GO:0009154	purine ribonucleotide catabolic process
0.682188	1.03E-07	0.001	GO:0032103	positive regulation of response to external stimulus
0.680796	5.54E-17	<0.001	GO:0009141	nucleoside triphosphate metabolic process
0.68045	7.70E-27	<0.001	GO:0007049	cell cycle
0.678009	1.86E-14	<0.001	GO:0009143	nucleoside triphosphate catabolic process
0.676854	9.75E-06	0.029	GO:0006275	regulation of DNA replication

0.675903	2.14E-14	<0.001	GO:0009261	ribonucleotide catabolic process
0.675447	3.62E-13	<0.001	GO:0007243	intracellular protein kinase cascade
0.674106	5.67E-07	0.002	GO:0005976	polysaccharide metabolic process
0.672496	8.15E-09	0.001	GO:0019717	synaptosome
0.669398	1.36E-10	<0.001	GO:0032870	cellular response to hormone stimulus
0.668574	4.50E-15	<0.001	GO:0005667	transcription factor complex
0.668544	3.41E-44	<0.001	GO:0019899	enzyme binding
0.664779	9.00E-08	0.001	GO:0051091	positive regulation of sequence-specific DNA binding transcription factor activity
0.664472	7.53E-07	0.002	GO:0022900	electron transport chain
0.664282	4.40E-11	<0.001	GO:0050804	regulation of synaptic transmission
0.662649	1.50E-09	<0.001	GO:0051052	regulation of DNA metabolic process
0.662324	3.03E-09	<0.001	GO:0019216	regulation of lipid metabolic process
0.66135	2.52E-08	0.001	GO:0050727	regulation of inflammatory response
0.660056	4.26E-07	0.002	GO:0017124	SH3 domain binding
0.658768	7.29E-06	0.024	GO:0017038	protein import
0.658499	1.82E-14	<0.001	GO:0051345	positive regulation of hydrolase activity
0.657703	1.30E-19	<0.001	GO:0005198	structural molecule activity
0.655781	3.99E-09	<0.001	GO:0003924	GTPase activity
0.654604	1.63E-08	0.001	GO:0009416	response to light stimulus
0.654458	2.01E-06	0.003	GO:0050679	positive regulation of epithelial cell proliferation
0.654025	4.84E-14	<0.001	GO:0072523	purine-containing compound catabolic process
0.653839	4.10E-06	0.012	GO:0016779	nucleotidyltransferase activity
0.653182	8.35E-06	0.029	GO:0032868	response to insulin stimulus
0.653182	8.35E-06	0.029	GO:0044085	cellular component biogenesis
0.651561	1.54E-14	<0.001	GO:0009166	nucleotide catabolic process
0.651383	1.12E-13	<0.001	GO:0006195	purine nucleotide catabolic process
0.650857	3.12E-14	<0.001	GO:0010608	posttranscriptional regulation of gene expression
0.650407	1.13E-06	0.002	GO:0019221	cytokine-mediated signaling pathway
0.649123	6.79E-12	<0.001	GO:0051603	proteolysis involved in cellular protein catabolic process
0.647667	9.55E-06	0.029	GO:0030324	lung development
0.647396	1.17E-31	<0.001	GO:0003723	RNA binding
0.646651	2.14E-32	<0.001	GO:0006468	protein phosphorylation
0.64586	6.34E-15	<0.001	GO:0034655	nucleobase, nucleoside, nucleotide and nucleic acid catabolic process
0.64586	6.34E-15	<0.001	GO:0034656	nucleobase, nucleoside and nucleotide catabolic process
0.64544	1.56E-18	<0.001	GO:0005625	soluble fraction
0.64482	3.32E-37	<0.001	GO:0035556	intracellular signal transduction
0.644079	1.12E-20	<0.001	GO:0051247	positive regulation of protein metabolic process
0.643642	2.35E-17	<0.001	GO:0006163	purine nucleotide metabolic process
0.642522	7.30E-07	0.002	GO:0002253	activation of immune response
0.641275	1.48E-06	0.002	GO:0000151	ubiquitin ligase complex
0.640748	2.59E-15	<0.001	GO:0005743	mitochondrial inner membrane
0.640378	1.38E-21	<0.001	GO:0006396	RNA processing
0.639245	8.33E-11	<0.001	GO:0010035	response to inorganic substance
0.639245	8.33E-11	<0.001	GO:0051969	regulation of transmission of nerve impulse
0.638166	1.68E-10	<0.001	GO:0051272	positive regulation of cellular component movement
0.637772	6.03E-18	<0.001	GO:0009894	regulation of catabolic process
0.636147	1.05E-15	<0.001	GO:0019866	organelle inner membrane
0.632402	2.81E-82	<0.001	GO:0043234	protein complex
0.631617	6.41E-14	<0.001	GO:0010740	positive regulation of intracellular protein kinase

				cascade
0.629803	1.13E-14	<0.001	GO:0044270	cellular nitrogen compound catabolic process
0.629384	2.98E-07	0.002	GO:0043281	regulation of caspase activity
0.626996	2.59E-14	<0.001	GO:0046700	heterocycle catabolic process
0.625861	4.41E-06	0.013	GO:0031400	negative regulation of protein modification process
0.620286	9.34E-09	0.001	GO:0050678	regulation of epithelial cell proliferation
0.618901	1.21E-07	0.001	GO:0001525	angiogenesis
0.618901	1.21E-07	0.001	GO:0051098	regulation of binding
0.617754	3.86E-17	<0.001	GO:0072521	purine-containing compound metabolic process
0.616976	1.11E-12	<0.001	GO:0006281	DNA repair
0.616821	1.27E-11	<0.001	GO:0043408	regulation of MAPKKK cascade
0.616484	1.28E-30	<0.001	GO:0030234	enzyme regulator activity
0.615908	8.75E-07	0.002	GO:0006184	GTP catabolic process
0.615908	8.75E-07	0.002	GO:0010038	response to metal ion
0.614666	1.64E-10	<0.001	GO:0031644	regulation of neurological system process
0.6139	2.75E-25	<0.001	GO:0044451	nucleoplasm part
0.612711	6.48E-16	<0.001	GO:0032270	positive regulation of cellular protein metabolic process
0.61208	9.88E-07	0.002	GO:0042110	T cell activation
0.610888	5.53E-07	0.002	GO:0046039	GTP metabolic process
0.609894	1.00E-36	<0.001	GO:0051246	regulation of protein metabolic process
0.609429	1.99E-06	0.003	GO:0043235	receptor complex
0.609002	8.48E-18	<0.001	GO:0010627	regulation of intracellular protein kinase cascade
0.604533	1.45E-05	0.036	GO:0019207	kinase regulator activity
0.604533	1.45E-05	0.036	GO:0030100	regulation of endocytosis
0.604533	1.45E-05	0.036	GO:0032147	activation of protein kinase activity
0.602654	2.73E-14	<0.001	GO:0009725	response to hormone stimulus
0.602403	7.80E-50	<0.001	GO:0044428	nuclear part
0.601681	1.06E-09	<0.001	GO:0009314	response to radiation
0.60135	3.45E-33	<0.001	GO:0016310	phosphorylation
0.600886	2.65E-103	<0.001	GO:0044237	cellular metabolic process
0.599444	6.46E-16	<0.001	GO:0006974	response to DNA damage stimulus
0.599216	6.70E-10	<0.001	GO:0030036	actin cytoskeleton organization
0.599073	1.13E-12	<0.001	GO:0031401	positive regulation of protein modification process
0.598956	1.18E-10	<0.001	GO:0042327	positive regulation of phosphorylation
0.598924	4.57E-32	<0.001	GO:0032268	regulation of cellular protein metabolic process
0.598699	2.12E-23	<0.001	GO:0033554	cellular response to stress
0.598272	5.09E-06	0.014	GO:0030098	lymphocyte differentiation
0.598272	5.09E-06	0.014	GO:0043406	positive regulation of MAP kinase activity
0.596911	7.41E-11	<0.001	GO:0010562	positive regulation of phosphorus metabolic process
0.596911	7.41E-11	<0.001	GO:0045937	positive regulation of phosphate metabolic process
0.596024	4.45E-13	<0.001	GO:0008134	transcription factor binding
0.594941	1.53E-08	0.001	GO:0030335	positive regulation of cell migration
0.594797	2.65E-10	<0.001	GO:0030029	actin filament-based process
0.59475	2.69E-09	<0.001	GO:0051090	regulation of sequence-specific DNA binding transcription factor activity
0.59365	2.61E-19	<0.001	GO:0043069	negative regulation of programmed cell death
0.593312	6.34E-16	<0.001	GO:0031966	mitochondrial membrane
0.593212	2.92E-11	<0.001	GO:0032880	regulation of protein localization
0.593055	1.12E-102	<0.001	GO:0008152	metabolic process
0.592441	1.69E-09	<0.001	GO:0001934	positive regulation of protein phosphorylation

0.592322	1.71E-08	0.001	GO:0022890	inorganic cation transmembrane transporter activity
0.592153	1.16E-20	<0.001	GO:0008284	positive regulation of cell proliferation
0.591236	5.88E-19	<0.001	GO:0043066	negative regulation of apoptosis
0.589966	3.91E-13	<0.001	GO:0031329	regulation of cellular catabolic process
0.589719	1.92E-08	0.001	GO:2000147	positive regulation of cell motility
0.589205	1.12E-17	<0.001	GO:0006753	nucleoside phosphate metabolic process
0.589205	1.12E-17	<0.001	GO:0009117	nucleotide metabolic process
0.588424	4.38E-13	<0.001	GO:0005525	GTP binding
0.58761	1.53E-13	<0.001	GO:0019001	guanyl nucleotide binding
0.58761	1.53E-13	<0.001	GO:0032561	guanyl ribonucleotide binding
0.587569	1.73E-19	<0.001	GO:0060548	negative regulation of cell death
0.587539	5.89E-20	<0.001	GO:0019904	protein domain specific binding
0.587131	2.15E-08	0.001	GO:0050778	positive regulation of immune response
0.584998	6.31E-36	<0.001	GO:0006796	phosphate metabolic process
0.584065	7.75E-36	<0.001	GO:0006793	phosphorus metabolic process
0.584055	8.00E-95	<0.001	GO:0044424	intracellular part
0.583786	1.37E-07	0.001	GO:0052548	regulation of endopeptidase activity
0.583777	1.50E-97	<0.001	GO:0044238	primary metabolic process
0.581996	1.44E-05	0.036	GO:0002237	response to molecule of bacterial origin
0.581642	2.84E-18	<0.001	GO:0006259	DNA metabolic process
0.580777	3.25E-10	<0.001	GO:0071900	regulation of protein serine/threonine kinase activity
0.580632	3.16E-18	<0.001	GO:0022402	cell cycle process
0.578619	3.92E-18	<0.001	GO:0055086	nucleobase, nucleoside and nucleotide metabolic process
0.575377	3.91E-85	<0.001	GO:0044260	cellular macromolecule metabolic process
0.573547	3.70E-22	<0.001	GO:0042325	regulation of phosphorylation
0.573423	1.08E-06	0.002	GO:0071902	positive regulation of protein serine/threonine kinase activity
0.573143	1.19E-07	0.001	GO:0060627	regulation of vesicle-mediated transport
0.572441	9.33E-23	<0.001	GO:0019220	regulation of phosphate metabolic process
0.572441	9.33E-23	<0.001	GO:0051174	regulation of phosphorus metabolic process
0.571543	1.80E-87	<0.001	GO:0043170	macromolecule metabolic process
0.570258	1.21E-06	0.002	GO:0043087	regulation of GTPase activity
0.569913	2.39E-24	<0.001	GO:0048584	positive regulation of response to stimulus
0.569442	2.16E-06	0.003	GO:0048511	rhythmic process
0.569429	4.62E-08	0.001	GO:0030155	regulation of cell adhesion
0.568775	3.17E-09	<0.001	GO:0006917	induction of apoptosis
0.56863	8.25E-08	0.001	GO:0006511	ubiquitin-dependent protein catabolic process
0.568353	7.03E-20	<0.001	GO:0046483	heterocycle metabolic process
0.567658	6.91E-06	0.023	GO:0050670	regulation of lymphocyte proliferation
0.566958	5.14E-08	0.001	GO:0040017	positive regulation of locomotion
0.566766	3.54E-52	<0.001	GO:0044267	cellular protein metabolic process
0.566679	3.52E-09	<0.001	GO:0012502	induction of programmed cell death
0.566049	4.23E-15	<0.001	GO:0043549	regulation of kinase activity
0.564868	2.96E-13	<0.001	GO:0009057	macromolecule catabolic process
0.564307	2.49E-88	<0.001	GO:0005515	protein binding
0.564157	1.79E-15	<0.001	GO:0051338	regulation of transferase activity
0.56405	7.68E-06	0.026	GO:0032944	regulation of mononuclear cell proliferation
0.563995	1.49E-06	0.002	GO:0033124	regulation of GTP catabolic process
0.561556	1.59E-19	<0.001	GO:0001932	regulation of protein phosphorylation
0.55977	2.36E-59	<0.001	GO:0019538	protein metabolic process
0.5591	1.03E-06	0.002	GO:0002521	leukocyte differentiation

0.558345	5.29E-06	0.014	GO:0001763	morphogenesis of a branching structure
0.557888	4.05E-10	<0.001	GO:0033674	positive regulation of kinase activity
0.557302	9.52E-09	0.001	GO:0051129	negative regulation of cellular component organization
0.555866	9.83E-18	<0.001	GO:0004672	protein kinase activity
0.555769	3.71E-18	<0.001	GO:0010647	positive regulation of cell communication
0.554584	1.08E-12	<0.001	GO:0009628	response to abiotic stimulus
0.553501	1.54E-07	0.001	GO:0019941	modification-dependent protein catabolic process
0.553403	1.05E-05	0.029	GO:0070663	regulation of leukocyte proliferation
0.553265	3.64E-06	0.012	GO:0070727	cellular macromolecule localization
0.553262	8.01E-15	<0.001	GO:0043068	positive regulation of programmed cell death
0.551993	3.42E-10	<0.001	GO:0051347	positive regulation of transferase activity
0.551255	1.71E-21	<0.001	GO:0031399	regulation of protein modification process
0.551029	1.70E-07	0.001	GO:0000280	nuclear division
0.551029	1.70E-07	0.001	GO:0007067	mitosis
0.550893	1.94E-13	<0.001	GO:0045859	regulation of protein kinase activity
0.549465	2.24E-16	<0.001	GO:0009967	positive regulation of signal transduction
0.548939	1.93E-14	<0.001	GO:0043065	positive regulation of apoptosis
0.548513	8.79E-28	<0.001	GO:0043067	regulation of programmed cell death
0.548378	2.31E-23	<0.001	GO:0044093	positive regulation of molecular function
0.547337	9.98E-11	<0.001	GO:0044271	cellular nitrogen compound biosynthetic process
0.547335	7.43E-10	<0.001	GO:0050776	regulation of immune response
0.546598	8.94E-15	<0.001	GO:0010942	positive regulation of cell death
0.546127	2.08E-07	0.001	GO:0043632	modification-dependent macromolecule catabolic process
0.545791	2.14E-25	<0.001	GO:0016772	transferase activity, transferring phosphorus-containing groups
0.544842	1.64E-26	<0.001	GO:0042127	regulation of cell proliferation
0.544745	4.29E-27	<0.001	GO:0042981	regulation of apoptosis
0.544545	4.20E-69	<0.001	GO:0008150	biological_process
0.543357	2.38E-17	<0.001	GO:0023056	positive regulation of signaling
0.54287	2.59E-09	<0.001	GO:0032101	regulation of response to external stimulus
0.542002	1.17E-27	<0.001	GO:0010941	regulation of cell death
0.540166	7.27E-07	0.002	GO:0080135	regulation of cellular response to stress
0.539937	4.24E-10	<0.001	GO:0046982	protein heterodimerization activity
0.538566	9.67E-06	0.029	GO:0006916	anti-apoptosis
0.538566	9.67E-06	0.029	GO:0034613	cellular protein localization
0.538509	7.53E-10	<0.001	GO:0001775	cell activation
0.537662	5.99E-06	0.016	GO:0006605	protein targeting
0.536482	3.09E-07	0.002	GO:0051641	cellular localization
0.536308	7.59E-11	<0.001	GO:0051270	regulation of cellular component movement
0.53623	2.64E-12	<0.001	GO:0008285	negative regulation of cell proliferation
0.53623	2.64E-12	<0.001	GO:0044057	regulation of system process
0.53562	1.19E-07	0.001	GO:0043009	chordate embryonic development
0.535591	1.68E-77	<0.001	GO:0009987	cellular process
0.53555	1.42E-06	0.002	GO:0032269	negative regulation of cellular protein metabolic process
0.535355	1.07E-05	0.03	GO:0006725	cellular aromatic compound metabolic process
0.534819	2.40E-18	<0.001	GO:0043085	positive regulation of catalytic activity
0.534607	6.60E-06	0.02	GO:0048545	response to steroid hormone stimulus
0.534426	1.76E-08	0.001	GO:0033036	macromolecule localization
0.532167	1.17E-05	0.035	GO:0010959	regulation of metal ion transport
0.530822	5.54E-08	0.001	GO:0044087	regulation of cellular component biogenesis

0.530285	1.21E-09	<0.001	GO:0051179	localization
0.530024	9.83E-80	<0.001	GO:0005488	binding
0.528593	1.45E-08	0.001	GO:0045860	positive regulation of protein kinase activity
0.528184	4.94E-06	0.014	GO:0050867	positive regulation of cell activation
0.526928	2.80E-07	0.002	GO:0001701	in utero embryonic development
0.526928	2.80E-07	0.002	GO:0051248	negative regulation of protein metabolic process
0.526839	1.74E-07	0.001	GO:0009792	embryo development ending in birth or egg hatching
0.525567	8.77E-06	0.029	GO:0002696	positive regulation of leukocyte activation
0.525134	3.36E-06	0.01	GO:0005996	monosaccharide metabolic process
0.524847	1.62E-20	<0.001	GO:0016301	kinase activity
0.523328	1.77E-10	<0.001	GO:0002684	positive regulation of immune system process
0.522514	2.09E-07	0.001	GO:0070201	regulation of establishment of protein localization
0.522371	8.78E-07	0.002	GO:0002009	morphogenesis of an epithelium
0.52221	1.93E-09	<0.001	GO:0030334	regulation of cell migration
0.52221	1.93E-09	<0.001	GO:0044265	cellular macromolecule catabolic process
0.521848	5.28E-14	<0.001	GO:0009719	response to endogenous stimulus
0.521571	8.01E-35	<0.001	GO:0048583	regulation of response to stimulus
0.521493	1.78E-43	<0.001	GO:0034645	cellular macromolecule biosynthetic process
0.521077	1.40E-29	<0.001	GO:0005622	intracellular
0.52104	6.96E-44	<0.001	GO:0009059	macromolecule biosynthetic process
0.520097	5.97E-07	0.002	GO:0048285	organelle fission
0.520011	2.29E-12	<0.001	GO:0080134	regulation of response to stress
0.519719	4.05E-06	0.012	GO:0030811	regulation of nucleotide catabolic process
0.519719	4.05E-06	0.012	GO:0033121	regulation of purine nucleotide catabolic process
0.519568	1.12E-27	<0.001	GO:0009966	regulation of signal transduction
0.51914	3.73E-08	0.001	GO:0045321	leukocyte activation
0.518728	3.75E-09	<0.001	GO:0071495	cellular response to endogenous stimulus
0.518442	7.65E-63	<0.001	GO:0044446	intracellular organelle part
0.518428	6.06E-10	<0.001	GO:0033043	regulation of organelle organization
0.518169	1.83E-63	<0.001	GO:0044422	organelle part
0.518014	4.06E-07	0.002	GO:0031347	regulation of defense response
0.517099	5.81E-52	<0.001	GO:0044249	cellular biosynthetic process
0.515864	3.45E-17	<0.001	GO:0016773	phosphotransferase activity, alcohol group as acceptor
0.511231	8.62E-08	0.001	GO:0002694	regulation of leukocyte activation
0.510532	1.55E-25	<0.001	GO:0010646	regulation of cell communication
0.510135	7.36E-15	<0.001	GO:0051094	positive regulation of developmental process
0.509811	8.90E-13	<0.001	GO:0060341	regulation of cellular localization
0.509785	3.61E-07	0.002	GO:0051051	negative regulation of transport
0.509634	1.66E-51	<0.001	GO:0009058	biosynthetic process
0.509589	3.98E-09	<0.001	GO:2000145	regulation of cell motility
0.509141	1.67E-09	<0.001	GO:0008283	cell proliferation
0.508511	1.03E-08	0.001	GO:0018193	peptidyl-amino acid modification
0.507723	2.15E-30	<0.001	GO:0023051	regulation of signaling
0.506691	1.16E-12	<0.001	GO:0044282	small molecule catabolic process
0.506413	6.78E-53	<0.001	GO:0006139	nucleobase, nucleoside, nucleotide and nucleic acid metabolic process
0.502552	8.31E-08	0.001	GO:0050865	regulation of cell activation
0.502235	3.49E-08	0.001	GO:0009790	embryo development
0.501276	7.58E-06	0.024	GO:0032844	regulation of homeostatic process
0.500733	1.57E-21	<0.001	GO:0051128	regulation of cellular component organization
0.500602	1.89E-14	<0.001	GO:0017111	nucleoside-triphosphatase activity

0.499619	1.36E-20	<0.001	GO:0044248	cellular catabolic process
0.498914	1.53E-10	<0.001	GO:0004674	protein serine/threonine kinase activity
0.498911	1.79E-13	<0.001	GO:0044429	mitochondrial part
0.498803	1.74E-08	0.001	GO:0022403	cell cycle phase
0.498594	9.02E-07	0.002	GO:0008104	protein localization
0.497696	1.45E-06	0.002	GO:0051249	regulation of lymphocyte activation
0.497674	7.32E-15	<0.001	GO:0016462	pyrophosphatase activity
0.495297	3.74E-26	<0.001	GO:0050790	regulation of catalytic activity
0.495232	9.40E-15	<0.001	GO:0016818	hydrolase activity, acting on acid anhydrides, in phosphorus-containing anhydrides
0.494422	1.02E-14	<0.001	GO:0016817	hydrolase activity, acting on acid anhydrides
0.493968	8.21E-14	<0.001	GO:0002682	regulation of immune system process
0.493421	4.11E-06	0.012	GO:0022604	regulation of cell morphogenesis
0.492828	7.24E-58	<0.001	GO:0003674	molecular_function
0.492251	2.77E-06	0.004	GO:0046649	lymphocyte activation
0.491442	2.99E-30	<0.001	GO:0065009	regulation of molecular function
0.491418	4.77E-24	<0.001	GO:0000267	cell fraction
0.491092	4.47E-06	0.013	GO:0051223	regulation of protein transport
0.487028	3.42E-27	<0.001	GO:0031325	positive regulation of cellular metabolic process
0.486349	1.60E-10	<0.001	GO:0044283	small molecule biosynthetic process
0.485859	3.91E-51	<0.001	GO:0034641	cellular nitrogen compound metabolic process
0.48422	3.59E-28	<0.001	GO:0009893	positive regulation of metabolic process
0.483258	4.40E-16	<0.001	GO:0005829	cytosol
0.482808	1.37E-10	<0.001	GO:0022603	regulation of anatomical structure morphogenesis
0.481892	5.15E-16	<0.001	GO:0070887	cellular response to chemical stimulus
0.48172	8.50E-13	<0.001	GO:0071310	cellular response to organic substance
0.480212	1.56E-37	<0.001	GO:0000166	nucleotide binding
0.478463	3.07E-06	0.004	GO:0034097	response to cytokine stimulus
0.478248	3.97E-07	0.002	GO:0048729	tissue morphogenesis
0.478072	2.98E-68	<0.001	GO:0043229	intracellular organelle
0.476686	7.09E-68	<0.001	GO:0043226	organelle
0.476416	1.69E-25	<0.001	GO:0010604	positive regulation of macromolecule metabolic process
0.476404	9.15E-58	<0.001	GO:0005634	nucleus
0.474993	4.27E-43	<0.001	GO:0048518	positive regulation of biological process
0.473584	4.83E-49	<0.001	GO:0006807	nitrogen compound metabolic process
0.472731	2.79E-12	<0.001	GO:0051336	regulation of hydrolase activity
0.472714	8.24E-49	<0.001	GO:0005575	cellular_component
0.472563	1.55E-07	0.001	GO:0034621	cellular macromolecular complex subunit organization
0.472541	5.79E-06	0.014	GO:0003682	chromatin binding
0.472384	3.01E-09	<0.001	GO:0051130	positive regulation of cellular component organization
0.472286	2.16E-08	0.001	GO:0044262	cellular carbohydrate metabolic process
0.471115	2.78E-21	<0.001	GO:0009056	catabolic process
0.470987	1.38E-05	0.036	GO:0010324	membrane invagination
0.470134	1.60E-09	<0.001	GO:0045597	positive regulation of cell differentiation
0.468424	1.10E-51	<0.001	GO:0044464	cell part
0.467616	2.79E-10	<0.001	GO:0048646	anatomical structure formation involved in morphogenesis
0.466621	4.38E-08	0.001	GO:0040012	regulation of locomotion
0.46581	1.42E-38	<0.001	GO:0048522	positive regulation of cellular process
0.463411	7.68E-20	<0.001	GO:0031328	positive regulation of cellular biosynthetic process

0.462262	5.85E-34	<0.001	GO:0048523	negative regulation of cellular process
0.462042	5.77E-06	0.014	GO:0016567	protein ubiquitination
0.460494	1.31E-07	0.001	GO:0045596	negative regulation of cell differentiation
0.459399	3.19E-28	<0.001	GO:0035639	purine ribonucleoside triphosphate binding
0.459248	1.30E-28	<0.001	GO:0032553	ribonucleotide binding
0.459	9.02E-54	<0.001	GO:0005737	cytoplasm
0.458726	1.12E-19	<0.001	GO:0009891	positive regulation of biosynthetic process
0.458433	4.88E-27	<0.001	GO:0006464	protein modification process
0.45695	2.22E-06	0.003	GO:0052547	regulation of peptidase activity
0.456648	3.29E-06	0.01	GO:0034622	cellular macromolecular complex assembly
0.456322	3.49E-28	<0.001	GO:0032555	purine ribonucleotide binding
0.455731	1.07E-05	0.03	GO:0030425	dendrite
0.455203	2.54E-35	<0.001	GO:0048519	negative regulation of biological process
0.454843	4.91E-28	<0.001	GO:0042221	response to chemical stimulus
0.454252	5.63E-28	<0.001	GO:0017076	purine nucleotide binding
0.453817	7.14E-24	<0.001	GO:0006950	response to stress
0.452627	1.91E-12	<0.001	GO:0043933	macromolecular complex subunit organization
0.451599	2.78E-06	0.004	GO:0051259	protein oligomerization
0.451466	6.20E-10	<0.001	GO:0043005	neuron projection
0.451223	2.44E-36	<0.001	GO:0090304	nucleic acid metabolic process
0.451143	1.81E-11	<0.001	GO:0065003	macromolecular complex assembly
0.448874	1.87E-08	0.001	GO:0051050	positive regulation of transport
0.448356	1.32E-26	<0.001	GO:0043412	macromolecule modification
0.448346	4.63E-11	<0.001	GO:0071844	cellular component assembly at cellular level
0.448173	1.08E-17	<0.001	GO:0005102	receptor binding
0.448139	1.06E-06	0.002	GO:0043025	neuronal cell body
0.446791	7.00E-19	<0.001	GO:0010033	response to organic substance
0.445808	1.52E-15	<0.001	GO:0022607	cellular component assembly
0.444507	3.47E-17	<0.001	GO:0010557	positive regulation of macromolecule biosynthetic process
0.439408	4.14E-09	<0.001	GO:0071822	protein complex subunit organization
0.439281	1.61E-57	<0.001	GO:0043231	intracellular membrane-bounded organelle
0.438848	2.05E-57	<0.001	GO:0043227	membrane-bounded organelle
0.43707	3.04E-07	0.002	GO:0005730	nucleolus
0.436263	2.52E-18	<0.001	GO:0050793	regulation of developmental process
0.434751	2.33E-18	<0.001	GO:0032879	regulation of localization
0.433852	4.34E-44	<0.001	GO:0019222	regulation of metabolic process
0.43379	1.36E-15	<0.001	GO:0031324	negative regulation of cellular metabolic process
0.432337	5.03E-08	0.001	GO:0051093	negative regulation of developmental process
0.432337	5.03E-08	0.001	GO:0051240	positive regulation of multicellular organismal process
0.431816	2.54E-09	<0.001	GO:0006915	apoptosis
0.430772	1.96E-09	<0.001	GO:0012501	programmed cell death
0.429055	1.43E-21	<0.001	GO:0065008	regulation of biological quality
0.428708	4.47E-08	0.001	GO:0007010	cytoskeleton organization
0.428073	1.76E-10	<0.001	GO:0045944	positive regulation of transcription from RNA polymerase II promoter
0.427584	4.78E-08	0.001	GO:0040008	regulation of growth
0.427148	8.66E-39	<0.001	GO:0080090	regulation of primary metabolic process
0.426535	3.17E-12	<0.001	GO:0045934	negative regulation of nucleobase, nucleoside, nucleotide and nucleic acid metabolic process
0.426287	2.67E-08	0.001	GO:0006461	protein complex assembly
0.426192	1.11E-05	0.03	GO:0003006	developmental process involved in reproduction
0.425401	1.06E-38	<0.001	GO:0031323	regulation of cellular metabolic process

0.425197	3.62E-12	<0.001	GO:2000113	negative regulation of cellular macromolecule biosynthetic process
0.424089	4.62E-31	<0.001	GO:0003676	nucleic acid binding
0.423713	2.50E-37	<0.001	GO:0060255	regulation of macromolecule metabolic process
0.422856	7.00E-10	<0.001	GO:0008219	cell death
0.422177	4.35E-15	<0.001	GO:0010605	negative regulation of macromolecule metabolic process
0.421261	4.56E-21	<0.001	GO:0051239	regulation of multicellular organismal process
0.420544	5.71E-12	<0.001	GO:0051172	negative regulation of nitrogen compound metabolic process
0.420228	3.71E-06	0.012	GO:0044297	cell body
0.419897	1.41E-06	0.002	GO:0009887	organ morphogenesis
0.419309	3.46E-15	<0.001	GO:0051173	positive regulation of nitrogen compound metabolic process
0.417161	2.22E-06	0.003	GO:0045202	synapse
0.416371	1.57E-15	<0.001	GO:0009892	negative regulation of metabolic process
0.416305	3.17E-13	<0.001	GO:0005626	insoluble fraction
0.416134	1.18E-09	<0.001	GO:0016265	death
0.416011	4.68E-07	0.002	GO:0030030	cell projection organization
0.415271	1.37E-07	0.001	GO:0044427	chromosomal part
0.41186	9.02E-14	<0.001	GO:2000026	regulation of multicellular organismal development
0.410995	1.16E-10	<0.001	GO:0051253	negative regulation of RNA metabolic process
0.410686	3.41E-12	<0.001	GO:0045595	regulation of cell differentiation
0.409674	8.81E-13	<0.001	GO:0051049	regulation of transport
0.408927	9.81E-12	<0.001	GO:0031327	negative regulation of cellular biosynthetic process
0.407501	2.02E-11	<0.001	GO:0010558	negative regulation of macromolecule biosynthetic process
0.404995	2.85E-06	0.004	GO:0051046	regulation of secretion
0.4046	1.04E-28	<0.001	GO:0071840	cellular component organization or biogenesis
0.403914	1.24E-12	<0.001	GO:0051254	positive regulation of RNA metabolic process
0.401641	4.03E-07	0.002	GO:0023057	negative regulation of signaling
0.400959	6.82E-18	<0.001	GO:0005524	ATP binding
0.400933	4.06E-18	<0.001	GO:0032559	adenyl ribonucleotide binding
0.400534	4.29E-07	0.002	GO:0010648	negative regulation of cell communication
0.398472	6.10E-18	<0.001	GO:0030554	adenyl nucleotide binding
0.397757	2.93E-11	<0.001	GO:0009890	negative regulation of biosynthetic process
0.397348	7.43E-06	0.024	GO:0044456	synapse part
0.396852	3.00E-13	<0.001	GO:0045935	positive regulation of nucleobase, nucleoside, nucleotide and nucleic acid metabolic process
0.396005	3.68E-22	<0.001	GO:0043228	non-membrane-bounded organelle
0.396005	3.68E-22	<0.001	GO:0043232	intracellular non-membrane-bounded organelle
0.395713	1.43E-10	<0.001	GO:0046983	protein dimerization activity
0.393987	1.55E-09	<0.001	GO:0045892	negative regulation of transcription, DNA-dependent
0.393378	1.97E-22	<0.001	GO:0071841	cellular component organization or biogenesis at cellular level
0.391661	4.02E-11	<0.001	GO:0005624	membrane fraction
0.391109	1.04E-23	<0.001	GO:0016070	RNA metabolic process
0.390558	3.88E-26	<0.001	GO:0016043	cellular component organization
0.389615	2.21E-09	<0.001	GO:0002376	immune system process
0.388873	1.81E-11	<0.001	GO:0045893	positive regulation of transcription, DNA-dependent
0.383444	4.42E-06	0.013	GO:0009968	negative regulation of signal transduction
0.381831	1.02E-11	<0.001	GO:0010628	positive regulation of gene expression
0.38074	9.26E-10	<0.001	GO:0010629	negative regulation of gene expression
0.380307	2.54E-07	0.002	GO:0048585	negative regulation of response to stimulus

0.376796	5.86E-20	<0.001	GO:0071842	cellular component organization at cellular level
0.376282	6.23E-06	0.02	GO:0000122	negative regulation of transcription from RNA polymerase II promoter
0.375866	2.16E-06	0.003	GO:0044463	cell projection part
0.374038	1.43E-11	<0.001	GO:0042995	cell projection
0.372124	3.22E-07	0.002	GO:0046907	intracellular transport
0.371316	1.62E-09	<0.001	GO:0042592	homeostatic process
0.364224	1.44E-09	<0.001	GO:0055114	oxidation-reduction process
0.363538	4.05E-22	<0.001	GO:2000112	regulation of cellular macromolecule biosynthetic process
0.363521	5.69E-14	<0.001	GO:0006996	organelle organization
0.363249	1.49E-05	0.037	GO:0051960	regulation of nervous system development
0.361366	3.14E-22	<0.001	GO:0010556	regulation of macromolecule biosynthetic process
0.361076	6.25E-23	<0.001	GO:0031326	regulation of cellular biosynthetic process
0.359629	2.09E-10	<0.001	GO:0006357	regulation of transcription from RNA polymerase II promoter
0.357462	7.80E-16	<0.001	GO:0048856	anatomical structure development
0.356708	1.51E-22	<0.001	GO:0009889	regulation of biosynthetic process
0.356521	7.28E-33	<0.001	GO:0044444	cytoplasmic part
0.354583	2.13E-09	<0.001	GO:0031090	organelle membrane
0.354013	2.33E-07	0.001	GO:0030054	cell junction
0.353371	5.76E-22	<0.001	GO:0010468	regulation of gene expression
0.353019	1.14E-05	0.031	GO:0060284	regulation of cell development
0.352114	2.27E-15	<0.001	GO:0016740	transferase activity
0.347783	1.41E-06	0.002	GO:0048878	chemical homeostasis
0.346679	1.19E-06	0.002	GO:0044092	negative regulation of molecular function
0.344034	6.07E-08	0.001	GO:0048513	organ development
0.343549	1.22E-07	0.001	GO:0042802	identical protein binding
0.342106	2.93E-14	<0.001	GO:0044281	small molecule metabolic process
0.338559	2.26E-21	<0.001	GO:0032502	developmental process
0.335217	1.91E-06	0.002	GO:0006508	proteolysis
0.334752	2.89E-09	<0.001	GO:0009653	anatomical structure morphogenesis
0.334348	3.98E-34	<0.001	GO:0050789	regulation of biological process
0.333947	2.87E-34	<0.001	GO:0065007	biological regulation
0.3304	2.29E-19	<0.001	GO:0051171	regulation of nitrogen compound metabolic process
0.328146	1.99E-07	0.001	GO:0051649	establishment of localization in cell
0.327394	7.15E-19	<0.001	GO:0019219	regulation of nucleobase, nucleoside, nucleotide and nucleic acid metabolic process
0.319727	7.21E-31	<0.001	GO:0050794	regulation of cellular process
0.317847	1.19E-25	<0.001	GO:0003824	catalytic activity
0.314982	1.90E-12	<0.001	GO:0003677	DNA binding
0.314576	6.14E-11	<0.001	GO:0005739	mitochondrion
0.312162	6.52E-12	<0.001	GO:0048869	cellular developmental process
0.306842	1.02E-22	<0.001	GO:0051716	cellular response to stimulus
0.304928	3.13E-06	0.004	GO:0045184	establishment of protein localization
0.302844	1.60E-14	<0.001	GO:0051252	regulation of RNA metabolic process
0.302109	8.32E-25	<0.001	GO:0050896	response to stimulus
0.301954	5.43E-09	<0.001	GO:0030154	cell differentiation
0.295129	1.65E-13	<0.001	GO:0006355	regulation of transcription, DNA-dependent
0.293768	1.06E-05	0.03	GO:0015031	protein transport
0.287859	4.89E-12	<0.001	GO:0016787	hydrolase activity
0.287747	4.02E-10	<0.001	GO:0032774	RNA biosynthetic process
0.286517	5.41E-10	<0.001	GO:0006351	transcription, DNA-dependent

0.269509	3.20E-06	0.004	GO:0044430	cytoskeletal part
0.267198	6.23E-12	<0.001	GO:0051234	establishment of localization
0.258541	4.84E-11	<0.001	GO:0006810	transport
0.25563	2.73E-14	<0.001	GO:0007165	signal transduction
0.205473	1.94E-07	0.001	GO:0005886	plasma membrane
0.16938	2.48E-06	0.003	GO:0043167	ion binding
0.162819	6.88E-06	0.023	GO:0046872	metal ion binding
0.162626	6.45E-06	0.02	GO:0043169	cation binding
down-regulated in AKI				
2.714081	2.47E-12	<0.001	GO:0022625	cytosolic large ribosomal subunit
2.669134	1.30E-48	<0.001	GO:0022627	cytosolic small ribosomal subunit
2.550234	2.54E-08	<0.001	GO:0005851	eukaryotic translation initiation factor 2B complex
2.439031	2.42E-06	0.006	GO:0032057	negative regulation of translational initiation in response to stress
2.417586	8.86E-10	<0.001	GO:0031145	anaphase-promoting complex-dependent proteasomal ubiquitin-dependent protein catabolic process
2.417586	8.86E-10	<0.001	GO:0042273	ribosomal large subunit biogenesis
2.408012	1.38E-16	<0.001	GO:0005852	eukaryotic translation initiation factor 3 complex
2.328368	7.58E-08	<0.001	GO:0043558	regulation of translational initiation in response to stress
2.29386	1.25E-17	<0.001	GO:0005680	anaphase-promoting complex
2.182223	1.76E-07	<0.001	GO:0000028	ribosomal small subunit assembly
2.161552	1.98E-21	<0.001	GO:0070979	protein K11-linked ubiquitination
2.14974	1.59E-10	<0.001	GO:0042274	ribosomal small subunit biogenesis
2.120618	1.32E-40	<0.001	GO:0015935	small ribosomal subunit
2.085515	2.79E-16	<0.001	GO:0000152	nuclear ubiquitin ligase complex
2.073061	3.49E-07	<0.001	GO:0043555	regulation of translation in response to stress
2.07102	1.20E-05	0.027	GO:0032055	negative regulation of translation in response to stress
1.986901	1.01E-36	<0.001	GO:0006413	translational initiation
1.951196	2.18E-60	<0.001	GO:0003735	structural constituent of ribosome
1.913325	1.03E-06	0.001	GO:0005675	holo TFIIH complex
1.886003	5.21E-08	<0.001	GO:0031369	translation initiation factor binding
1.845002	1.15E-23	<0.001	GO:0003743	translation initiation factor activity
1.737411	1.47E-74	<0.001	GO:0006412	translation
1.685001	3.35E-07	<0.001	GO:0030071	regulation of mitotic metaphase/anaphase transition
1.668927	9.04E-53	<0.001	GO:0005840	ribosome
1.654318	8.69E-12	<0.001	GO:0015934	large ribosomal subunit
1.642323	2.27E-09	<0.001	GO:0019843	rRNA binding
1.629224	8.60E-06	0.013	GO:0014003	oligodendrocyte development
1.621297	2.14E-10	<0.001	GO:0006446	regulation of translational initiation
1.546891	1.73E-19	<0.001	GO:0008135	translation factor activity, nucleic acid binding
1.483213	2.19E-07	<0.001	GO:0070936	protein K48-linked ubiquitination
1.482756	2.43E-06	0.006	GO:0043022	ribosome binding
1.408585	5.13E-06	0.009	GO:0021782	glial cell development
1.372578	5.10E-16	<0.001	GO:0000209	protein polyubiquitination
1.365713	2.17E-11	<0.001	GO:0022613	ribonucleoprotein complex biogenesis
1.33871	3.08E-10	<0.001	GO:0031461	cullin-RING ubiquitin ligase complex
1.311638	7.20E-11	<0.001	GO:0071843	cellular component biogenesis at cellular level
1.299625	3.71E-48	<0.001	GO:0030529	ribonucleoprotein complex
1.272354	1.48E-36	<0.001	GO:0005198	structural molecule activity
1.252284	2.70E-10	<0.001	GO:0006364	rRNA processing

1.235944	3.89E-10	<0.001	GO:0016072	rRNA metabolic process
1.23063	4.38E-10	<0.001	GO:0044085	cellular component biogenesis
1.199687	4.52E-18	<0.001	GO:0000280	nuclear division
1.199687	4.52E-18	<0.001	GO:0007067	mitosis
1.169574	1.72E-17	<0.001	GO:0048285	organelle fission
1.152851	1.21E-05	0.027	GO:0022618	ribonucleoprotein complex assembly
1.144937	1.34E-05	0.027	GO:0007088	regulation of mitosis
1.144937	1.34E-05	0.027	GO:0051783	regulation of nuclear division
1.13442	2.82E-11	<0.001	GO:0006417	regulation of translation
1.131776	3.96E-09	<0.001	GO:0000151	ubiquitin ligase complex
1.122022	1.77E-05	0.032	GO:0071826	ribonucleoprotein complex subunit organization
1.063174	2.64E-17	<0.001	GO:0022403	cell cycle phase
1.041854	1.04E-05	0.018	GO:0043161	proteasomal ubiquitin-dependent protein catabolic process
1.037422	3.72E-16	<0.001	GO:0051301	cell division
1.03654	1.12E-05	0.02	GO:0010498	proteasomal protein catabolic process
1.03654	1.12E-05	0.02	GO:0031396	regulation of protein ubiquitination
1.011165	3.86E-12	<0.001	GO:0016567	protein ubiquitination
1.005735	3.31E-50	<0.001	GO:0044267	cellular protein metabolic process
0.986677	2.26E-05	0.038	GO:0003729	mRNA binding
0.97052	1.64E-11	<0.001	GO:0032446	protein modification by small protein conjugation
0.92242	1.16E-07	<0.001	GO:0007346	regulation of mitotic cell cycle
0.91639	4.44E-07	<0.001	GO:0034470	ncRNA processing
0.90864	6.23E-16	<0.001	GO:0022402	cell cycle process
0.906509	5.04E-43	<0.001	GO:0019538	protein metabolic process
0.897429	2.06E-09	<0.001	GO:0010608	posttranscriptional regulation of gene expression
0.896903	3.51E-15	<0.001	GO:0007049	cell cycle
0.884309	6.34E-19	<0.001	GO:0003723	RNA binding
0.867062	6.09E-10	<0.001	GO:0070647	protein modification by small protein conjugation or removal
0.831685	3.35E-07	<0.001	GO:0010564	regulation of cell cycle process
0.807825	8.27E-29	<0.001	GO:0043228	non-membrane-bounded organelle
0.807825	8.27E-29	<0.001	GO:0043232	intracellular non-membrane-bounded organelle
0.799349	1.58E-08	<0.001	GO:0005730	nucleolus
0.795333	5.74E-06	0.009	GO:0034660	ncRNA metabolic process
0.794772	4.95E-35	<0.001	GO:0032991	macromolecular complex
0.759191	1.34E-25	<0.001	GO:0034645	cellular macromolecule biosynthetic process
0.751417	3.45E-25	<0.001	GO:0009059	macromolecule biosynthetic process
0.68865	2.77E-28	<0.001	GO:0044260	cellular macromolecule metabolic process
0.677292	3.34E-07	<0.001	GO:0051726	regulation of cell cycle
0.672809	7.20E-14	<0.001	GO:0005622	intracellular
0.634083	1.27E-19	<0.001	GO:0044249	cellular biosynthetic process
0.630665	2.51E-24	<0.001	GO:0043170	macromolecule metabolic process
0.623944	2.67E-19	<0.001	GO:0009058	biosynthetic process
0.58448	6.61E-08	<0.001	GO:0032268	regulation of cellular protein metabolic process
0.556683	2.64E-10	<0.001	GO:0044428	nuclear part
0.55154	1.16E-19	<0.001	GO:0044237	cellular metabolic process
0.545761	1.79E-16	<0.001	GO:0044446	intracellular organelle part
0.5348	6.12E-16	<0.001	GO:0044422	organelle part
0.533598	5.47E-07	<0.001	GO:0051246	regulation of protein metabolic process
0.523919	7.39E-18	<0.001	GO:0044238	primary metabolic process
0.509038	4.91E-10	<0.001	GO:0071841	cellular component organization or biogenesis at cellular level

0.490454	4.23E-08	<0.001	GO:0006464	protein modification process
0.46879	1.34E-07	<0.001	GO:0043412	macromolecule modification
0.453634	4.73E-14	<0.001	GO:0008152	metabolic process
0.452783	4.46E-06	0.007	GO:0006996	organelle organization
0.436349	6.45E-12	<0.001	GO:0044444	cytoplasmic part
0.419423	6.73E-08	<0.001	GO:0071840	cellular component organization or biogenesis
0.390999	8.33E-06	0.013	GO:0071842	cellular component organization at cellular level
0.39013	7.50E-11	<0.001	GO:0044424	intracellular part
0.384815	4.12E-09	<0.001	GO:0005737	cytoplasm
0.361587	3.89E-06	0.006	GO:0043234	protein complex
0.351998	1.40E-05	0.027	GO:0003676	nucleic acid binding
0.289094	1.07E-06	0.001	GO:0043229	intracellular organelle
0.287773	1.19E-06	0.001	GO:0043226	organelle
0.261417	9.36E-06	0.015	GO:0009987	cellular process

Additionally, this analysis was also performed instead of using an expansion model by restricting interactions specifically only amongst the query entries. Figure E4 shows an example of an up-regulated direct interaction cluster found in the condensed network analysis.

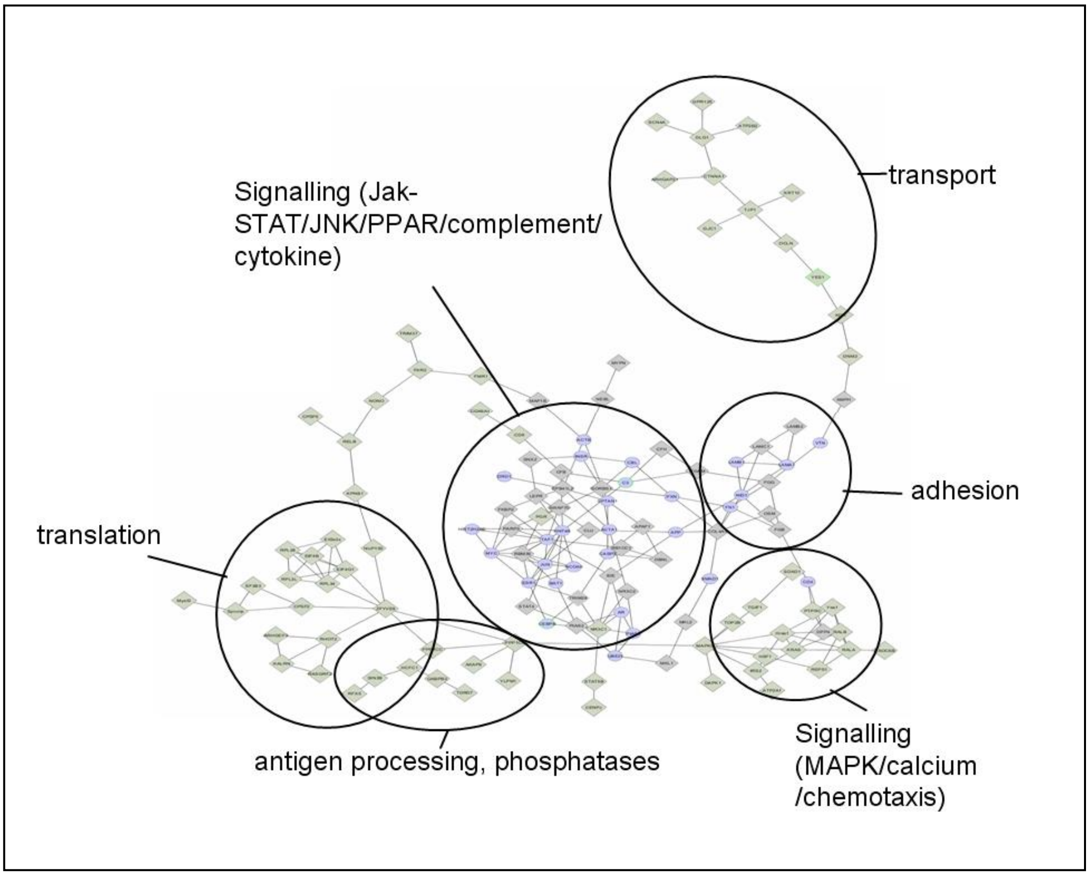

Figure E4. Condensed protein interaction cluster up-regulated in AKI. Protein interactions were mapped using MiMI and functional clusters identified based on their shared associated terminologies such as GO terms.

The mapping of proteins which could be clustered in this way were then also analysed by GO term associations and the results are listed in Table E5.

Table E5. GO term associations of direct protein interaction clusters. The GO analysis of all clustered molecules is shown for both up- and down-regulated sub-groups. The LOD value is the logarithm (base 10) of the odds ratio, and statistical significance for each GO term (listed by attribute ID and name) is provided by the p-value, and the resampling adjusted p-value.

LOD	p-value	adjusted p-value	attribute ID	attribute name
AKI up-regulated interactome cluster				
2.173522	4.41E-06	0.005	GO:0005980	glycogen catabolic process
2.173522	4.41E-06	0.005	GO:0009251	glucan catabolic process
2.173522	4.41E-06	0.005	GO:0044247	cellular polysaccharide catabolic process
1.606059	5.53E-07	0.001	GO:0005977	glycogen metabolic process
1.606059	5.53E-07	0.001	GO:0006073	cellular glucan metabolic process
1.606059	5.53E-07	0.001	GO:0044042	glucan metabolic process
1.524254	8.69E-09	<0.001	GO:0015980	energy derivation by oxidation of organic compounds
1.521223	1.34E-06	0.002	GO:0006112	energy reserve metabolic process
1.479262	2.09E-06	0.002	GO:0003743	translation initiation factor activity
1.474161	1.98E-07	0.001	GO:0000502	proteasome complex
1.450246	2.84E-06	0.005	GO:0044264	cellular polysaccharide metabolic process
1.381163	5.88E-06	0.007	GO:0006413	translational initiation
1.365362	9.03E-08	<0.001	GO:0005923	tight junction
1.365362	9.03E-08	<0.001	GO:0070160	occluding junction
1.333634	1.17E-06	0.002	GO:0043197	dendritic spine
1.327667	1.26E-06	0.002	GO:0008135	translation factor activity, nucleic acid binding
1.315972	1.46E-06	0.002	GO:0044309	neuron spine
1.303373	2.25E-07	0.001	GO:0014069	postsynaptic density
1.281624	1.68E-05	0.02	GO:0035023	regulation of Rho protein signal transduction
1.204624	9.61E-07	0.002	GO:0046578	regulation of Ras protein signal transduction
1.09921	4.46E-06	0.005	GO:0043523	regulation of neuron apoptosis
1.09921	4.46E-06	0.005	GO:0051056	regulation of small GTPase mediated signal transduction
1.053323	8.65E-06	0.011	GO:0045211	postsynaptic membrane
1.03466	1.13E-05	0.015	GO:0008022	protein C-terminus binding
1.019271	1.41E-05	0.017	GO:0032870	cellular response to hormone stimulus
0.999553	1.87E-05	0.022	GO:0097060	synaptic membrane
0.962034	6.35E-17	<0.001	GO:0008150	biological_process
0.959246	2.44E-06	0.004	GO:0042598	vesicular fraction
0.951617	1.01E-05	0.014	GO:0005911	cell-cell junction
0.942995	3.47E-16	<0.001	GO:0003674	molecular_function
0.93171	1.18E-06	0.002	GO:0051301	cell division
0.918781	1.70E-05	0.02	GO:0005792	microsome
0.903697	1.28E-13	<0.001	GO:0005575	cellular_component
0.884942	2.91E-05	0.047	GO:0051259	protein oligomerization
0.862648	9.95E-20	<0.001	GO:0005488	binding
0.852001	3.58E-13	<0.001	GO:0000267	cell fraction

0.849193	1.73E-05	0.021	GO:0008283	cell proliferation
0.842008	3.10E-07	0.001	GO:0043066	negative regulation of apoptosis
0.837532	3.47E-07	0.001	GO:0043069	negative regulation of programmed cell death
0.837499	1.56E-10	<0.001	GO:0005626	insoluble fraction
0.832159	2.33E-05	0.044	GO:0044456	synapse part
0.823248	2.72E-05	0.045	GO:0032403	protein complex binding
0.819203	5.47E-07	0.001	GO:0060548	negative regulation of cell death
0.809308	2.20E-21	<0.001	GO:0005515	protein binding
0.808113	2.88E-09	<0.001	GO:0005624	membrane fraction
0.806813	1.93E-06	0.002	GO:0007049	cell cycle
0.801551	3.32E-07	0.001	GO:0046483	heterocycle metabolic process
0.7792	1.03E-07	<0.001	GO:0006468	protein phosphorylation
0.773519	6.93E-07	0.002	GO:0030054	cell junction
0.772549	1.03E-05	0.014	GO:0043005	neuron projection
0.766997	1.74E-12	<0.001	GO:0044464	cell part
0.753919	6.35E-06	0.007	GO:0071310	cellular response to organic substance
0.726346	2.65E-05	0.045	GO:0019904	protein domain specific binding
0.720969	1.62E-14	<0.001	GO:0009987	cellular process
0.717319	3.18E-05	0.048	GO:0004672	protein kinase activity
0.716737	3.56E-17	<0.001	GO:0008152	metabolic process
0.707652	3.75E-06	0.005	GO:0005829	cytosol
0.698504	6.35E-08	<0.001	GO:0010033	response to organic substance
0.69726	6.63E-08	<0.001	GO:0006796	phosphate metabolic process
0.696433	6.83E-08	<0.001	GO:0006793	phosphorus metabolic process
0.696272	5.62E-07	0.001	GO:0016310	phosphorylation
0.696023	5.03E-06	0.006	GO:0048584	positive regulation of response to stimulus
0.690497	1.18E-14	<0.001	GO:0044424	intracellular part
0.688661	3.52E-07	0.001	GO:0051246	regulation of protein metabolic process
0.684577	4.01E-07	0.001	GO:0005102	receptor binding
0.67584	1.40E-15	<0.001	GO:0044237	cellular metabolic process
0.672862	6.76E-09	<0.001	GO:0048583	regulation of response to stimulus
0.672826	1.84E-15	<0.001	GO:0044238	primary metabolic process
0.667432	5.14E-06	0.006	GO:0035556	intracellular signal transduction
0.65662	1.02E-11	<0.001	GO:0048518	positive regulation of biological process
0.649287	8.25E-06	0.01	GO:0016772	transferase activity, transferring phosphorus-containing groups
0.646751	8.81E-06	0.011	GO:0032268	regulation of cellular protein metabolic process
0.642987	5.13E-06	0.006	GO:0042981	regulation of apoptosis
0.640648	5.47E-06	0.006	GO:0042995	cell projection
0.638321	5.83E-06	0.007	GO:0043067	regulation of programmed cell death
0.631338	2.26E-08	<0.001	GO:0042221	response to chemical stimulus
0.626946	5.86E-09	<0.001	GO:0044267	cellular protein metabolic process
0.624596	8.48E-06	0.01	GO:0010941	regulation of cell death
0.624477	2.90E-05	0.047	GO:0022607	cellular component assembly
0.623084	1.62E-05	0.019	GO:0019899	enzyme binding
0.611146	1.26E-09	<0.001	GO:0019538	protein metabolic process
0.610545	9.05E-08	<0.001	GO:0032555	purine ribonucleotide binding
0.610274	9.16E-08	<0.001	GO:0032553	ribonucleotide binding
0.60865	9.78E-08	<0.001	GO:0017076	purine nucleotide binding
0.598266	3.03E-05	0.048	GO:0010557	positive regulation of macromolecule biosynthetic process
0.597151	1.01E-05	0.014	GO:0009966	regulation of signal transduction
0.596542	2.61E-07	0.001	GO:0035639	purine ribonucleoside triphosphate binding
0.594784	2.15E-06	0.002	GO:0023051	regulation of signaling
0.594464	1.90E-05	0.041	GO:0009891	positive regulation of biosynthetic process

0.593831	7.89E-12	<0.001	GO:0043170	macromolecule metabolic process
0.587312	3.16E-11	<0.001	GO:0044260	cellular macromolecule metabolic process
0.578295	1.57E-08	<0.001	GO:0048522	positive regulation of cellular process
0.572375	1.69E-06	0.002	GO:0006464	protein modification process
0.566822	5.31E-06	0.006	GO:0065008	regulation of biological quality
0.565978	1.45E-07	0.001	GO:0000166	nucleotide binding
0.56073	2.51E-08	<0.001	GO:0043234	protein complex
0.560674	2.53E-06	0.004	GO:0016740	transferase activity
0.557642	1.28E-10	<0.001	GO:0044444	cytoplasmic part
0.555871	1.76E-10	<0.001	GO:0003824	catalytic activity
0.550898	3.54E-06	0.005	GO:0043412	macromolecule modification
0.534585	2.43E-08	<0.001	GO:0060255	regulation of macromolecule metabolic process
0.529454	2.72E-10	<0.001	GO:0065007	biological regulation
0.521955	6.36E-08	<0.001	GO:0032991	macromolecular complex
0.520465	5.01E-10	<0.001	GO:0050789	regulation of biological process
0.518679	2.38E-05	0.044	GO:0044281	small molecule metabolic process
0.515399	4.31E-07	0.001	GO:0044249	cellular biosynthetic process
0.503069	4.37E-08	<0.001	GO:0019222	regulation of metabolic process
0.496396	9.97E-07	0.002	GO:0009058	biosynthetic process
0.493996	2.57E-07	0.001	GO:0006807	nitrogen compound metabolic process
0.493801	8.95E-09	<0.001	GO:0050896	response to stimulus
0.490923	4.21E-09	<0.001	GO:0043231	intracellular membrane-bounded organelle
0.490514	4.33E-09	<0.001	GO:0043227	membrane-bounded organelle
0.489126	4.28E-07	0.001	GO:0034641	cellular nitrogen compound metabolic process
0.488558	4.07E-08	<0.001	GO:0005737	cytoplasm
0.488223	5.02E-09	<0.001	GO:0043229	intracellular organelle
0.486907	5.50E-09	<0.001	GO:0043226	organelle
0.481669	6.10E-07	0.002	GO:0080090	regulation of primary metabolic process
0.476062	7.95E-07	0.002	GO:0031323	regulation of cellular metabolic process
0.463628	4.04E-06	0.005	GO:0006139	nucleobase, nucleoside, nucleotide and nucleic acid metabolic process
0.461024	5.93E-06	0.007	GO:0032502	developmental process
0.459454	3.66E-07	0.001	GO:0051716	cellular response to stimulus
0.459174	2.75E-05	0.045	GO:0048519	negative regulation of biological process
0.429946	2.52E-07	0.001	GO:0050794	regulation of cellular process
0.397175	1.30E-05	0.016	GO:0005634	nucleus
AKI down-regulated interactome cluster				
3.237431	2.29E-06	0.003	GO:0005680	anaphase-promoting complex
3.131558	3.63E-06	0.004	GO:0000152	nuclear ubiquitin ligase complex
3.046478	5.26E-06	0.005	GO:0070979	protein K11-linked ubiquitination
2.575075	4.34E-05	0.016	GO:0031461	cullin-RING ubiquitin ligase complex
2.390822	9.98E-05	0.024	GO:0000209	protein polyubiquitination
2.345944	0.000122	0.027	GO:0000151	ubiquitin ligase complex
2.013206	4.17E-05	0.016	GO:0044267	cellular protein metabolic process
1.901769	0.000106	0.024	GO:0019538	protein metabolic process

Interactome cluster analysis for the AKI up-regulated dataset showed specific clustering for proteins of the proteasome, apoptosis cluster, clusters involved in gene activation and translation, cytoskeletal re-organisation, several signalling pathways (MAPK, ErbB, EGF, PPAR and VEGF) and cell division/DNA replication. Clusters from proteins only found in the AKI samples show additionally to include metabolic

processes (glycolysis, purine and pyrimidine biosynthesis/degradation), calcium signalling and complement activation. Clusters found in the down-regulated dataset are, as mentioned above, involved in exocytosis. An added benefit of this analysis is also that a list of proteins can be generated which can be used for specific pathway mapping (see below).

Pathway mapping onto existing pathways (KEGG, PathVisio(WikiPathways))

An important step in large-scale data analysis is to explore the dataspace of known and pre-assembled molecular pathways. However, databases holding such collections are very sparse and for obvious reasons can not provide a comprehensive assembly of known/described pathways found in the literature. Therefore it is important to take any results derived from such a mapping effort more as a guide rather than a finite outcome. Additionally, the naming of pre-assembled pathways can also be misleading, and should not be taken at face value and the focus should be on the molecular entities contained within a pathway itself. This is best exemplified by a hypothetical result where only one branch of a multi-faceted 20 branch pathway could be mapped to near completion using a query-dataset. Statistically the whole pathway might appear to be of significance, but only closer (manual) inspection would reveal that only a small proportion is of relevance. And therefore the analysis should dismiss the pathway *per se*, but retain the side-branch to be assembled and combined into a new pathway with other such smaller cascades.

Pathway mapping was done by KEGG database searching (http://www.genome.jp/kegg/tool/map_pathway1.html) using all significant up-regulated proteins, as well as statistical testing of WikiPathways (http://wikipathways.org/index.php/WikiPathways) using PathVisio (http://www.pathvisio.org/) and the MIM plug-in (http://discover.nci.nih.gov/mim/mim_pathvisio.html) of the entire significant dataset. The species restriction was set to mouse, and query-lists consisted of either KEGG or EnsEMBL accession numbers respectively. Table E6 lists the combined results of these searches.

Table E6. Pathway analysis using PathVisio (WikiPathways) and KEGG. All significant AKI-modulated proteins were used to search the WikiPathway database using PathVisio or only the up-regulated dataset to query the online version of the KEGG database. Hits are listed according to positive hits and for PathVisio the measured (fold-change cross-correlation significant) hits. The PathVisio total values are the total number of elements in the pathway queried, and the % is the total number of hits versus consistent hits. The Z-score is the Z-statistic of the queried molecules in comparison to the total number of entries in the specified pathway. The statistics were calculated based on fold changes >2 and p-values <0.05.

pathway name	KEGG pathway ID	PathVisio positive	PathVisio measured	PathVisio total	PathVisio %	number of KEGG hits	Z-score	molecules
Metabolic pathways	mmu01100	-	-	-	-	79	-	Amy2a4; Amy2a3; Amy2a2; cytochrome c oxidase subunit 7C, mitochondrial-like; Pafah2; Cyp27a1; Nt5c3; Atic; Amy2a5; Pygb; Pygl; Impa2; Adsl; Bhmt; Cbr2; Cox6a1; Cox7c; Cyp1a1; Cyp1a2; Cyp2j5; Dgka; G6pc; Gaa; Galnt3; Gk2; H2-Ke6; Itpa; Alad; Inpp5j; Amacr; Man2a1; Ndst2; ND1; Plcb2; Plcb4; Ptgis; Chpt1; Tbxas1; Nags; Tpo; Umps; Urod; Pik3c3; Cps1; Man1b1; Polr2b; Csgalnact1; Galnt5; Sptlc1; Mecr; Pla2g2e; Agpat3; Tpk1; Cyp2c54; Nt5c; Acsl4; St6galnac6; Cdipt; Treh; Dpys; Alg5; 4933434I20Rik; Atp5g2; Gnpda2; Gmppa; Cers5; Tm7sf2; Plce1; Gbe1; Pgs1; Cyp2j9; Pck2; Dhcr24; Nt5c2; Pla2g4d; Chsy3; Nme3; Ndst3; Rdh10
Pathways in cancer	mmu05200	31	31	372	100.00%	30	0.68	Birc3; Araf; Ctnna1; Ctnna2; Cdk2; Cdk4; Cdk6; Col4a1; Col4a3; Csf2ra; Egfr; Fgfr1; Fgfr2; Gli2; Gli3; Hras1; Kras; Lama3; Lamb2; Mmp2; Msh2; Pparg; Stat5b; Tgfb1; Wnt6; Lamc3; Rala; Ralb; Dapk1; Cul2
PI3K-Akt signaling pathway	mmu04151	-	-	-	-	29	-	Pkn2; Itga1; Cdk2; Cdk4; Cdk6; Col4a1; Col4a3; Col1a2; Egfr; Eif4e; Fgfr1; Fgfr2; Fgfr4; G6pc; Hras1; Jak3; Kras; Lama3; Lamb2; Osm; Creb3l2; Ppp2r5d; Thbs2; Lamc3; Ppp2r5c; Eif4e2; Col24a1; Pck2; Eif4b
MAPK signaling pathway	mmu04010	26	26	279	100.00%	21	0.62	Cacnb1; Egfr; Fgfr1; Fgfr2; Fgfr4; Hras1; Hspb1; Kras; Ppm1a; Ppp3cb; Mapk11; Rasgrf2; Relb; Tgfb1; Tnfrsf1a; Mapk13; Mapk8ip3; Map3k14; Map3k13; Rps6ka5; Pla2g4d
Regulation of actin cytoskeleton	mmu04810	22	22	226	100.00%	20	0.57	Itga1; Araf; Diap1; Egfr; Enah; Fgfr1; Fgfr2; Fgfr4; Hras1; Kras; Ppp1r12a; Ppp1ca; Ppp1cc; Pxn; Scin; Ssh2; Nckap1; Arhgef6; Cyfip2; Wasf1
EGFR1 Signaling Pathway	-	21	21	177	100.00%	-	0.55	-
Insulin Signaling	-	21	21	160	100.00%	-	0.55	-
PluriNetWork	-	21	21	291	100.00%	-	0.55	-
Insulin signaling pathway	mmu04910	20	20	148	100.00%	19	0.54	Pygb; Pygl; Araf; Socs3; Eif4e; Ppp1r3a; Flot1; G6pc; Hras1; Kras; Lipe; Ppp1ca; Ppp1cc; Ptprf; Sorbs1; Eif4e2; Shc4; Irs2; Pck2
Viral carcinogenesis	mmu05203	-	-	-	-	20	-	D1Pas1; Cdk1; Cdk2; Cdk4; Cdk6; Chek1; Ccr8; Ddx3x; Dlg1; Gtf2h1; Hras1; Jak3; Kras; Lyn; Pxn; Ranbp1; Scin; Stat5b; Creb3l2; Ddx3y
HTLV-I infection	mmu05166	-	-	-	-	20	-	Trrap; Adcy6; Bub3; Cdk4; Chek1; Dlg1; Hras1; Jak3; Kras; Lck; Mybl1; Ppp3cb; Ranbp1; Relb; Stat5b; Tgfb1; Tnfrsf1a; Wnt6; Adcy1; Map3k14

Focal adhesion	mmu04510	19	19	210	100.00%	20	0.53	Itga1; Birc3; Col4a1; Col4a3; Col1a2; Diap1; Egfr; Fyn; Hras1; Lama3; Lamb2; Ppp1r12a; Ppp1ca; Ppp1cc; Pxn; Thbs2; Lamc3; Shc4; Parva; Col24a1
Proteoglycans in cancer	mmu05205	-	-	-	-	19	-	Ank1; Araf; Col1a2; Ctsl; Egfr; Fgfr1; Hras1; Kras; Mmp2; Ppp1r12a; Ppp1ca; Ppp1cc; Mapk11; Pxn; Tgfb1; Wnt6; Mapk13; Plce1; Eif4b
Axon guidance	mmu04360	18	18	136	100.00%	16	0.51	Epha7; Ephb6; Fyn; Hras1; Kras; Ntng2; Plxna1; Ppp3cb; Sema3a; Sema3c; Sema6a; Slit3; Unc5d; Plxna4; Ngef; Plxnc1
Endocytosis	mmu04144	18	18	235	100.00%	14	0.51	Dnm2; Egfr; Ehd1; Fgfr2; Fgfr4; Grk4; Hras1; Pdcd6ip; Tgfb1; Ap2s1; Rab11fip5; Sh3kbp1; Vps36; Stambp
Focal Adhesion	-	18	18	192	100.00%	-	0.51	-
Chemokine signaling pathway	mmu04062	17	17	198	100.00%	15	0.5	Adcy6; Ccr8; Fgr; Grk4; Hck; Hras1; Jak3; Kras; Lyn; Plcb2; Plcb4; Pxn; Stat5b; Shc4; Adcy1
Amoebiasis	mmu05146	16	16	151	100.00%	15	0.48	Col4a1; Col4a3; Col1a2; Gna15; Hspb1; Lama3; Lamb2; Plcb2; Plcb4; Serpinb9f; Serpinb9; Tgfb1; Lamc3; Adcy1; Col24a1
Regulation of Actin Cytoskeleton	-	16	16	157	100.00%	-	0.48	-
Epstein-Barr virus infection	mmu05169	-	-	-	-	16	-	Cdk1; Cdk2; Fgr; Hspb1; Jak3; Lyn; Mapk11; Ptma; Relb; Polr2b; Mapk13; Map3k14; Psmd8; Psmd14; Psmd6; Psmd1
Calcium signaling pathway	mmu04020	15	15	195	100.00%	14	0.47	Slc8a3; Atp2a1; Atp2b2; Egfr; Gna15; Grin1; Grm1; Ntsr1; Plcb2; Plcb4; Ppp3cb; Stim1; Adcy1; Plce1
Osteoclast differentiation	mmu04380	15	15	124	100.00%	15	0.47	Socs3; Fhl2; Fyn; Lck; Ncf2; Tnfrsf11b; Pparg; Ppp3cb; Mapk11; Relb; Tec; Tgfb1; Tnfrsf1a; Mapk13; Map3k14
Adipogenesis	-	15	15	133	100.00%	-	0.47	-
T Cell Receptor Signaling Pathway	mmu04660	15	15	134	100.00%	11	0.47	Cdk4; Dlg1; Fyn; Hras1; Kras; Lck; Ppp3cb; Mapk11; Tec; Mapk13; Map3k14
Adherens junction	mmu04520	14	14	81	100.00%	14	0.45	Ctnna1; Ctnna2; Egfr; Fert2; Fgfr1; Fyn; Ptprb; Ptprf; Ptprj; Pvrl2; Sorbs1; Tjp1; Yes1; Wasf1
Cell cycle	mmu04110	14	14	131	100.00%	12	0.45	Bub3; Cdk1; Cdk2; Cdk4; Cdk6; Chek1; Mcm2; Mcm7; Tgfb1; Zbtb17; Cul1; Orc3
Glutamatergic synapse	mmu04724	14	14	144	100.00%	12	0.45	Adcy6; Gria2; Grin1; Grm1; Plcb2; Plcb4; Ppp3cb; Slc17a8; Homer1; Homer3; Adcy1; Pla2g4d
RNA transport	mmu03013	14	14	183	100.00%	13	0.45	4930444G20Rik; Eif2s1; Eif4e; Kpnb1; Nup155; Eif4a3; Eif4g1; Eif2s3x; Eif4e2; Eif4b; Cyfip2; Nup210l; Wibg
Tight junction	mmu04530	14	14	141	100.00%	12	0.45	Ctnna1; Ctnna2; Cdk4; Epb4.1l2; Hras1; Kras; Mpdz; Ocln; Tjp1; Yes1; Cldn8; Magi3
Ubiquitin mediated proteolysis	mmu04120	14	14	144	100.00%	12	0.45	Aire; Birc3; Socs3; Rnf7; Ube2o; Ube2m; Rfwd2; Cul1; Ppil2; Trim37; Cul2; Cul5
Cytokine-cytokine receptor interaction	mmu04060	13	13	250	100.00%	13	0.43	Ccr8; Csf2ra; Ctf1; Egfr; Lepr; Lifr; Tnfrsf11b; Osm; Tgfb1; Tnfrsf1a; Tpo; Tnfsf4; Ifnlr1
GnRH signaling pathway	mmu04912	13	13	109	100.00%	11	0.43	Adcy6; Egfr; Hras1; Kras; Mmp2; Plcb2; Plcb4; Mapk11; Mapk13; Adcy1; Pla2g4d
Huntington's disease	mmu05016	13	13	211	100.00%	11	0.43	cytochrome c oxidase subunit 7C, mitochondrial-like; Cox6a1; Cox7c; Grin1; Plcb2; Plcb4; Pparg; Creb3l2; Polr2b; Ap2s1; Atp5g2
Long-term potentiation	mmu04720	13	13	79	100.00%	12	0.43	Araf; Gria2; Grin1; Grm1; Hras1; Kras; Plcb2; Plcb4; Ppp1ca; Ppp1cc; Ppp3cb; Adcy1
Purine metabolism	mmu00230	13	13	322	100.00%	12	0.43	Nt5c3; Atic; Adcy6; Adsl; Itpa; Polr2b; Pde5a; Adcy1; Nt5c; Nudt2; Nt5c2; Nme3
B Cell Receptor Signaling Pathway	mmu04662	13	13	157	100.00%	4	0.43	Hras1; Kras; Lyn; Ppp3cb
IL-3 Signaling Pathway	-	13	13	101	100.00%	-	0.43	-

IL-6 signaling Pathway	-	13	13	100	100.00%	-	0.43	-
Alzheimer's disease	mmu05010	12	12	200	100.00%	10	0.42	cytochrome c oxidase subunit 7C, mitochondrial-like; Atp2a1; Cox6a1; Cox7c; Grin1; Plcb2; Plcb4; Ppp3cb; Tnfrsf1a; Atp5g2
Gap junction	mmu04540	12	12	104	100.00%	10	0.42	Adcy6; Cdk1; Egfr; Grm1; Hras1; Kras; Plcb2; Plcb4; Tjp1; Adcy1
Hepatitis C	mmu05160	12	12	154	100.00%	12	0.42	Araf; Socs3; Egfr; Eif2s1; Hras1; Kras; Ocln; Mapk11; Tnfrsf1a; Nr1h3; Mapk13; Cldn8
Long-term depression	mmu04730	12	12	83	100.00%	9	0.42	Araf; Gria2; Grm1; Hras1; Kras; Lyn; Plcb2; Plcb4; Pla2g4d
T cell receptor signaling pathway	-	12	12	123	100.00%	-	0.42	-
Toxoplasmosis	mmu05145	12	12	148	100.00%	9	0.42	Birc3; Lama3; Lamb2; Mapk11; Tgfb1; Tnfrsf1a; Lamc3; Mapk13; Irak4
Cell cycle	-	12	12	90	100.00%	-	0.42	-
Kit Receptor Signaling Pathway	-	12	12	67	100.00%	-	0.42	-
Estrogen signaling pathway	mmu04915	-	-	-	-	12	-	Adcy6; Egfr; Esr2; Grm1; Hras1; Kras; Mmp2; Plcb2; Plcb4; Creb3l2; Shc4; Adcy1
Dopaminergic synapse	mmu04728	-	-	-	-	12	-	Gria2; Kif5b; Plcb2; Plcb4; Ppp1ca; Ppp1cc; Ppp3cb; Mapk11; Creb3l2; Ppp2r5d; Mapk13; Ppp2r5c
Hepatitis B	mmu05161	-	-	-	-	12	-	D1Pas1; Cdk2; Cdk4; Cdk6; Ddx3x; Hras1; Kras; Stat4; Stat5b; Creb3l2; Tgfb1; Ddx3y
Arachidonic acid metabolism	mmu00590	11	11	179	100.00%	8	0.4	Cbr2; Cyp2j5; Ptgis; Tbxas1; Pla2g2e; Cyp2c54; Cyp2j9; Pla2g4d
Jak-STAT signaling pathway	mmu04630	11	11	158	100.00%	11	0.4	Socs3; Csf2ra; Ctf1; Jak3; Lepr; Lifr; Osm; Stat4; Stat5b; Tpo; Ifnlr1
Leukocyte transendothelial migration	mmu04670	11	11	126	100.00%	9	0.4	Ctnna1; Ctnna2; Mmp2; Ncf2; Ocln; Mapk11; Pxn; Mapk13; Cldn8
Oocyte meiosis	mmu04114	11	11	122	100.00%	10	0.4	Adcy6; Cdk1; Cdk2; Ppp1ca; Ppp1cc; Ppp3cb; Ppp2r5d; Ppp2r5c; Cul1; Adcy1
Vascular smooth muscle contraction	mmu04270	11	11	146	100.00%	10	0.4	Adcy6; Araf; Ppp1r12a; Plcb2; Plcb4; Ppp1ca; Ppp1cc; Pla2g2e; Adcy1; Pla2g4d
Wnt signaling pathway	mmu04310	11	11	165	100.00%	10	0.4	Prickle1; 4930444G20Rik; Plcb2; Plcb4; Ppp3cb; Wnt6; Cul1; Ruvbl1; Bambi; Vangl2
MAPK signaling pathway	-	11	11	162	100.00%	-	0.4	-
Retrograde endocannabinoid signaling	mmu04723	-	-	-	-	11	-	Adcy6; Gabra1; Gabrd; Gria2; Grm1; Plcb2; Plcb4; Mapk11; Slc17a8; Mapk13; Adcy1
ABC transporters	mmu02010	10	10	397	100.00%	8	0.38	Abcb7; Abca8a; Abcc4; Abcg5; Abcb11; Abcb10; Abcb8; Abcc3
Chagas disease (American trypanosomiasis)	mmu05142	10	10	121	100.00%	10	0.38	Ace; Gna15; Plcb2; Plcb4; Mapk11; Tgfb1; Tnfrsf1a; Mapk13; Irak4; Adcy1
Natural killer cell mediated cytotoxicity	mmu04650	10	10	140	100.00%	10	0.38	Araf; Fyn; Hras1; Klra4; Klra7; Kras; Lck; Ncr1; Ppp3cb; Shc4
Olfactory transduction	mmu04740	10	10	1019	100.00%	8	0.38	Olfr867; Olfr1211; Olfr1495; Olfr806; Olfr1033; Olfr695; Olfr134; Olfr181
p53 signaling pathway	mmu04115	10	10	73	100.00%	8	0.38	Cdk1; Cdk2; Cdk4; Cdk6; Chek1; Rfwd2; Ppm1d; Lrdd
Pancreatic cancer	mmu05212	10	10	81	100.00%	9	0.38	Araf; Cdk4; Cdk6; Egfr; Kras; Tgfb1; Rala; Ralb; Arhgef6
Small cell lung cancer	mmu05222	10	10	94	100.00%	9	0.38	Birc3; Cdk2; Cdk4; Cdk6; Col4a1; Col4a3; Lama3; Lamb2; Lamc3
Tuberculosis	mmu05152	10	10	205	100.00%	8	0.38	Ppp3cb; Mapk11; Tgfb1; Tnfrsf1a; Pik3c3; Mapk13; Irak4; Card9
IL-2 Signaling Pathway	-	10	10	76	100.00%	-	0.38	-
metapathway biotransformation	-	10	10	144	100.00%	-	0.38	-

Hippo signaling pathway	mmu04390	-	-	-	-	10	-	Bmp8a; Ctnna1; Ctnna2; Dlg1; Gli2; Ppp1ca; Ppp1cc; Wwc1; Tgfb1; Wnt6
Non-alcoholic fatty liver disease (NAFLD)	mmu04932	-	-	-	-	10	-	cytochrome c oxidase subunit 7C, mitochondrial-like; Socs3; Cox6a1; Cox7c; Eif2s1; Lepr; Tgfb1; Tnfrsf1a; Nr1h3; Irs2
Herpes simplex infection	mmu05168	-	-	-	-	10	-	Cdk1; Cdk2; Socs3; Eif2s1; Per3; Ppp1ca; Ppp1cc; Pvrl2; Tnfrsf1a; Cul1
Bladder cancer	mmu05219	9	9	50	100.00%	8	0.36	Araf; Cdk4; Egfr; Hras1; Kras; Mmp2; Dapk1; Rps6ka5
Chronic myeloid leukemia	mmu05220	9	9	81	100.00%	8	0.36	Araf; Cdk4; Cdk6; Hras1; Kras; Stat5b; Tgfb1; Shc4
Complement and coagulation cascades	mmu04610	9	9	80	100.00%	7	0.36	C3ar1; F10; F7; Fga; Kng1; Gm5077; Fgg
ECM-receptor interaction	mmu04512	9	9	88	100.00%	9	0.36	Itga1; Col4a1; Col4a3; Col1a2; Lama3; Lamb2; Thbs2; Lamc3; Col24a1
Fc epsilon RI signaling pathway	mmu04664	9	9	95	100.00%	7	0.36	Fyn; Hras1; Kras; Lyn; Mapk11; Mapk13; Pla2g4d
Measles	mmu05162	9	9	154	100.00%	8	0.36	Cdk2; Cdk4; Cdk6; Eif2s1; Fyn; Jak3; Stat5b; Irak4
Phagosome	mmu04145	9	9	192	100.00%	8	0.36	Dync2h1; Ctsl; Marco; Mpo; Ncf2; Sec22b; Thbs2; Pik3c3
Progesterone-mediated oocyte maturation	mmu04914	9	9	93	100.00%	8	0.36	Adcy6; Araf; Cdk1; Cdk2; Kras; Mapk11; Mapk13; Adcy1
Pyrimidine metabolism	mmu00240	9	9	192	100.00%	9	0.36	Nt5c3; Itpa; Umps; Polr2b; Nt5c; Dpys; Nudt2; Nt5c2; Nme3
VEGF signaling pathway	mmu04370	9	9	87	100.00%	8	0.36	Hras1; Hspb1; Kras; Ppp3cb; Mapk11; Pxn; Mapk13; Pla2g4d
Alpha6-Beta4 Integrin Signaling Pathway	-	9	9	67	100.00%	-	0.36	-
Integrin-mediated cell adhesion	-	9	9	102	100.00%	-	0.36	-
Non-odorant GPCRs	-	9	9	267	100.00%	-	0.36	-
Wnt Signaling Pathway NetPath	-	9	9	110	100.00%	-	0.36	-
Serotonergic synapse	mmu04726	-	-	-	-	9	-	Araf; Cyp2j5; Hras1; Kras; Plcb2; Plcb4; Cyp2c54; Cyp2j9; Pla2g4d
TNF signaling pathway	mmu04668	-	-	-	-	9	-	Birc3; Socs3; Mapk11; Creb3l2; Tnfrsf1a; Mapk13; Map3k14; Rps6ka5; Mlkl
Circadian entrainment	mmu04713	-	-	-	-	9	-	Adcy6; Adcyap1r1; Gria2; Grin1; Per3; Plcb2; Plcb4; Adcy1; Rps6ka5
Cholinergic synapse	mmu04725	-	-	-	-	9	-	Adcy6; Fyn; Hras1; Kras; Plcb2; Plcb4; Creb3l2; Kcnq5; Adcy1
NF-kappa B signaling pathway	mmu04064	-	-	-	-	9	-	Birc3; Bcl2a1a; Lck; Lyn; Relb; Tnfrsf1a; Irak4; Map3k14; Lrdd
Aminoacyl-tRNA biosynthesis	mmu00970	8	8	136	100.00%	6	0.34	Rars2; Wars; Aars2; Aars; Tarsl2; Tars2
Cell adhesion molecules (CAMs)	mmu04514	8	8	163	100.00%	6	0.34	Ntng2; Ocln; Ptprf; Pvrl2; Nlgn2; Cldn8
Fc gamma R-mediated phagocytosis	mmu04666	8	8	106	100.00%	6	0.34	Dnm2; Hck; Lyn; Scin; Amph; Wasf1
Glioma	mmu05214	8	8	77	100.00%	7	0.34	Araf; Cdk4; Cdk6; Egfr; Hras1; Kras; Shc4
Linoleic acid metabolism	mmu00591	8	8	81	100.00%	6	0.34	Cyp1a2; Cyp2j5; Pla2g2e; Cyp2c54; Cyp2j9; Pla2g4d
Lysosome	mmu04142	8	8	134	100.00%	7	0.34	Tpp1; Ctsl; Ctsw; Gaa; Man2b1; Naga; Gnptab
Melanogenesis	mmu04916	8	8	111	100.00%	8	0.34	Adcy6; Hras1; Kras; Plcb2; Plcb4; Creb3l2; Wnt6; Adcy1
Melanoma	mmu05218	8	8	79	100.00%	7	0.34	Araf; Cdk4; Cdk6; Egfr; Fgfr1; Hras1; Kras
Neurotrophin signaling	mmu04722	8	8	142	100.00%	7	0.34	Hras1; Kras; Mapk11; Mapk13; Irak4; Shc4; Rps6ka5

pathway								
Phosphatidylinositol signaling system	mmu04070	8	8	108	100.00%	8	0.34	Impa2; Dgka; Inpp5j; Plcb2; Plcb4; Pik3c3; Cdipt; Plce1
PPAR signaling pathway	mmu03320	8	8	90	100.00%	8	0.34	Cyp27a1; Gk2; Pparg; Sorbs1; Nr1h3; Acsl4; Angptl4; Pck2
Prostate cancer	mmu05215	8	8	102	100.00%	8	0.34	Araf; Cdk2; Egfr; Fgfr1; Fgfr2; Hras1; Kras; Creb3l2
Retinol metabolism	mmu00830	8	8	109	100.00%	5	0.34	Cyp1a1; Cyp1a2; Cyp2c54; Retsat; Rdh10
Staphylococcus aureus infection	mmu05150	8	8	85	100.00%	5	0.34	C3ar1; Dsg1a; Dsg1b; Gm5077; Fgg
Starch and sucrose metabolism	mmu00500	8	8	151	100.00%	10	0.34	Amy2a4; Amy2a3; Amy2a2; Amy2a5; Pygb; Pygl; G6pc; Gaa; Treh; Gbe1
Calcium Regulation in the Cardiac Cell	-	8	8	154	100.00%	-	0.34	-
Endochondral Ossification	-	8	8	67	100.00%	-	0.34	-
G Protein Signaling Pathways	-	8	8	96	100.00%	-	0.34	-
IL-7 Signaling Pathway	-	8	8	45	100.00%	-	0.34	-
MicroRNAs in cardiomyocyte hypertrophy	-	8	8	109	100.00%	-	0.34	-
TGF-beta Receptor Signaling Pathway	-	8	8	152	100.00%	-	0.34	-
Alcoholism	mmu05034	-	-	-	-	8	-	Araf; Grin1; Hras1; Kras; Ppp1ca; Ppp1cc; Creb3l2; Shc4
MicroRNAs in cancer	mmu05206	-	-	-	-	8	-	Cdk6; Egfr; Hras1; Kras; Notch2; Shc4; Irs2; Rps6ka5
Influenza A	mmu05164	-	-	-	-	8	-	Socs3; Eif2s1; Mapk11; Tnfrsf1a; Prss2; Mapk13; Irak4; Nlrx1
Prolactin signaling pathway	mmu04917	-	-	-	-	8	-	Socs3; Esr2; Hras1; Kras; Mapk11; Stat5b; Mapk13; Shc4
Adipocytokine signaling pathway	mmu04920	7	7	78	100.00%	7	0.32	Socs3; G6pc; Lepr; Tnfrsf1a; Irs2; Acsl4; Pck2
Dilated cardiomyopathy	mmu05414	7	7	99	100.00%	5	0.32	Itga1; Adcy6; Cacnb1; Tgfb1; Adcy1
Inositol phosphate metabolism	mmu00562	7	7	115	100.00%	7	0.32	Impa2; Inpp5j; Plcb2; Plcb4; Pik3c3; Cdipt; Plce1
Non-small cell lung cancer	mmu05223	7	7	66	100.00%	6	0.32	Araf; Cdk4; Cdk6; Egfr; Hras1; Kras
Proteasome	mmu03050	7	7	51	100.00%	7	0.32	Psme1; Psme2; Psma1; Psmd8; Psmd14; Psmd6; Psmd1
Ribosome biogenesis in eukaryotes	mmu03008	7	7	98	100.00%	7	0.32	Pwp2; Eif6; Tcof1; Sbds; Rexo1; Nop56; Rbm28
RIG-I-like receptor signaling pathway	mmu04622	7	7	76	100.00%	7	0.32	D1Pas1; Ddx3x; Mapk11; Mapk13; Ddx3y; Nlrx1; Tmem173
Spliceosome	mmu03040	7	7	148	100.00%	5	0.32	Eif4a3; Snrpb; Snrpa; Prpf6; Naa38
ESC Pluripotency Pathways	-	7	7	114	100.00%	-	0.32	-
G1 to S cell cycle control	-	7	7	64	100.00%	-	0.32	-
IL-4 signaling Pathway	-	7	7	63	100.00%	-	0.32	-
IL-5 Signaling Pathway	-	7	7	70	100.00%	-	0.32	-
Nuclear receptors in lipid metabolism and toxicity	-	7	7	40	100.00%	-	0.32	-
Nuclear Receptors	-	7	7	38	100.00%	-	0.32	-

Proteasome Degradation	-	7	7	62	100.00%	-	0.32	-
Senescence and Autophagy	-	7	7	100	100.00%	-	0.32	-
Synaptic vesicle cycle	mmu04721	-	-	-	-	7	-	Napa; Dnm2; Stxbp1; Syt1; Slc17a8; Ap2s1; Unc13a
Salmonella infection	mmu05132	-	-	-	-	7	-	Pkn2; Dync2h1; Mapk11; Tjp1; Mapk13; Klc4; Wasf1
Insulin secretion	mmu04911	-	-	-	-	7	-	Adcy6; Adcyap1r1; Plcb2; Plcb4; Creb3l2; Adcy1; Kcnn1
Amyotrophic lateral sclerosis (ALS)	mmu05014	6	6	72	100.00%	6	0.29	Gria2; Grin1; Ppp3cb; Mapk11; Tnfrsf1a; Mapk13
Arrhythmogenic right ventricular cardiomyopathy (ARVC)	mmu05412	6	6	80	100.00%	4	0.29	Itga1; Cacnb1; Ctnna1; Ctnna2
Bacterial invasion of epithelial cells	mmu05100	6	6	95	100.00%	6	0.29	Ctnna1; Ctnna2; Dnm2; Pxn; Shc4; Wasf1
Bile secretion	mmu04976	6	6	95	100.00%	6	0.29	Adcy6; Abcc4; Abcg5; Abcb11; Adcy1; Abcc3
Cardiac muscle contraction	mmu04260	6	6	87	100.00%	4	0.29	cytochrome c oxidase subunit 7C, mitochondrial-like; Cacnb1; Cox6a1; Cox7c
Drug metabolism - cytochrome P450	mmu00982	6	6	176	100.00%	3	0.29	Cyp1a2; Fmo4; Cyp2c54
Endometrial cancer	mmu05213	6	6	60	100.00%	6	0.29	Araf; Ctnna1; Ctnna2; Egfr; Hras1; Kras
ErbB signaling pathway	mmu04012	6	6	99	100.00%	6	0.29	Araf; Egfr; Hras1; Kras; Stat5b; Shc4
Hematopoietic cell lineage	mmu04640	6	6	86	100.00%	4	0.29	Itga1; Cd33; Csf2ra; Tpo
Hypertrophic cardiomyopathy (HCM)	mmu05410	6	6	93	100.00%	4	0.29	Itga1; Ace; Cacnb1; Tgfb1
Leishmaniasis	mmu05140	6	6	84	100.00%	5	0.29	Ncf2; Mapk11; Tgfb1; Mapk13; Irak4
Oxidative phosphorylation	mmu00190	6	6	248	100.00%	5	0.29	cytochrome c oxidase subunit 7C, mitochondrial-like; Cox6a1; Cox7c; ND1; Atp5g2
Parkinson's disease	mmu05012	6	6	169	100.00%	5	0.29	cytochrome c oxidase subunit 7C, mitochondrial-like; Cox6a1; Cox7c; ND1; Atp5g2
RNA degradation	mmu03018	6	6	93	100.00%	6	0.29	Pan2; Ttc37; Pan3; Dis3; Naa38; Cnot10
Salivary secretion	mmu04970	6	6	101	100.00%	5	0.29	Adcy6; Atp2b2; Plcb2; Plcb4; Adcy1
Complement and Coagulation Cascades	-	6	6	64	100.00%	-	0.29	-
Delta-Notch Signaling Pathway	mmu04330	6	6	85	100.00%	2	0.29	Notch2; Dtx2
Glycogen Metabolism	-	6	6	42	100.00%	-	0.29	-
Myometrial Relaxation and Contraction Pathways	-	6	6	161	100.00%	-	0.29	-
Amphetamine addiction	mmu05031	-	-	-	-	6	-	Gria2; Grin1; Ppp1ca; Ppp1cc; Ppp3cb; Creb3l2
Thyroid hormone synthesis	mmu04918	-	-	-	-	6	-	Adcy6; Plcb2; Plcb4; Creb3l2; Tpo; Adcy1
Ovarian steroidogenesis	mmu04913	-	-	-	-	6	-	Adcy6; Cyp1a1; Cyp2j5; Adcy1; Cyp2j9; Pla2g4d
Apoptosis	mmu04210	5	5	90	100.00%	5	0.27	Birc3; Ppp3cb; Tnfrsf1a; Irak4; Map3k14
B cell receptor signaling pathway	-	5	5	86	100.00%	-	0.27	-
DNA replication	mmu03030	5	5	48	100.00%	5	0.27	Lig1; Mcm2; Mcm7; Rnaseh2b; Rfc5
Endocrine and other factor-regulated calcium reabsorption	mmu04961	5	5	67	100.00%	5	0.27	Adcy6; Dnm2; Plcb2; Plcb4; Ap2s1

Fat digestion and absorption	mmu04975		5	5	63	100.00%		4	0.27	Apoa4; Npc1l1; Pla2g2e; Abcg5
Metabolism of xenobiotics by cytochrome P450	mmu00980		5	5	159	100.00%		4	0.27	Cbr2; Cyp1a1; Cyp1a2; Cyp2c54
N-Glycan biosynthesis	mmu00510		5	5	96	100.00%		4	0.27	Man2a1; Man1b1; Alg5; 4933434I20Rik
NOD-like receptor signaling pathway	mmu04621		5	5	74	100.00%		5	0.27	Birc3; Mapk11; Mapk13; Card9; Mefv
Renal cell carcinoma	mmu05211		5	5	80	100.00%		5	0.27	Araf; Hras1; Kras; Tgfb1; Cul2
Taste transduction	mmu04742		5	5	72	100.00%		5	0.27	Adcy6; Plcb2; Tas2r136; Tas2r143; Tas1r2
Amino Acid metabolism	-		5	5	205	100.00%	-		0.27	-
DNA Replication	-		5	5	47	100.00%	-		0.27	-
Electron Transport Chain	-		5	5	116	100.00%	-		0.27	-
estrogen signalling	-		5	5	76	100.00%	-		0.27	-
Hypothetical Network for Drug Addiction	-		5	5	36	100.00%	-		0.27	-
Inflammatory Response Pathway	-		5	5	32	100.00%	-		0.27	-
Tryptophan metabolism	mmu00380		5	5	48	100.00%		2	0.27	Cyp1a1; Cyp1a2
Transcriptional misregulation in cancer	mmu05202	-	-	-	-			5	-	Birc3; Bcl2a1a; Mpo; Pparg; Zbtb17
GABAergic synapse	mmu04727	-	-	-	-			5	-	Adcy6; Gabra1; Gabrd; Plcl1; Adcy1
Morphine addiction	mmu05032	-	-	-	-			5	-	Adcy6; Gabra1; Gabrd; Grk4; Adcy1
Nicotine addiction	mmu05033	-	-	-	-			5	-	Gabra1; Gabrd; Gria2; Grin1; Slc17a8
HIF-1 signaling pathway	mmu04066	-	-	-	-			5	-	Egfr; Eif4e; Pfkfb3; Eif4e2; Cul2
Acute myeloid leukemia	mmu05221		4	4	64	100.00%		4	0.24	Araf; Hras1; Kras; Stat5b
Antigen processing and presentation	mmu04612		4	4	85	100.00%		3	0.24	Ctsl; Psme1; Psme2
Base excision repair	mmu03410		4	4	47	100.00%		3	0.24	Lig1; Parp3; Mutyh
Carbohydrate digestion and absorption	mmu04973		4	4	76	100.00%		7	0.24	Amy2a4; Amy2a3; Amy2a2; Amy2a5; G6pc; Plcb2; Tas1r2
Colorectal cancer	mmu05210		4	4	71	100.00%		4	0.24	Araf; Kras; Msh2; Tgfb1
Dorso-ventral axis formation	mmu04320		4	4	33	100.00%		4	0.24	Egfr; Kras; Notch2; Fmn2
Drug metabolism - other enzymes	mmu00983		4	4	100	100.00%		3	0.24	Itpa; Umps; Dpys
Gastric acid secretion	mmu04971		4	4	90	100.00%		4	0.24	Adcy6; Plcb2; Plcb4; Adcy1
Hedgehog signaling pathway	mmu04340		4	4	58	100.00%		3	0.24	Gli2; Gli3; Wnt6
Lysine degradation	mmu00310		4	4	137	100.00%		3	0.24	Whsc1l1; Plod3; Ehmt1
Mismatch repair	mmu03430		4	4	34	100.00%		4	0.24	Lig1; Msh2; Mlh3; Rfc5
Nucleotide excision repair	mmu03420		4	4	52	100.00%		4	0.24	Ercc2; Gtf2h1; Lig1; Rfc5
TGF-beta signaling pathway	mmu04350		4	4	89	100.00%		4	0.24	Bmp8a; Tgfb1; Cul1; Bambi
Blood Clotting Cascade	-		4	4	22	100.00%	-		0.24	-
cytochrome P450	-		4	4	49	100.00%	-		0.24	-
GPCRs, Class A	-		4	4	235	100.00%	-		0.24	-

Rhodopsin-like									
IL-9 Signaling Pathway	-		4	4	24	100.00%	-	0.24	-
MAPK Cascade	-		4	4	32	100.00%	-	0.24	-
One Carbon Metabolism	mmu01200		4	4	49	100.00%	1	0.24	Cps1
p38 MAPK Signaling Pathway (BioCarta)	-		4	4	34	100.00%	-	0.24	-
Retinol metabolism	-		4	4	50	100.00%	-	0.24	-
Splicing factor NOVA regulated synpatic proteins	-		4	4	45	100.00%	-	0.24	-
Pertussis	mmu05133	-	-	-	-		4	-	Mapk11; Mapk13; Irak4; Gm5077
Fanconi anemia pathway	mmu03460	-	-	-	-		4	-	Fancm; Top3a; Brip1; Mus81
Alanine, aspartate and glutamate metabolism	mmu00250		3	3	97	100.00%	3	0.21	Aspa; Adsl; Cps1
Aldosterone-regulated sodium reabsorption	mmu04960		3	3	55	100.00%	1	0.21	Kras
Basal cell carcinoma	mmu05217		3	3	62	100.00%	3	0.21	Gli2; Gli3; Wnt6
Glycerolipid metabolism	mmu00561		3	3	113	100.00%	3	0.21	Dgka; Gk2; Agpat3
Homologous recombination	mmu03440		3	3	49	100.00%	3	0.21	Top3a; Mus81; Xrcc3
Intestinal immune network for IgA production	mmu04672		3	3	52	100.00%	3	0.21	Pigr; Tgfb1; Map3k14
Malaria	mmu05144		3	3	72	100.00%	3	0.21	Klra4; Tgfb1; Thbs2
mTOR signaling pathway	mmu04150		3	3	64	100.00%	3	0.21	Eif4e; Eif4e2; Eif4b
Nicotinate and nicotinamide metabolism	mmu00760		3	3	108	100.00%	3	0.21	Nt5c3; Nt5c; Nt5c2
Peroxisome	mmu04146		3	3	87	100.00%	3	0.21	Amacr; Acsl4; Pex11g
Porphyrin and chlorophyll metabolism	mmu00860		3	3	231	100.00%	3	0.21	Alad; Urod; Ftmt
Primary immunodeficiency	mmu05340		3	3	38	100.00%	3	0.21	Aire; Jak3; Lck
Renin-angiotensin system	mmu04614		3	3	27	100.00%	2	0.21	Ace; Ren1
Ribosome	mmu03010		3	3	183	100.00%	5	0.21	Gm4705; Rpl28; Mrpl2; Rpl34-ps1; Rpl34
Steroid hormone biosynthesis	mmu00140		3	3	171	100.00%	3	0.21	Cyp1a1; H2-Ke6; Srd5a1
Thyroid cancer	mmu05216		3	3	35	100.00%	3	0.21	Hras1; Kras; Pparg
Toll-like receptor signaling pathway	mmu04620		3	3	110	100.00%	3	0.21	Mapk11; Mapk13; Irak4
Tryptophan metabolism	-		3	3	174	100.00%	-	0.21	-
Type II diabetes mellitus	mmu04930		3	3	60	100.00%	2	0.21	Socs3; Irs2
Vasopressin-regulated water reabsorption	mmu04962		3	3	48	100.00%	3	0.21	Dync2h1; Adcy6; Creb3l2
ACE Inhibitor Pathway	-		3	3	13	100.00%	-	0.21	-
Circadian Exercise	-		3	3	49	100.00%	-	0.21	-
Diurnally regulated genes with circadian orthologs	-		3	3	48	100.00%	-	0.21	-
ErbB signaling pathway	-		3	3	47	100.00%	-	0.21	-

Eukaryotic Transcription Initiation	-		3	3	41	100.00%	-		0.21	-
Fatty Acid Beta Oxidation	-		3	3	46	100.00%	-		0.21	-
Fatty Acid Biosynthesis	-		3	3	26	100.00%	-		0.21	-
G13 Signaling Pathway	-		3	3	38	100.00%	-		0.21	-
GPCRs, Other	-		3	3	210	100.00%	-		0.21	-
Hedgehog Signaling Pathway	-		3	3	22	100.00%	-		0.21	-
Leptin Insulin Overlap	-		3	3	20	100.00%	-		0.21	-
Mitochondrial Gene Expression	-		3	3	23	100.00%	-		0.21	-
Oxidative phosphorylation	-		3	3	65	100.00%	-		0.21	-
Peptide GPCRs	-		3	3	70	100.00%	-		0.21	-
Signaling of Hepatocyte Growth Factor Receptor	-		3	3	34	100.00%	-		0.21	-
TGF Beta Signaling Pathway	-		3	3	52	100.00%	-		0.21	-
Triacylglyceride Synthesis	-		3	3	26	100.00%	-		0.21	-
Wnt Signaling Pathway and Pluripotency	-		3	3	98	100.00%	-		0.21	-
Wnt Signaling Pathway	-		3	3	61	100.00%	-		0.21	-
Glycosaminoglycan biosynthesis - chondroitin sulfate / dermatan sulfate	mmu00532	-	-	-	-			3	-	Csgalnact1; Ust; Chsy3
Cocaine addiction	mmu05030	-	-	-	-			3	-	Gria2; Grin1; Creb3l2
Legionellosis	mmu05134	-	-	-	-			3	-	Hsf1; Sec22b; Hbs1l
Chemical carcinogenesis	mmu05204	-	-	-	-			3	-	Cyp1a1; Cyp1a2; Cyp2c54
Glycosaminoglycan biosynthesis - heparan sulfate / heparin	mmu00534	-	-	-	-			3	-	Ndst2; Hs6st2; Ndst3
African trypanosomiasis	mmu05143		2	2	57	100.00%		2	0.17	Plcb2; Plcb4
alpha-Linolenic acid metabolism	mmu00592		2	2	73	100.00%		2	0.17	Pla2g2e; Pla2g4d
Arginine and proline metabolism	mmu00330		2	2	223	100.00%		2	0.17	Nags; Cps1
Basal transcription factors	mmu03022		2	2	50	100.00%		2	0.17	Ercc2; Gtf2h1
Caffeine metabolism	mmu00232		2	2	39	100.00%		1	0.17	Cyp1a2
Circadian rhythm - mammal	mmu04710		2	2	26	100.00%		2	0.17	Per3; Cul1
Cysteine and methionine metabolism	mmu00270		2	2	148	100.00%		1	0.17	Bhmt
Galactose metabolism	mmu00052		2	2	100	100.00%		2	0.17	G6pc; Gaa
Glycolysis / Gluconeogenesis	mmu00010		2	2	120	100.00%		2	0.17	G6pc; Pck2

Glycosaminoglycan biosynthesis - chondroitin sulfate	-	2	2	24	100.00%	-	0.17	-
Mineral absorption	mmu04978	2	2	65	100.00%	2	0.17	Slc34a2; Ftmt
Mucin type O-Glycan biosynthesis	mmu00512	2	2	46	100.00%	2	0.17	Galnt3; Galnt5
Nitrogen metabolism	mmu00910	2	2	101	100.00%	2	0.17	Cps1; Car12
Notch signaling pathway	-	2	2	54	100.00%	-	0.17	-
Pantothenate and CoA biosynthesis	mmu00770	2	2	68	100.00%	2	0.17	Vnn1; Dpys
Primary bile acid biosynthesis	mmu00120	2	2	67	100.00%	2	0.17	Cyp27a1; Amacr
Prion diseases	mmu05020	2	2	45	100.00%	1	0.17	Fyn
Pyruvate metabolism	mmu00620	2	2	131	100.00%	2	0.17	Acot12; Pck2
Regulation of autophagy	mmu04140	2	2	53	100.00%	2	0.17	Pik3c3; Becn1
Rheumatoid arthritis	mmu05323	2	2	97	100.00%	2	0.17	Ctsl; Tgfb1
SNARE interactions in vesicular transport	mmu04130	2	2	37	100.00%	1	0.17	Sec22b
Sphingolipid metabolism	mmu00600	2	2	84	100.00%	3	0.17	Sptlc1; Neu3; Cers5
Steroid biosynthesis	mmu00100	2	2	86	100.00%	2	0.17	Tm7sf2; Dhcr24
Systemic lupus erythematosus	mmu05322	2	2	165	100.00%	2	0.17	Snrpb; Gm5077
Tyrosine metabolism	mmu00350	2	2	164	100.00%	1	0.17	Tpo
Viral myocarditis	mmu05416	2	2	103	100.00%	2	0.17	Fyn; Eif4g1
Vitamin digestion and absorption	mmu04977	2	2	77	100.00%	2	0.17	Apoa4; Folh1
Apoptosis	-	2	2	84	100.00%	-	0.17	-
Cytokines and Inflammatory Response (BioCarta)	-	2	2	31	100.00%	-	0.17	-
Cytoplasmic Ribosomal Proteins	-	2	2	82	100.00%	-	0.17	-
Dopminergic Neurogenesis	-	2	2	31	100.00%	-	0.17	-
Eicosanoid Synthesis	-	2	2	36	100.00%	-	0.17	-
EPO Receptor Signaling	-	2	2	26	100.00%	-	0.17	-
Estrogen metabolism	-	2	2	29	100.00%	-	0.17	-
Fatty Acid Omega Oxidation	-	2	2	9	100.00%	-	0.17	-
GPCRs, Class B Secretin-like	-	2	2	23	100.00%	-	0.17	-
Heme Biosynthesis	-	2	2	21	100.00%	-	0.17	-
Id Signaling Pathway	-	2	2	53	100.00%	-	0.17	-
IL-1 Signaling Pathway	-	2	2	38	100.00%	-	0.17	-
Matrix Metalloproteinases	-	2	2	30	100.00%	-	0.17	-
Mismatch repair	-	2	2	9	100.00%	-	0.17	-
Notch Signaling Pathway	-	2	2	47	100.00%	-	0.17	-

Nucleotide Metabolism	-		2	2	36	100.00%	-	0.17	-
Ovarian Infertility Genes	-		2	2	32	100.00%	-	0.17	-
Oxidative Stress	-		2	2	29	100.00%	-	0.17	-
Prostaglandin Synthesis and Regulation	-		2	2	40	100.00%	-	0.17	-
selenium	-		2	2	88	100.00%	-	0.17	-
serotonin and anxiety	-		2	2	23	100.00%	-	0.17	-
SIDS Susceptibility Pathways	-		2	2	67	100.00%	-	0.17	-
Statin Pathway	-		2	2	23	100.00%	-	0.17	-
Striated Muscle Contraction	-		2	2	45	100.00%	-	0.17	-
Toll Like Receptor signaling	-		2	2	33	100.00%	-	0.17	-
Urea cycle and metabolism of amino groups	-		2	2	37	100.00%	-	0.17	-
Inflammatory bowel disease (IBD)	mmu05321	-	-	-	-		2	-	Stat4; Tgfb1
Biosynthesis of amino acids	mmu01230	-	-	-	-		2	-	Nags; Cps1
Other types of O-glycan biosynthesis	mmu00514	-	-	-	-		2	-	Plod3; Chst10
Other glycan degradation	mmu00511	-	-	-	-		2	-	Man2b1; Neu3
mRNA processing	-		44	45	552	97.78%	-	-0.49	-
TNF-alpha NF-kB Signaling Pathway	-		16	17	185	94.12%	-	-1.6	-
Neuroactive ligand-receptor interaction	mmu04080		14	15	357	93.33%	14	-1.76	Adcyap1r1; C3ar1; Gabra1; Gabrd; Gria2; Grin1; Grm1; Lepr; Ntsr1; Thrb; Prss2; Hrh4; Rxfp1; Chrna10
mRNA surveillance pathway	mmu03015		13	14	98	92.86%	13	-1.86	Ppp1ca; Ppp1cc; Eif4a3; Papolg; Ppp2r5d; Rngtt; Ppp2r5c; Cpsf6; Cpsf2; Hbs1l; Fip1l1; Dazap1; Wibg
Androgen Receptor Signaling Pathway	-		11	12	114	91.67%	-	-2.07	-
Pancreatic secretion	mmu04972		9	10	127	90.00%	9	-2.34	Amy2a4; Adcy6; Atp2a1; Atp2b2; Plcb2; Plcb4; Prss2; Pla2g2e; Adcy1
Protein processing in endoplasmic reticulum	mmu04141		8	9	189	88.89%	10	-2.5	Vimp; Eif2s1; Dnaja1; Hsph1; Ufd1l; Man1b1; Ubqlnl; Cul1; Sec23b; Dnaja2
Glycerophospholipid metabolism	mmu00564		7	8	162	87.50%	8	-2.7	Dgka; Lcat; Chpt1; Pla2g2e; Agpat3; Cdipt; Pgs1; Pla2g4d
Protein digestion and absorption	mmu04974		6	7	115	85.71%	8	-2.92	Slc8a3; Col4a1; Col4a3; Col9a1; Col1a2; Dpp4; Prss2; Col24a1
Translation Factors	-		6	7	51	85.71%	-	-2.92	-
Amino sugar and nucleotide sugar metabolism	mmu00520		3	4	216	75.00%	4	-4.04	Pgm3; Renbp; Gnpda2; Gmppa
Ether lipid metabolism	mmu00565		3	4	75	75.00%	4	-4.04	Pafah2; Chpt1; Pla2g2e; Pla2g4d

The analysis of metabolic maps using KEGG and PathVisio(WikiPathways) pathway maps showed that a substantial number of metabolic processes could be modulated by AKI. The statistically most significant metabolic pathways are in lipid metabolism (arachidonic acid metabolism), nucleotide metabolism (purine and pyrimidine pathways), and carbohydrate metabolism (glucose). Signalling pathways which appear to be significant in terms of number of proteins up-regulated in these are, as analysed by independent methods previously and listed above, WNT signalling (leading to cell cycle activation and/or proteolysis), PPAR pathway (leading to activation of genes involved in differentiation, gluconeogenesis and fatty acid oxidation), VEGF signalling (leading to actin reorganisation), NFkappaB pathway (leading to immune response), chemokine signalling (leading to cytokine production, apoptosis and regulation of the actin cytoskeleton), ErbB-mediated gene activation (leading to migration/invasion, adhesion, differentiation, etc.), T-cell receptor pathway (leading to proliferation, differentiation and immune-response), MAPK signalling pathway (from TGFB, EGF, FGF and TrkA/B), JAK-STAT signalling (from cytokine signalling to ubiquitin-mediated proteolysis), TGFbeta- and ubiquitin-mediated proteolysis, endosomal pathway, insulin signalling pathway (leading to glucose uptake, lipid homeostasis, proliferation and MAPK signalling), A potentially linked pathway from polysugar uptake and glycogen degradation to D-glucose production, by bridging via the glucoronate interconversion pathway to inositol-signalling (leading to cell proliferation/adhesion and regulation of the actin cytoskeleton), and lastly the calcium signalling (via glutamate signalling pathway, leading to MAPK-signalling and apoptosis).

Metabolic reactions

UniProt, BRENDA, MetaCyc and Reactome databases wee searched for enzymatic reactions using initially the enzymes listed in the AKI significant dataset, and then specifically for metabolites to find and map missing protein entries that can potentially bridge existing gaps between already mapped metabolites. A substantial amount of metabolic reaction information could also be extracted from the pre-assembled pathway maps using PathVisio(WikiPathways). All reactions (enzymes, cofactors and metabolites) thus found were placed and connected in the global map assembled in PathVisio.

Transcriptional events

An important aspect in any disease is the involvement of gene modulatory events, whether to uncover if they are functioning in an unexpected way, or just to identify how they fit into a global pathway map and sequence of events. For this, the dataspace was mapped to identify potential transcription factors and, by using various databases such as GeneCards and the literature, genes which potentially could be modulated by them were identified. Additionally, the data-sources were also searched to uncover specific transcriptional elements which can control the expression of key molecules in the AKI dataset as well as potentially drive a set of genes that comprise a functional group (i.e. PPARγ and fatty acid involved genes). Any such information was also placed/added to the global map.

Listing and delineation of hallmarks

Another focus was placed in delineating and to explain, on a molecular-mechanistic level, pathways known to be involved or associated with AKI. For this, the literature was first searched to assemble a list aof hallmarks followed by searching for mechanistic explanations (see also Table 1 in the main body of the manuscript). Quite a number of hallmarks are relatively simple to delineate in terms of involved molecular events. At the same time some, such as hyperglycaemia and elevated blood pressure, were found to be sparsely described, or the published literature offered explanations which were inconsistent. Figure E5 shows an example of a delineation process, where both hyperglycaemia and blood pressure modulation involves arachidonic acid and specifically its downstream metabolite 20-hydroxyeicosatetraenoic acid (20-HETE), and involving P450 proteins. Arachidonic acid, a potent modulator of Src-family kinases, can be produced by a variety of phospholipases, which in turn can be triggered through activation of the angiotensin system and other factors. Further literature searching also shows that arachidonic acid and 20-HETE are apparent direct contributors to nephropathies under hyperglycaemic conditions (see e.g. PMID: 9005967 and 23936373). Another arachidonic acid derived metabolite is another eicanosid 12(S)-HETE. Diabetes is known to lead to an over-production of this compound, which is a direct consequence of hyperglycaemia and the angiotensin-activated AT(1)-receptor (PMID:18799632). Gathering of such information allowed us to position the observed P450 proteins in our dataset into the

global molecular pathway. It it also apparent that other alternative chains of events using other P450 molecules could potentially lead to the same outcome.

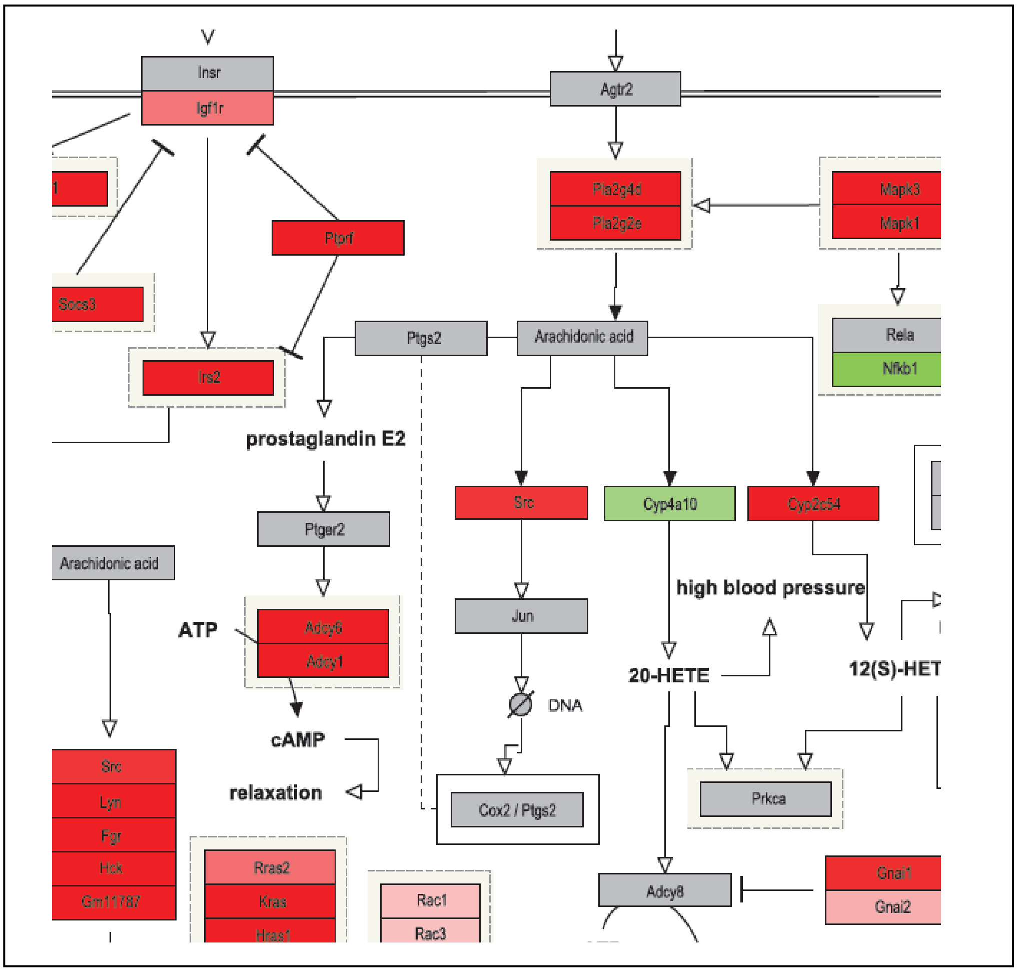

Figure E5. Involvement of arachidonic acid in blood pressure modulation and other molecular events. The literature was searched for ‘arachidonic acid’ including additional keywords such as ‘hyperglycaemia’, ‘elevated blood pressure’ and ‘Src’ and the information thus obtained was put into a molecular concept and placed into the global molecular map.

<u>Extending the knowledgebase/Literature searching/Pharmacological intervention</u>

The literature was searched mainly via PubMed using specific keywords pertinent to the pathway/molecule/module under investigation or a combination of terms. All data usefull in explaining the molecular events surrounding AKI or the pathways of interest found by this manual data trawling were incorporated into the global pathway map. Mapping of pharmacological interventions (see Figure 3 in the manuscript) was done in this way where known chemical compounds used to modulate or interfere

with AKI-effects found in the literature were placed into the context model of the global pathway.

Assembly of a global map of AKI modulatory cascades

All the analyses described above were then further explored by integrating the results into a global molecular pathway map. The GO analyses allowed to pinpoint potential molecular events occurring in AKI, and the interactome analysis provided a physical assembly of direct and indirect clusters. Pathway analysis involving signalling, metabolic and transcriptional cascades allowed to initially lay out the various molecular modules, which were then inter-connected where possible. This process of resampling, iterative mapping, delineation and pathway placement was repeated until an acceptable global pathway map could be established (see Figure E6 below). Simultaneously, other proteins which are known to catalyse the same reactions, or in case of multi-protein complexes together form a functional entity, than those found in the high stringency dataset were also added to allow for a better understanding of molecular events, as well as serve as contingency for cross-connections where orthologs may have been analysed and mentioned in the literature, and also to allow for greater flexibility when assembling the final global map.

Data reduction, streamlining and pathway clustering

The high stringency dataset was then mapped onto the global map, and any alternative protein which was not part of this dataset and performing the same reaction than a mapped protein was removed. Whole pathways which were either unsupported, showed sparse mapping or contradictory expression levels in the same chain of events were also removed and the remaining cascades rearranged and re-connected. This resulted in the pathway model shown in Supplementary figure S1. Focussing on the hallmarks resulted in Figure 3 shown in the main body of text, and resampling and further condensation of the molecular events based on labelled pathways is shown in Figure 4 shown in the main text. The specific pathway involving the NMDA receptor is in Figure 2B in the main body of the text.

Figure E6. Global molecular map of events associated with AKI. Signalling cascades, metabolic reactions and transcriptional events were assembled using a Systems Biology approach involving extensive literature searches and mining of various databases. Molecular events, at this stage, were associated with any molecule which can perform the specific reactions, and mapping (coloration) was the pathway map was done using the entire dataspace of all molecules observed in the AKI study.

Pathways in Acute Kidney Injury (AKI)

species: mouse

© Holger Husi 2013

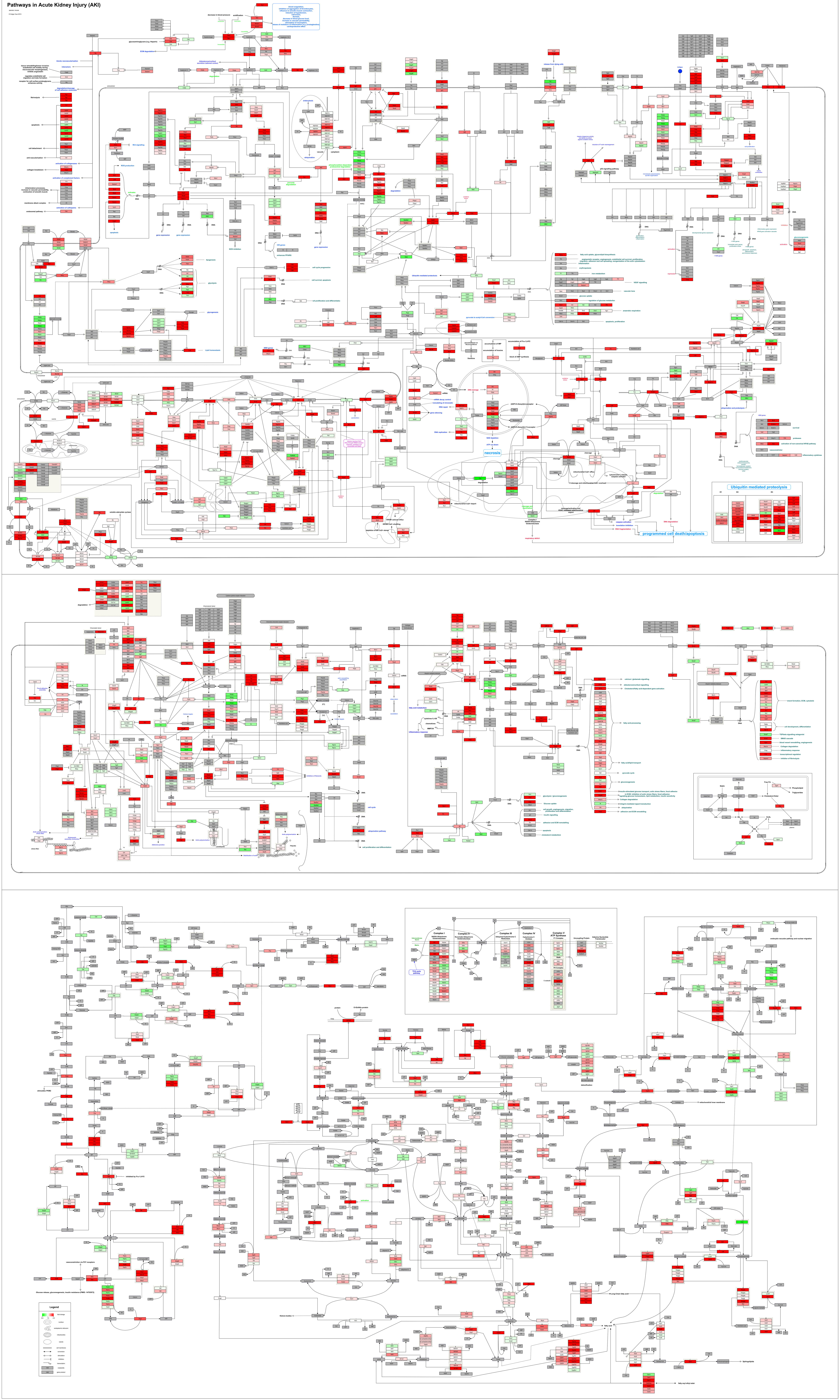

Supplement: Additional file 2 — This file contains in-depth methods and results used to generate the molecular pathway map. It also contains the results of GO, interactome and KEGG pathway mapping analysis, as well as the original assembly of pathway cascades used to establish the global pathway shown in Additional file 3: Figure S1. [file 1752-0509-7-110-S2.pdf]
